# Generative AI for efficient statistical computation of fluids

**DOI:** 10.1038/s41467-026-76390-x

**Published:** 2026-08-17

**Authors:** Bogdan Raonić, Roberto Molinaro, Samuel Lanthaler, Tobias Rohner, Victor Armegioiu, Stephan Simonis, Dana Grund, Yannick Ramic, Zhong Yi Wan, Fei Sha, Siddhartha Mishra, Leonardo Zepeda-Núñez

**Affiliations:** 1 Seminar for Applied Mathematics, Department of Mathematics, ETH Zurich, Zurich, Switzerland; 2 ETH AI Center, Zurich, Switzerland; 3 Jua.ai, Zurich, Switzerland; 4 University of Vienna, Vienna, Austria; 5 Institute for Applied and Numerical Mathematics, Karlsruhe Institute of Technology, Karlsruhe, Germany; 6 Department of Environmental Systems Science, ETH Zurich, Zurich, Switzerland; 7 Google Deepmind, Mountain View, CA USA; 8 Google Research, Mountain View, CA USA; 9 Present Address: Meta, Menlo Park, CA USA

**Keywords:** Computational science, Applied mathematics

## Abstract

We present GenCFD, a generative modeling approach for fast, accurate, and robust statistical computation of three-dimensional turbulent fluid flows. While motivated from conditional score-based diffusion models, GenCFD is both empirically and theoretically validated on generating turbulent flows. Extensive numerical experimentation of challenging three-dimensional fluids demonstrates that GenCFD provides an accurate approximation of relevant statistical quantities of interest while also efficiently generating high-quality realistic samples of such flows. Moreover, we present rigorous theoretical results on analytically tractable models with mathematically relevant features of turbulent fluid flows. The analysis uncovers the mechanism underlying the success of the diffusion modeling approach. In particular, we highlight the importance of modeling distributions by GenCFD, while the mean-square-loss used by the deterministic machine learning approaches fails to accurately characterize statistical features of chaotic dynamics.

## Abstract (web summary)

Turbulent fluid dynamics pose a formidable computational challenge: chaotic, multiscale Navier–Stokes solutions require large ensembles for reliable statistical estimates, making direct numerical simulation intractable for many high–Reynolds-number problems. This work demonstrates that generative AI models, specifically diffusion-based approaches, can efficiently produce statistically accurate samples of complex three-dimensional turbulent fluid flows, surpassing traditional deterministic neural surrogates that tend to regress to mean behavior.

## Introduction

From predicting the Earth’s climate and the paths of tsunamis to designing next-generation aircraft and understanding the flow of blood in the human body, the ability to model fluid motion is fundamental to modern science and engineering^1^. Yet, the study of fluid flows remains a formidable challenge, as they encompass a vast range of spatial and temporal scales with a rich phenomenology of states.

A central difficulty arises in flows at high Reynolds numbers (${{{\rm{Re}}}}$), a regime where inertial forces overwhelm viscous damping, causing the flow to evolve chaotically into states containing energetic, multiscale eddies^1^. This phenomenon, known as turbulence, is characterized by a high sensitivity to initial conditions and is famously considered one of the most important unsolved problems in classical physics^2^.

Fluids are mathematically described by (variants of) the Navier–Stokes equations, a system of nonlinear partial differential equations (PDEs) that lack general analytical solutions. Consequently, the field has come to rely on computational fluid dynamics (CFD), where numerical algorithms such as finite difference^3^, finite element^4^, and spectral methods^5^ are used to simulate flows in silico. The gold standard, direct numerical simulation (DNS), resolves all scales of motion but suffers from the (in)famous curse of dimensionality: its cost scales as ${{{{\rm{Re}}}}}^{3}$, rendering it prohibitive for the high-${{{\rm{Re}}}}$ flows common in science and engineering^6^. While alternative approaches like large eddy simulations (LES) reduce this cost by modeling the smallest turbulent eddies rather than resolving them directly^7^, they remain computationally prohibitive for many large-scale applications, such as global atmospheric simulations.

**Fig. 1:**
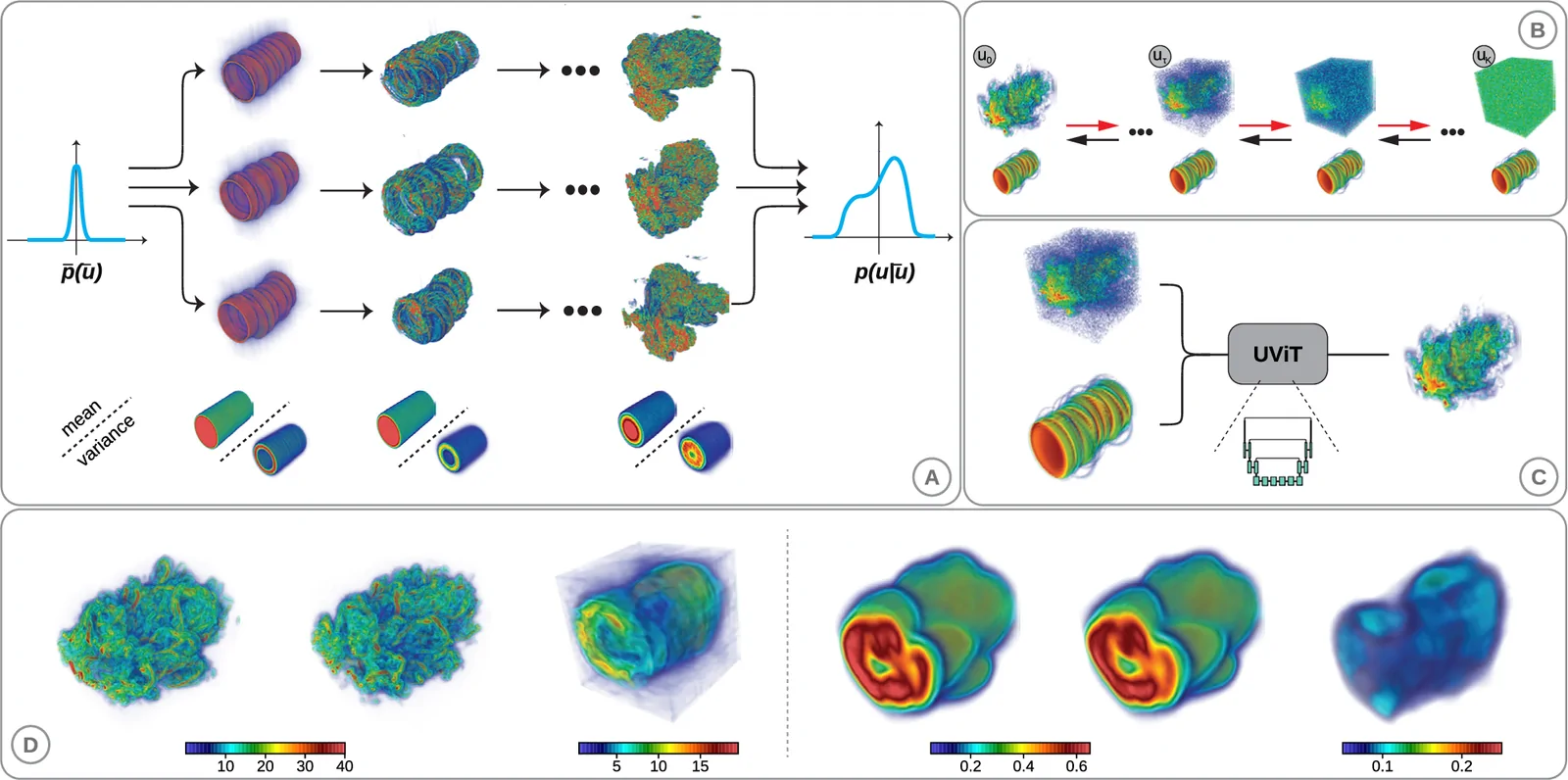
Visual summary of this article. **A** Our goal here is the statistical computation of a fluid flow, i.e., computing the push-forward of the distribution $\bar{p}(\bar{u})$ on the inputs (initial and boundary conditions) with respect to the solution operator ${{{\mathcal{S}}}}$ of a PDE to provide the target distribution $p(u| \bar{u})$, with $u={{{\mathcal{S}}}}(\bar{u})$ (at any desired time) as its output. In UQ for CFD, one draws samples $\bar{u} \sim \bar{p}$, evolves them in time with a CFD solver, and computes statistics such as mean and variance from samples $u \sim p(\cdot | \bar{u})$. ML algorithms simply replace the CFD solver with a neural network surrogate, trained to minimize the mean-square error with respect to each *u*. On the other hand, our method GenCFD is based on **B** a conditional diffusion model, which at inference (black arrows) generates $u \sim p(\cdot | \bar{u})$, given input $\bar{u} \sim \bar{p}$ and isotropic Gaussian noise *u*_*K*_, by solving the reverse SDE (6) backward in time. During training of the diffusion model, noise is added iteratively (red arrows) to transform any *u* ~ *p* to a noisy sample and **C** The denoiser (UViT for GenCFD) is a neural network that is trained to output a clean sample of the solution *u*, given input $\bar{u}$ and noise. The denoiser replaces the score function in the reverse SDE (6). **D** Results for the cylindrical shear flow dataset: individual realizations of the vorticity intensity (left sub-panel) and standard deviation of the pointwise kinetic energy (right sub-panel), respectively, at *T* = 1 for the ground truth (left), GenCFD (center), and an ML baseline C-FNO (right). The ground truth is generated by a DNS with a spectral hyperviscosity method. Please note the different ranges of the colorbars in (**D**). A detailed description of the methods and the datasets is provided in SI Sec. 2. Extensive numerical results are documented in SI Sec. 4.

Furthermore, the very nature of turbulence imposes a more fundamental limitation. Given the high sensitivity of fluid flows to small perturbations in inputs, such as initial and boundary conditions (see Fig. 1A), any single, deterministic simulation has limited predictive power^8,9^. For chaotic systems, the scientifically robust and engineering-relevant paradigm is statistical computation, often referred to as forward uncertainty quantification (UQ)^9^. This approach focuses on computing stable statistical quantities (e.g., mean, variance, etc.) over an ensemble of possible outcomes, which is imperative for reliable design and optimization^9^.

Alas, this creates a conundrum: to compute the desired stable statistics, UQ requires evolving a large ensemble of inputs, each with an already expensive CFD simulation. Although the cost grows linearly with the number of ensemble members, the slow (square-root) convergence of random sampling necessitates a very large ensemble for accurate estimates^9–11^, making the overall pipeline virtually intractable^9^. Overcoming this barrier to fast and accurate statistical computation of fluid flows represents a grand challenge in modern computational science^9^.

Recent advances in machine learning (ML) have offered a potential path forward, with neural network surrogates that can accelerate deterministic PDE solutions by orders of magnitude^12–18^. These methods are promising candidates to replace the expensive numerical solver in the UQ pipeline. Unfortunately, when these (deterministic) neural networks are trained to minimize standard metrics like mean-squared error, they learn to predict only the average behavior of the flow. As shown in Fig. 1D, this causes the predicted ensemble to collapse to an unphysical, smoothed-out mean, failing to capture the variance and the rich multiscale structure of the turbulent state^19,20^.

In response, a class of generative AI models leveraging techniques for image and video generation^21–26^ has recently emerged, aiming to learn the underlying probability distribution of the flow directly. Despite promising initial results (see Supplementary Information (SI) Table 1), their application to complex, real-world fluid dynamics has left three critical questions unanswered, which in return have hampered their application to downstream tasks. First, can generative models move beyond simplified two-dimensional cases with smoother solutions (see SI Sec. 1) to capture the full statistical complexity and spectral properties of high-Reynolds-number, three-dimensional turbulence? Second, can these models generate accurate conditional distributions, which are essential for engineering design and UQ, rather than generating samples of a flow under a single prior (see SI Sec. 1.3)? Third, how can a probabilistic model be rigorously justified for a system governed by deterministic equations, and what are the precise mechanisms that allow it to succeed where purely deterministic surrogates fail, even in the case of toy models?

This work provides positive answers to these questions by introducing GenCFD, a generative AI framework that:

1. generates realistic and statistically accurate samples of complex three-dimensional turbulent flows (see SI Sec. 2) measured using a comprehensive suite of statistical metrics (see SI Sec. 2.8 and SI Table 7), orders of magnitude faster than conventional CFD solvers (see SI Table 21);
2. produces accurate conditional distributions even when provided with a single sample per conditioning, for a variety of challenging problems (as shown in Fig. 1D), thus rendering it a practical tool for downstream scientific and engineering tasks; and
3. operates on a sound theoretical footing, which we elucidate with rigorous mathematical arguments applied to analytically tractable model systems (see SI Sec. 3) that uncover the precise mechanism by which a diffusion model provides accurate statistical computation for chaotic dynamical systems.

Thus, with GenCFD, we present a generative AI algorithm that can transform the simulation of fluid flows and has the potential to impact a wide range of downstream tasks in physics, climate science, and engineering.

## Results

### Accurate and efficient statistical computation

**Fig. 2:**
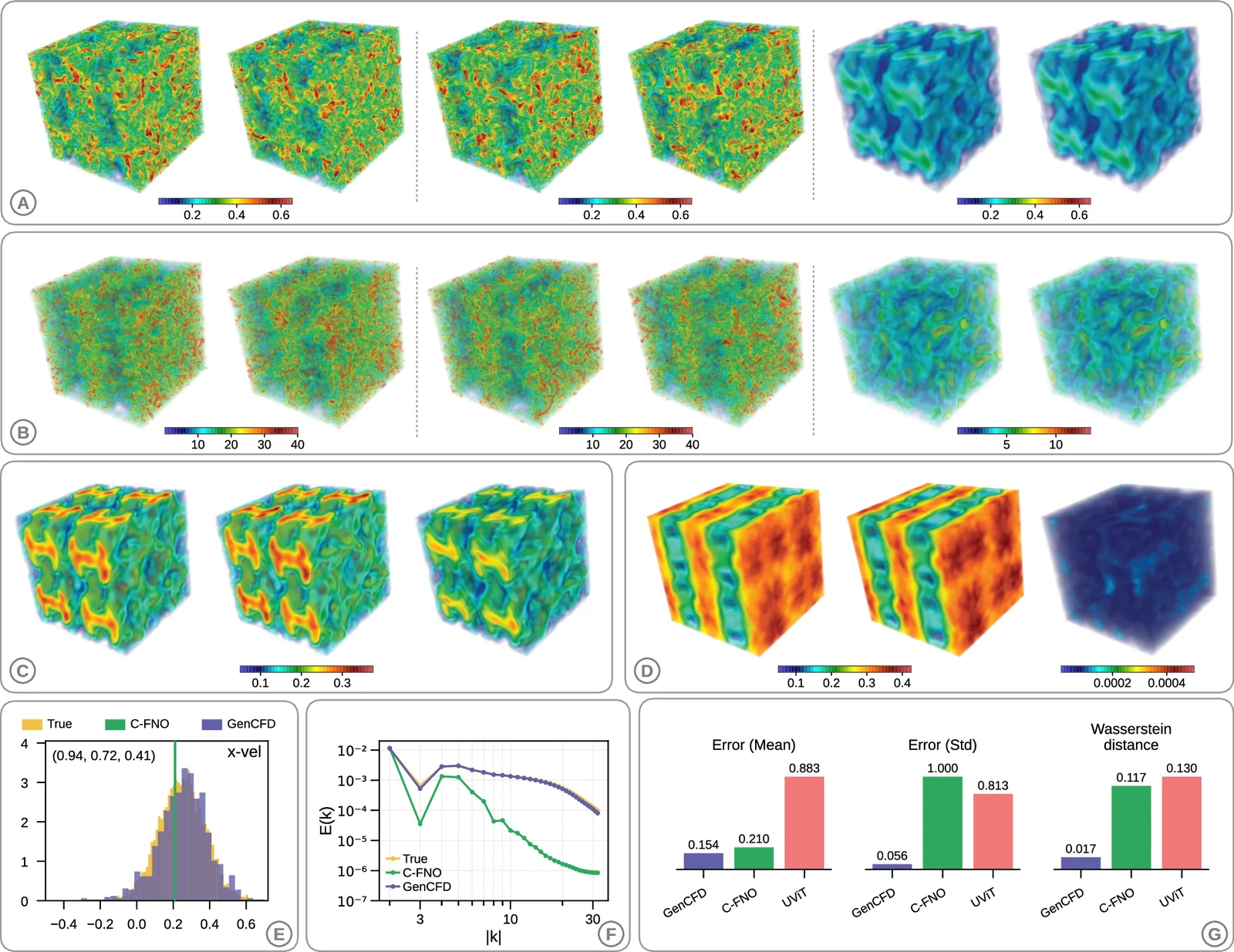
Results for the Taylor–Green vortex dataset. **A**, **B** Two Samples of the flow at time *T* = 2 for the same initial condition, respectively produced with ground truth (left), GenCFD (center), and C-FNO baseline (right) for the pointwise kinetic energy (top row) and vorticity intensity (bottom row). **C** Mean and **D** standard deviation, of the pointwise kinetic energy at time *T* = 2 with ground truth (left), GenCFD (center), and C-FNO (right). **E** PDF of the *x*-velocity component (*x*-vel) at the spatial point (0.94, 0.72, 0.41) and *T* = 2 and **F** energy spectrum *E*(*k*) over wavenumber ∣*k*∣ at *T* = 2. **G** Errors in predicting the mean (left), standard deviation (center) and 1 − Wasserstein distance (right) of the *x*-velocity component at time *T* = 2 with GenCFD, C-FNO, and UViT (error values are provided in SI Table 11). Ground truth is generated by a DNS with a spectral hyperviscosity method. Please note the different ranges of the colorbars in (**B**, **D**). A detailed description of the methods and the datasets is provided in SI Sec. 2. Extensive numerical results are documented in SI Sec. 4. Source data are provided as a Source Data file.

Besides generating high-quality, realistic samples (see Fig. 2A, B, F and SI Sec. 4.1), GenCFD accurately approximates statistical quantities such as the conditional mean (of the pointwise kinetic energy shown in Fig. 2C), the conditional variance (of the kinetic energy shown in Fig. 2D) and even conditional point PDFs (of the *x*-velocity component shown in Fig. 2E). GenCFD provides an accurate approximation to the variance of the flow, especially when compared to the baselines: the deterministic neural operators struggle to approximate the variance of the flow as the initial condition is (nearly) a Dirac measure. This behavior also holds for the *L*^2^ error of the higher-order central moments (up to order five) with respect to the ground truth: we observe that GenCFD accurately approximates the system response, whereas the baselines produce almost no response (see SI Fig. 29 and SI Table 17). Similarly, the point PDFs are well-spread out by this time (*T* = 2) and GenCFD approximates them accurately, whereas the generated PDF from the baselines collapses to a single point. These observations are reinforced by the quantitative results presented in Fig. 2G and SI Table 11. In Fig. 2G, we present the *L*^1^-errors in the mean and the standard deviation of the *x*-component of the velocity, as well as the spatially integrated 1-Wasserstein distance between the target distribution and the conditional distribution generated by GenCFD (see SI Sec. 2 for definitions).

Capturing the correct spectral behavior is fundamental in the study of turbulent fluids as energy cascades down to the smaller scales via a power law decay of the spectrum^1^. Figure 2F shows the energy spectrum for the ground truth, GenCFD, and the best-performing baseline (C-FNO), from which we observe that GenCFD approximates the energy spectrum (and its power law decay) accurately, all the way down to the smallest resolved scales, whereas the spectrum generated by C-FNO and other baselines is inaccurate as it presents a non-physical exponential decay. A more refined description of the energy cascade for turbulent fluid flows is provided by quantities such as structure functions. In SI Sec. 4.9, we show how GenCFD is able to accurately approximate finer statistical metrics such as the variation of the third-order longitudinal structure function of the underlying ground truth numerical simulation (see SI Fig. 5), further demonstrating the ability to learn the complex underlying physics of fluid flows.

In summary, GenCFD is one order of magnitude more accurate than the baselines at capturing the variance, energy spectrum, and higher-order moments; and approximating the ground truth conditional distribution in terms of Wasserstein distance. Error metrics for the Leray-projected velocity fields are provided in SI Table 10. On the other hand, the baselines trained to minimize the mean absolute (square) error are observed to regress to the mean and fail to capture the intrinsic statistical spread of the fluid flows considered here. Given similar observations in the weather and climate modeling community^19,20^, articles such as Lang et al.^27^ have proposed replacing MAE (MSE) minimization with training neural networks (operators) to minimize CRPS losses. We investigate this alternative training paradigm in SI Sec. 4.7 (see also SI Figs. 3–4 and SI Tables 8–9). While CRPS training does improve the statistical spread of baselines by adding uncorrelated noise to the mean, it is still an order of magnitude less accurate in terms of statistical metrics of the flow when compared to GenCFD, for our underlying learning tasks, which aim to learn statistical solutions at medium to long time scales, conditioned on the initial data distribution.

### Application to other fluid flows

GenCFD’s accurate generation of turbulent fluid flows extends to the other four flows considered here. For the three-dimensional cylindrical shear flow^11^, we observe exactly the same qualitative and quantitative results obtained for the Taylor–Green vortex, as shown in Fig. 1D where we present a sample of the vorticity intensity and the computed variance at time *T* = 1, for the ground truth, GenCFD, and C-FNO for a given initial condition (see also SI Figs. 7–11 and SI Tables 12, 19, and 20 for further results for this benchmark).

**Fig. 3:**
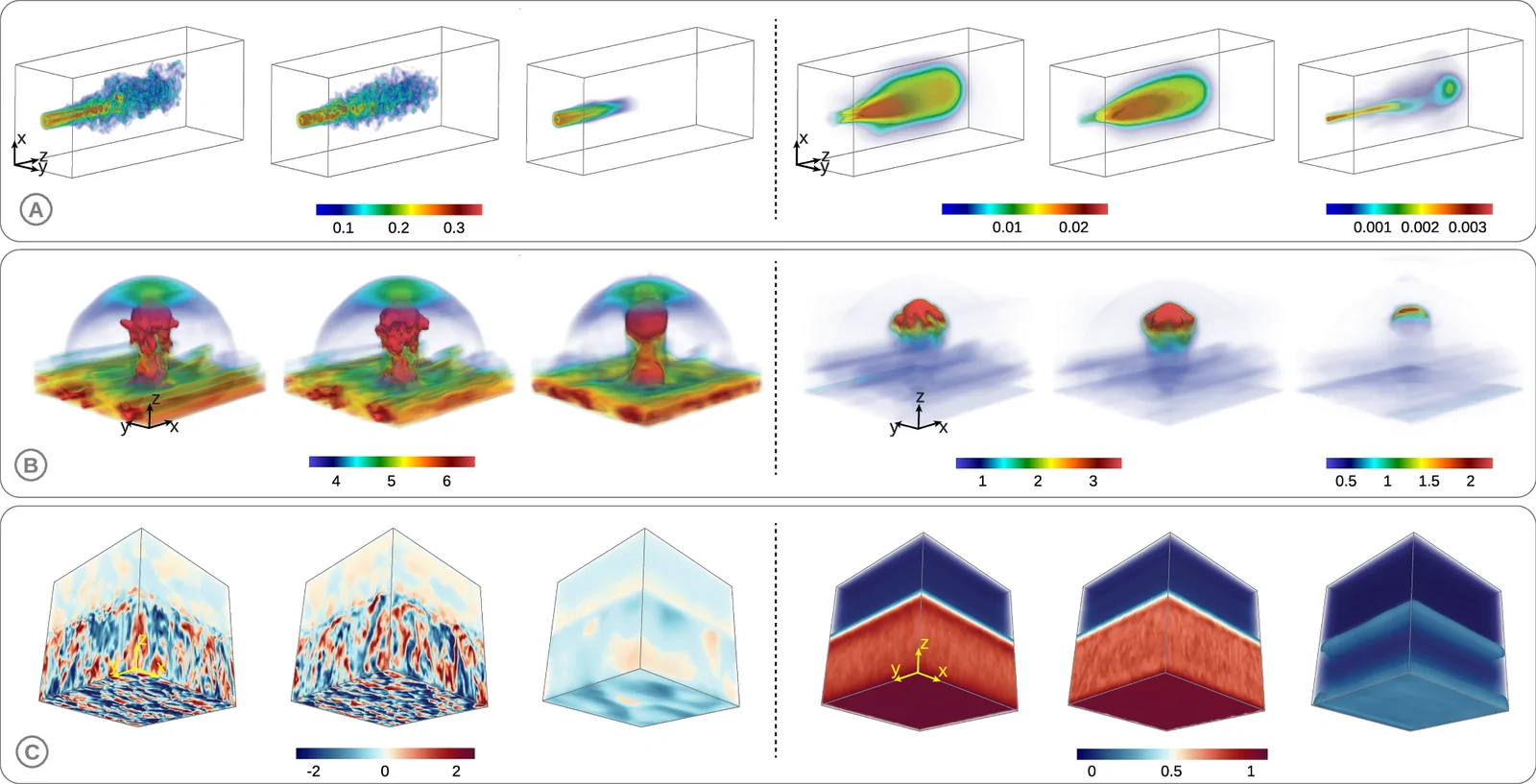
Results for the other flows: nozzle flow, cloud-shock interaction, and dry convective boundary layer. **A** A sample of the vorticity intensity (left sub-panel) and the standard deviation of the (pointwise) kinetic energy (right sub-panel) for the nozzle flow at time *T* = 1 with ground truth (left), GenCFD (center), and UViT (right). **B** A sample of the density (left sub-panel) and standard deviation of the density (right sub-panel) at time *T* = 0.6 for the cloud-shock interaction problem with the compressible Euler equations with ground truth (left), GenCFD (center), and C-FNO (right). **C** A sample of the *x*-component of the velocity (left sub-panel) and standard deviation of the *x*-velocity (right sub-panel) at time *T* = 2.4 for the dry convective planetary boundary layer dataset with ground truth (left), GenCFD (center), and UViT (right). The ground truth is generated by an LES with a lattice Boltzmann method (nozzle flow), a DNS with a high-resolution finite volume method (cloud-shock interaction), and an LES with a WENO finite difference method (convective planetary boundary layer), respectively. Please note the different ranges of the colorbars in Panels A and B (right subpanels). A detailed description of the methods and the datasets is provided in SI Sec. 2. Extensive numerical results are documented in SI Sec. 4.

In Fig. 3, we present a representative glimpse of the experimental results for the other three challenging datasets exhibiting different physics and boundary conditions while embodying different downstream applications. We start with a three-dimensional nozzle flow at a Reynolds number of up to ${{{\rm{Re}}}}=20000$ for the Navier–Stokes equations (see SI Sec. 2). This is a prototypical example of turbulent jet flows which are widely studied in engineering^6^. The simulated flow field differs from the other datasets in i) being both wall-bounded as well as having a freestream leading to ii) non-trivial wall boundary conditions in place of the previously considered periodic boundary conditions, and iii) the entire flow needs to be generated from a single scalar input, i.e., the injection velocity, which is in the form of a boundary condition rather than the initial condition as in the Taylor–Green vortex and cylindrical shear flow examples in Fig. 2 and Fig. 1 respectively. As visualized with a sample of (pointwise) vorticity intensity (see Fig. 3A), the flow, with the ground truth generated by an LES (see SI Sec. 2), consists of an energetic jet emanating from the inlet and evolving in a turbulent manner to an intricate collection of multiscale whirls and eddies further downstream. From Fig. 3A (see also SI Figs. 15–16), we observe that GenCFD is able to generate samples of this complex flow realistically, while the best-performing baseline (UViT in this case) fails to generate the small-scale features in the vorticity and collapses onto a (laminar) jet in the middle of the flow. Similarly, statistics of this complex flow are accurately approximated by GenCFD while the baselines fail to account for the variance (Fig. 3A). Further qualitative and quantitative results in SI Figs. 15–18 and 24, and SI Table 14, show that GenCFD accurately generates this complex flow from just a single scalar input outperforming the baselines that are, at best, only able to generate the mean behavior.

In Fig. 3B, we consider the three-dimensional cloud-shock interaction problem, which is a well-established benchmark for compressible fluid flows^28^ (see SI Sec. 2). As visualized with the density profile in Fig. 3B, an incoming supersonic shock wave hits a high-density cloud and leads to the excitation of shock waves while creating a zone of turbulent mixing in their wake. Even though the underlying equations (compressible Euler vs. incompressible Navier–Stokes, see SI Sec. 2.1) and the flow dynamics (see SI Sec. 2.6.3, presence of discontinuous shock waves) are very different from the previously considered examples, GenCFD is able to generate realistic flow samples, while also yielding accurate approximations of statistical quantities of interest (Fig. 3B and SI Figs. 12–14, 24 and SI Table 13). On the other hand, baselines such as C-FNO fail to capture the turbulent mixing zone, although the strong shock wave is accurately computed.

Finally, in Fig. 3C, we present results for the dry convective planetary boundary layer, a well-known benchmark in the atmospheric sciences^29^, heavily used for understanding the statistics of boundary layer flows and validating and calibrating turbulence models in meteorology. This atmospheric flow corresponds to the dynamics of air under the effect of a surface heat flux (modeling radiative heating through a summer day) and a weak, large-scale geostrophic wind which induces shear at the surface, leading to a complex combination of updraft and downdraft plumes driving (vertically) anisotropic turbulent motion (see Fig. 3C for a visualization of the *x*-component of velocity). Not only are the underlying PDEs (anelastic flow equations, see SI Sec. 2) different from the previous datasets, but the physics of this flow are far richer, due to the presence of heat transfer. Nevertheless, GenCFD generates high-quality samples of this flow and accurately approximates the variance, outperforming the baselines (Fig. 3C). We provide a detailed qualitative and quantitative analysis of this benchmark in the SI Figs. 19–22, 24 and SI Table 15, particularly for (horizontally averaged) statistics, which further showcase the performance of GenCFD. In particular, the results show the generalization capability of GenCFD as the training and test distributions are very different in this case (see SI Fig. 21 to visualize this distribution shift for horizontally averaged quantities).

As mentioned earlier, the (approximately) Dirac test distribution for all the datasets is different from the underlying training distribution, highlighting the ability of GenCFD to generalize. In addition, GenCFD is able to robustly generalize to unseen test distributions (see SI Sec. 4.5), either with no additional training (zero shot) or when fine-tuned with a few downstream samples (few shot), as shown in the SI (see SI Figs. 9–10, 27–28 and SI Table 18).

### Capturing complex temporal dynamics

GenCFD also captures the complex temporal dynamics of turbulent fluid flows, including transitions from laminar to turbulent regimes as shown in the SI Sec. 4, SI Figs. 25–28, and SI Table 16. In those figures and tables, we present samples and statistics for the Taylor–Green vortex at time *T* = 0.8, when the flow is still laminar and not yet turbulent, showcasing that GenCFD provides accurate samples and approximations to time-varying statistical quantities. We attribute this ability to our lead time conditioning and an all-to-all training procedure, which leverages the semi-group property of the underlying solution operator (see SI Sec. 2).

### Efficiency

GenCFD is sample efficient. Our benchmark suite is inherently out-of-distribution, given that the model has only seen one set of input-output pairs per input condition during training. Nevertheless, GenCFD is able to accurately capture the conditional distribution: it is able to generate a large diversity in samples, for the same input condition, as shown in SI Figs. 7, 8, 23, and 30–33 for the shear flow benchmark (see also Fig. 2A, B for the Taylor–Green vortex), showcasing the low sample-complexity of GenCFD in generating high-quality fluid flows.

GenCFD is computationally efficient. As shown in SI Table 21, GenCFD is able to generate samples with one to five orders of magnitude speedup over traditional solvers in generating fluid flows (see SI Table 21), using a consumer-grade GPU (NVIDIA RTX 4090). In particular, GenCFD can generate the benchmark fluid flows in less than 4 s, while it takes a standard CFD solver anywhere between minutes (on GPUs) and hours (on CPUs).

This speedup, coupled with its statistical accuracy, renders GenCFD well-suited for widespread downstream applications.

### Theoretical results

Why does GenCFD succeed in generating realistic fluid flows and accurately approximating their statistical and spectral behavior when baselines fail to do so? To address this question, we present theoretical arguments, based on rigorous mathematical analysis in the SI Sec. 3, with a heuristic summary here. Following SI Sec. 2 and for the setting considered here, the goal of statistical computation for a PDE with the (approximate) solution operator ${{{\mathcal{S}}}}$ and any initial datum $\bar{u}\in {{{\mathcal{X}}}}$, is to compute the conditional distribution corresponding to the law of the random variable ${{{{\rm{Law}}}}}_{\delta \bar{u}}{{{\mathcal{S}}}}(\bar{u}+\delta \bar{u})$, over randomly chosen very small perturbations $\delta \bar{u}$, with $\parallel \delta \bar{u}{\parallel }_{{{{\mathcal{X}}}}}\approx 0$.

The main argument hinges on the mismatch between the sensitivity of the underlying physics, and an insensitivity hypothesis of deterministic neural networks. On the one hand, given the sensitive dependence of turbulent fluid flows to inputs, it is reasonable to hypothesize that there exists a sensitivity scale $\bar{\epsilon }\ll 1$, such that for perturbations $\parallel \delta \bar{u}{\parallel }_{{{{\mathcal{X}}}}} \sim \bar{\epsilon }$, the outputs are well-separated, i.e., $\parallel {{{\mathcal{S}}}}(\bar{u}+\delta \bar{u})-{{{\mathcal{S}}}}(\bar{u}){\parallel }_{{{{\mathcal{Y}}}}}\gg 1$. On the other hand, we argue in the SI Sec. 3 that RMSE-trained neural networks are insensitive to small-scale perturbations, i.e., ${\Psi }_{\theta }(\bar{u}+\delta \bar{u})\approx {\Psi }_{\theta }(\bar{u})$, whenever $\parallel \delta \bar{u}{\parallel }_{{{{\mathcal{X}}}}} < \bar{\epsilon }$. This phenomenon stems from several factors, such as the empirically observed fact that trained neural networks operate at the edge of chaos^30^, the well-known spectral bias of neural networks^31^, and the need for bounded gradients while training neural networks with gradient descent. Therefore, training a neural network *Ψ*_*θ*_ to learn the target conditional distribution using the ensemble perturbation approach amounts to minimizing

1 $${{\mathbb{E}}}_{\delta \bar{u}}\parallel {\Psi }_{\theta }(\bar{u}+\delta \bar{u})-{{{\mathcal{S}}}}(\bar{u}+\delta \bar{u}){\parallel }^{2}\approx {{\mathbb{E}}}_{\delta \bar{u}}\parallel {\Psi }_{\theta }(\bar{u})\\ -{{{\mathcal{S}}}}(\bar{u}+\delta \bar{u}){\parallel }^{2}\quad (\,{\mbox{insensitivity hypothesis}})$$

2 $$=	 \parallel {\Psi }_{\theta }(\bar{u}) -{{\mathbb{E}}}_{\delta \bar{u}}{{{\mathcal{S}}}}(\bar{u}+\delta \bar{u}){\parallel }^{2}+{{{{\rm{Var}}}}}_{\delta \bar{u}}[{{{\mathcal{S}}}}(\bar{u}+\delta \bar{u})].\quad \\ 	 (\,{\mbox{bias-variance decomposition}})$$

Note that we cannot replace ${{{\mathcal{S}}}}(\bar{u}+\delta \bar{u})$ with ${{{\mathcal{S}}}}(\bar{u})$ above due to the sensitive dependence of ${{{\mathcal{S}}}}$ on inputs. As the second term in the above sum is independent of *θ*, the optimal neural network is given by ${\Psi }_{\theta }(\bar{u})={{\mathbb{E}}}_{\delta \bar{u}}{{{\mathcal{S}}}}(\bar{u}+\delta \bar{u})$, which is precisely the mean of the ensemble. This simple argument explains the extensive empirical observation, both here and in the literature^19,20^, of why ensembles predicted by neural networks, trained to minimize least-square errors for learning turbulent fluids, regress to the mean flow and fail to generate sufficient variance.

However, the same insensitivity hypothesis also implies that the denoisers *D*_*θ*_ in a diffusion model such as GenCFD, being neural networks, will be as insensitive to small input perturbations, i.e., ${D}_{\theta }(\bar{u}+\delta \bar{u})\approx {D}_{\theta }(\bar{u})$, when $\parallel \delta \bar{u}{\parallel }_{{{{\mathcal{X}}}}} < \bar{\epsilon }$. Then, how are diffusion models, such as GenCFD, based on the same neural networks, able to approximate the target distribution? The answer to this lies in the nature of the loss function (8) in training diffusion models, as the following calculation shows. Starting with the specific form of the diffusion training loss (8) in our context as,

3 $${{{\mathcal{J}}}}({D}_{\theta })={{\mathbb{E}}}_{\delta \bar{u}}{{\mathbb{E}}}_{\eta }\left[\parallel {D}_{\theta }({{{\mathcal{S}}}}(\bar{u}+\delta \bar{u})+\eta ;\bar{u}+\delta \bar{u},\sigma )-{{{\mathcal{S}}}}(\bar{u}+\delta \bar{u}){\parallel }^{2}\right]$$

4 $$\approx {{\mathbb{E}}}_{\delta \bar{u}}{{\mathbb{E}}}_{\eta }\left[\parallel {D}_{\theta }({{{\mathcal{S}}}}(\bar{u}+\delta \bar{u})+\eta ;\bar{u},\sigma )-{{{\mathcal{S}}}}(\bar{u}+\delta \bar{u}){\parallel }^{2}\right]\quad (\,{\mbox{insensitivity hypothesis}})$$

5 $$={{\mathbb{E}}}_{u}{{\mathbb{E}}}_{\eta }\left[\parallel {D}_{\theta }(u+\eta ;\bar{u},\sigma )-u{\parallel }^{2}\right],$$

where the last line follows by a change of variables to $u={{{\mathcal{S}}}}(\bar{u}+\delta \bar{u})$. Thus, ${{{\mathcal{J}}}}$ is the denoiser training objective or diffusion loss for recovering the distribution corresponding to the ${{{{\rm{Law}}}}}_{\delta \bar{u}}{{{\mathcal{S}}}}(\bar{u}+\delta \bar{u})$, conditioned on the input $\bar{u}$, which is precisely the goal of our statistical computation. Moreover, the change in training objective implies that the insensitivity of trained neural networks only leads to the recovery of the mean of the denoised sample from the noisy inputs. This formal argument reveals the mechanism through which a diffusion model can leverage the unstable nature of sensitive maps, such as solution operators of fluid flows, to accurately approximate the conditional distributions, even when the underlying neural networks themselves are not sensitive to small perturbations, justifying the empirically observed performance of GenCFD. This also aligns well with recent work on spontaneous stochasticity^32–35^, which provides evidence that high-Reynolds number flows are non-deterministic at a fundamental level, further justifying a probabilistic approach. In particular, the underlying score function is a more well-behaved object than the individual trajectories in this context, making the approximation much easier through Tweedie’s formula by denoising noisy inputs with neural networks.

**Fig. 4:**
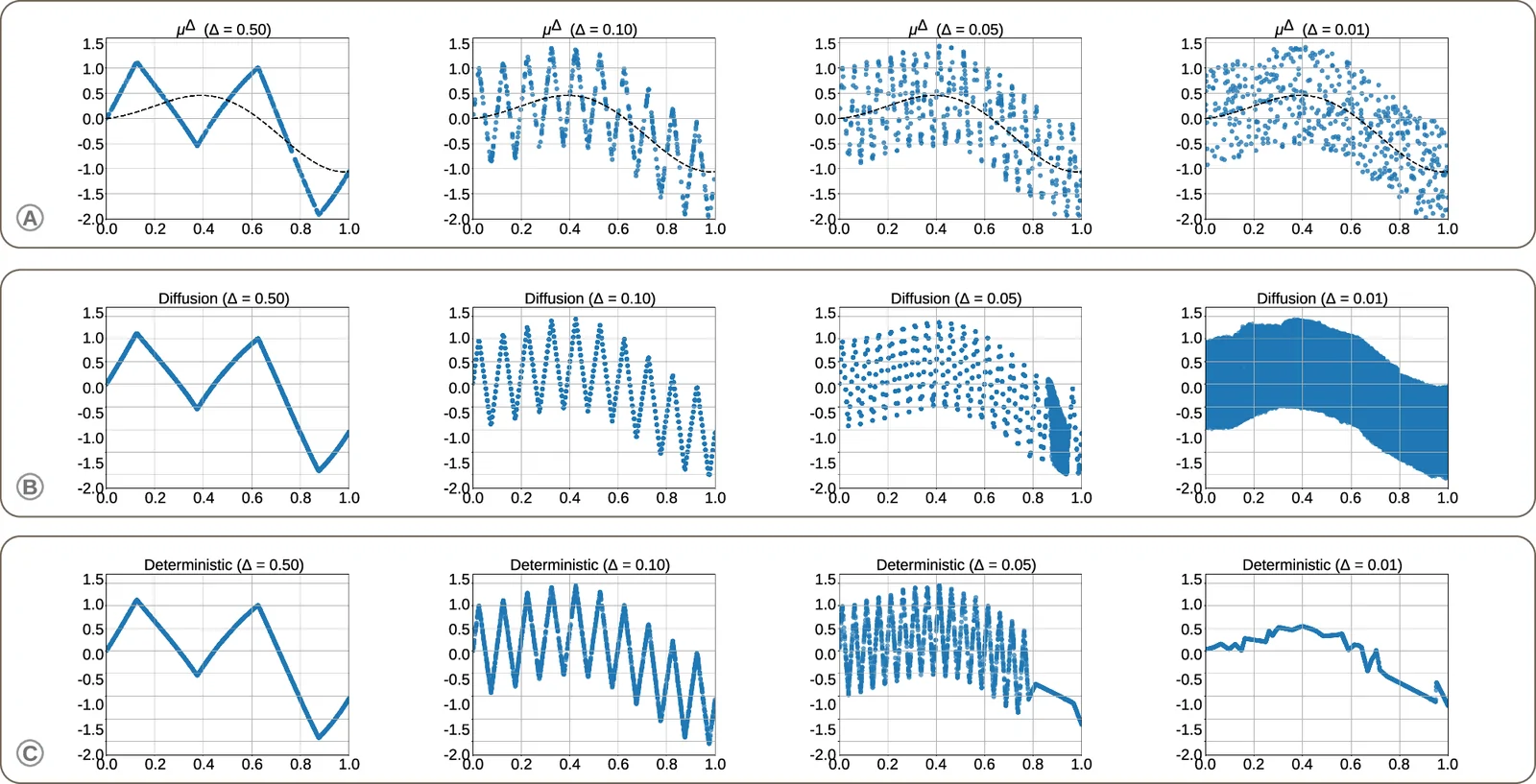
Results for Toy Model *#*1. This solvable toy model captures relevant aspects of turbulent fluids while being analytically tractable (for a detailed problem formulation, see SI Sec. 2). **A** Visualization of the underlying maps ${{{{\mathcal{S}}}}}^{\Delta }$ (as in SI Sec. 2) for different values of *Δ* representing oscillations at higher and higher frequencies (inversely proportional to *Δ*) being added to a mean function (in black), with the *Δ* → 0 limit of ${{{{\mathcal{S}}}}}^{\Delta }$ being uniform probability distributions at each point. **B** Results for different values of *Δ* with a score-based diffusion model (with an MLP as the denoiser) after 10,000 epochs of training to minimize the denoiser training objective or diffusion loss (8). **C** Results for different values of *Δ*, with an MLP after 10,000 epochs of training to minimize the mean-square loss. **B** clearly shows how the diffusion model can accurately approximate the underlying function for large *Δ*, while approximating the underlying probability distribution for *Δ* ≈ 0. On the other hand, **C** shows that the same neural network (MLP) trained to minimize the mean-square loss is able to recover the underlying map for large *Δ* but collapses to the mean as *Δ* ≈ 0. These observations are rigorously proved in SI Sec. 3. Source data are provided as a Source Data file.

We rigorously prove these statements for solvable toy models, which capture essential features of turbulent fluid flows while still being analytically tractable. To this end, in SI Sec. 2 (see also Fig. 4A for visualization), we consider a sequence of simple maps between intervals, indexed by a small parameter *Δ* that encodes the input sensitivity. These maps contain oscillations on progressively finer and finer scales as *Δ* → 0. Consequently, the asymptotic limit of these maps can only be described in terms of (pointwise) statistics which are explicitly computed in the SI Sec. 3. As their Lipschitz constant blows up when *Δ* → 0, these maps mimic the high sensitivity to small (spatial) input perturbations of ${{{\mathcal{S}}}}$. On the other hand, the spectral bias of neural networks^31^ establishes that neural networks fail to approximate high frequencies of one-dimensional oscillatory maps, rendering them insensitive to perturbations at such small scales. We corroborate these statements with simple numerical examples: Fig. 4C shows how a multilayer perceptron (MLP) trained to minimize the mean-square error between its prediction and the underlying map behaves as *Δ* → 0 and increasingly higher-frequency oscillations are introduced. We see that for relatively large *Δ*, the ML model provides an accurate approximation, at least after a lot of training steps (see SI Fig. 34). However, when *Δ* ≪ 1, as predicted by the theory, the model fails to approximate the fine-scale oscillations and regresses to the mean. On the other hand, as shown in Fig. 4B, a diffusion model (the same MLP but trained with the diffusion loss (8)) is able to approximate the underlying map for all values of *Δ*, including when *Δ* ≪ 1, where it predicts the correct limit distribution. These observations are rigorously proved in the SI Sec. 3 by deriving explicit formulae for the optimal denoisers, putting our proposed theory on a firm mathematical footing for this toy problem. Moreover, in the SI Sec. 3, we also present and rigorously analyze a second toy problem which mimics the spectral behavior of fluid flows, reproducing energy spectra similar to Fig. 2F (see SI Fig. 35).

## Discussion

We introduce GenCFD, a generative AI framework for the fast and accurate statistical computation of turbulent fluid flows. Our conditional score-based diffusion model overcomes the long-standing challenge of statistical computation, or forward UQ, by generating realistic, statistically accurate samples of complex three-dimensional flows. This capability is demonstrated across a diverse suite of five challenging benchmarks (spanning canonical turbulence, engineering applications, and atmospheric science) and evaluated using a suite of comprehensive metrics (SI Table 7). GenCFD consistently captures the correct mean, variance, energy spectrum, and higher-order statistics, whereas deterministic machine learning baselines regress to the conditional mean. This statistical accuracy is matched by a substantial gain in computational efficiency, with GenCFD delivering speedups of one to five orders of magnitude over state-of-the-art CFD solvers (SI Table 21), reducing simulation times from hours to seconds.

The success of GenCFD is underpinned by an insight into the limitations of applying deep learning to chaotic systems. We provide theoretical arguments for an insensitivity hypothesis: the architectural and algorithmic biases of neural networks towards stability and smoothness create a fundamental mismatch with the sensitive, unstable nature of turbulence. When trained with standard RMSE-based losses, these networks are forced to average over the fine-scale variations they cannot represent, leading to a collapse to the conditional mean. GenCFD circumvents this limitation through its denoising training objective borrowed from diffusion models. Because the probabilistic score is largely insensitive to small perturbations of the initial condition, this transforms the learning task from a virtually intractable pointwise matching of sensitive trajectories to a tractable problem of distribution estimation, revealing the mechanism by which diffusion models can learn the statistical structure of chaos using the same insensitive network components that cause deterministic models to fail.

GenCFD’s demonstrated capabilities and flexibility open a broad range of applications across computational science. For example, in Earth systems, GenCFD’s capability to accurately generate the dry convective planetary boundary layer provides a direct pathway to tackling one of the largest sources of uncertainty in climate projections: the emulation of high-resolution moist flows like clouds^36^. The paper’s theoretical underpinnings also shed light on the strong performance of emerging diffusion-based probabilistic weather emulators^19,20^. For physical inverse problems, our theory clarifies why diffusion models are the correct mathematical tool for sampling from the posterior in chaotic/unstable systems^37^. This principle extends naturally to other multiscaled domains, including materials science, for modeling phase transitions and the homogenization of composite materials^38^.

Although generative models have recently been used in several applications such as statistical downscaling^39,40^, physical inverse problems^41,42^, ensemble augmentation^43^, and data assimilation^44^ (see SI Table 1 for a comparison with these approaches), we believe this work complements and extends these approaches. By demonstrating (both numerically and theoretically) that generative models can learn the underlying conditional statistics of chaotic dynamical systems where deterministic approaches fail, GenCFD not only provides a practical tool for fluid dynamics but also opens a pathway for the data-driven statistical computation and forward UQ of complex physical systems across science and engineering.

## Methods

### Computing the conditional probability distribution

The mathematical challenge of forward UQ (or statistical computation) is to characterize how uncertainty in the inputs of a PDE propagates to its solution, which can be rendered tractable by leveraging the conditional distribution. Fluid flows are governed by variants of the Navier–Stokes equations (defined in SI Sec. 2), which can be written as an abstract nonlinear PDE, ${{{{\mathcal{L}}}}}_{\bar{u}}[u]=0$. Here, ${{{\mathcal{L}}}}$ is a differential operator, $\bar{u}\in {{{\mathcal{X}}}}$ represents the inputs (e.g., initial and boundary conditions), and $u\in {{{\mathcal{Y}}}}$ is the corresponding solution in suitable function spaces ${{{\mathcal{X}}}}$ and ${{{\mathcal{Y}}}}$. This defines a solution operator, ${{{\mathcal{S}}}}:{{{\mathcal{X}}}}\to {{{\mathcal{Y}}}}$, that maps inputs to solutions. Given a probability distribution of inputs, $\mu \in {{{\rm{Prob}}}}({{{\mathcal{X}}}})$, the goal of statistical computation is to determine the push-forward measure, ${{{{\mathcal{S}}}}}_{\#}\mu \in {{{\rm{Prob}}}}({{{\mathcal{Y}}}})$, which describes the resulting distribution of solutions. This is a formidable task due to the infinite-dimensional nature of the underlying function spaces^9^.

To render this problem tractable, we reformulate it by seeking to learn the conditional probability distribution of the solution, *u*, given the input, $\bar{u}$. As derived in SI Sec. 2, learning the conditional density $p(u| \bar{u})$ becomes the cornerstone for forward UQ, as it allows for the approximation of the push-forward measure for any arbitrary input distribution *μ* via Monte Carlo integration: ${{{{\mathcal{S}}}}}_{\#}\mu \approx \int\,p(u| \bar{u})d\mu (\bar{u})$.

The conventional approach to approximating $p(u| \bar{u})$ is illustrated in Fig. 1A. First, an ensemble of inputs is sampled from a distribution $\bar{p}$ (typically a mollified Dirac delta centered on a specific $\bar{u}$). Each sample is then evolved using a CFD solver that approximates the solution operator ${{{\mathcal{S}}}}$. The empirical distribution of this output ensemble serves as an approximation of the target conditional distribution, $p(u| \bar{u})$^9–11,45,46^. However, this process is prohibitive, as it requires a large number of simulations with already costly CFD solvers to achieve statistical convergence.

A seemingly straightforward solution is to replace the expensive CFD solver with a neural network surrogate, ${\Psi }_{\theta }\approx {{{\mathcal{S}}}}$, whose parameters *θ* are optimized by minimizing a mean-squared or absolute error metric. However, as previously demonstrated^19,20^ and shown again in Fig. 1 (D), this approach fails for chaotic systems, as such deterministic neural networks learn to predict the average behavior, causing the predicted ensembles to collapse to the conditional mean and severely underestimating the variance and higher-order moments of the underlying conditional distribution (cf. SI Table 17 and SI Fig. 29). We provided a theoretical explanation of this phenomenon “Theoretical results“.

### GenCFD

Instead of developing fast surrogates for ensemble-based computations, we seek to learn the underlying conditional distribution directly. As such, we propose GenCFD: a conditional diffusion model that generates samples from the probability distribution $p(u| \bar{u})$. As illustrated in Fig. 1B, a conditional diffusion model^47,48^ approximates the target conditional probability distribution with a two-step process. In the first forward step, given a pair of samples, $\bar{u} \sim \bar{p}$ and $u \sim p(u| \bar{u})$, noise is iteratively added to *u*_0_ = *u* in order to transform it to a sample *u*_*K*_ that follows a known distribution such as an isotropic Gaussian of the form ${p}_{K}({u}_{K}| \bar{u}) \sim {{{\mathcal{N}}}}({u}_{K};0,{\sigma }_{K}^{2}I)$, with zero mean and a prescribed variance ${\sigma }_{K}^{2}I$. In general, this iterative process is implemented by solving a suitable stochastic differential equation (SDE, see SI Sec. 2 for details) forward in time^49^. Next, the key step is the so-called reverse step (Fig. 1B) where given $\bar{u}$ and a noisy sample ${u}_{K} \sim {p}_{K}({u}_{K}| \bar{u})$, the reverse SDE (we note that we are using a variance exploding formulation, which simplifies this equation. See SI Sec. 2.2 for further details)

6 $$d{u}_{\tau }=-2{\dot{\sigma }}_{\tau }{\sigma }_{\tau }{\nabla }_{{u}_{\tau }}\log {p}_{\tau }({u}_{\tau }| \bar{u})d\tau+\sqrt{2{\dot{\sigma }}_{\tau }{\sigma }_{\tau }}d{\widehat{W}}_{\tau }$$

is solved backward in pseudo-time *τ* ∈ [0, *K*] with a terminal distribution *p*_*K*_ and ${\widehat{W}}_{\tau }$ is the Brownian motion in backward time. While postponing detailed notation for this SDE to SI Sec. 2, we would like to emphasize that solving it from *τ* = *K* to *τ* = 0 recovers the target conditional distribution as ${p}_{0}(u| \bar{u})=p(u| \bar{u})$^49^.

However, solving the SDE (6) requires the explicit form of the so-called score-function $\log {p}_{\tau }({u}_{\tau }| \bar{u})$ of the underlying distribution at each *τ* ∈ [0, *K*], which is not available. Instead, we follow score-based diffusion models^48,49^ and use Tweedie’s formula, ${\mathbb{E}}[u| {u}_{\tau },\bar{u}]={u}_{\tau }+{\sigma }_{\tau }^{2}{\nabla }_{{u}_{\tau }}\log {p}_{\tau }({u}_{\tau }| \bar{u})$, to approximate

7 $${\nabla }_{{u}_{\tau }}\log {p}_{\tau }({u}_{\tau }| \bar{u})\approx \frac{{D}_{\theta }({u}_{\tau }(\bar{u}),\bar{u},{\sigma }_{\tau })-{u}_{\tau }}{{\sigma }_{\tau }^{2}}.$$

Here, the so-called denoiser *D*_*θ*_, a neural network with trainable parameters *θ*, takes the condition $\bar{u} \sim \bar{p}$, the noisy sample ${u}_{\tau }(\bar{u})$ (drawn from a Gaussian ${{{\mathcal{N}}}}(\cdot ;u,{\sigma }_{\tau }^{2}I)$) and the noise level *σ*_*τ*_ as inputs in order to output the clean underlying sample. Hence, as illustrated in Fig. 1C, we need to train the denoiser *D*_*θ*_ to remove noise from the noisy sample ${u}_{\tau }(\bar{u})=u+\eta$, $\eta \sim {{{\mathcal{N}}}}(0,{\sigma }_{\tau }^{2}I)$ and output the clean underlying sample *u*. This is achieved by training the denoiser to minimize the denoiser training objective or diffusion loss,

8 $${{{\mathcal{J}}}}({D}_{\theta })={{\mathbb{E}}}_{\bar{u} \sim \bar{p}}{{\mathbb{E}}}_{u| \bar{u}}{{\mathbb{E}}}_{\eta \sim {{{\mathcal{N}}}}(0,{\sigma }_{\tau }^{2}I)}\parallel {D}_{\theta }(u+\eta ;\bar{u},{\sigma }_{\tau })-u{\parallel }^{2},$$

with true minimizer ${D}_{\theta }({u}_{\tau };\bar{u},{\sigma }_{\tau })={\mathbb{E}}[u| {u}_{\tau },\bar{u}]$. At inference, the reverse SDE (6), with its score-function replaced by the trained denoiser, is (numerically) integrated backward in time to generate samples from the target distribution $p(u| \bar{u})$, given the input condition $\bar{u}$ and isotropic Gaussian noise *u*_*K*_.

We chose a specific neural network architecture for the denoiser in our generative AI algorithm, coupled with several problem-specific innovations. As detailed in SI Sec. 2 and illustrated in Fig. 1C, it is a U-ViT^22^ type neural operator specifically adapted for multiscale information processing (see SI Fig. 6 for a schematic), to which we incorporate lead-time conditioning, all-to-all training^18^, and special variance-capturing loss functions.

### Benchmark datasets and baselines

We tested our framework on a suite of five challenging fluid flows (see SI Sec. 2 for detailed description of datasets), where we considered three state-of-the-art neural operators as baselines (defined in SI Sec. 2): the UViT model, which is also the architecture of the model underpinning GenCFD, the popular Fourier Neural Operator (FNO)^15^, and a variant of it that adds local convolutional layers to the Fourier layers, which we term as C-FNO (see SI Sec. 2). All these baselines are trained to minimize the mean-square error ${{\mathbb{E}}}_{\bar{u} \sim \bar{p}}\parallel {\Psi }_{\theta }(\bar{u})-{{{\mathcal{S}}}}(\bar{u}){\parallel }^{2}$, where *Ψ*_*θ*_ represents (generically) the baseline. The numerical convergence of the reference simulations under mesh refinement is documented in SI Fig. 2. The detailed architectures and training hyperparameters for GenCFD and all baselines are provided in SI Tables 2–6.

For the baselines, we approximate the conditional distribution using the de facto ensemble-based approach in UQ: we generate ensembles of ${({\Psi }_{\theta })}_{\#}\bar{p}$, where $\bar{p}$ is a mollified Dirac delta, following SI Sec. 2 (see SI Fig. 1 (right) for a schematic). The choice of these baselines, particularly the lack of diffusion-based approaches, is discussed in SI Sec. 2.4.

GenCFD and the baselines are trained with the same data as specified in SI Sec. 2.6. The data are composed of pairs ${\{({u}^{i},{\bar{u}}^{i})\}}_{i}$ which are built by drawing samples ${\bar{u}}^{i} \sim \mu$, where *μ* is a training distribution, and ${{u}}^{i}$ is obtaining by solving the PDE with input ${\bar{u}}^{i}$, i.e., ${u}^{i}={{{\mathcal{S}}}}({\bar{u}}^{i})$. However, at test time, we focus on benchmarking with respect to the conditional distribution $p(u| \bar{u})$, whose ground truth is approximated by using an input distribution $\bar{p}\approx {\delta }_{{\bar{u}}}$, for some ${\bar{u}} \in {{{\mathcal{X}}}}$ (see Fig. 1A for an illustration), coupled to a state-of-the-art CFD solver. We do this as it allows us to evaluate the ability of the models to generalize out of distribution. This distribution shift is substantial as shown in SI Table 22, making the out-of-distribution evaluation a stringent test for generalization. Moreover, it is well-known that, even if the initial distribution is (approximately) a Dirac measure, the intrinsic chaotic evolution of turbulent fluids spreads out the measure^8,10,45^ (see also Fig. 1A and SI Sec. 2), potentially resulting in spontaneous stochasticity that precludes a deterministic description^32–35^.

## Supplementary information

Supplementary information

Transparent Peer Review file

## Data Availability

Representative sample trajectories from the training and evaluation datasets, along with references to the complete dataset, are released as open access^50^ under a CC BY 4.0 license. The processed data in Figs. 2 and 4 (and the respective SI Figs. 2, 5, 11, 14, 18, 21, 23, 24, 34, and 35 in SI Sec. 5) are provided as a Source Data file with this paper.

## Code availability

The code for the model, including neural network architecture, training, inference, and evaluation, can be found on GitHub in https://github.com/camlab-ethz/GenCFD and is publicly released under an Apache 2.0 license on Zenodo^51^.
