# Supplementary material for: Generative AI for efficient statistical computation of fluids
Source: Nat Commun. 2026 Aug 17;17:9846. doi: 10.1038/s41467-026-76390-x (PMC13578293; doi:10.1038/s41467-026-76390-x)
Supplement: Supplementary file 1 [file 41467_2026_76390_MOESM1_ESM.pdf]

Supplementary Information to: Generative AI for efficient statistical computation of fluids

Bogdan Raonić [1,2,*], Roberto Molinaro [1,3,*], Samuel Lanthaler [4,*], Tobias Rohner [1,*], Victor Armegioiu [1], Stephan Simonis [1,5], Dana Grund [1,6], Yannick Ramic [1], Zhong Yi Wan [7], Fei Sha [8,§], Siddhartha Mishra [1,2,†], Leonardo Zepeda-Núñez [9,†]

[1] Seminar for Applied Mathematics, Department of Mathematics, ETH Zurich, Switzerland,
[2] ETH AI Center, Zurich, Switzerland,
[3] Jua.ai, Zurich, Switzerland,
[4] University of Vienna, Vienna, Austria,
[5] Institute for Applied and Numerical Mathematics, Karlsruhe Institute of Technology, Karlsruhe, Germany,
[6] Department of Environmental Systems Science, ETH Zurich, Switzerland,
[7] Google Deepmind, Mountain View, CA, USA,
[8] Meta, Menlo Park, CA, USA,
[9] Google Research, Mountain View, CA, USA,
[*] Equal contribution,
[§] Work done at Google Research, Mountain View, CA, USA,
[†] Joint supervision and corresponding authors,
e-mail: siddhartha.mishra@sam.math.ethz.ch (S. Mishra), lzepedanunez@google.com (L. Zepeda-Núñez).

Abstract. This Supplementary Information (SI) provides an overview of related work (SI 1), a detailed description of the methodology (SI 2), detailed theoretical results (SI 3), further numerical experiments (SI 4), Supplementary Figures (SI 5), and Supplementary Tables (SI 6).

Contents

List of Supplementary Figures 4

List of Supplementary Tables 6

SI 1 Related Work 7
- SI 1.1 Weather and Climate Applications . . . 7
- SI 1.2 Computational Fluid Dynamics (CFD) Applications . . . 8
- SI 1.3 Conditional Distribution . . . 8

SI 2 Detailed Methods 10
- SI 2.1 Problem Formulation . . . 10
- SI 2.2 Score-based Diffusion Models . . . 12
- SI 2.3 The Denoiser . . . 14
  - SI 2.3.1 The Denoiser Architecture . . . 14
  - SI 2.3.2 Denoiser Training . . . 16
  - SI 2.3.3 Inference of the Diffusion Model . . . 18
- SI 2.4 Baselines . . . 18
  - SI 2.4.1 FNO . . . 18
  - SI 2.4.2 C-FNO . . . 19
  - SI 2.4.3 UViT . . . 19
  - SI 2.4.4 GenCFD* $\circ$ FNO . . . 19
- SI 2.5 Training and Test Protocols . . . 20
- SI 2.6 Datasets . . . 21
  - SI 2.6.1 Taylor–Green Vortex (TGV) . . . 21
  - SI 2.6.2 Cylindrical Shear Flow (CSF) . . . 21
  - SI 2.6.3 Cloud-Shock Interaction (CSI) . . . 22
  - SI 2.6.4 Nozzle Flow (NF) . . . 23
  - SI 2.6.5 Dry Convective Planetary Boundary Layer (CBL) . . . 24
- SI 2.7 Details of Models and Hyperparameters . . . 26
- SI 2.8 Evaluation Metrics . . . 27
- SI 2.9 Details on Toy Model #1 of the Main Text . . . 28
  - SI 2.9.1 Numerical Results . . . 28

SI 3 Detailed Theory 30
- SI 3.1 Main Results . . . 30
- SI 3.2 Toy Model #1: Illustrating the Consequences of Input Sensitivity Mismatch . . . 33
- SI 3.3 Toy Model #2: Illustrating Spectral Accuracy of Diffusion Models . . . 35
  - SI 3.3.1 Motivation . . . 35
  - SI 3.3.2 Model . . . 36
  - SI 3.3.3 Theory . . . 36
- SI 3.4 Mathematical Derivation . . . 38
  - SI 3.4.1 Characterizing the Optimal Denoiser . . . 38
  - SI 3.4.2 Proofs for Section SI 3.4.1. . . . 39
  - SI 3.4.3 Proof of Proposition SI 3.3 . . . 42
  - SI 3.4.4 Proof of Proposition SI 3.12 . . . 43
  - SI 3.4.5 Proofs for Section SI 3.2 . . . 44

SI 3.4.6 Proofs for Section SI 3.3 52

SI 4 Further Experimental Results **57**

SI 4.1 GenCFD Generates High-quality Samples of the Flow 57
SI 4.2 GenCFD Accurately Approximates Statistical Quantities of Interest 58
SI 4.3 GenCFD Provides Accurate Spectral Resolution 58
SI 4.4 GenCFD Scales with Data 59
SI 4.5 Statistical Computation with GenCFD is Robust 59
SI 4.6 Statistical Computation with GenCFD is Fast 60
SI 4.7 Comparison with CRPS Training 61
SI 4.8 Role of Incompressibility 62
SI 4.9 Additional Statistical Metrics for Turbulent Fluid Flows. 63

SI 5 Supplementary Figures **65**

SI 6 Supplementary Tables **95**

List of Supplementary Figures

Figure SI 1 Schematic of the train/testing task. 65
Figure SI 2 Statistical convergence of the ground truth under mesh refinement. 65
Figure SI 3 Visualization of the vorticity of the Taylor–Green vortex at $T = 2.0$. 66
Figure SI 4 Visualization of the velocity u_x of the Taylor–Green vortex at $T = 2.0$. 66
Figure SI 5 Third-order structure function of the Taylor–Green vortex at $T = 2.0$ and convergence of its slope in the inertial range towards the ground truth. 67
Figure SI 6 Schematic of the architecture of the UViT neural network. 67
Figure SI 7 Visualization of pointwise kinetic energy for three randomly generated samples for the cylindrical shear flow experiment at time $T = 1$. 68
Figure SI 8 Visualization of pointwise vorticity intensity for three randomly generated samples for the cylindrical shear flow experiment at time $T = 1$. 69
Figure SI 9 Visualization of the mean of the pointwise kinetic energy for the cylindrical shear flow experiment at time $T = 1$, for four different initial distributions. . 70
Figure SI 10 Visualization of the standard deviation of the kinetic energy for the cylindrical shear flow experiment at time $T = 1$, for four different initial distributions. . 71
Figure SI 11 Histograms of the point PDFs, at two different points, of all velocity components for the cylindrical shear flow experiment at time $T = 1$. 72
Figure SI 12 Visualization from two different angles of the density at time $T = 0.06$ for the cloud-shock interaction experiment. 73
Figure SI 13 Visualization of the mean and the standard deviation of the density at time $T = 0.06$ for the cloud-shock interaction experiment. 74
Figure SI 14 Histograms of the point PDFs at two different points of the density, x-velocity and pressure, at time $T = 0.06$ of the cloud-shock interaction experiment. . . 75
Figure SI 15 Visualization of two random samples, for the same injection velocity, of the pointwise kinetic energy at time $T = 130$ for the nozzle flow experiment. . . . 76
Figure SI 16 Visualizations of two random samples, for the same injection velocity, of the pointwise vorticity intensity at time $T = 130$ for the nozzle flow experiment. . 77
Figure SI 17 Visualization of the mean of the pointwise kinetic energy at time $T = 130$ for the nozzle flow experiment. 78
Figure SI 18 Histograms of the point PDFs of the three velocity components at the spatial point $(0.547, 0.328, 0.271)$ at time $T = 130$ for the nozzle flow experiment. . . 78
Figure SI 19 Visualization of two random samples, of the velocity component u_x at time $T = 2.4$ for the convective boundary layer experiment. 79
Figure SI 20 Visualization of the mean of the velocity component u_x at time $T = 2.4$ for the convective boundary layer experiment. 80
Figure SI 21 Histograms of the point PDFs of the temperature and the three velocity components at the spatial point $(0.508, 0.055, 0.852)$ at time $T = 2.4$ for the convective boundary layer experiment. 80
Figure SI 22 Profiles (horizontal statistics) for the convective boundary layer experiment for GenCFD and ground truth at final simulation time $T = 7200$ s. 81
Figure SI 23 Scaling of standard deviation and Wasserstein metric error of GenCFD vs. number of training samples for the cylindrical shear flow. 82
Figure SI 24 Energy spectra, generated by the ground truth, GenCFD and the best-performing baseline for four of the 3D datasets and the spectrum of Toy Model #2. 83

Figure SI 25 Visualization of pointwise kinetic energy for three randomly generated samples for the Taylor–Green vortex experiment at time $T = 0.8$. 84

Figure SI 26 Visualization of pointwise vorticity intensity for three randomly generated samples for the Taylor–Green vortex experiment at time $T = 2$. 85

Figure SI 27 Visualization of the mean of the approximate statistical solution for the Taylor–Green vortex experiment at time $T = 2$, for four different initial distributions. 86

Figure SI 28 Visualization of the standard deviation of the pointwise kinetic energy for the Taylor–Green vortex experiment at time $T = 0.8$, for four different initial distributions. 87

Figure SI 29 Visualization of the third to fifth central moments of the approximate statistical solution for the Taylor–Green vortex experiment at time $T = 2$. 88

Figure SI 30 Visualization of pointwise kinetic energy and vorticity for second batch of three different randomly generated samples for the cylindrical shear flow experiment at time $T = 1$. 89

Figure SI 31 Visualization of pointwise kinetic energy and vorticity for third batch of three different randomly generated samples for the cylindrical shear flow experiment at time $T = 1$. 90

Figure SI 32 Visualization of pointwise kinetic energy and vorticity for fourth batch of three different randomly generated samples for the cylindrical shear flow experiment at time $T = 1$. 91

Figure SI 33 Visualization of the kinetic energy of samples drawn from the ground truth and approximated conditional distribution and corresponding computed vorticity intensity at time $T = 1$. 92

Figure SI 34 Results for Toy Model #1 at different Δ 93

Figure SI 35 Results for Toy Model #2 at two different frequencies. 94

List of Supplementary Tables

Table SI 1	Comparative summary of generative models for fluid dynamics.	95
Table SI 2	Parameters in the convective boundary layer experiment.	95
Table SI 3	Details of the GenCFD architectures used in the benchmarks.	96
Table SI 4	Experimental details for the GenCFD models.	96
Table SI 5	Details of the FNO architectures used in the benchmarks.	96
Table SI 6	Details of the C-FNO architectures used in the benchmarks.	97
Table SI 7	Overview of error metrics and other case-specific quantities evaluated in the present benchmark computations.	97
Table SI 8	Error metrics for different models for the Taylor–Green vortex experiment.	97
Table SI 9	Error metrics for CRPS in the Taylor–Green vortex experiment.	98
Table SI 10	Error metrics for the GenCFD generated velocity fields and their Leray projected versions, for the Taylor–Green vortex at $T = 2.0$.	98
Table SI 11	Error metrics for different models for the Taylor–Green vortex experiment.	99
Table SI 12	Error metrics for different models at $T = 1.0$ for the cylindrical shear flow experiment.	99
Table SI 13	Results for error metrics for different models at time $T = 1.0$ for the cloud-shock interaction experiment.	100
Table SI 14	Results for error metrics for different models at time $T = 1.0$ for the nozzle flow experiment.	101
Table SI 15	Results for error metrics for different models at time $T = 1.0$ for the dry convective boundary layer experiment.	102
Table SI 16	Error metrics for different models at $T = 0.8$ for the Taylor–Green vortex experiment.	102
Table SI 17	Error metrics of higher central moments for different models at $T = 2.0$ for the Taylor–Green vortex experiment.	103
Table SI 18	Error metrics for GenCFD for the cylindrical shear flow experiment at $T = 1$.	103
Table SI 19	Errors in Wasserstein metric for the distribution generated by GenCFD for the cylindrical shear flow experiment.	103
Table SI 20	Errors in Wasserstein metric for the distribution generated by GenCFD for the cylindrical shear flow experiment at $T = 1$.	103
Table SI 21	Runtimes and speedup for generating a single sample with the CFD solvers and with GenCFD.	104
Table SI 22	Error metrics for GenCFD and the training statistics surrogate model for the Taylor–Green vortex experiment at $T = 2$.	104

SI 1 Related Work

Generative AI has rapidly permeated the scientific community. A significant number of papers have emerged in the last two years seeking to generate samples of turbulent flows for related downstream applications, such as inflow-generation, super-resolution, etc. Beyond the theoretical insights provided herein justifying diffusion models, this manuscript distinguishes itself from recent works along two primary axes:

- the *size and complexity* of the physical systems under consideration, and
- the rigor of the *evaluation criteria*, with a focus on the entire underlying conditional probability distribution.

Regarding the first axis, we analyze five distinct, highly turbulent three-dimensional examples with different boundary conditions and physics. In contrast, the majority of existing studies focus on two-dimensional (2D) examples. When three-dimensional (3D) problems are considered, they are typically limited to one or, more rarely, two cases that exhibit relatively smooth flow behavior.

Regarding the second axis, we benchmark our model on its ability to capture the *conditional* probability distribution. This is enabled by a micro-perturbation approach, inherited from the definition of statistical solutions, which allows for the effective approximation of the conditional distribution. Accurately learning this distribution is key to generating valid samples from arbitrary initial conditions.

In what follows, we situate the contributions of this manuscript with respect to several recent papers, evaluating them against these two criteria.

SI 1.1 Weather and Climate Applications

For weather and climate applications, SEEDS [1] and GenCast [2] are some of the early papers using diffusion models. SEEDS [1] seeks to improve the computation of ensembles by conditionally sampling ensemble members conditioned on known ones. While it directly targets a conditional distribution, its application and setup are fundamentally different from ours given that the atmospheric dynamics are predominantly two-dimensional from a turbulence perspective, lacking the intricate 3D vortex structures central to our study. Furthermore, their benchmarks are based on approximately 100 ensemble members, in contrast to the thousands of members used in our work to establish ground-truth statistics.

The auto-regressive model proposed in [2] builds a forecast trajectory sequentially with snapshots spaced 12 hours apart, rather than sampling directly from the distribution at a given horizon. As it also targets atmospheric flows, the underlying dynamics are again largely quasi-2D from a turbulence standpoint, and the authors also have a limited number of samples for benchmarking against a ground truth of the conditional distribution. Along this category, ArchesWeatherGen [3] follows a similar idea to GenCast, although it uses a flow matching approach instead of a diffusion model, and it leverages a tailored transformer architecture instead of the graph-based sparse attention mechanism used in GenCast. ArchesWeatherGen also deals with the same flows, thus a quasi-2D turbulence, and does not have an ensemble to benchmark the conditional distribution. Finally, weather and climate AI models, like the ones mentioned above, have been trained on large datasets such as ERA5, which are orders of magnitude larger in size than the volume of training data considered here. What stands out for us is the strong performance of our model, given this volume of training data.

SI 1.2 Computational Fluid Dynamics (CFD) Applications

For CFD problems, several recent works have explored generative models, though they typically differ from our approach in system complexity, physical fidelity, or the nature of the learning task.

To our knowledge, the work in [4] is the first paper (with a preprint appearing in June 2023) that used a diffusion model to model 3D turbulent flows. Their work is based on a small dataset of 45 simulations of incompressible flow generated with an LES approach in OpenFOAM. This use of LES yields smoother fields than the DNS data we employ for most of our examples, as the smallest turbulent scales are modeled rather than resolved. Furthermore, their discretization is coarser resulting in smooth fields due to numerical dissipation, and the authors do not benchmark the conditional distribution. More recently, a similar approach is proposed in [5] to generate short-sequences of turbulent flows. This work, presented as a short paper, only outlines the method at a high level with limited examples and a sparse suite of benchmarking metrics.

Other works employ diffusion models as one component within a larger hybrid framework. In [6], the authors propose G-LED, a framework that learns dynamics on a latent space using a recurrent neural network, which, similarly to [7], relies on a coarse representation of the field, and then employs a diffusion model for super-resolution of the final state. G-LED is an auto-regressive model that builds trajectories snapshot-by-snapshot. While it tackles 3D problems, the examples are characterized by smooth, low-Reynolds-number flows (see Fig. 6 in [6]) and lack the intricate geometric features of the turbulent flows that we consider. Crucially, the authors use an LES ground truth, which, as noted, represents an already-approximated version of the flow, and they do not benchmark their model with respect to the conditional distribution.

A similar approach is also followed in [8], where a deterministic neural operator is used to predict a smoothed-out snapshot of the flow (approximating the conditional mean), and then a diffusion model conditioned on this prediction is used to reintroduce high-frequency details. Their work focuses primarily on 2D problems, with only one 3D example that also relies on an LES ground truth, thereby not capturing the full spectrum of turbulent behavior. The authors do not benchmark the conditional distribution of the flow.

A more recent approach [9] uses a generative model, either a diffusion or a flow matching model, on graphs to capture the pressure distribution on the surface of a wing. While the results are visually impressive and challenging from a geometric generalization perspective, this problem is distinct from ours. The pressure field is more regular than the velocity or vorticity fields we model, and the authors focus on sampling from the equilibrium state. They do not target the conditional distribution during the transient phase, which is where the transition from deterministic to turbulent flow is observed.

Finally, a different approach was considered in [10], where a neural field representation is combined with a latent diffusion model. Their experiments are predominantly in 2D. They present one 3D example of flow through a rough channel, but it is limited to an unconditional sampling task at a significantly coarser discretization ($32 \times 32 \times 64$) compared to the high-resolution grids (e.g., 128^3 for the Taylor–Green vortex) employed in our study. Consequently, their approach does not benchmark the conditional distribution. Their work also explores other applications such as super-resolution and inpainting.

SI 1.3 Conditional Distribution

Fig. SI 1 depicts the main difference between the typical evaluation for probabilistic model, and the evaluation performed in this paper. Fig. SI 1 (left) shows the typical regime for evaluation, for a given

prior distribution μ, and trajectory samples, we seek to learn the posterior given by the push-forward $\mathcal{S}_\sharp\mu$. Fig. SI 1 (right) shows the distinct testing methodology used in this manuscript. Here, using the same trajectories for training (e.g., see bold trajectory in Fig. SI 1 (left) and (right)), we seek to benchmark the conditional distribution (or statistical solution) given by $p(u_t|\bar{u})$, where the Dirac delta is approximated by mollified version of itself ($\bar{p}(\bar{u})$). Here we use an ensemble-based approximation of the conditional distribution using the mollified Dirac delta that is considered as ground truth. Then, we benchmark GenCFD so that it accurately captures $p(u_t|\bar{u})$ at different time-stamps, this is performed for several initial conditions drawn from μ (or in the case of the generalization tests, a different distribution ν).

SI 2 Detailed Methods

SI 2.1 Problem Formulation

Governing Equations. The PDEs governing fluid flows are special cases of the generic time-dependent PDE

$$\begin{aligned} \partial_t u(x,t) + \mathcal{L}\left(u, \nabla_x u, \nabla_x^2 u, \dots\right) &= 0, \quad \forall x \in D \subset \mathbb{R}^d, t \in (0,T), \\ \mathcal{B}(u) &= u_b, \quad \forall (x,t) \in \partial D \times (0,T), \\ u(x,0) &= \bar{u}(x), \quad x \in D, \end{aligned} \tag{1}$$

where, d is the spatial dimension, T is the time-horizon, $u \in C(\mathcal{X};[0,T])$ is the solution of (1), for a function space $\mathcal{X} \subset L^p(D;\mathbb{R}^n)$ for some $1 \leq p < \infty$, $\bar{u} \in \mathcal{X}$ is the initial datum, u_b is the boundary datum, and $\mathcal{L}$, $\mathcal{B}$ are the underlying differential and boundary operators, respectively.

Concrete examples of (1) are given by the well-known Navier–Stokes equations [11] for incompressible fluid flows, which take the form

$$\begin{aligned} \partial_t u + (u \cdot \nabla) u + \nabla p &= \nu \Delta u, \\ \operatorname{div} u &= 0, \end{aligned} \tag{2}$$

in a domain $D \subset \mathbb{R}^d$ with suitable boundary conditions. Here, $u : [0,T] \times D \to \mathbb{R}^d$ is the velocity field and $p : [0,T] \times D \to \mathbb{R}$ is the pressure. The parameter ν is the so-called *kinematic viscosity* of the fluid and is inversely proportional to the *Reynolds number* (Re).

Similarly, compressible fluid flow is modeled by the *compressible Navier–Stokes* equations [12]. Again, most compressible fluids of interest have Re $\gg 1$. Consequently, one is interested in the corresponding infinite Reynolds number limit which yields the *compressible Euler equations* [12]. These nonlinear PDEs are special cases of so-called *hyperbolic systems of conservation laws*: a large class of PDEs of the generic form [13]

$$\partial_t u(x,t) + \nabla \cdot F(u(x,t)) = 0. \tag{3}$$

Here $u : D \times [0,T] \to \mathbb{R}^m$ is the physical state with m components. The function $F : \mathbb{R}^m \to \mathbb{R}^{d \times m}$ is the physical flux, which describes how the physical state variables are transported through the system, and $\nabla_x \cdot F(u(x,t))$ is the (spatial) divergence of the vector field $F \circ u : D \times [0,T] \to \mathbb{R}^{d\times m}$, $(x,t) \mapsto F(u(x,t))$, with components $[\nabla_x \cdot F(u(x,t))]_j = \sum_{k=1}^d \partial_{x_k}(F_{k,j}(u(x,t)))$.

In the specific example of the compressible Euler equations, the state variables are $u = [\rho, \rho v, E]$, with density ρ, velocity $v = [v_{x_1}, v_{x_2}, \cdots, v_{x_d}]$, pressure p and total energy E related by the ideal gas equation of state

$$E = \frac{1}{2}\rho |u|^2 + \frac{p}{\gamma - 1}, \tag{4}$$

with gas constant γ. The corresponding flux function is given by,

$$F = [\rho v, \rho v \otimes v + pI, (E+p)v]. \tag{5}$$

Statistical Computation. For simplicity of the exposition, we assume that the boundary conditions in the PDE (1) are fixed. Then, the solutions of the time-dependent PDE (1) are given in terms of the underlying *solution operator* $\mathcal{S} : [0,T] \times \mathcal{X} \mapsto \mathcal{X}$ such that $u(t) = \mathcal{S}^t(\bar{u}) = \mathcal{S}(t, \bar{u})$ is the solution of (1) at any time $t \in [0,T]$.

As mentioned in the Main Text, statistical computation of (1), also termed as forward uncertainty quantification (UQ), refers to the computation of the *push-forward measure* $\hat{\mu}_t = \mathcal{S}^t_{\#}\bar{\mu}$ of some input measure $\bar{u} \sim \bar{\mu} \in \operatorname{Prob}(\mathcal{X})$ by the solution operator $\mathcal{S}^t$ of (1).

Unfortunately, this solution operator may not be necessarily well-defined [14] (particularly when $d = 3$), and even when $\mathcal{S}^t$ is well-defined, it can be very sensitive to initial conditions, i.e., $\|\mathcal{S}^t(\bar{u}) - \mathcal{S}^t(\tilde{u})\|_{\mathcal{X}}$ can grow exponentially in time, even when $\|\bar{u} - \tilde{u}\|_{\mathcal{X}} \ll 1$ [11]. Then, the question arises:

How can we even define the push-forward measure $\hat{\mu}_t = \mathcal{S}^t_{\#}\bar{\mu}$?

To answer this question, we observe that, in practice, one cannot access the solution operator $\mathcal{S}^t$ explicitly. Instead, one approximates $\mathcal{S}^t$ with numerical simulations (or analytical approximations) resulting in an operator $\mathcal{S}^{t,\Delta} \approx \mathcal{S}^t$ (in a suitable sense), for small enough values of a *discretization parameter* Δ. As $\mathcal{S}^{t,\Delta} : \mathcal{X}^\Delta \to \mathcal{X}^\Delta$ maps between finite dimensional spaces $\mathcal{X}^\Delta \cong \mathbb{R}^N$ for $N \gg 1$, the push-forward $\hat{\mu}^\Delta_t = \mathcal{S}^{t,\Delta}_{\#}\bar{\mu}$ is always well-defined. Given this, we consider the limit

$$\hat{\mu}_t = \lim_{\Delta\to 0} \hat{\mu}^\Delta_t = \lim_{\Delta\to 0} \mathcal{S}^{t,\Delta}_{\#}\bar{\mu}, \tag{6}$$

in a suitable topology. Clearly, if $\mathcal{S}^{t,\Delta} \to \mathcal{S}^t$ as $\Delta \to 0$, the above limit is simply $\hat{\mu}_t = \mathcal{S}^t_{\#}\bar{\mu}$. The interesting case materializes when the deterministic approximation $\mathcal{S}^{t,\Delta}$ does not converge to a well-defined limit as $\Delta \to 0$ as in the case of computing unstable and turbulent fluid flows [15, 14, 16, 17]. Nevertheless, the limit (6) can still be well-defined and one can even observe strong convergence to the limit, in the sense that $W_p(\hat{\mu}^\Delta_t, \hat{\mu}_t) \to 0$ as $\Delta \to 0$, for the appropriate p-Wasserstein metric on measures [16, 17]. These observations are formalized under the rubric of the theory of *statistical solutions* of PDEs [18, 19, 20] and the limit measure $\hat{\mu}_t$ is termed as the statistical solution of the PDE (1).

Given the above discussion, the goal of statistical computation for fluid flows can be reformulated as follows: we are given initial data $\bar{\mu} \in \mathrm{Prob}(\mathcal{X})$, which we approximate by a finite-dimensional projection $\bar{\mu}^\Delta$ for a given discretization parameter $\Delta > 0$. With the approximate solution operator $\mathcal{S}^{t,\Delta}$ given by a suitable finite-dimensional discretization of (1), and the map $\left(\mathcal{S}^{t,\Delta} \times \mathrm{ID}\right) : \mathcal{X} \to \mathcal{X} \times \mathcal{X}$, such that $\left(\mathcal{S}^{t,\Delta} \times \mathrm{ID}\right)(\bar{u}) = (\mathcal{S}^{t,\Delta}(\bar{u}), \bar{u})$, we consider the distribution, $\mu^\Delta_t := \left(\mathcal{S}^{t,\Delta} \times \mathrm{ID}\right)_{\#}\bar{\mu}^\Delta$ in order to explicitly condition the evolution in terms of the initial data. The corresponding limit distribution is given by

$$\mu_t = \lim_{\Delta\to 0} \mu^\Delta_t = \lim_{\Delta\to 0} \left(\mathcal{S}^{t,\Delta} \times \mathrm{ID}\right)_{\#}\bar{\mu}^\Delta. \tag{7}$$

Following [20, 16, 17], the limit can be well-defined under suitable hypotheses and $W_p(\mu^\Delta_t, \mu_t) \to 0$, even when the solution operator $\mathcal{S}^{t,\Delta}$ does not converge. In general, this limit measure admits a *conditional representation*

$$\mu_t(du, d\bar{u}) = P_t(du \,|\, \bar{u})\; \bar{\mu}(d\bar{u}), \tag{8}$$

where $P_t(du \,|\, \bar{u})$ represents the conditional probability distribution of $u(t)$ given $\bar{u}$. If $\bar{\mu} = \delta_{\bar{u}}$ and $\mathcal{S}^{t,\Delta}$ converge to the solution operator $\mathcal{S}^t$ of (1), then $P_t(du \,|\, \bar{u}) = \delta_{\mathcal{S}^t(\bar{u})}$. However, the interesting case of unstable and turbulent fluid flows corresponds to a *non-Dirac spread-out* conditional probability measure, even when the initial measure is concentrated on a function (See Main Text Fig. 1 (A) for an illustration).

Given the empirical fact that $\mu^\Delta_t \to \mu_t$ as $\Delta \to 0$, we choose Δ sufficiently small and rely on a disintegration property similar to (8) to realize the conditional probability measure $P^\Delta_t(du \,|\, \bar{u})$ with

$$\mu^\Delta_t(du, d\bar{u}) = P^\Delta_t(du \,|\, \bar{u})\; \bar{\mu}^\Delta(d\bar{u}). \tag{9}$$

This brings us to the goal for our statistical computation of fluid flows:

For discretization parameter $\Delta \ll 1$ and given initial measure $\bar{\mu}^\Delta$, compute the conditional probability measure $P^\Delta_t(du \,|\, \bar{u})$ (9) that characterizes uncertainty in a fluid flow.

For notational simplicity, we fix t and Δ and suppress the dependence on t and Δ in (9). We also observe that all the measures in (9) are supported in finite dimensions.

We will usually represent $\bar{\mu}$ and $P(du|\bar{u})$ by their (generalized) densities $\bar{p}(\bar{u})$ and $p(u \,|\, \bar{u})$, respectively. Hence, as stated in the Main Text, the goal of statistical computation boils down to approximating the conditional distribution $p(u \,|\, \bar{u})$, given the initial measure $\bar{p}(\bar{u})$ (Main Text Fig. 1 (A)).

We numerically demonstrate the convergence of the push-forward measure for the 1-Wasserstein distance in the case of the Taylor–Green vortex (a description of the experimental setup can be found in Sec. SI 2.6). The push-forward of the initial measure $\bar{\mu}$ is computed under a series of operators $\mathcal{S}^{t,\Delta}$ for the mesh resolution $\Delta = \frac{1}{N}$ and $N = 64, 128, 256, 512$. We compute the 1-Wasserstein distance of the first, and second correlation marginal between each resolution $N = 64, 128, 256$, and the reference resolution $N = 512$. Clear convergence in the mesh resolution can be observed in Fig. SI 2. Note that convergence in the Wasserstein distance of the first correlation marginal implies convergence of all statistical moments of pointwise quantities, while the convergence in the second correlation marginal implies convergence of all moments of two-point evaluations of the flow field.

SI 2.2 Score-based Diffusion Models

As mentioned in the Main Text, we will adapt a specific generative AI algorithm, namely score-based diffusion models [21, 22, 23, 24] for computing the conditional distribution $p(u \,|\, \bar{u})$, conditioned on the initial (*prior*) measure $\bar{p}(\bar{u})$. We recall from the Main Text that diffusion models, [25] and references therein, learn probability distributions based on a very simple idea, realized in terms of a process with two steps (see Main Text Fig. 1 (B) for an illustration). In a forward step, *noise* is iteratively added to samples drawn from the target distribution in order to transform it to a known noisy distribution, typically of the Gaussian type. The key *reverse* step is based on *denoising*. In it, noise is iteratively removed from samples drawn from the known noisy distribution and they are transformed into samples that follow the target distribution. Different diffusion models differ in how the denoising step is performed in practice. Here, we adapt the widely used *score-based diffusion models*, [26] and references therein.

Learning Unconditional Distributions with Score-based Diffusion Models. For the ease of exposition, we will first consider the case of a target distribution $p \in \mathrm{Prob}(\mathbb{R}^N)$ that we wish to learn from data, the *forward step* in a score-based diffusion model consists of adding noise to samples drawn from p by solving the *stochastic differential equation* (SDE) [26]

$$du_\tau = \frac{\dot{s}_\tau}{s_\tau} u_\tau \, d\tau + s_\tau \sqrt{2\dot{\sigma}_\tau \sigma_\tau} \, dW_\tau, \quad \text{for } \tau \in [0, K], \tag{10}$$

with time index τ, which stands for the time variable in the *diffusion process* and is not related to the time t used to express the physical time evolution in the PDE (1). The drift and diffusion coefficients are given in terms of the *shape function* s_τ and noise function σ_τ, respectively, and W_τ is the N-dimensional standard Wiener process. The shape and noise functions s_τ, σ_τ are chosen such that setting $s_0 = 1, \sigma_0 = 0$ results in aligning the marginal distribution $p_0 = p$ with the target distribution p.

Solving the SDE (10) forward in time τ results in the addition of noise to the samples $u_0 \sim p_0 = p$, transforming them to samples drawn from a so-called *Gaussian Perturbation Kernel*

$$p_K(u_K) \sim \mathcal{N}(u_K; 0, s_K^2 \sigma_K^2 I), \tag{11}$$

leading to a terminal distribution which is indistinguishable from an *isotropic Gaussian with zero mean* at time $\tau = K$.

The *reverse step* in a score-based diffusion model consists of solving the so-called *reverse SDE*

$$du_\tau = \left[\frac{\dot{s}_\tau}{s_\tau} u_\tau - 2\dot{\sigma}_\tau \sigma_\tau s_\tau^2 \nabla_u \log p_\tau(u_\tau)\right] d\tau + s_\tau \sqrt{2\dot{\sigma}_\tau \sigma_\tau} d\widehat{W}_\tau \tag{12}$$

backward in time with terminal distribution p_K (as defined in (11)), while p_τ is the underlying distribution at any time $\tau \in [0, K]$. This reverse process yields the desired target distribution $p_0 = p$ as the initial distribution at $\tau = 0$.

While the forward SDE (10) is straightforward to simulate, once the so-called *diffusion schedule* (s_τ, σ_τ) is given, solving the reverse-SDE (12) needs the (approximate) knowledge of the so-called *score function* $\nabla_u \log p_\tau(u)$. The approximation of this score function lies at the heart of any diffusion model.

For our work, we will adopt the widely-used framework of [26] and approximate the score function in (12) via a *denoiser* $D_\theta(u + \epsilon_\tau, \sigma_\tau)$, which is a parametric function with parameters $\theta \in \Theta \subset \mathbb{R}^M$. Given a sample $u \sim p$, drawn from the target distribution p and the given noise level $\epsilon_\tau = \epsilon\sigma_\tau$, for the noise function σ_τ and a parameter ϵ, the parameters θ of the denoiser are learned by minimizing the error in predicting the underlying *clean* sample u. Tweedie's formula [25] then relates the score-function in (12) as

$$\nabla_u \log p_\tau(u_\tau) \approx \frac{D_\theta(\hat{u}_\tau, \sigma_\tau) - \hat{u}_\tau}{s_\tau \sigma_\tau^2}, \text{ with } \hat{u}_\tau := \frac{u_\tau}{s_\tau}, \tag{13}$$

enabling the solution of the reverse SDE (12). Thus, one needs to specify the diffusion schedule and the denoiser architecture in order to characterize a diffusion model.

Learning Conditional Distributions. Given that our goal of statistical computation of fluid flows entails computing conditional distributions $p(u \,|\, \bar{u})$, we need to adapt the score-based diffusion model presented above. To this end, we follow the approach of [27] and modify the denoiser in (13) to take the form

$$D_\theta(u_\tau, \sigma_\tau) \to D_\theta(u_\tau(\bar{u}), \bar{u}, \sigma_\tau), \tag{14}$$

with noise σ_τ now added to samples $u(\bar{u})$ drawn from the underlying conditional distribution $p(u \,|\, \bar{u})$. Moreover, samples drawn from the prior distribution $\bar{u} \sim \bar{p}$ are explicit inputs to the denoiser in (14). Theorem 1 of [27] shows that Tweedie's formula (13) can be readily modified in this case to yield

$$\nabla_u \log p_\tau(u_\tau | \bar{u}) \approx \frac{D_\theta(u_\tau(\bar{u}), \bar{u}, \sigma_\tau) - \hat{u}_\tau}{s_\tau \sigma_\tau^2}. \tag{15}$$

Samples from the target conditional distribution $p(u \,|\, \bar{u})$ are now drawn by simulating the reverse SDE with the score function $\nabla_u \log p_\tau(u_\tau \,|\, \bar{u})$ being approximated by the denoiser (15).

Learning Time-Conditioned Distributions. Given the time-dependent nature of our underlying PDE (1), we need to learn probability distributions $p(u \,|\, (t, \bar{u}))$, with $t \in [0, T]$ being the time and $\bar{u} \sim \bar{p}$, the (finite-dimensional approximation of) initial data. Hence, the denoiser (14) has to be further modified to condition it on the time variable t so that the entire trajectory of the distribution can be generated. Moreover, given the results of [28] where an *all-to-all* training procedure was proposed for learning solution operators of time-dependent PDEs, we will similarly exploit the *semi-group* property of the solution operator of (1) to *condition the denoiser on lead times.* To this end, we further modify the denoiser (14) to

$$D_\theta(u_\tau(\bar{u}), \bar{u}, \sigma_\tau) \to D_\theta(t_n - t_\ell, u_\tau(t_n, \bar{u}), u(t_\ell, \bar{u}), \sigma_\tau), \tag{16}$$

with times $0 \le t_\ell \le t_n \le T$, initial data $\bar{u} \sim \bar{p}$ and $u(t_\ell, \bar{u})$ being the state of the system at a previous time step t_ℓ and noise σ_τ added to the current state $u(t_n, \bar{u})$ of the system at time t_n. Consequently,

these intermediate conditional distributions can be chained together to learn the target conditional distribution by

$$p(u \mid \bar{u}) = \prod_{\ell=1}^{L} p(u(t_\ell, \bar{u}) \mid u(t_{\ell-1}, \bar{u})), \tag{17}$$

with $0 \leq t_0 < t_1 < \ldots < t_\ell < \ldots < t_L = T$ being a set of monotonically increasing lead times and $p(u \mid \bar{u})$ being the conditional distribution at the final time T, given the initial condition $\bar{u} \sim \bar{p}$.

SI 2.3 The Denoiser

The main remaining step in specifying our conditional score-based diffusion model is to choose the architecture and training process for the denoiser in (16).

SI 2.3.1 The Denoiser Architecture

We choose a UViT [29] as the model for our denoiser (16), see Fig. SI 6 for a schematic. As seen in this figure, the model takes the lead time, the noisy sample at the current time, the underlying sample at a previous time, and the noise level to output a *denoised* or clean sample. The inputs are lifted into a latent space and processed through a set of *convolutional hidden layers*, which are stacked together in an *encoder-decoder* form as suggested in the widely used U-Net architecture of [30] in order to enable *multi-scale* information processing. In contrast to the standard U-Net, UViT replaces a convolutional layer at the bottleneck (base of the U-Net) with a *global attention layer* such that global mixing can take place in latent space. In three space dimensions, *axial attention* blocks [31] replace the global attention layer for computational efficiency. Residual skip connections are added to transfer information between the encoder and decoder at all hidden layers. Finally, the noise level and lead time are conditioned into the model at all levels by incorporating them inside the conditional layer norms of the model. All these steps are further detailed below.

As illustrated in Fig. SI 6, the operations of the denoiser start with the input data $\bar{u} \in \mathbb{R}^{(v \times h \times w)}$ being projected into an embedding space of dimension C_0 through a convolutional layer. The data is then sequentially downsampled n times, reducing its resolution in each dimension by a factor of 2^n. Each downsampling step is followed by a residual block composed of n_{res} layers, where each layer is structured as a sequence of a group normalization layer (denoted as GN in the figure), a non-linear activation function f, and a convolutional layer $\mathcal{C}$. This sequence is repeated twice. Meanwhile, the noise level σ_τ and lead time t_n are embedded using two independent Fourier projections, concatenated, and processed by a multi-layer perceptron (MLP) comprised of two linear layers L and a non-linear activation function f. The MLP generates scale a and shift b parameters, which are used to condition the second group normalization in the residual blocks. Following the residual blocks, multi-head attention mechanisms are applied in each layer. To address the computational cost of global attention in 3D data, axial attention is implemented instead [31]. On the other hand, data upsampling is performed through a *depth-to-space* operation. In other words, a linear transformation is first performed to increase the number of channels by a factor of 4, transforming the input tensor of shape (h, w, c) to $(h, w, 4c)$ and subsequently reshaped as $(c, 2h, 2w)$. Similarly to the downsampling stack, in the upsampling stack, each upsampling layer is followed by residual and attention blocks. Skip connections are used between downsampling and upsampling stacks.

Below, we rigorously formulate the building pieces of the main blocks of the denoiser architecture.

Affine Transformation. A linear layer in neural networks performs an affine transformation on the input data $x \in \mathbb{R}^{h\times w\times c}$, defined as:

$$L : \mathbb{R}^c \to \mathbb{R}^{\hat{c}}, \quad L(x) = xW + b, \tag{18}$$

where $W \in \mathbb{R}^{c\times\hat{c}}$ is the weight matrix containing the learnable parameters, and $b \in \mathbb{R}^{\hat{c}}$ is the bias vector, which also consists of learnable parameters.

Convolution. The discrete, multi-channel convolution with an s-stride of the input x is defined as follows:

$$\mathcal{C} : \mathbb{R}^{h\times w\times c} \to \mathbb{R}^{\hat{h}\times\hat{w}\times\hat{c}}, \quad \mathcal{C}(x) = (x \star K_w)[i, j, \hat{l}] \coloneqq \sum_{m,n=0}^{k-1} \sum_{l=1}^{c} K_w[m, n, l, \hat{l}] \cdot x[is + m, js + n, l], \tag{19}$$

where l, $\hat{l}$ correspond to the indices of the input and output channels, respectively, and $i = 0, \ldots, h-1$, $j = 0, \ldots, w-1$, $\hat{l} = 1, \ldots, \hat{c}$. Downsampling in the architecture in Fig. SI 6 is performed with a convolution operation with kernel size 3 and stride 2.

Group Normalization. Group normalization (GN) is a technique used to normalize the features of an input tensor $x \in \mathbb{R}^{h\times w\times c}$. Unlike batch normalization, which normalizes across the batch dimension, group normalization divides the channels into groups and normalizes the features within each group. Specifically, the channels are split into G groups, each containing $\frac{c}{G}$ channels. For each group g, the mean μ_g and variance σ_g^2 are computed as

$$\mu_g = \frac{1}{m}\sum_{i\in g} x_i, \quad \sigma_g^2 = \frac{1}{m}\sum_{i\in g}(x_i - \mu_g)^2, \tag{20}$$

respectively, where $m = \frac{hwc}{G}$ is the number of elements in each group. The normalized output is then given by

$$\hat{x}_i = \frac{x_i - \mu_g}{\sqrt{\sigma_g^2 + \epsilon}}, \tag{21}$$

where ϵ is a small constant for numerical stability. Finally, a learnable scale γ and shift β are applied to each normalized group, yielding the final output

$$\tilde{x}_i = \gamma\hat{x}_i + \beta. \tag{22}$$

Fourier Embedding and Adaptive Scale. Given an input tensor $x \in \mathbb{R}^{h\times w\times 1}$, where the last dimension typically represents the temporal lead time t or the diffusion noise σ, Fourier embeddings transform these coordinates into a higher-dimensional space as

$$\gamma(x) = [\sin(2\pi Bx), \cos(2\pi Bx)], \tag{23}$$

where $B \in \mathbb{R}^{d_f\times 2}$ is a matrix of frequencies, chosen from a fixed grid, and d_f is the dimensionality of the Fourier feature space. The resulting embedding $\gamma(x)$ has a shape of $h \times w \times 2d_f$, capturing both the sine and cosine components at multiple frequencies. Two independent Fourier embeddings are used for the lead time and diffusion noise. The embeddings $\gamma_t(x)$ and $\gamma_\sigma(x)$ are then concatenated together to

$$\gamma(x) = \mathrm{Concat}(\gamma_t(x), \gamma_\sigma(x)) \tag{24}$$

and transformed through an MLP $\mathcal{M}$, defined as

$$\mathcal{M}(\gamma(x)) = \mathrm{GeLU}(\gamma(x)W_1 + b_1)W_2 + b_2, \tag{25}$$

with $W_1 \in \mathbb{R}^{2d_f \times C_0}$, $b_1 \in \mathbb{R}^{C_0}$, $W_2 \in \mathbb{R}^{C_0 \times 2C_0}$, $b_2 \in \mathbb{R}^{2C_0}$. The output of the MLP is then split into a scale $a \in \mathbb{R}^{C_0}$ and shift $b \in \mathbb{R}^{C_0}$ used to adjust suitable group normalization in the residual blocks of the UVit as

$$\tilde{x} = (a+1)GN(x) + b. \tag{26}$$

Multi-Head Attention. Given an input tensor $x \in \mathbb{R}^{h\times w\times c}$ ($x \in \mathbb{R}^{h\times w\times d\times c}$ for 3D problems), following common practice in vision, the tensor is first reshaped into a new one of shape (hw, c) ((hwd, c) for 3D problems). To preserve spatial information, a positional embedding $p \in \mathbb{R}^{hw\times c}$, learned during training, is added to the reshaped tensor, yielding $x' = x + p$. The multi-head attention is then defined as

$$\mathrm{MHA}(x') = \mathrm{Concat}(\mathcal{A}_1(x'), \ldots, \mathcal{A}_h(x'))W^O, \tag{27}$$

where $W^O \in \mathbb{R}^{c\times c}$ is the output projection matrix, and $\mathcal{A}_l(x')$, for $l = 1, \ldots, h$, represents the outputs of each attention head. Each head $\mathcal{A}_k$ computes scaled dot-product attention as follows:

$$\mathcal{A}_k(x') = \mathrm{Attention}(Q_k, K_k, V_k) = \mathrm{softmax}\left(\frac{Q_k K_k^\top}{\sqrt{d_k}}\right) V_k, \tag{28}$$

where $Q_k = x'W_k^Q$, $K_k = x'W_k^K$, and $V_k = x'W_k^V$ are the query, key, and value projections for the k-th head. d_k is the dimension of each head's subspace (typically $d_k = \frac{c}{h}$ for h heads), and $W_k^{Q,K,V} \in \mathbb{R}^{d_k\times d_k}$ are the respective projection matrices. The attention block in the UVit architecture is defined as

$$\tilde{x} = x + \mathrm{MHA}(\mathrm{GN}(x)), \tag{29}$$

where GN is a group norm with 32 groups.

Axial Attention. The memory requirements of standard attention scale quadratically with the sequence length. In the case of multidimensional problems, it becomes prohibitively expensive also if performed at the bottleneck of a U-structure like the ones used in UVit. To circumvent this problem, axial attention has been proposed [31]. Axial attention performs attention sequentially over each axis i of the tensor x, mixing information only along the axis i. It is implemented by simply transposing all axes except i to the batch axis, and performing MHA as described above. In the case of 3D problems, given the input tensor $x \in \mathbb{R}^{h\times w\times d\times c}$, the axial attention block along the axis i can be defined as

$$\tilde{x} = x + \mathrm{GN}_2(\mathrm{MHAA}_i(\mathrm{GN}_1(x))), \tag{30}$$

where MHAA_i denotes multi-head axial attention along the $i - th$ axis, i.e.

$$\mathrm{MHAA}_i(x) = \mathrm{MHA}(\mathrm{Transpose}_i(x)). \tag{31}$$

Here, $\mathrm{Transpose}_i$ is the operation transposing all axes except i to the batch axis. The block is repeated for each axis.

SI 2.3.2 Denoiser Training

The parameters $\theta \in \Theta \subset \mathbb{R}^M$ that define the denoiser (16) need to be determined from training data. To this end, we consider training data in the form of *trajectories* $S_i = \{u(t_\ell, \bar{u}^i)\}_{\ell=1}^L$, for $1 \le i \le I$, with all $\bar{u}^i \sim \bar{p}$ and $0 = t_0 < t_1 < \ldots < t_\ell < \ldots < t_L = T$. Then, the denoiser parameters are given as the (local) minimizers for $\theta \in \Theta$ for the *denoising loss function*:

$$L(D_\theta, \sigma) = \mathbb{E}_{\bar{u}^i}\mathbb{E}_{(u(t_\ell,\bar{u}^i),u(t_n,\bar{u}^i))}\mathbb{E}_\eta \left\| D_\theta(t_n - t_\ell, u(t_n, \bar{u}^i) + \eta, u(t_\ell, \bar{u}^i), \sigma) - u(t_n, \bar{u}^i)\right\|^2. \tag{32}$$

Here, $\bar{u}^i \sim \bar{p}$, $(u(t_\ell, \bar{u}^i), u(t_n, \bar{u}^i)) \sim S_i$, $\eta \sim \mathcal{N}(0, \sigma^2 I)$, $t_n > t_\ell$, and $0 < t_n \leq T$. Thus, for any noise level σ, the denoiser is trained in order to remove the noise from a noisy sample and output the *clean* sample (see Main Text Fig. 1 (C) for an illustration).

Our training of the denoiser-based diffusion model largely follows the methodology proposed in [26]. For ease of notation, in the rest we will set $\sigma_\tau = \sigma$, $u_\tau = u$, and focus without loss of generality and for the ease of presentation on the unconditional case. In this case, the loss function for training the denoiser D_θ simplifies to

$$\mathcal{L}(D_\theta, \sigma) = \mathbb{E}_{u \sim p_{\text{data}}} \mathbb{E}_{\eta \sim \mathcal{N}(0, \sigma^2 I)} \| D_\theta(u + \eta, \sigma) - u \|^2 \tag{33}$$

and

$$\mathcal{L}(D_\theta) = \mathbb{E}_{\sigma \sim p_{\text{train}}} \left[\lambda(\sigma) L(D_\theta, \sigma) \right], \tag{34}$$

where λ is a weight dependent on the noise level σ and $p_{train} = p_{\text{train}}(\ln \sigma) = \mathcal{U}(\ln(\sigma_{\min}), \ln(\sigma_{\max}))$, $\sigma_{\min} = 10^{-3}, \sigma_{\max} = 80$.

Moreover, in score-based diffusion models, it is common [26] to precondition the denoiser predictions as

$$D_\theta(u + \eta, \sigma) = c_{\text{skip}}(\theta)(u + \eta) + c_{\text{out}}(\theta) F_\theta \left(c_{in}(\sigma)(u + \eta); c_{\text{noise}}(\sigma) \right), \tag{35}$$

where F_θ is the raw U-Net model. Upon defining

$$F_{\text{target}} = \frac{1}{c_{\text{out}}(\sigma)} (u - c_{\text{skip}}(\sigma)(u + \eta)), \tag{36}$$

the loss function can be rewritten as

$$\mathcal{L}(D_\theta) = \mathbb{E}_{\sigma, u, n} \left[w(\sigma) \| F_\theta \left(c_{\text{in}}(\sigma)(u + \eta), c_{\text{noise}}(\sigma) \right) - F_{\text{target}} \|_2^2 \right]. \tag{37}$$

Here, c_{noise} is chosen to be $c_{\text{noise}}(\sigma) = \frac{1}{4} \log(\sigma)$. On the other hand, c_{in}, c_{out}, c_{skip} are derived by imposing the following requirements:

$$\text{Var}[c_{\text{in}}(\sigma)(u + \eta)] = 1 \quad \Rightarrow \quad c_{\text{in}}(\sigma) = \frac{1}{\sqrt{\sigma^2 + \sigma_{\text{data}}^2}}, \tag{38}$$

$$\text{Var}[F_{\text{target}}] = 1 \quad \Rightarrow \quad c_{\text{out}}(\sigma) = \sigma \cdot \sqrt{\sigma^2 + \sigma_{\text{data}}^2}, \tag{39}$$

$$c_{\text{skip}}(\sigma) = \text{argmin}_{c_{\text{skip}}^2} c_{\text{skip}}(\sigma) \quad \Rightarrow \quad c_{\text{skip}}(\sigma) = \frac{\sigma_{\text{data}}}{\sigma^2 + \sigma_{\text{data}}^2}. \tag{40}$$

Finally, the weight λ is obtained by requiring the effective weight $w(\sigma)$ to be uniform across noise levels, i.e.

$$w(\sigma) = 1 \quad \Rightarrow \quad \lambda(\sigma) = \frac{\sigma^2 + \sigma_{\text{data}}^2}{(\sigma \sigma_{\text{data}})^2}. \tag{41}$$

Details can be found in Appendix B.6 of [26]. In all the experiments addressed in this paper, $\sigma_{data} = 0.5$.

Variance Capturing Loss. In order to improve the approximation of the standard deviation of the generated samples, the following term can be added to the loss function:

$$\mathcal{L}_{sq}(D_\theta) = \mathbb{E}_{\sigma, u, \eta} \left[w(\sigma) \| D_\theta(u + \eta, \sigma)^2 - u^2 \|_2^2 \right]. \tag{42}$$

This is motivated by the fact that the variance of a random variable x is defined as

$$\mathbb{V}[x] = \mathbb{E}[x^2] - \mathbb{E}[x]^2. \tag{43}$$

If we assume that the denoiser prediction $\tilde{u} = D_\theta(u+\eta, \sigma)$ is a Gaussian perturbation of the noise-free data u, i.e. $\tilde{u} = u + \psi$ (for instance, for large σ, the denoiser predictions are still affected by noise), then

$$\mathbb{V}[\tilde{u}] = \mathbb{V}[u+\psi] = \mathbb{E}[u^2] + \mathbb{E}[\psi^2] + 2\mathbb{E}[u\psi] - \mathbb{E}[u]^2 \tag{44}$$

and

$$\mathbb{V}[\tilde{u}] - \mathbb{V}[u] = \mathbb{E}[\psi^2] + 2\mathbb{E}[u\psi]. \tag{45}$$

Therefore, the error in the variance learned samples can be reduced by minimizing (42), weighted by a factor λ_{sq}, together with (34), i.e.

$$L(D_\theta) = \mathcal{L}(D_\theta) + \lambda_{sq}\mathcal{L}_{sq}(D_\theta). \tag{46}$$

SI 2.3.3 Inference of the Diffusion Model

At the time of inference, samples can be generated by solving the following SDE:

$$du = 2\left(\frac{\dot{\sigma}}{\sigma} + \frac{\dot{s}}{s}\right) d\tau - 2s\frac{\dot{\sigma}}{\sigma} D_\theta\left(\frac{u}{s}, \sigma\right) d\tau + s\sqrt{2\dot{\sigma}\sigma}dw. \tag{47}$$

Details can be found in [26], Appendix B. More specifically, we use the Euler–Maruyama method, where the time steps are defined according to a sequence of noise levels $\{\sigma_i\}$, $\tau_i = \sigma^{-1}(\sigma_i)$, $i = 1, ..., N$, $N = 128$ and

$$\sigma_i = \left(\sigma_{\max}^{\frac{1}{\rho}} + \frac{i}{N-1}(\sigma_{\min}^{\frac{1}{\rho}} - \sigma_{\max}^{\frac{1}{\rho}})\right)^{\rho}. \tag{48}$$

At this point, it only remains to define the σ and s scheduling.

Sigma Scheduler. Two most common choices of the noise scheduler σ are

- exponential: $\sigma_\tau = \mathcal{O}(\exp(\tau))$,
- tangent: $\sigma_\tau = \mathcal{O}(\tanh(\tau))$.

In all experiments, the exponential noise scheduler is used, as it was empirically determined to be a more effective choice for our models.

Diffusion Scheme. We employ the variance-exploding (VE) schedule, which sets the forward scheduler $s_\tau = 1$, $\forall t$.

SI 2.4 Baselines

In addition to our conditional score-based diffusion model, GenCFD, we consider the following ML baselines for all our experiments. Note that all these baselines are trained to minimize the mismatch between their predictions and the ground truth in the mean square or L^2-norm.

SI 2.4.1 FNO

A *Fourier neural operator* (FNO) $\mathcal{G}$ [32] is a composition

$$\mathcal{G} : \mathcal{X} \to \mathcal{Y}, \quad \mathcal{G} = Q \circ \mathcal{L}_L \circ \cdots \circ \mathcal{L}_1 \circ R. \tag{49}$$

It has a *lifting operator* $u(x) \mapsto R(u(x), x)$, where R is represented by a linear function $R : \mathbb{R}^{d_u} \to \mathbb{R}^{d_v}$ where d_u is the number of components of the input function and d_v is the *lifting dimension*. The operator Q is a non-linear projection, instantiated by a shallow neural network with a single hidden layer, 128 neurons and GeLU activation function, such that $v^{L+1}(x) \mapsto \mathcal{G}(u)(x) = Q\left(v^{L+1}(x)\right)$.

Each *hidden layer* $\mathcal{L}_\ell : v^\ell(x) \mapsto v^{\ell+1}(x)$ is of the form

$$v^{\ell+1}(x) = \sigma\left(W_\ell \cdot v^\ell(x) + \left(K_\ell v^\ell\right)(x)\right), \tag{50}$$

with $W_\ell \in \mathbb{R}^{d_v \times d_v}$ a trainable weight matrix (residual connection), σ an activation function, corresponding to GeLU, and the *non-local Fourier layer*

$$K_\ell v^\ell = \mathcal{F}_N^{-1}\left(P_\ell(k) \cdot \mathcal{F}_N v^\ell(k)\right), \tag{51}$$

where $\mathcal{F}_N v^\ell(k)$ denotes the (truncated)-Fourier coefficients of the discrete Fourier transform (DFT) of $v^\ell(x)$, computed based on the given s grid values in each direction. The maximal number of modes is set to M. Note that $P_\ell(k) \in \mathbb{C}^{d_v \times d_v}$ is a complex Fourier multiplication matrix indexed by $k \in \mathbb{Z}^d$, and $\mathcal{F}_N^{-1}$ denotes the inverse DFT.

For time-dependent problems, the lead time is conditioned into the FNO model at all levels by incorporating it inside the conditional instance norms of the model. Those normalization layers are applied before *each* Fourier layer.

SI 2.4.2 C-FNO

We enhanced the performance of the FNO model by incorporating *local convolution operations* in addition to the global convolutions performed in the original Fourier layer. Let $\mathcal{C}_{m,d_v}$ be a discrete, local convolution operator applied with a kernel of size $d_v \times d_v \times m^d$, acting on the space of functions of v^ℓ. The *modified* Fourier layer is then defined by

$$\tilde{v}^\ell(x) = \mathcal{C}_{3,d_v} \circ \sigma \circ \mathcal{C}_{5,d_v} \circ v^\ell(x), \tag{52}$$

$$v^{\ell+1}(x) = \sigma\left(W_\ell \cdot \tilde{v}^\ell(x) + \left(K_\ell \tilde{v}^\ell\right)(x)\right). \tag{53}$$

Thus, we alternate between global Fourier layers and local convolutions in this architecture.

SI 2.4.3 UViT

For the UViT baseline, we use the same architecture as the UViT in the diffusion model. However, the learning objective differs since the baseline is deterministic. As a result, training this baseline is significantly different from training the diffusion model. Thus, the use of the UViT model can be considered as an *ablation* for the role of the diffusion training objective (32) in the performance of GenCFD.

SI 2.4.4 GenCFD* ∘ FNO

This baseline, used solely for the Taylor–Green vortex dataset, serves as an ablation study and consists of a GenCFD* model conditioned only on the coarse FNO prediction. Unlike the original GenCFD, it does not incorporate conditioning on $\bar{u}$ as input. While not entirely unconditioned, GenCFD* relies solely on the coarse-scale solution to reconstruct fine-scale details. This setup follows the approach presented in [8].

SI 2.5 Training and Test Protocols

GenCFD and the above-mentioned baselines are trained on all the datasets by sampling data $\bar{u} \sim \mu_0$, where the underlying probability distributions for each dataset are described below.

However, as mentioned in the Main Text, at *test time*, our goal is the statistical computation of fluid flow (1) with *Dirac* input distributions, i.e., the inputs (for simplicity, initial conditions) are given by

$$\hat{\mu}_0 = \delta_{\hat{u}}, \tag{54}$$

which implies that $\bar{\mu}^\Delta = \delta_{\hat{u}^\Delta}$ in (8), with $\hat{u}^\Delta \approx \hat{u}$. Thus, we test the ability of our algorithm and the baselines to compute statistical solutions in the interesting case where the input is a Dirac and it is only the chaotic evolution of the flow that makes the conditional measure (8) *spread out*. To generate the ground truth with such Dirac initial conditions, we use an *ensemble perturbation* approach outlined below.

Ensemble Perturbation Approach for Ground Truth Generation. Given a distribution of inputs (say initial conditions) $\bar{u} \sim \bar{\mu}$, we approximate the ground-truth distribution at time $T = t$ by the empirical measure $\hat{\mu}_t^\Delta$ induced by Monte Carlo samples on $\bar{\mu}^\Delta$ which are propagated in time by a classical numerical method (See Main Text Fig. 1 (A) for an illustration). This strategy is used to approximate the conditional probability measure $P_t^\Delta(du|\bar{u})$ as follows:

$$P_t^\Delta(du|\bar{u}) = \hat{\mu}_{\bar{u},t}^\Delta \quad \text{with} \quad \bar{\mu}_{\bar{u}}^\Delta \approx \bar{\mu}_{\bar{u}} = \delta_{\bar{u}}. \tag{55}$$

Due to the deterministic nature of the classical simulation, the initial distribution $\bar{\mu}_{\bar{u}} = \delta_{\bar{u}}$ also needs to be approximated by another distribution $\bar{\mu}_{\bar{u}}^\Delta \approx \bar{\mu}_{\bar{u}}$ with $B_{\frac{\varepsilon}{2}}(\bar{u}) \subseteq \operatorname{supp} \bar{\mu}_{\bar{u}}^\Delta \subseteq B_\varepsilon(\bar{u})$ for some $\varepsilon > 0$. This slight modification causes trajectories to diverge and approximate the underlying distribution. Note that this approximation can be made as accurate as desired by choosing a small enough value for ε. In general, we take $\varepsilon \sim \Delta$ for the experiments performed in this paper.

Note that we now have two nested probability distributions. Firstly, the distribution of initial conditions $\bar{\mu}$, and secondly the approximations $\bar{\mu}_{\bar{u}}^\Delta$ of $\delta_{\bar{u}}$ for each concrete initial condition $\bar{u}$. The sampling is therefore also realized by two stages of Monte Carlo sampling. Note that $|\operatorname{supp} \bar{\mu}| \gg |\operatorname{supp} \bar{\mu}_{\bar{u}}^\Delta|$. This motivates the terminology of samples of $\bar{\mu}$ being called *macro*-samples, while samples of $\bar{\mu}_{\bar{u}}^\Delta$ approximating the Dirac-delta distribution are called *micro*-samples.

To summarize, we are interested in Monte Carlo samples of $P_t(du|\bar{u})$ where $\bar{u} \sim \bar{\mu}$. The algorithm to draw them is as follows:

1. Draw *macro* samples $\bar{u}^1, \bar{u}^2, \dots, \bar{u}^{M_{\text{macro}}} \sim \bar{\mu}$,
2. For each macro sample $\bar{u}^i$ draw *micro* samples $\bar{u}_{\bar{u}^i}^1, \bar{u}_{\bar{u}^i}^2, \dots, \bar{u}_{\bar{u}^i}^{M_{\text{micro}}} \sim \bar{\mu}_{\bar{u}^i}^\Delta$,
3. Evolve each sample $\bar{u}_{\bar{u}^i}^j$ in time to get $u_{\bar{u}^i}^j(t)$,
4. Approximate each $P_t(du|\bar{u} = \bar{u}^i)$ by

$$P_t(du|\bar{u} = \bar{u}^i) \approx \frac{1}{M_{\text{micro}}} \sum_{j=1}^{M_{\text{micro}}} \delta_{u_{\bar{u}^i}^j(t)}. \tag{56}$$

This is the strategy that generates the ground-truth distribution, with respect to which we test GenCFD and the baselines.

SI 2.6 Datasets

In the Main Text, we have presented results with five challenging three-dimensional flow datasets, which we describe in detail below. Representative subsets consisting of a small number of sample trajectories for each test case are published open access on Zenodo [33] under a CC BY 4.0 license. The complete datasets are available under a CC BY 4.0 license in a Google Cloud storage bucket https://console.cloud.google.com/storage/browser/gencfd/.

SI 2.6.1 Taylor–Green Vortex (TGV)

We simulate the well-known Taylor–Green vortex [34] for the incompressible Navier–Stokes equations (2) in a probabilistic setting by adding small perturbations to the velocity field. The initial conditions are given by

$$\bar{u}_x(x, y, z) = A\cos(1\pi x)\sin(2\pi y)\sin(2\pi z) + \varepsilon_x(x, y, z), \tag{57}$$
$$\bar{u}_y(x, y, z) = B\sin(1\pi x)\cos(2\pi y)\sin(2\pi z) + \varepsilon_y(x, y, z), \tag{58}$$
$$\bar{u}_z(x, y, z) = C\sin(1\pi x)\sin(2\pi y)\cos(2\pi z) + \varepsilon_z(x, y, z), \tag{59}$$

where we choose $A = 1$, $B = -1$, $C = 0$ to fulfill the incompressibility constraint. The perturbations ε_x, ε_y, and ε_z are defined to be

$$\varepsilon_d(x, y, z) = \frac{1}{8}\sum_{(i,j,k)\in\{0,1\}^3} \delta_{d,i,j,k}\alpha_i(4\pi x)\alpha_j(4\pi y)\alpha_k(4\pi z), \quad \text{where } \alpha_i(x) = \begin{cases}\sin(x) & \text{if } i = 0,\\ \cos(x) & \text{if } i = 1,\end{cases} \tag{60}$$

where $\delta_{d,i,j,k} \sim \mathcal{U}_{[-0.025,0.025]}$.

For simulating a Dirac distributed initial condition, we fix the values $\delta_{d,i,j,k}$ and add a second perturbation of a similar form as ε_d to the flow field. The difference being that the frequency of the Fourier modes is doubled, and their amplitudes are chosen proportional to the mesh size.

Although the initial datum only contains a few large frequencies, the turbulent cascade into progressively smaller scales leads to the dynamic generation of higher frequencies, see Main Text Fig. 2 (A and B) for illustrations of the pointwise kinetic energy and vorticity intensity. Thus, the solution transitions from a laminar to a turbulent regime with time. We simulate this experiment with a spectral viscosity method implemented within the publicly available, state-of-the-art, GPU-optimized **Azeban** code [35] at a spatial resolution of $128 \times 128 \times 128$.

Here, the underlying solution operator maps the initial data to (trajectories of) the solution at later times. In the experiments conducted, the total time duration was scaled to $T = 2.0$. We test the solution at $T_{\text{test}} = 0.8$ and $T_{\text{test}} = 2.0$. The all-to-all training was performed using snapshots corresponding to the time points $\{0, 0.4, 0.8, 1.2, 1.6, 2.0\}$. There are 15 input-output pairs per trajectory in the training set.

SI 2.6.2 Cylindrical Shear Flow (CSF)

The cylindrical shear flow for the incompressible Navier–Stokes equations (3) is heavily inspired by the flat vortex sheet experiment in [16] and is introduced as a 3D equivalent to the latter [35]. The initial conditions are given by

$$\bar{u}_x(x, y, z) = \tanh\left(2\pi\frac{r - 0.25}{\rho}\right), \tag{61}$$

$$\bar{u}_y(x,y,z) = 0, \tag{62}$$
$$\bar{u}_z(x,y,z) = 0, \tag{63}$$

where $r^2 = (y - 0.5 + \sigma_y^j(x))^2 + (z - 0.5 + \sigma_z^j(x))^2$ and ρ is the smoothness parameter. We define the perturbations $\sigma_y^j(x)$ and $\sigma_z^j(x)$ in the following way: Let α_k^y and α_k^z be i.i.d. uniformly distributed on $[0,1]$ and let β_k^y and β_k^z be i.i.d. uniformly distributed on $[0, 2\pi]$. Then $\sigma_y^j(x)$ and σ_z^j are given by

$$\sigma_y^j(x) = \delta \sum_{k=1}^{p} \alpha_k^y \sin(2\pi k x - \beta_k^y), \tag{64}$$
$$\sigma_z^j(x) = \delta \sum_{k=1}^{p} \alpha_k^z \sin(2\pi k x - \beta_k^z). \tag{65}$$

These initial conditions are well defined in the limit $\rho \to 0$ where the interface between the flow directions becomes discontinuous and are then equal to

$$\bar{u}_x(x,y,z) = \begin{cases} -1 & \text{for } r \leq 0.25, \\ 1 & \text{otherwise,} \end{cases} \tag{66}$$
$$\bar{u}_y(x,y,z) = 0, \tag{67}$$
$$\bar{u}_z(x,y,z) = 0, \tag{68}$$

where r is defined as above.

For simulating a Dirac distributed initial condition, we fix the values of α_k^y, α_k^z, β_k^y, and β_k^z. Then we extend the perturbation by an additional three modes with amplitude proportional to the mesh size.

We choose $p = 10$ and $\delta = 0$ as our default configuration. The initial shear flow is then evolved by numerically solving the three-dimensional incompressible Navier–Stokes equations with the spectral viscosity based **Azeban** code [35]. The resulting approximate solutions follow a complex temporal evolution and contain a large range of small-scale eddies as seen in Main Text Fig. 1 (D). The dataset is also generated at the resolution of 128^3.

Again, the underlying solution operator maps the initial velocity field to the velocity field at later times. In the experiments conducted, the total time duration was scaled to $T = 1.0$. The all-to-all training was performed using snapshots corresponding to the time points $\{0, 0.25, 0.5, 0.75, 1.0\}$. There are 10 input-output pairs per trajectory in the training set.

SI 2.6.3 Cloud-Shock Interaction (CSI)

This dataset is the three-dimensional version of the well-known shock-bubble test case for compressible fluid flows [36, 37, 38] where a supersonic shock wave hits a high-density cloud (bubble) and leads to the excitation of shock waves while creating a zone of turbulent mixing in their wake.

For this experiment, we subdivide the domain $[0,1]^3$ into three subdomains. The initial condition is then initialized as a constant on each of these subdomains. We define

$$S = \{(x,y) \in [0,1]^3 \mid x \leq 0.05 + \sigma_x(y)\}, \tag{69}$$
$$C = \{(x,y) \in [0,1]^3 \mid r \leq 0.13 + \sigma_r(\operatorname{atan2}(y - 0.5, z - 0.5))\}, \tag{70}$$
$$E = [0,1]^3 \setminus (S \cup C), \tag{71}$$

where $r^2 = (x - 0.25)^2 + (y - 0.5)^2 + (z - 0.5)^2$ and σ_x, and σ_r are defined as

$$\sigma_x(y) = \frac{\delta}{\sum_{k=1}^{p} \alpha_k^x} \sum_{k=1}^{p} \alpha_k^x \cos(2\pi k(y + \beta_k^x)), \tag{72}$$

$$\sigma_r(\varphi) = \frac{\delta}{\sum_{k=1}^{p} \alpha_k^r} \sum_{k=1}^{p} \alpha_k^r \cos(2\pi k(\varphi + \beta_k^r)). \tag{73}$$

The parameters α_k^x, α_k^r, β_k^x, and β_k^r are all uniformly distributed in $[0,1]$. Furthermore, for the in-distribution data, we use $\delta = 0.06$ and $p = 10$. Combining all of this, the initial conditions for this experiment are given by

$$(\bar{\rho}, \bar{u}_x, \bar{u}_y, \bar{p}) = \begin{cases} (0.386859, 11.2536, 0, 167.345) & \text{if } (x,y,z) \in S, \\ (10, 0, 0, 1) & \text{if } (x,y,z) \in C, \\ (1, 0, 0, 1) & \text{if } (x,y,z) \in E. \end{cases} \tag{74}$$

For generating Dirac distributed initial conditions, the values of α_k^x, α_k^r, β_k^x, and β_k^r are fixed. Then the perturbations are extended by the next three higher modes with their amplitudes set to be proportional to the mesh size.

The dataset is simulated with the high-resolution finite volume GPU-optimized **Alsvinn** code of [38] at a resolution of 256^3.

For this dataset, the solution operator maps the initial conditions (density, momenta and energy) to the solution at later times. In the experiments we conducted, the total time duration was scaled to $T = 1.0$. The all-to-all training was performed using snapshots corresponding to the time points $\{0, 0.25, 0.5, 0.75, 1.0\}$. There are 10 input-output pairs per trajectory in the training set.

SI 2.6.4 Nozzle Flow (NF)

This dataset is generated from a three-dimensional fluid flow through a nozzle geometry of diameter $2l_\mathrm{c}$ and length $4l_\mathrm{c}$ into a larger domain of diameter $11l_\mathrm{c}$ and length $56l_\mathrm{c}$ that is filled with the same medium. The injection with a randomized inflow velocity profile of maximal magnitude u_c generates a wall-bounded turbulent pipe flow at $\mathrm{Re} = 10000$ up to $\mathrm{Re} = 20000$ inside the nozzle, where $\mathrm{Re} \coloneqq u_\mathrm{c} l_\mathrm{c}/\nu_\mathrm{c}$, $u_\mathrm{c} = 1$ m/s to $u_\mathrm{c} = 2$ m/s, $l_\mathrm{c} = 1$ m, and $\nu_\mathrm{c} = 1.0 \times 10^{-4}$ m^2/s. Consequently, in the larger domain a turbulent round jet is formed, see Main Text Fig. 3 (A). Turbulent jet flows appear in many engineering applications such as fluid mixing, combustion or acoustic control. The vortex generation method in the computational setup from [39] is modified such that random perturbations

$$u_\mathrm{vort}(x,y,z,t) = \mathrm{frac}(t) \frac{1}{2\pi} \sum_{i=1}^{n_\mathrm{vort}} \Gamma_i \frac{\left(1 - \exp\left(-\frac{|[x,y,z]-p_i|^2}{2\sigma^2}\right)\right)}{|[x,y,z] - p_i|^2} \left((p_i - [x,y,z]) \times d_\mathrm{in}\right) \tag{75}$$

are added to the inflow mean velocity

$$u_\mathrm{mean}(x,y,z,t) = \mathrm{frac}(t)[2.77u_\mathrm{c} \left(1 - \left(\frac{1}{l_\mathrm{c}} \left((x - 5.5l_\mathrm{c})^2 + (z - 5.5l_\mathrm{c})^2\right)^{\frac{1}{2}}\right)^{\frac{9}{8}}\right), 0, 0], \tag{76}$$

where

$$\Gamma_i = 4s_i \left(\frac{\pi}{6\ln(3) - 9\ln(2)} \frac{k_i A_\mathrm{in}}{n_\mathrm{vort}}\right)^{\frac{1}{2}} \tag{77}$$

and

$$k_i = \frac{3}{2}\left(|u_{\mathrm{mean}}(p_i)|I_{\mathrm{turb}}\right)^2 \tag{78}$$

denote the circulations per vortex, and the turbulent kinetic energies, respectively, for $i = 1, 2, \dots, n_{\mathrm{vort}}$, and $\mathrm{frac}(t) \in [0, 1]$ is polynomially increased to unity until $t = 0.5$ s. Here, $n_{\mathrm{vort}} = 50$ is the number of vortices of size $\sigma = 0.1 l_{\mathrm{c}}$ located at inlet positions

$$[0, r_i, \theta_i] = [0, l_{\mathrm{c}}\left(\gamma_i^{(r)}\right)^{\frac{1}{2}}, 2\pi\gamma_i^{(\theta)}] \tag{79}$$

with the respective Cartesian locations $p_i \in \mathbb{R}^3$ and signs s_i. The random parameters $\gamma_i^{(r)}$ and $\gamma_i^{(\theta)}$ are i.i.d. uniformly in $[0, 1)$, and s_i are i.i.d. discrete uniformly on $\{-1, 1\}$. Moreover, $I_{\mathrm{turb}} = 0.05$ is the turbulence intensity, A_{in} is the inlet area of the nozzle, and $d_{\mathrm{in}} \in \mathbb{R}^d$ is the normalized inflow direction. The mean velocity inlet profile (76) as well as the perturbation (75) are Langevin-combined [39] to synthetically produce a turbulent inflow velocity

$$u_{\mathrm{in}}(x, y, z, t_n) \coloneqq u_{\mathrm{mean}}(x, y, z, t_n) + u_{\mathrm{vort}}(x, y, z, t_n) - \frac{u_{\mathrm{vort}}(x, y, z, t_n) \cdot \nabla u_{\mathrm{in}}(x, y, z, t_{n-1})}{|\nabla u_{\mathrm{in}}(x, y, z, t_{n-1})|} d_{\mathrm{in}} \tag{80}$$

at a discrete timestep $t_n \in N$, where $u_{\mathrm{in}}(x, y, z, 0) = [0, 0, 0, 0]$. We use a lattice Boltzmann method with an LES model to approximate the initial boundary value problem in the nozzle geometry that is based on the weakly compressible Navier–Stokes equations at a Mach number of $\mathrm{Ma} = 4.7 \times 10^{-3}$ with the velocity inlet condition (80), a constant pressure boundary at the outlet, and no-slip boundary conditions at the remaining cylinder walls. We simulate this experiment with the open-source highly parallel C++ library **OpenLB** [40, 41] that scales efficiently on hundreds of GPGPU nodes [42]. For the NF experiment, the domain is discretized with 10.19×10^6 grid points at a resolution of $124 \times 124 \times 663$ and we compute a time horizon until $t = 130$ s with a step size $\Delta t = 2.479 \cdot 10^{-4}$.

Consequently, in this dataset, the solution operator maps the initial conditions and the boundary conditions (inflow velocity) to the velocity field at later times. In the experiments we conducted, the time horizon was rescaled and non-dimensionalized to $T = 1.3$, with the testing time set to $T_{\mathrm{test}} = 1.0$. Note that the all-to-all training was performed using snapshots corresponding to the time points $\{0, 0.2, 0.4, 0.6, 0.8, 1.0, 1.2\}$. Thus, there are 21 input-output pairs per trajectory in the training set.

SI 2.6.5 Dry Convective Planetary Boundary Layer (CBL)

This test case describes the growth of a CBL as encountered during a summer day [43]. Forced at the surface by solar radiative heating and weak geostrophic winds [44], warm plumes ascend to the top of the boundary layer (*boundary layer height* $z_i \approx 1$ km), balanced by larger scale downdrafts, resulting in turbulent dynamics spanning a wide range of scales. CBL dynamics are crucial to understanding the fundamental properties and sensitivities of the (strato) cumulus clouds that can form above a CBL as moisture is added to the simulation, which are in turn a major source of uncertainty in current climate projections [45].

In order to allow for larger time steps, an anelastic approximation to the fully compressible Navier–Stokes equations is adopted [46, 47], eliminating sound waves from the system. Given a hydrostatic reference state

$$\alpha_0 \frac{\partial p_0}{\partial x_3} = -g, \tag{81}$$

with reference profiles $\alpha_0(x_3) = \rho_0(x_3)^{-1}$ for specific volume and $p_0(x_3)$ for pressure, and gravity constant $g = 9.80665$ ms^{-2}, density changes are neglected in thermodynamic and continuity equations.

This leaves the dynamic pressure perturbation p' to be diagnosed by solving an elliptic equation at each time step.

The anelastic equations with velocities u_1, u_2, u_3 and entropy s as prognostic equations are then given by

$$\frac{\partial u_i}{\partial t} + \frac{1}{\rho_0}\frac{\partial(\rho_0 u_i u_j)}{\partial x_i} = -\frac{1}{\rho_0}\frac{\partial(\rho_0 \tau_{ij})}{\partial x_j} - \frac{\partial \alpha_0 p'}{\partial x_i} + b\delta_{13} - \epsilon_{ijk}\delta_{j3} f(u_k - U_{g,k}), \tag{82}$$

$$\frac{\partial s}{\partial t} + \frac{1}{\rho_0}\frac{\partial(\rho_0 u_i s)}{\partial x_i} = -\frac{1}{\rho_0}\frac{\partial(\rho_0 \gamma_i)}{\partial x_i}, \tag{83}$$

$$\frac{\partial \rho_0 u_i}{\partial x_i} = 0, \tag{84}$$

within the domain D and the time span $[0, T]$, where we use standard conventions for summing, the Kronecker delta δ_{ij}, and the Levi–Civita tensor ϵ_{ijk}. The Coriolis parameter $f = 0.376e-4\ \mathrm{s}^{-1}$ acts upon the difference to the large scale geostrophic wind U_g, and we refer to [46] for the definition of the buoyancy term b. As is characteristic for large eddy simulations (LES), which do not aim to resolve the smallest turbulent eddies, the effect of turbulent motion smaller than the grid size is modeled by the sub-grid scale (SGS) stresses τ_{ij} for velocity and γ_i for entropy. The radiative forcing is prescribed as horizontally constant heat flux Q_* at the lower boundary.

We solve the anelastic equations with the open source Python and Cython based **PyCLES** library [47] at a resolution of 128^3 and a grid spacing of 40 m and 16 m horizontally and vertically, respectively. In agreement with previous findings [48], we omit the modeling of the SGS terms ($\gamma_{s,i} = \tau_{ij} = 0$) and rely on the favorable dissipative properties of the WENO scheme which provide an *implicit large eddy simulation*.

The simulation is initialized with zero velocities and a horizontally constant profile of potential temperature

$$\theta = T\left(\frac{p_0}{p}\right)^{\frac{R}{c_p}}, \tag{85}$$

given by a characteristic profile of the shape

$$\theta(x_3) = \begin{cases} T_g, & 0 < x_3 < z_a, \\ T_g + (x_3 - z_a)(\partial_3\theta)_i, & z_a < x_3 < z_b, \\ T_g + (\partial_3\theta)_i(z_a - z_b) + (x_3 - z_b)(\partial_3\theta)_e, & \end{cases} \tag{86}$$

with dry constants $R = 287.1\ \mathrm{JK^{-1}kg^{-1}}$ and $c_p = 1004.0\ \mathrm{JK^{-1}kg^{-1}}$, and reference pressure p_0 at ground level. We fix the environmental profile, given by the lapse rate $(\partial_3\theta)_e = 0.003\ \mathrm{K\,m^{-1}}$ and a fixed intercept, to match the original test case formulation [43], and define the boundary layer height to be $z_i = (z_b - z_a)/2$. This leaves as free parameters of the initial condition the boundary layer temperature T_g, the initial boundary layer height z_i, and the inversion lapse rate (sharpness) $(\partial_3\theta)_i$. Together with the geostrophic wind U_g and the heat forcing Q_*, each realization of the CBL simulation is hence parameterized by five free parameters which we sample uniformly in the training distribution, as summarized in Table SI 2.

Based on this deterministic setup, convection is initialized by random perturbations at the lowest model levels. For the same initial profile of θ_0, different realizations of the CBL are hence obtained by varying the random seed. For the experiments in this paper, all training samples were generated with the same random seed (and varying input parameters), since there is no dependence on the exact initial perturbation after the model spinup phase. In order to sample a Dirac measure, however, the random seed is varied while keeping the input parameters fixed.

The boundary layer height z_i is computed as in [43] by the *maximum gradient method* as the horizontal mean of the vertical location with the largest temperature gradient. Due to the constant Q_* forcing, z_i grows linearly in time. In Fig. SI 22, we evaluate GenCFD against the numerical truth on horizontal statistics, including statistical moments of prognostic variables, the vertical temperature flux, and turbulent kinetic energy (TKE).

In the experiments conducted, the total duration of 7200 s in model time was scaled to $T = 2.4$. We test the solution at $T_{\text{test}} = 2.4$. The all-to-all training was performed using snapshots corresponding to the time points $\{1.2, 1.4, 1.6, 1.8, 2.0, 2.2, 2.4\}$. There are 21 input-output pairs per trajectory in the training set. During the testing phase, the model is conditioned on the time step $t = 1.2$, with the assumption that the spin-up phase has been completed by this point. Thus, the models aim to learn the statistics of the solution operator which maps initial conditions and parameters to solutions at later times.

SI 2.7 Details of Models and Hyperparameters

Here, we describe the selection procedure for GenCFD and the baselines.

GenCFD. In all experiments, axial attention was applied at every layer of the UViT denoiser. Each of the tested models consistently used 4 axial attention blocks and 8 attention heads. The UViT architecture (see Fig. SI 6) consisted of 3 downsampling layers, each with a downsampling ratio of 2. The intermediate channel dimension for the input and output spaces was set to 128, and the Fourier embedding dimension for physical (PDE) time and noise levels was also 128. In the CSF, TGV, CSI, and NF experiments, the number of channels per downsampling layer followed the configuration $(64, 128, 256)$. The resulting model is referred to as the *base* architecture. For the CBL benchmark, a smaller architecture, referred to as the *small* architecture, was used for improved memory efficiency and faster training. This version employed channels per layer in the sequence $(48, 96, 192)$. Table SI 3 presents the sizes of the models used in our experiments. Note that the ground-truth data is typically generated at a resolution that is higher than the resolution of the computational grid of the UViT denoiser (Table SI 3). Hence, the data is downsampled onto this computational grid using numerical downsampling.

Moreover, in Table SI 4, we outline the experimental details for each dataset, including the number of training samples, batch sizes, gradient steps (and corresponding epochs), as well as the number of GPUs and their memory capacity used for training. Each model was trained for approximately 72 to 120 hours, except for the one used in the NF benchmark, which completed training in just 24 hours. It is worth noting that training times could be significantly reduced by utilizing multiple GPUs with larger memory.

Baselines. The training and inference procedures differ between the baselines (FNO (FNO (see Table SI 5), C-FNO (see Table SI 6), and UViT) and GenCFD. The GenCFD model was trained without using early stopping, whereas the deterministic models were trained until convergence using early stopping to prevent overfitting. In every benchmark, we observed that the training of deterministic models invariably collapsed to the mean of the output distribution. Even when the deterministic models were trained for extended durations and allowed to overfit the training set, the outcome remained unchanged. The deterministic models completed training in approximately 24 to 48 hours. Note that the same number of training samples was used as in the GenCFD trainings.

For each experiment and baseline (except for CBL, due to memory constraints), cross-validation was conducted using a random grid search over a range of hyperparameters – typically 10 to 20 for the FNO models and 4 to 10 for UViT, respectively. Since the deterministic UViT model shares the same architecture as GenCFD across all benchmarks, only the learning rate was varied during the hyperparameter sweeps.

SI 2.8 Evaluation Metrics

Evaluation of the model's performance employs a suite of metrics to quantify the fidelity and diversity of the generated samples relative to the ground-truth distribution:

- **L^2-norm error between the mean** of the ground truth (μ_{exact}) and the approximated distribution (μ):
$$e_\mu = \|\mu_{\text{exact}} - \mu\|_2. \tag{87}$$
- **L^2-norm error between the standard deviation** of the ground truth (σ_{exact}) and the approximated distribution (σ):
$$e_\sigma = \|\sigma_{\text{exact}} - \sigma\|_2. \tag{88}$$
The standard deviation error is normalized with respect to the ground-truth norm.
- **L^2-norm error between the higher-order central moments** of the ground truth ($\mu'_{i,\text{exact}}$) and those of the approximated distribution (μ'_i):
$$e_{\mu'_i} = \|\mu'_{\text{i,exact}} - \mu'_i\|_2, \tag{89}$$
where the subindex i describes the order of the central moment, with $i \geq 3$ indicating higher-order moments beyond mean and variance. The prime ($'$) indicates that these are central moments, defined as:
$$\mu'_i = \mathbb{E}\left[(u - \mathbb{E}[u])^i\right], \tag{90}$$
where u denotes the random variable associated with the distribution.
- **Average 1-point Wasserstein distance** between the ground truth p_{exact} and the approximated distribution p (conditional and unconditional) computed over M spatial points:
$$W_s(p_{\text{exact}}, p) = \sum_{i=1}^{M} \left(\int_0^1 \left| F^{-1}_{exact}(u(x_i)) - F^{-1}(u(x_i)) \right|^s \, dx \right)^{1/s}, \tag{91}$$
with F being the CDFs.
- **Continuous ranked probability score (CRPS)**: Given an ensemble of predictions $U = \{u\}_{m=1}^{M}$, $x_m \sim p$, and a single observation u_{exact} from the ground-truth distributions $u_{\text{exact}} \sim p_{\text{exact}}$, the pointwise CRPS score is defined as
$$\text{CRPS}[U, u_{\text{exact}}] = \frac{1}{M} \sum_{m=1}^{M} \|u_m - u_{\text{exact}}\|_2^2 - \frac{1}{2M^2} \sum_{m=1}^{M} \sum_{j=1}^{M} \|u_m - u_j\|_2^2. \tag{92}$$
Given an ensemble of observations $U_{\text{exact}} = \{u_{\text{exact}}\}_{n=1}^{N}$, we can extend the definition of the CRPS as
$$\text{CRPS}[U, U_{\text{exact}}] = \frac{1}{N} \sum_{n=1}^{N} \text{CRPS}[U, u_{\text{exact},n}]. \tag{93}$$
It should be noted that $\text{CRPS}[U, U_{\text{exact}}]$ is a function of the spatial coordinates x. To get a single global indicator of the ensembles' similarities, the (relative) L^2-norm of $\text{CRPS}[U, U_{\text{exact}}]$ can be computed as
$$\text{CRPS}_G[U, U_{\text{exact}}] = \|\text{CRPS}[U, U_{\text{exact}}]\|_2^2. \tag{94}$$
Moreover, the CRPS (92) is also normalized with respect to the L^2-norm of the true observation u_{exact}.

- **Energy spectra**: Given a flow field $u : \mathbb{R}^d \to \mathbb{R}^d$ where d denotes the dimension of space, we denote its component-wise Fourier transform by $\hat{u} : \mathbb{R}^d \to \mathbb{C}^d$. The energy spectrum is then defined as
$$E_k = \frac{1}{2} \int_{|\xi|=k} \|\hat{u}(\xi)\|^2 \, \mathrm{d}\xi. \tag{95}$$
As our solutions $u^\Delta : \mathbb{T}^d \to \mathbb{R}^d$ lie on the d-dimensional torus $\mathbb{T}^d$, we make use of their discrete Fourier transforms $\hat{u}_k^\Delta$ in order to compute the energy spectrum. Furthermore, we integrate over the ball in L^1, as that aligns with the computational grid. All in all, the energy spectra of our discrete solutions are computed as
$$E_k = \frac{\Delta^d}{2} \sum_{\|\xi\|_1 = k} \left\| \hat{u}_\xi^\Delta \right\|^2. \tag{96}$$

The metrics used for each benchmark are summarized in Table SI 7.

SI 2.9 Details on Toy Model #1 of the Main Text

In the toy problem mentioned in the Main Text, we aim to mimic essential aspects of the behavior of turbulent flows, in particular the sensitive dependence of outputs on small perturbations of the inputs. To this end, we fix $\Delta = 1/N$ and consider a very simple one-dimensional model by setting $u, \bar{u} \in \mathbb{R}$ and introducing a sequence of one-dimensional mappings
$$\mathcal{S}^\Delta(\bar{u}) = m(\bar{u}) + s_N(\bar{u}), \quad s_N(\bar{u}) \coloneqq \Lambda(N\bar{u}), \tag{97}$$
with $m : \mathbb{R} \to \mathbb{R}$ being any *mean* function and Λ being the 1-periodic extension of the hat-function, with values $\Lambda(0) = \Lambda(1) = -1$, $\Lambda(1/2) = 1$. The parameter $\Delta = 1/N$ allows us to control the input-sensitivity of the underlying map $\mathcal{S}^\Delta$, increasing sensitivity as $\Delta \to 0$. Fix the initial measure $\bar{\mu} = \mathcal{U}[0, 1]$ to be the uniform measure on $[0, 1]$. Let $\mu^\Delta = (\mathcal{S}^\Delta \times \mathrm{Id})_\# \bar{\mu}$ denote the push-forward measure that we wish to approximate.

We observe from the Main Text Fig. 4 (A) how $\mathcal{S}^\Delta$ becomes more and more oscillatory as $\Delta \to 0$. In particular, it does not seem possible to realize a deterministic limit. It is also easy to show that $\mathrm{Lip}(\mathcal{S}^\Delta) \sim 4N \to \infty$. Nevertheless, we will later show that the $\Delta \to 0$ limit is well-defined *statistically* and the resulting conditional distribution is given by the uniform distribution $p(u \mid \bar{u}) = \mathcal{U}[m(\bar{u}) - 1, m(\bar{u}) + 1]$, centered around the mean m.

Thus, this toy problem mimics several relevant features of turbulent fluid flow such as i) no deterministic limit under mesh refinement, ii) unstable behavior of the numerical approximation operator $\mathcal{S}^\Delta$ in the limit, iii) the limit under mesh refinement is well-defined statistically and iv) the limit measure is not a Dirac but is *spread out*.

SI 2.9.1 Numerical Results

We implemented toy problem #1, and trained both deterministic models and diffusion models for various values of the parameter $\Delta > 0$.

Our theoretical considerations assumed a bounded Lipschitz constant L^* as a mathematical proxy for the limitations in learning oscillatory functions. In practice, the approximation of neural networks is limited by (at least) three factors: (i) the available training data, (ii) the model capacity (architecture), (iii) the approximate optimization by stochastic gradient descent.

Training Data. We train all models with a total of $N = 2048$ training samples, sampled uniformly on the interval $[0, 1]$. Since we focus on values of $\Delta \geq 0.02 \gg 1/N$, we expect the training samples to allow (in principle) for near-perfect interpolation of $\mathcal{S}^{\Delta}$.

Architecture. Our experiments are based on small models, all of which are chosen as vanilla dense, feedforward MLPs with ReLU activation. The deterministic model employs 2 hidden layers, and width 256, mapping a one-dimensional input (corresponding to $\bar{u}$) to a one-dimensional output,

$$\bar{u} \mapsto \Psi_{\mathrm{det}}(\bar{u}). \tag{98}$$

The diffusion model has depth 3 and width 512, mapping a three-dimensional input (corresponding to $(u; \bar{u}, \sigma)$) to a one-dimensional output, i.e.

$$(\bar{u}, u, \sigma) \mapsto D(u; \bar{u}, \sigma). \tag{99}$$

From limited experimentation during the implementation, the qualitative results of the experiments are observed to be robust to changes in the hyperparameters of the networks. Our goal is to examine the qualitative behavior of the deterministic and diffusion models when $\Delta \to 0$, independently. No attempt is made to provide a quantitative comparison between the deterministic and diffusion models (which is probably meaningless for these toy problems).

Training. All models are implemented and trained in **PyTorch**. We use the Adam optimizer with the learning rate set to 10^{-3}. Training is performed for a fixed number of epochs, for a maximum of 10000 epochs. The deterministic models are trained with MSE loss. For these one-dimensional, highly oscillatory toy problems, it is not always clear whether true stagnation of the training progress is observed. We therefore opt to illustrate not only the final results after 10000 epochs, but also the training progress. The observed difference between the deterministic and diffusion models during training is another interesting outcome of these toy problems.

Illustration of Results. To illustrate the trained deterministic models, we sample 2048 point in $\bar{u}$, and show a scatter plot of $(\bar{u}, \Psi(\bar{u}))$. Similarly, for the denoising models, we show a scatter plot as follows: We first sample 300 points uniformly in $\bar{u}$, and then generate 100 samples from the learned conditional distribution $p(u \,|\, \bar{u})$ for each point in $\bar{u}$, giving a scatter plot of 30000 samples in total. The noise process is chosen as a variance-preserving process, as in [21]. The backward denoising process is run with 200 timesteps and a cosine noise schedule [49].

SI 3 Detailed Theory

In this section, we present rigorous mathematical statements (and their proofs) that justify and expand on the theoretical discussion in the Main Text, specifically in the Section on Theoretical Results. Our aim is to explain, with rigorous mathematical analysis, the observations from our numerical experiments. In particular, we focus on explaining how i) diffusion models are able to learn the underlying probability distributions while deterministic ML baselines regress to mean fields and ii) diffusion models provide accurate spectral resolution (coverage) and are able to approximate small scales, right up to the smallest eddies in the data, while deterministic ML models have poor spectral resolution.

SI 3.1 Main Results

Characterization of Optimal Denoisers. As the time-conditioning in (32) is not relevant for this theoretical discussion, we omit it and consider the *denoiser training objective* or Diffusion loss as

$$\mathcal{J}(D_\theta) = \mathbb{E}_{\bar{u}\sim\bar{p}}\mathbb{E}_{u|\bar{u}}\mathbb{E}_{\eta\sim\mathcal{N}(0,\sigma^2)}\|D_\theta(u+\eta;\bar{u},\sigma) - u\|^2. \tag{100}$$

Hence, our aim is to remove noise from the noisy sample $u_\sigma = u + \eta$, $\eta \sim \mathcal{N}(0,\sigma^2)$, conditioned on the input $\bar{u}$, during the training of the denoiser. It turns out (see Lemma SI 3.9) that we can explicitly characterize the optimal denoiser $D_{\text{opt}} = \operatorname{argmin}_\theta \mathcal{J}(D_\theta)$ for any noise level $\sigma > 0$ by $D_{\text{opt}}(u_\sigma;\bar{u},\sigma) = \mathbb{E}[u \,|\, (\bar{u}, u_\sigma)]$. Therefore, if the noise process has ended up in a location u_σ, the optimal denoiser considers the distribution of the conditional random variable u given $(\bar{u}, u_\sigma)$, i.e. all possible origins u of the noise process conditioned on the input $\bar{u}$ and the noised sample u_σ, and selects the most likely origin as the expected value in this distribution.

This key observation can be used to further characterize the optimal denoiser in the zero-noise ($\sigma \to 0$) limit (see Proposition SI 3.11). In particular, we prove that, in this limit, the optimal denoiser $D_{\text{opt}}(w;\bar{u},\sigma = 0)$ evaluated at a point w, is identified with the closest point w^* in the support of $p(u \,|\, \bar{u})$, corresponding to a projection onto the data manifold. An immediate consequence of the identity $D_{\text{opt}}(u_\sigma;\bar{u},\sigma) = \mathbb{E}[u \,|\, (\bar{u}, u_\sigma)]$ for $\sigma > 0$ is the fact that

Proposition SI 3.1. *If $u \,|\, \bar{u}$ is in fact deterministic, i.e. $u = \mathcal{F}(\bar{u})$, then $D_{\text{opt}}(u_\sigma;\bar{u},\sigma) \equiv \mathcal{F}(\bar{u})$ for all $\bar{u}, \sigma$.*

The above proposition makes it clear that, if a conditional distribution is generated by an underlying deterministic map $\mathcal{F}$, then the optimal denoiser will simply collapse to this deterministic map. In practice, the diffusion model is trained on

$$\mathcal{J}^\Delta(D_\theta) = \mathbb{E}_{\bar{u}\sim\bar{p}}\mathbb{E}_{u^\Delta\,|\,\bar{u}}\mathbb{E}_{\eta\sim\mathcal{N}(0,\sigma^2)}\|D_\theta(u^\Delta+\eta;\bar{u},\sigma) - u^\Delta\|^2, \tag{101}$$

with $u^\Delta \,|\, \bar{u} = \mathcal{S}^\Delta(\bar{u})$ obtained from a numerical solver. Given that (101) stems from the *deterministic* approximate solution operator $\mathcal{S}^\Delta$, the optimal denoiser should be concentrated around $\mathcal{S}^\Delta(\bar{u})$ for any $\Delta > 0$. Hence, there should be no difference between using a denoiser training objective (101) and a purely deterministic loss,

$$\mathcal{J}^\Delta_{\text{det}}(\Psi_\theta) = \mathbb{E}_{\bar{u}\sim\bar{p}}\|\Psi_\theta(\bar{u}) - \mathcal{S}^\Delta(\bar{u})\|^2. \tag{102}$$

In other words, this result suggests *there should be very little difference between the simulations carried out using the ML baselines and our proposed conditional-diffusion model, seemingly contradicting our experimental observations.* Therefore, we need to formulate a more refined analysis in order to resolve this apparent contradiction.

Input Sensitivity. To this end, we recall that, for fluid flows, the approximate solution operator outputs solutions which contain energetic eddies across a very large range of scales, exhibiting chaotic dynamics. Consequently, the behavior of $\mathcal{S}^\Delta$ asymptotically as $\Delta \to 0$ is very oscillatory and unstable [17, 16]. It is precisely this lack of stability in the $\Delta \to 0$ limit that could prevent us from realizing an *unconstrained* optimal denoiser within the class of neural networks. Our refined theoretical analysis is based on the assumption of a *mismatch in input sensitivity*: the underlying solution operator $\mathcal{S}^\Delta$ is highly sensitive to small perturbations $\delta\bar{u}$. Even perturbations of a small size $\|\delta\bar{u}\|_{\mathcal{X}} \sim \tilde{\epsilon}$ can entail

$$\|\mathcal{S}^\Delta(\bar{u} + \delta\bar{u}) - \mathcal{S}^\Delta(\bar{u})\|_{\mathcal{Y}} \gg 1. \tag{103}$$

This is precisely the *sensitivity hypothesis* of the underlying operators in the Main Text.

In contrast, we argue that our trained neural network model could not be able to match this input sensitivity; i.e. a sufficiently small input perturbation $\|\delta\bar{u}\|_{\mathcal{X}} \sim \tilde{\epsilon} \ll 1$ only leads to a small output perturbation,

$$\|\Psi_\theta(\bar{u} + \delta\bar{u}) - \Psi_\theta(\bar{u})\|_{\mathcal{Y}} \ll 1 \quad \text{and} \quad \|D_\theta(u_\sigma; \bar{u} + \delta\bar{u}, \sigma) - D_\theta(u_\sigma; \bar{u}, \sigma)\|_{\mathcal{Y}} \ll 1. \tag{104}$$

This is the *insensitivity hypothesis for neural networks*, that is discussed in the Main Text. Why does this hypothesis hold? Why are neural networks *insensitive to very small perturbations in inputs*?

A possible answer lies in the notion that *neural networks learn and generalize well at the edge of chaos* [50, 51]. In this framework based on statistical physics, the forward pass of neural networks is viewed as a dynamical system. The underlying principle states that optimal computational capability of neural networks (or other systems, including the brain) emerges when the dynamical system is at a critical point between order and chaos. A relevant *measure of chaos* is the Lyapunov exponent of the input-to-output map, defined as $\gamma \approx \frac{1}{T}\log(|\delta x_T|/|\delta x_0|)$ [50, eq. (S27)], defined in terms of a temporal parameter T (which can be the depth for deep networks or the number of rollout steps for autoregressive neural network predictions) and the quotient of the magnitude of output perturbations $|\delta x_T|$ versus the magnitude of (small) input perturbations $|\delta x_0|$. This quantity is equivalent to the Lipschitz constant $\mathrm{Lip}(\Psi_\theta) \approx |\delta x_T|/|\delta x_0|$. Based on both theoretical considerations and extensive empirical evidence, it has been demonstrated that neural networks maximize their performance and generalization capability when $\gamma \approx 0$ [50], i.e. when $\mathrm{Lip}(\Psi_\theta) \approx 1$. Thus, this *edge of chaos* principle leads to an obvious tension (or indeed contradiction) between the opposing goals of keeping Lipschitz constants of neural networks *bounded of order* 1 to ensure optimal performance, and the requirement of having *exponentially large* Lipschitz constants as would be required to fit the exponential input-sensitivity of turbulent/chaotic flows.

A second and related motivation for the assumption of insensitivity of neural networks is by viewing it as a high-dimensional analogue of the well-known spectral bias of neural networks [52]. As observed for function regression in one dimension, neural networks are biased against fitting high-order Fourier modes. In that context, the lack of regularity of an underlying mapping, i.e. its input sensitivity, is encoded by a slow decay of the Fourier spectrum; fitting a highly input-sensitive function by a neural network necessitates the accurate approximation of high-order Fourier modes, and hence overcoming this observed spectral bias.

Finally, neural networks are trained by variants of stochastic gradient descent algorithms that require that the underlying gradients are well-behaved, i.e, are bounded and of order one. Otherwise, the well-known *exploding and vanishing gradient problem* [53] will be encountered and will lead to a failure of neural network training. Clearly, these gradients are related to the Lipschitz constants of the neural network Ψ_θ with respect to the inputs. Hence, ensuring well-bounded gradients, necessary for neural network training, also adds weight to the insensitivity hypothesis that we propose here.

Thus, we argue that our assumption of a mismatch in input-sensitivity is natural, given the underlying chaotic dynamics of fluid flows or any sensitive map. While leaving a more rigorous investigation of this assumption for future work, we posit it here as a postulate, based on which theoretical consequences are to be derived below. This will allow us to explain several of our empirical observations.

Lipschitz Continuity Quantifies Input Sensitivity. The informal inequality (103) implies that discretized solution operators of interest have very large Lipschitz constants, i.e. that $\mathrm{Lip}(\mathcal{S}^\Delta) \gg 1$ for small Δ. To capture this in our mathematical analysis, we argue that these Lipschitz constants are so large, that for all practical purposes the relevant regime is captured by assuming $\mathrm{Lip}(\mathcal{S}^\Delta) \to \infty$, as $\Delta \to 0$; in fact, for PDEs such as the Navier–Stokes equations, whether $\mathrm{Lip}(\mathcal{S}^\Delta)$ remains finite or not is a long-standing open problem, and the potential ill-posedness (in the sense of Hadamard [54]) in the limit $\Delta \to 0$ is a realistic possibility. In contrast, we surmise that training by stochastic gradient descent tends to bias neural networks towards stability and away from highly oscillatory multiscale mappings. We mathematically formulate this hypothesis as follows:

Hypothesis SI 3.2. *Practical minimization of the denoiser objective* (100) *is only possible within a subclass of mappings D_θ satisfying a Lipschitz bound* $\mathrm{Lip}(D_\theta) \le L^*$*, for some cut-off $L^* \ge 1$.*

In fact, the Lipschitz bound is one possible mathematical requirement for ruling out wild oscillations and can be replaced by other equivalent criteria such as bounds on total variation or conditions on band-limited approximations [55]. Under this hypothesis, it is straightforward to show that *constrained minimization* of the deterministic training objective (102), within the model class specified by hypothesis SI 3.2, *cannot approximate the true minimizer*. This merely reflects the fact that $\mathrm{Lip}(\mathcal{S}^\Delta) \gg L^*$, as $\Delta \to 0$, and hence the optimal $D^\Delta_{\mathrm{opt}} = \mathcal{S}^\Delta$ (cp. Proposition SI 3.1) has a divergent Lipschitz constant in this limit. In particular, the unconstrained minimizer is unstable and cannot be approximated under Hypothesis SI 3.2, highlighting the potential role of instabilities and multiscale structure of the underlying operators in hindering the success of deterministic approximations of the solution operator of turbulent flows by neural networks.

Statistical Computation with Diffusion Models is still Tractable. On the other hand, how can training of the denoiser (100) within the model class of Hypothesis SI 3.2 lead to an accurate statistical computation of fluid flows? A positive answer is provided by the following proposition (see p. 42 for a proof):

Proposition SI 3.3. *Let μ, μ^Δ be two probability measures with bounded support on $\{|u| \le M\}$. Assume that the optimal conditional denoiser $D_{\mathrm{opt}}(u; \bar{u}, \sigma)$ for μ is L^*-Lipschitz continuous for some $L^* \ge 1$. Let D^Δ be the optimal* constrained *denoiser D^Δ for μ^Δ,*

$$D^\Delta(u; \bar{u}, \sigma) = \underset{\mathrm{Lip}(D_\theta) \le L^*}{\mathrm{argmin}} \, \mathcal{J}^\Delta(D_\theta, \sigma). \tag{105}$$

Then, we have

$$\mathbb{E}_{(u,\bar{u})\sim\mu} \mathbb{E}_{\eta\sim\mathcal{N}(0,\sigma^2)} \left\| D^\Delta(u+\eta; \bar{u}, \sigma) - D_{\mathrm{opt}}(u+\eta; \bar{u}, \sigma) \right\|^2 \le C L^* W_1(\mu^\Delta, \mu), \quad \forall \, \sigma > 0, \tag{106}$$

with constant C depending on M, but otherwise independent of μ^Δ, μ, and independent of L^* and σ.

As the whole premise of statistical computation of fluids, backed up by the theory of statistical solutions [15, 16], rests on the observation that $W_1(\mu^\Delta, \mu) \to 0$ as $\Delta \to 0$ [16, 17, 35], we see from (106) that the *constrained* denoiser achieves an almost optimal loss for μ, even though in this setting, we may have $\mathrm{Lip}(D^\Delta_{\mathrm{opt}}) = \mathrm{Lip}(\mathcal{S}^\Delta) \to \infty$, and D^Δ_θ will not be able to approximate the true optimizer $D^\Delta_{\mathrm{opt}}(u; \bar{u}, \sigma) = \mathcal{S}^\Delta(\bar{u})$ at any training resolution $\Delta \ll 1$.

Hence, Proposition SI 3.3 reveals the *mechanism* through which a diffusion model can leverage the unstable and multiscale nature of the underlying operator (modeling fluid flow for instance) to enable accurate statistical computation even though deterministic approximation in this context is not tractable.

The key assumption in Proposition SI 3.3 is the Lipschitz continuity of the optimal denoiser. Due to our current lack of mathematical understanding of the fine properties of statistical solutions, an end-to-end rigorous proof guaranteeing this property for fluid flows remains out of reach with existing mathematical tools. Furthermore, a highly technical proof would not necessarily shed further light on the fundamental mechanisms through which diffusion models work. Instead, we here choose another approach and present two *solvable toy models* which mimic relevant aspects of the behavior of turbulent fluids while being analytically tractable. These problems shed further light on the fundamental difficulties encountered by deterministic models, and illustrate how such difficulties can be overcome by probabilistic diffusion models.

SI 3.2 Toy Model #1: Illustrating the Consequences of Input Sensitivity Mismatch

We recall that toy model #1 is a one-dimensional model which mimics essential aspects of the behavior of turbulent fluid flows (see Sec. SI 2.9). At the same time, it is analytically tractable and allows for a rigorous mathematical analysis that we describe here. Recall that we fix $\Delta = 1/N$ and that, for $u, \bar{u} \in \mathbb{R}$, we have introduced a sequence of one-dimensional mappings,

$$\mathcal{S}^{\Delta}(\bar{u}) = m(\bar{u}) + s_N(\bar{u}), \quad s_N(\bar{u}) \coloneqq \Lambda(N\bar{u}), \tag{107}$$

where $m : \mathbb{R} \mapsto \mathbb{R}$ is any *mean* function and Λ is a 1-periodic hat-function. We fix the initial measure $\bar{\mu} = \mathcal{U}([0, 1])$ to be the uniform measure on $[0, 1]$. We observe from Fig. SI 34 how $\mathcal{S}^{\Delta}$ becomes more and more oscillatory as $\Delta \to 0$. In particular, it does not seem possible to realize a deterministic limit. It is also easy to show that $\mathrm{Lip}(\mathcal{S}^{\Delta}) \sim 4N \to \infty$.

We now study L^*-Lipschitz minimizers of the deterministic loss

$$\mathcal{J}^{\Delta}_{\text{det}}(\Psi_\theta) = \mathbb{E}_{\bar{u}\sim\bar{\mu}} |\Psi_\theta(\bar{u}) - \mathcal{S}^{\Delta}(\bar{u})|^2, \tag{108}$$

and the conditional diffusion training objective

$$\mathcal{J}^{\Delta}(D_\theta) = \mathbb{E}_{\bar{u}\sim p_{\text{prior}}} \mathbb{E}_{u^{\Delta} \mid \bar{u}} \mathbb{E}_{\eta\sim\mathcal{N}(0,\sigma^2)} |D_\theta(u^{\Delta} + \eta; \bar{u}, \sigma) - u^{\Delta}|^2, \tag{109}$$

where $u^{\Delta} \mid \bar{u} = \mathcal{S}^{\Delta}(\bar{u})$. The sensitivity hypothesis in the Main Text suggests that, for some sensitivity scale $\tilde{\epsilon} > 0$, and $\delta\bar{u} \sim \mathcal{U}([-\tilde{\epsilon}, \tilde{\epsilon}])$, the Lipschitz optimizer of $\mathcal{J}^{\Delta}_{\text{det}}$ satisfies $\Psi^{\Delta}(\bar{u}) \approx \mathbb{E}_{\delta\bar{u}} \left[\mathcal{S}^{\Delta}(\bar{u} + \delta\bar{u})\right]$, while the constrained optimizer of $\mathcal{J}^{\Delta}$ is approximately equal to the optimal denoiser for $\mathrm{Law}_{\delta\bar{u}} \left[\mathcal{S}^{\Delta}(\bar{u} + \delta\bar{u})\right]$. Our goal is to make this intuition rigorous, via asymptotic analysis as $\Delta \to 0$.

Remark SI 3.4 (Leading-order Analysis)**.** *With the notation and assumptions above, we have to leading order in* $\tilde{\epsilon}$,

$$\mathcal{S}^{\Delta}(\bar{u} + \delta\bar{u}) = \mathcal{S}^{\Delta}(\bar{u}) + O(N\tilde{\epsilon}), \tag{110}$$

$$m(\bar{u} + \delta\bar{u}) = m(\bar{u}) + O(\mathrm{Lip}(m)\tilde{\epsilon}), \tag{111}$$

$$\Psi_\theta(\bar{u} + \delta\bar{u}) = \Psi_\theta(\bar{u}) + O(L^*\tilde{\epsilon}). \tag{112}$$

We will be interested in the regime $\mathrm{Lip}(m) \sim L^* \ll N = \Delta^{-1}$, *where the gap* $L^* \ll \Delta^{-1}$ *corresponds to a* sensitivity mismatch *between* Ψ_θ *and* $\mathcal{S}^{\Delta}$. *Our main observation is that for* $\Delta \ll \tilde{\epsilon} \ll 1/L^*$, *the remainder terms for* Ψ_θ *and* m *can be neglected, but this is clearly not admissible for* $\mathcal{S}^{\Delta}$. *As a*

consequence, Ψ_θ cannot accurately capture the variation of $\mathcal{S}^\Delta$ at input scale $\tilde{\epsilon}$. To enable rigorous analysis while capturing this relevant regime, we will fix $L^*, \tilde{\epsilon}$ and consider the asymptotic limit $\Delta \to 0$, in the following.

Deterministic Models Collapse to the Mean. We denote the constrained optimizer of the deterministic problem formulation by,

$$\Psi^\Delta := \operatorname*{argmin}_{\mathrm{Lip}(\Psi_\theta)\le L^*} \mathcal{J}^\Delta_{\mathrm{det}}(\Psi_\theta). \tag{113}$$

We note that the underlying map $\mathcal{S}^\Delta$ has the smallest length-scale Δ, whereas the smallest length scale of Ψ^Δ is uniformly bounded due to the imposed Lipschitz bound. Thus, in the limit $\Delta \to 0$, the scale separation between the approximated maps $\mathcal{S}^\Delta$ and the approximants Ψ^Δ increases arbitrarily. As argued in Remark SI 3.4, it is in this limit that we can expect our leading-order analysis of the Main Text to be rigorously justified.

We now assume that the mean function $m(\bar{u})$ is L^*-Lipschitz. We fix a (arbitrary) constant $\tilde{\epsilon} > 0$. The discussion in the Main Text is based on the approximate identity $\Psi_\theta(\bar{u} + \delta\bar{u}) \approx \Psi_\theta(\bar{u})$ for $|\delta\bar{u}| \le \tilde{\epsilon}$. Since we assume that $\Psi_\theta(\bar{u})$ and $m(\bar{u})$ obey the same Lipschitz bound, we also expect that $m(\bar{u} + \delta\bar{u}) \approx m(\bar{u})$ to the same accuracy, and hence

$$\mathcal{S}^\Delta(\bar{u} + \delta\bar{u}) = m(\bar{u} + \delta\bar{u}) + s_N(\bar{u} + \delta\bar{u}) \approx m(\bar{u}) + s_N(\bar{u} + \delta\bar{u}). \tag{114}$$

This motivates the following definition:

$$\tilde{\Psi}^\Delta(\bar{u}) := m(\bar{u}) + \mathbb{E}_{\delta\bar{u}}\left[s_N(\bar{u} + \delta\bar{u})\right], \quad \delta\bar{u} \sim \mathcal{U}([-\tilde{\epsilon}, \tilde{\epsilon}]). \tag{115}$$

Thus, we have $\tilde{\Psi}^\Delta(\bar{u}) \approx \mathbb{E}_{\delta\bar{u}}\left[\mathcal{S}^\Delta(\bar{u} + \delta\bar{u})\right] \approx \Psi^\Delta(\bar{u})$, under the assumptions of the Main Text. We next confirm this intuition by showing that Ψ^Δ and $\tilde{\Psi}^\Delta$ are asymptotically equivalent, as $\Delta \to 0$.

Proposition SI 3.5. *With the definitions above, we have*

$$\lim_{\Delta\to 0} \mathbb{E}_{\bar{u}\sim\bar{\mu}} |\Psi^\Delta(\bar{u}) - \tilde{\Psi}^\Delta(\bar{u})|^2 = 0, \quad \text{and} \quad \lim_{\Delta\to 0} \mathbb{E}_{\bar{u}\sim\bar{\mu}} |\Psi^\Delta(\bar{u}) - m(\bar{u})|^2 = 0. \tag{116}$$

The last proposition rigorously justifies the approximate identity $\Psi^\Delta(\bar{u}) \approx \mathbb{E}_{\delta\bar{u}}\left[\mathcal{S}^\Delta(\bar{u} + \delta\bar{u})\right]$, provided $\tilde{\epsilon}$ is chosen sufficiently small so that $m(\bar{u} + \delta\bar{u}) \approx m(\bar{u})$, and shows that the optimal constrained model Ψ^Δ collapses to the mean, in the limit $\Delta \to 0$.

Probabilistic Models Predict Uncertainty. We next consider the probabilistic problem formulation of conditional diffusion models. We denote by

$$D^\Delta(u_\sigma; \bar{u}, \sigma) := \operatorname*{argmin}_{\mathrm{Lip}(D_\theta)\le L^*} \mathcal{J}^\Delta(D_\theta), \tag{117}$$

the optimal constrained denoiser for $\mu^\Delta(du \,|\, \bar{u}) = \delta(u - \mathcal{S}^\Delta(\bar{u}))$ and with $\bar{u} \sim \mathcal{U}([0,1])$. We again assume that $m(\bar{u})$ is Lipschitz continuous. Again, under the assumptions of the Main Text, we then have $\mathcal{S}^\Delta(\bar{u} + \delta\bar{u}) \approx m(\bar{u}) + s_N(\bar{u} + \delta\bar{u})$. We thus consider the conditional probability,

$$\nu^\Delta(du \,|\, \bar{u}) := \mathrm{Law}_{\delta\bar{u}}\left[m(\bar{u}) + s_N(\bar{u} + \delta\bar{u})\right], \quad \delta\bar{u} \sim \mathcal{U}([-\tilde{\epsilon}, \tilde{\epsilon}]), \tag{118}$$

for which $\nu^\Delta(du \,|\, \bar{u}) \approx \mathrm{Law}_{\delta\bar{u}}\left[\mathcal{S}^\Delta(\bar{u} + \delta\bar{u})\right]$, consistent with the discussion in the Main Text. We denote the optimal (unconstrained) denoiser of $\nu^\Delta(du \,|\, \bar{u})$ by,

$$\tilde{D}^\Delta(u_\sigma; \bar{u}, \sigma) := \operatorname*{argmin}_{D} \mathbb{E}_{\bar{u}\sim\bar{\mu}} \mathbb{E}_{u\sim\nu^\Delta(\,\cdot\,|\,\bar{u})} \mathbb{E}_{\eta\sim\mathcal{N}(0,\sigma^2)} \|D(u + \eta; \bar{u}, \sigma) - u\|^2. \tag{119}$$

The difference between D^Δ and $\tilde{D}^\Delta$ is that u is sampled from $\mu^\Delta(du \,|\, \bar{u})$ and $\nu^\Delta(du \,|\, \bar{u})$, respectively. In addition, D^Δ is a *constrained* minimizer with $\mathrm{Lip}(D^\Delta) \le L^*$ imposed, whereas $\tilde{D}^\Delta$ is an *unconstrained* minimizer.

It turns out that ν^Δ is asymptotically equivalent to a simpler measure μ, as $\Delta \to 0$: We thus finally define $\mu \in \mathrm{Prob}(\mathbb{R} \times [0,1])$ as the uniform measure on

$$\mathcal{I}(m) := \{(u, \bar{u}) \in \mathbb{R} \times [0,1] \,|\, u \in [m(\bar{u}) - 1, m(\bar{u}) + 1]\}, \tag{120}$$

so that $\mu(du \,|\, \bar{u}) = \mathcal{U}([m(\bar{u}) - 1, m(\bar{u}) + 1])$. It will be shown in Lemma SI 3.15 that $\nu^\Delta \to \mu$.

The following result shows that the optimal constrained denoiser for $\mu^\Delta = \delta(u - \mathcal{S}^\Delta(\bar{u}))$ is asymptotically equivalent to the optimal (unconstrained) denoiser for $\nu^\Delta(du \,|\, \bar{u}) = \mathrm{Law}_{\delta\bar{u}}\,[m(\bar{u}) + s_N(\bar{u} + \delta\bar{u})] \approx \mathrm{Law}_{\delta\bar{u}}\left[\mathcal{S}^\Delta(\bar{u} + \delta\bar{u})\right]$. This rigorously justifies the conclusion drawn from the sensitivity hypothesis of the Main Text, in the regime $\Delta \ll 1/L^*$.

Proposition SI 3.6. *With the definitions above, and for L^* a constant sufficiently large depending only on the Lipschitz constant of $m(\bar{u})$, we have*

$$\lim_{\Delta \to 0} \mathbb{E}_{(u,\bar{u}) \sim \mu} \mathbb{E}_{\eta \sim \mathcal{N}(0,\sigma^2)} \left\| D^\Delta(u + \eta; \bar{u}, \sigma) - \tilde{D}^\Delta(u + \eta; \bar{u}, \sigma) \right\|^2 = 0, \tag{121}$$

uniformly in $\sigma > 0$.

SI 3.3 Toy Model #2: Illustrating Spectral Accuracy of Diffusion Models

From the experimental results in the Main Text (specifically Main Text Fig. 2 (F)), we observed that neural networks trained to minimize least square errors have a very small effective spectrum. The general ideas that went into the toy model of the last section can also be used to gain intuition regarding the success of diffusion models in reproducing correct energy spectra. The hypothesis is again that deterministically trained models cannot capture highly oscillatory behavior, causing them to collapse to the mean in the oscillatory limit.

SI 3.3.1 Motivation

We now consider a (translation-equivariant) equation like the Navier–Stokes equations. Assume we have an accurate approximation $\Psi \approx \mathcal{S}$ of the corresponding solution operator (or of $\mathcal{S}^\Delta$ for small $\Delta > 0$). For a given input $\bar{u}$, let $u = \mathcal{S}(\bar{u})$ be the corresponding solution. We define a parametric path in the input function space, $h \mapsto \bar{u}_h := \bar{u}(\,\cdot\, + h)$. We note that, by translation-equivariance of $\mathcal{S}$, we have $\mathcal{S}(\bar{u}_h) = u_h := u(\,\cdot\, + h)$. If Ψ is a good approximation of $\mathcal{S}$, then by assumption, we have

$$\Psi(\bar{u}_h) \approx u_h, \quad \forall h, \tag{122}$$

and for any continuous linear functional $\ell : L^2(D) \to \mathbb{R}$, we also have

$$\langle \ell, \Psi(\bar{u}_h) \rangle \approx \langle \ell, u_h \rangle, \quad \forall h. \tag{123}$$

But now, consider the Fourier expansion:

$$u(x) = \sum_{k \in \mathbb{Z}^d} \hat{u}(k) e^{ikx} \tag{124}$$

and define ℓ as the projection onto the k-th Fourier mode. Then

$$h \mapsto \langle \ell, \Psi(\bar{u}_h) \rangle \approx \langle \ell, \mathcal{S}(\bar{u}_h) \rangle = \hat{u}(k) e^{ikh}. \tag{125}$$

If $\|\Psi(\bar{u}_h) - \mathcal{S}(\bar{u}_h)\|_{L^2} \le \epsilon \ll 1$, then clearly, we must also have

$$\left| \langle \ell, \Psi(\bar{u}_h) \rangle - \hat{u}(k) e^{ikh} \right| \le \|\Psi(\bar{u}_h) - \mathcal{S}(\bar{u}_h)\|_{L^2} \le \epsilon. \tag{126}$$

There are only two options for this upper bound to hold: Either $|\hat{u}(k)| \lesssim \epsilon$ is inherently small (in which case $\langle \ell, \Psi(\bar{u}_h) \rangle \approx 0$ would do), or $|\hat{u}(k)| \gg \epsilon$ is not small, in which case $h \mapsto \langle \ell, \Psi(\bar{u}_h) \rangle / \hat{u}(k)$ must be a good approximation of the oscillatory function $h \mapsto e^{ikh}$. Just as in the previous section, if Ψ is constrained to be non-oscillatory, then it is impossible to achieve a highly accurate approximation of $h \mapsto e^{ikh}$ for large k. Instead, we expect to see a collapse to the mean in the limit $|k| \to \infty$.

SI 3.3.2 Model

The discussion above shows that for relevant solution operators $\mathcal{S}$ in fluid dynamics, with solutions exhibiting slowly decaying Fourier spectrum, the mapping

$$\bar{u}_h \mapsto \mathcal{S}(\bar{u}_h), \tag{127}$$

can be considered oscillatory in a sense related to Fourier analysis. The following toy model replaces the dependence on the input function $\bar{u}_h$ by a dependence on $h \in [0, 1]$, resulting in the following oscillatory model with parameter $k \in \mathbb{N}$:

$$h \mapsto \mathcal{S}^{(k)}(h), \quad \mathcal{S}^{(k)}(h) := (\cos(2\pi kh), \sin(2\pi kh)) \in \mathbb{R}^2. \tag{128}$$

This toy problem captures a core difficulty in accurately reproducing energy spectra for fluid flows: namely, Fourier modes of the solution at high wavenumber ($k \gg 1$) are increasingly sensitive to small perturbations of the initial data. The initial data is here replaced by $h \in [0, 1]$. In comparison to the previous toy problem, an additional feature of this toy model lies in the fact that the outputs are constrained to lie on the unit circle, no matter what value the wavenumber k assumes. This *toy constraint* is designed to mimic real physical laws (constraints) such as energy balance in fluid flows, and represents a stable statistical property (akin to the robustness of energy spectra in fluid flows).

For this toy problem, our analysis suggests that a deterministically trained model will collapse to $(0, 0)$ for large k. In contrast, the analysis presented below suggests that a practically trained conditional diffusion model will instead produce a denoiser,

$$D_\theta(u; h, \sigma) \approx \begin{cases} (\cos(2\pi kh), \sin(2\pi kh)), & (k \sim 1), \\ u/|u|, & (k \gg 1). \end{cases} \tag{129}$$

The conditional probability distribution corresponding to this denoiser is deterministic for small/moderate k, but it is non-deterministic for large k. The limiting denoiser as $k \to \infty$ pushes the noise distribution toward a uniform distribution on the circle, which is the correct statistical limit of the above oscillatory map.

SI 3.3.3 Theory

We will study L^*-Lipschitz minimizers of the deterministic loss,

$$\mathcal{J}^{(k)}_{\mathrm{det}}(\Psi_\theta) = \mathbb{E}_{h\sim\bar{\mu}} |\Psi_\theta(h) - \mathcal{S}^{(k)}(h)|^2, \tag{130}$$

with $\bar{\mu} = \mathcal{U}([0,1])$ the uniform measure, and the conditional diffusion training objective,

$$\mathcal{J}^{(k)}(D_\theta) = \mathbb{E}_{h\sim\bar{\mu}}\mathbb{E}_{u\,|\,h}\mathbb{E}_{\eta\sim\mathcal{N}(0,\sigma^2)}|D_\theta(u+\eta;h,\sigma) - u|^2, \tag{131}$$

where $u\,|\,h = \mathcal{S}^{(k)}(h)$. The length scale hypothesis in the Main Text suggests that, for some length scale $\tilde{\epsilon} > 0$, and $\delta h \sim \mathcal{U}([-\tilde{\epsilon},\tilde{\epsilon}])$, the Lipschitz optimizer of $\mathcal{J}^{(k)}_{\mathrm{det}}$ satisfies $\Psi_{\theta^*}(h) \approx \mathbb{E}_{\delta h}\left[\mathcal{S}^{\Delta}(h+\delta h)\right]$, and that the constrained optimizer of $\mathcal{J}^{(k)}$ is approximately equal to the optimal denoiser for $\mathrm{Law}_{\delta h}\left[\mathcal{S}^{\Delta}(h+\delta h)\right]$. Our goal is to make this intuition rigorous, via asymptotic analysis as $k\to\infty$.

Deterministic Models Collapse to the Mean. We denote the constraind optimizer of the deterministic problem formulation by,

$$\Psi^{(k)} := \underset{\mathrm{Lip}(\Psi_\theta)\le L^*}{\operatorname{argmin}}\ \mathcal{J}^{(k)}_{\mathrm{det}}(\Psi_\theta). \tag{132}$$

We note that the underlying map $\mathcal{S}^{(k)}$ has length-scale $1/k$, whereas the smallest length scale of $\Psi^{(k)}$ is uniformly bounded due to the imposed Lipschitz bound. We now denote

$$\tilde{\Psi}^{(k)}(h) := \mathbb{E}_{\delta h}\left[\mathcal{S}^{(k)}(h+\delta h)\right], \quad \delta h \sim \mathcal{U}([-\tilde{\epsilon},\tilde{\epsilon}]). \tag{133}$$

We expect that $\Psi^{(k)}(h) \approx \tilde{\Psi}^{(k)}(h)$, under the assumptions of the Main Text. We next confirm this intuition, by showing that $\Psi^{(k)}$ and $\tilde{\Psi}^{(k)}$ are asymptotically equivalent, as $k\to\infty$.

Proposition SI 3.7. *With the definitions above, we have*

$$\lim_{k\to\infty}\mathbb{E}_{h\sim\bar{\mu}}|\Psi^{(k)}(h) - \tilde{\Psi}^{(k)}(h)|^2 = 0. \tag{134}$$

In fact, both $\Psi^{(k)}, \tilde{\Psi}^{(k)} \to 0$ *collapse to* 0 *in* $L^2(\bar{\mu})$ *as* $k\to\infty$.

The last proposition rigorously justifies the approximate identity $\Psi^{(k)}(h) \approx \mathbb{E}_{\delta h}\left[\mathcal{S}^{(k)}(h+\delta h)\right]$, and shows that the optimal constrained model $\Psi^{(k)}$ collapses to the mean (zero), in the limit $k\to\infty$.

Probabilistic Models Predict Uncertainty. We next consider the probabilistic problem formulation of conditional diffusion models. We denote by

$$D^{(k)}(u_\sigma;h,\sigma) := \underset{\mathrm{Lip}(D_\theta)\le L^*}{\operatorname{argmin}}\ \mathcal{J}^{(k)}(D_\theta), \tag{135}$$

the optimal constrained denoiser for $\mu^{(k)}(du\,|\,h) = \delta(u - \mathcal{S}^{(k)}(h))$. Given the discussion in the Main Text, we consider the conditional probability,

$$\nu^{(k)}(du\,|\,h) := \mathrm{Law}_{\delta h}\left[\mathcal{S}^{(k)}(h+\delta h)\right], \quad \delta h \sim \mathcal{U}([-\tilde{\epsilon},\tilde{\epsilon}]), \tag{136}$$

for which $\nu^{(k)}(du\,|\,h) \approx \mathrm{Law}_{\delta h}\left[\mathcal{S}^{(k)}(h+\delta h)\right]$, consistent with the discussion in the Main Text. We define

$$\tilde{D}^{(k)}(u_\sigma;h,\sigma) := \underset{D}{\operatorname{argmin}}\,\mathbb{E}_{h\sim\bar{\mu}}\mathbb{E}_{u\sim\nu^{(k)}(\,\cdot\,|\,h)}\mathbb{E}_{\eta\sim\mathcal{N}(0,\sigma^2)}\|D(u+\eta;h,\sigma) - u\|^2. \tag{137}$$

to be the optimal (unconstrained) denoiser of $\nu^{(k)}(du\,|\,h)$. Similar to toy model #1, we will show that $\nu^{(k)}(du\,|\,h)$ is asymptotically equivalent to $\mu(du\,|\,h)$, the conditional distribution arising from the joint uniform probability $\mu = \mathcal{U}(\mathbb{S}^1)\otimes\mathcal{U}([0,1])$ on $\mathbb{S}^1\times[0,1]$, with $\mathbb{S}^1\subset\mathbb{R}^2$ denoting the unit circle. The following result shows that the optimal constrained denoiser for $\mu^{(k)} = \delta(u - \mathcal{S}^{(k)}(h))$ is asymptotically equivalent to the optimal (unconstrained) denoiser for $\nu^{(k)}(du\,|\,h) = \mathrm{Law}_{\delta h}\left[\mathcal{S}^{(k)}(h+\delta h)\right]$, up to an error term that is exponentially small in the Lipschitz constant L^*:

Proposition SI 3.8. *Let* $\mu := \mathcal{U}(\mathbb{S}^1) \otimes \mathcal{U}([0,1])$. *With the definitions above, and for constant* L^* *sufficiently large, we have*

$$\limsup_{k\to\infty} \mathbb{E}_{(u,h)\sim\mu}\mathbb{E}_{\eta\sim\mathcal{N}(0,\sigma^2)} \left\| D^{(k)}(u+\eta;h,\sigma) - \tilde{D}^{(k)}(u+\eta;h,\sigma) \right\|^2 \le Ce^{-L^*/8C}, \tag{138}$$

with C *and* L^* *independent of* $\sigma > 0$.

We note that the appearance of the exponentially small additional term is due to the fact that the limiting denoiser is not uniformly Lipschitz continuous at the origin. However, since the origin is far from the data manifold, this mismatch only leads to a very small error contribution. Thus, we argue that also in this case, this analysis can justify the conclusion drawn from the length scale hypothesis of the Main Text, in the asymptotic regime $k \to \infty$.

SI 3.4 Mathematical Derivation

SI 3.4.1 Characterizing the Optimal Denoiser

The simple form of the forward process

$$u_\sigma = u + \eta, \quad \eta \sim \mathcal{N}(0, \sigma^2 I), \tag{139}$$

with η independent of u and $\bar{u}$, allows for explicit solution of the optimal denoiser, giving us insight into its mathematical properties. If $p(u \,|\, \bar{u})$ is the conditional distribution of u given the initial data $\bar{u}$, then the diffusion process defines $u_\sigma \,|\, (u, \bar{u})$ as a Gaussian random variable. The denoiser and its gradient are closely related to the posterior distribution of $u \,|\, (u_\sigma, \bar{u})$:

Lemma SI 3.9. *Assume that* $u \sim p(\,\cdot \,|\, \bar{u})$, *and* u_σ *is obtained by the forward process* (139). *Then the minimizer of* $D_{\mathrm{opt}} = \mathrm{argmin}_D\, \mathcal{J}(D, \sigma)$ *(cp.* (100)*) is given by*

$$D_{\mathrm{opt}}(u_\sigma; \bar{u}, \sigma) = \mathbb{E}[u \,|\, (u_\sigma, \bar{u})]. \tag{140}$$

The posterior distribution $u \,|\, (u_\sigma, \bar{u}) \sim q_\sigma(u; u_\sigma, \bar{u})$ *is given by the following mathematical expression,*

$$q_\sigma(u; w, \bar{u}) = \frac{1}{Z_\sigma} e^{-|u-w|^2/2\sigma^2} p(u \,|\, \bar{u}), \qquad Z_\sigma = \int e^{-|u-w|^2/2\sigma^2} p(u \,|\, \bar{u})\, du. \tag{141}$$

For completeness, we include a proof of Lemma SI 3.9 after the statement of Proposition SI 3.11, below. In words, the explicit formula in Lemma SI 3.9 tells us the following: Given that the noise process has ended up at location u_σ and given the additional information about $\bar{u}$, the denoiser considers the distribution of all possible origins $u \,|\, (u_\sigma, \bar{u})$ of the noise process over the distribution $p(u \,|\, \bar{u})$ and it singles out the most likely origin as the expected value over this distribution, i.e. the value $D_{\mathrm{opt}}(u_\sigma; \bar{u}, \sigma) = \mathbb{E}[u \,|\, (u_\sigma, \bar{u})]$.

We also remark the following corollary, which is immediate from Lemma SI 3.9:

Corollary SI 3.10. *If* $p(\,\cdot \,|\, \bar{u})$ *is supported on a bounded set* $\{|u| \le M\}$, *then* $|D_{\mathrm{opt}}(u_\sigma; \bar{u}, \sigma)| \le M$ *for all* u_σ *and* σ.

It is interesting to consider the limit $\sigma \to 0$ of the posterior (141). We fix w independently of σ, and consider the limiting behavior as $\sigma \to 0$, conditioned on the event $u_\sigma = w$. In this limit, the enumerator and denominator individually (formally) converge to $wp(w \,|\, \bar{u})$ and $p(w \,|\, \bar{u})$, respectively. Thus if we evaluate the optimal denoiser (140) at $u_\sigma = w$, we expect $D_{\mathrm{opt}}(w; \bar{u}, \sigma) \to w$ as $\sigma \to 0$. This is true,

if $p(w \mid \bar{u}) > 0$ and if e.g. $w \mapsto p(w \mid \bar{u})$ is continuous. However, in general, $p(u \mid \bar{u})$ may be 0 in some locations, or may even be supported on a lower-dimensional data manifold. In this case, we may have $p(w \mid \bar{u}) = 0$ at w, and the behavior of $\lim_{\sigma \to 0} D_{\text{opt}}(w; \bar{u}, \sigma)$ is unclear, at first sight. We next show that $D_{\text{opt}}(w; \bar{u}, \sigma)$ converges to the closest point in the support of $p(\,\cdot \mid \bar{u})$:

Proposition SI 3.11. *Fix $w \in \mathbb{R}^d$. Assume that there exists a unique closest point $w^* \in \mathbb{R}^d$ in the support of $p(u \mid \bar{u})$, i.e. $w^* = \operatorname{argmin}_{u \in \operatorname{supp}(p(\,\cdot \mid \bar{u}))} |w - u|$. Then,*

$$D_{\text{opt}}(w; \bar{u}, \sigma) = \mathbb{E}_{u \sim q_\sigma(\,\cdot\,; w, \bar{u})}[u] \to w^*, \qquad \text{as } \sigma \to 0, \tag{142}$$

i.e. in this limit the optimal denoiser $D_{\text{opt}}(w; \sigma = 0)$ evaluated at w, points to the closest point w^* in the support of $p(u \mid \bar{u})$.

We are often interested in comparing optimal denoisers between two probability measures μ and μ^Δ. We end this section by stating two results that allow us to relate the distance between optimal denoisers to the Wasserstein distance between μ and μ^Δ. A first result is provided by the previously stated Proposition SI 3.3. Calculations on specific examples (e.g. toy model #2) show that the optimal denoiser D_{opt} can be singular in the limit $\sigma \to 0$, in the sense that the local Lipschitz constant may blow up in certain locations. However, under suitable hypotheses on the data distribution, this only happens at a positive distance from the data distribution. The following Proposition generalizes Proposition SI 3.3 to allow for this possibility.

Proposition SI 3.12. *With the notation of Proposition SI 3.3, assume that there exists a set $A \subset \mathbb{R}^d$ such that the restriction $D_{\text{opt}}(\,\cdot\,;\,\cdot\,, \sigma)|_A$ is L^*-Lipschitz continuous for all $\sigma > 0$. Let D^Δ be defined as before. Then*

$$\mathcal{E} := \mathbb{E}_{(u,\bar{u}) \sim \mu} \mathbb{E}_{\eta \sim \mathcal{N}(0,\sigma^2)} \left\| D^\Delta(u + \eta; \bar{u}, \sigma) - D_{\text{opt}}(u + \eta; \bar{u}, \sigma) \right\|^2, \tag{143}$$

is upper bounded by

$$\mathcal{E} \le C \left\{ L^* W_1(\mu^\Delta, \mu) + \text{Prob}_{\mu_\sigma} \left[\mathbb{R}^d \setminus A \right] + \text{Prob}_{\mu_\sigma^\Delta} \left[\mathbb{R}^d \setminus A \right] \right\}, \tag{144}$$

where $C = C(M) > 0$ is a constant depending only on M.

SI 3.4.2 Proofs for Section SI 3.4.1.

Proof of Lemma SI 3.9. We recall that by definition, $D_{\text{opt}}(u; \bar{u}, \sigma)$ minimizes the functional

$$\mathcal{J}(D, \sigma) = \mathbb{E}_{u \sim p(\,\cdot \mid \bar{u})} \mathbb{E}_{\eta \sim \mathcal{N}(0,\sigma^2)} \|D(u + \eta; \bar{u}, \sigma) - u\|^2. \tag{145}$$

We now replace the expectation over $(u, \eta) \sim p(u \mid \bar{u}) \otimes \mathcal{N}(0, \sigma^2 I)$ by the expectation over $(u, u_\sigma) \mid \bar{u}$, where $u_\sigma \mid (u, \bar{u}) = u + \eta$ is obtained from the noise process and $u \mid \bar{u} \sim p(u \mid \bar{u})$. Then,

$$\mathcal{J}(D, \sigma) = \mathbb{E}_{(u,u_\sigma) \mid \bar{u}} \|D(u_\sigma; \bar{u}, \sigma) - u\|^2. \tag{146}$$

Next, we note that $D(u_\sigma; \bar{u}, \sigma)$ depends on u_σ, but not on u. This motivates splitting the expectation up as $\mathbb{E}_{(u,u_\sigma) \mid \bar{u}} = \mathbb{E}_{u_\sigma \sim p_\sigma(\,\cdot \mid \bar{u})} \mathbb{E}_{u \mid (u_\sigma, \bar{u})}$, to obtain,

$$\begin{aligned} \mathcal{J}(D, \sigma) &= \mathbb{E}_{u_\sigma \sim p_\sigma(\,\cdot \mid \bar{u})} \mathbb{E}_{u \mid (u_\sigma, \bar{u})} \|D(u_\sigma; \bar{u}, \sigma) - u\|^2 && (147) \\ &= \mathbb{E}_{u_\sigma \sim p_\sigma(\,\cdot \mid \bar{u})} \|D(u_\sigma; \bar{u}, \sigma) - \mathbb{E}_{u \mid (u_\sigma, \bar{u})}[u]\|^2 + \mathbb{E}_{u_\sigma \sim p_\sigma(\,\cdot \mid \bar{u})} \text{Var}_{u \mid (u_\sigma, \bar{u})}[u], && (148) \end{aligned}$$

where the last identity follows from a simple bias-variance decomposition. Since the last term is independent of $D(u_\sigma;\bar{u},\sigma)$, it follows that $\mathcal{J}(D,\sigma)$ is minimized by the choice $D(u_\sigma;\bar{u},\sigma) = \mathbb{E}_{u\,|\,(u_\sigma,\bar{u})}[u]$. The formula for the posterior follows by a straightforward calculation from Bayes formula,

$$p(u\,|\,u_\sigma,\bar{u}) \propto p(u_\sigma\,|\,u,\bar{u})p(u\,|\,\bar{u}). \tag{149}$$

□

Proof of Proposition SI 3.11. Let $w\in\mathbb{R}^d$ be given, and let w^* denote the closest point to w in the support of $p(\,\cdot\,|\,\bar{u})$. Let $q_\sigma(u;w,\bar{u})$ denote the posterior measure (141). Since $D_{\mathrm{opt}}(w;\bar{u},\sigma) = \int u q_\sigma(u;w,\bar{u})\,du$ and $w^* = \int w^* q_\sigma(u;w,\bar{u})\,du$, we have

$$|D_{\mathrm{opt}}(w;\bar{u},\sigma) - w^*| \le \int |u-w^*| q_\sigma(u;w,\bar{u})\,du \tag{150}$$

$$= \frac{\int |u-w^*|e^{-|u-w|^2/2\sigma^2}p(u\,|\,\bar{u})\,du}{\int e^{-|u-w|^2/2\sigma^2}p(u\,|\,\bar{u})\,du}. \tag{151}$$

Denote $r := |w-w^*|$, and let $\epsilon>0$ be given. Since w^* is the unique closest point to w, in the support of $p(\,\cdot\,|\,\bar{u})$, it follows that there exists $\delta>0$, such that $|u-w|<r+\delta$ implies that $|u-w^*|<\epsilon$.[1] Then,

$$\int |u-w^*|e^{-|u-w|^2/2\sigma^2}p(u\,|\,\bar{u})\,du = \int_{r\le|u-w|<r+\delta} |u-w^*|e^{-|u-w|^2/2\sigma^2}p(u\,|\,\bar{u})\,du \tag{152}$$

$$+ \int_{|u-w|\ge r+\delta} |u-w^*|e^{-|u-w|^2/2\sigma^2}p(u\,|\,\bar{u})\,du \tag{153}$$

$$\le \epsilon \int e^{-|u-w|^2/2\sigma^2}p(u\,|\,\bar{u})\,du + e^{-(r+\delta)^2/2\sigma^2}\mathbb{E}_{u\sim p(\,\cdot\,|\,\bar{u})}[|u-w^*|], \tag{154}$$

and

$$\int e^{-|u-w|^2/2\sigma^2}p(u\,|\,\bar{u})\,du \ge \int_{r\le|u-w|\le r+\delta/2} e^{-|u-w|^2/2\sigma^2}p(u\,|\,\bar{u})\,du \tag{155}$$

$$\ge e^{-(r+\delta/2)^2/2\sigma^2}\int_{r\le|u-w|\le r+\delta/2} p(u\,|\,\bar{u})\,du \tag{156}$$

$$\ge e^{-(r+\delta/2)^2/2\sigma^2}\int_{|u-w^*|\le\delta/2} p(u\,|\,\bar{u})\,du. \tag{157}$$

We note that $\int_{|u-w^*|\le\delta/2} p(u\,|\,\bar{u})\,du > 0$, since w^* belongs to the support of p. It follows that

$$\frac{\int |u-w^*|e^{-|u-w|^2/2\sigma^2}p(u\,|\,\bar{u})\,du}{\int e^{-|u-w|^2/2\sigma^2}p(u\,|\,\bar{u})\,du} \le \frac{\epsilon\int e^{-|u-w|^2/2\sigma^2}p(u\,|\,\bar{u})\,du}{\int e^{-|u-w|^2/2\sigma^2}p(u\,|\,\bar{u})\,du} \tag{158}$$

$$+ \frac{e^{-(r+\delta)^2/2\sigma^2}\mathbb{E}_{u\sim p(\,\cdot\,|\,\bar{u})}[|u-w^*|]}{\int e^{-|u-w|^2/2\sigma^2}p(u\,|\,\bar{u})\,du} \tag{159}$$

$$\le \epsilon + \frac{e^{-(r+\delta)^2/2\sigma^2}\mathbb{E}_{u\sim p(\,\cdot\,|\,\bar{u})}[|u-w^*|]}{e^{-(r+\delta/2)^2/2\sigma^2}\int_{|u-w^*|\le\delta/2} p(u\,|\,\bar{u})\,du}. \tag{160}$$

[1] If not, then there exists a sequence $u_n \in \mathrm{supp}(p(\,\cdot\,|\,\bar{u}))$, such that $|u_n - w| \le r + \frac{1}{n}$, while at the same time $|u_n - w^*| \ge \epsilon > 0$. This sequence must have a limit point u^*, necessarily belonging to the (closed) support of $p(\,\cdot\,|\,\bar{u})$, $|u^*-w| \le \limsup_n |u_n - w| = r$, and $|u^* - w^*| \ge \epsilon > 0$; thus, u^* is as close to w as w^*, contradicting the uniqueness of w^*.

Letting $\sigma \to 0$, the last term converges to 0 on account of the fact that

$$e^{-(r+\delta)^2/2\sigma^2} \ll e^{-(r+\delta/2)^2/2\sigma^2}. \tag{161}$$

Thus,

$$\limsup_{\sigma\to 0} \frac{\int |u-w^*| e^{-|u-w|^2/2\sigma^2} p(u \,|\, \bar{u})\, du}{\int e^{-|u-w|^2/2\sigma^2} p(u \,|\, \bar{u})\, du} \le \epsilon. \tag{162}$$

Since $\epsilon > 0$ was arbitrary, and the left-hand side is independent of ϵ, we conclude that

$$\lim_{\sigma\to 0} |D_{\rm opt}(w; \bar{u}, \sigma) - w^*| \le \lim_{\sigma\to 0} \frac{\int |u-w^*| e^{-|u-w|^2/2\sigma^2} p(u \,|\, \bar{u})\, du}{\int e^{-|u-w|^2/2\sigma^2} p(u \,|\, \bar{u})\, du} = 0. \tag{163}$$

This concludes our proof. □

The following lemma will be used in the proof of Proposition SI 3.3 and SI 3.12.

Lemma SI 3.13. *Let $\mathcal{A} \subset H$ be a convex set in a Hilbert space H. Let $\mathcal{J}(D) = \|D - F\|^2$ be a quadratic functional on $\mathcal{A}$, where $F \in H$. If $D_{\rm opt} \in \operatorname{argmin}_{D\in\mathcal{A}} \mathcal{J}(D)$, then*

$$\mathcal{J}(D) - \mathcal{J}(D_{\rm opt}) \ge \|D - D_{\rm opt}\|^2, \quad \forall\, D \in \mathcal{A}. \tag{164}$$

Proof of Lemma SI 3.13. Fix $D \in \mathcal{A}$ and let $D_\tau := (1-\tau)D_{\rm opt} + \tau D$. Since $\mathcal{A}$ is convex, we have $D_\tau \in \mathcal{A}$ for all $\tau \in [0,1]$. Since $D_{\rm opt}$ is a minimizer of $\mathcal{J}$, it follows that $\frac{d}{d\tau}|_{\tau=0}\mathcal{J}(D_\tau) \ge 0$. Evaluating the derivative, this implies,

$$\left.\frac{d}{d\tau}\right|_{\tau=0} \mathcal{J}(D_\tau) = 2\langle \dot{D}_\tau, D_{\rm opt} - F\rangle = 2\langle D - D_{\rm opt}, D_{\rm opt} - F\rangle \ge 0, \quad \forall\, D \in \mathcal{A}. \tag{165}$$

Given $D \in \mathcal{A}$, we now obtain

$$\begin{aligned}
\mathcal{J}(D) - \mathcal{J}(D_{\rm opt}) &= \|D - F\|^2 - \|D_{\rm opt} - F\|^2 && (166)\\
&= \langle (D-F) - (D_{\rm opt} - F), (D-F) + (D_{\rm opt} - F)\rangle && (167)\\
&= \langle D - D_{\rm opt}, D + D_{\rm opt} - 2F\rangle && (168)\\
&= \langle D - D_{\rm opt}, D - D_{\rm opt}\rangle + \underbrace{2\langle D - D_{\rm opt}, D_{\rm opt} - F\rangle}_{\ge 0} && (169)\\
&\ge \|D - D_{\rm opt}\|^2. && (170)
\end{aligned}$$

□

The proof of Proposition SI 3.3 will also make use of the following:

Lemma SI 3.14. *Assume $\phi : \mathcal{X} \to \mathcal{X}$ is a Lipschitz function, and $\mu, \nu \in \mathrm{Prob}(\mathcal{X})$ are probability measures. Then,*

$$\left|\mathbb{E}_{u\sim\mu}\|\phi(u)\|^2 - \mathbb{E}_{u\sim\nu}\|\phi(u)\|^2\right| \le 2\|\phi\|_{L^\infty} \mathrm{Lip}(\phi) W_1(\mu,\nu). \tag{171}$$

Proof of Lemma SI 3.14. Let $\pi \in \mathrm{Prob}(\mathcal{X}\times\mathcal{X})$ be an optimal W_1-coupling between μ and ν. Then,

$$\mathbb{E}_{u\sim\mu}\|\phi(u)\|^2 - \mathbb{E}_{u\sim\nu}\|\phi(u)\|^2 = \int \|\phi(u)\|^2\, d\mu(u) - \int \|\phi(v)\|^2\, d\nu(v) \tag{172}$$

$$= \int \left\{ \|\phi(u)\|^2 - \|\phi(v)\|^2 \right\} \, d\pi(u,v) \tag{173}$$

$$= \int (\|\phi(u)\| + \|\phi(v)\|) \left\{ \|\phi(u)\| - \|\phi(v)\| \right\} \, d\pi(u,v) \tag{174}$$

$$\leq \int 2\|\phi\|_{L^\infty} \left\{ \|\phi(u) - \phi(v)\| \right\} \, d\pi(u,v) \tag{175}$$

$$\leq 2\|\phi\|_{L^\infty} \mathrm{Lip}(\phi) \int \left\{ \|u - v\| \right\} \, d\pi(u,v) \tag{176}$$

$$= 2\|\phi\|_{L^\infty} \mathrm{Lip}(\phi) W_1(\mu,\nu). \tag{177}$$

This proves the claimed bound if $\mathbb{E}_{u\sim\mu}\|\phi(u)\|^2 \geq \mathbb{E}_{u\sim\nu}\|\phi(u)\|^2$. For the reverse case, we can simply switch μ and ν in the above estimates. The claimed bound thus follows. $\square$

SI 3.4.3 Proof of Proposition SI 3.3

We now come to the proof of Proposition SI 3.3.

Proof of Proposition SI 3.3. The idea is to compare the optimal constrained denoiser

$$D^\Delta = \underset{\mathrm{Lip}(D_\theta)\leq L^*}{\mathrm{argmin}} \, \mathcal{J}^\Delta(D_\theta) \tag{178}$$

with the unconstrained denoiser $D_{\mathrm{opt}} = \mathrm{argmin}_D \, \mathcal{J}(D)$, for

$$\mathcal{J}(D) := \mathbb{E}_{(u,\bar{u})\sim\mu} \mathbb{E}_{\eta\sim\mathcal{N}(0,\sigma^2)} \|D(u+\eta;\bar{u},\sigma) - u\|^2. \tag{179}$$

By the assumptions of this proposition, D_{opt} is L^*-Lipschitz continuous. We note that, for any $\sigma > 0$,

$$\begin{aligned} \mathcal{J}(D^\Delta,\sigma) &\leq \mathcal{J}^\Delta(D^\Delta,\sigma) + \left|\mathcal{J}(D^\Delta,\sigma) - \mathcal{J}^\Delta(D^\Delta,\sigma)\right| \\ &\leq \mathcal{J}^\Delta(D_{\mathrm{opt}},\sigma) + \left|\mathcal{J}(D^\Delta,\sigma) - \mathcal{J}^\Delta(D^\Delta,\sigma)\right| \\ &\leq \mathcal{J}(D_{\mathrm{opt}},\sigma) + 2 \max_{D=D_{\mathrm{opt}},D^\Delta} \left|\mathcal{J}(D,\sigma) - \mathcal{J}^\Delta(D,\sigma)\right|. \end{aligned} \tag{180}$$

To prove the claim, it thus suffices to show that there exists $C > 0$, independent of Δ, L^* and σ, such that

$$2 \max_{D=D_{\mathrm{opt}},D^\Delta} \left|\mathcal{J}(D,\sigma) - \mathcal{J}^\Delta(D,\sigma)\right| \leq C L^* W_1(\mu^\Delta,\mu). \tag{181}$$

To prove such an estimate, we first recall that

$$\mathcal{J}(D,\sigma) = \mathbb{E}_{(u,\bar{u})\sim\mu} \mathbb{E}_{\eta\sim\mathcal{N}(0,\sigma^2)} \|D(u+\eta;\bar{u},\sigma) - u\|^2, \tag{182}$$

and similarly for $\mathcal{J}^\Delta$, except that $(u,\bar{u}) \sim \mu$ is replaced by $(u,\bar{u}) \sim \mu^\Delta$. Let us now momentarily fix $\eta \in \mathcal{X}$. Given a choice of either $D = D^\Delta$ or $D = D_{\mathrm{opt}}$, we introduce,

$$\phi_\eta(u,\bar{u}) := D(u+\eta;\bar{u},\sigma) - u. \tag{183}$$

The following estimate will hold for either choice of $D = D^\Delta, D_{\mathrm{opt}}$. By assumption on $D^\Delta, D_{\mathrm{opt}}$ being L^*-Lipschitz, it follows that $\mathrm{Lip}(\phi_\eta) \leq \mathrm{Lip}(D) + 1 \leq L^* + 1$. By Lemma SI 3.14, it therefore follows that

$$\begin{aligned} \left|\mathcal{J}(D,\sigma) - \mathcal{J}^\Delta(D,\sigma)\right| &= \left|\mathbb{E}_{(u,\bar{u})\sim\mu} \mathbb{E}_{\eta\sim\mathcal{N}(0,\sigma^2)} \|\phi_\eta(u,\bar{u})\|^2 - \mathbb{E}_{(u,\bar{u})\sim\mu^\Delta} \mathbb{E}_{\eta\sim\mathcal{N}(0,\sigma^2)} \|\phi_\eta(u,\bar{u})\|^2\right| \\ &\leq \mathbb{E}_{\eta\sim\mathcal{N}(0,\sigma^2)} \left|\mathbb{E}_{(u,\bar{u})\sim\mu} \|\phi_\eta(u,\bar{u})\|^2 - \mathbb{E}_{(u,\bar{u})\sim\mu^\Delta} \|\phi_\eta(u,\bar{u})\|^2\right| \end{aligned}$$

$$\leq 2\mathbb{E}_{\eta\sim\mathcal{N}(0,\sigma^2)}\left[\|\phi_\eta\|_{L^\infty}\right]\mathrm{Lip}(\phi_\eta)W_1(\mu,\mu^\Delta)$$
$$\leq 2(L^*+1)\,\mathbb{E}_{\eta\sim\mathcal{N}(0,\sigma^2)}\left[\|\phi_\eta\|_{L^\infty}\right]\,W_1(\mu,\mu^\Delta). \tag{184}$$

Comparing with (106), we finally need to show that $\mathbb{E}_{\eta\sim\mathcal{N}(0,\sigma^2)}\left[\|\phi_\eta\|_{L^\infty}\right] \leq B$ is bounded by a constant B independent of Δ. Then (106) holds with constant $2(L^*+1)B \leq 4L^*B =: C$, where we used $L^* \geq 1$ to get a simpler bound.

We note that by the explicit formula for $D_{\mathrm{opt}}(u_\sigma;\bar{u},\sigma) = \mathbb{E}[u\,|\,(u_\sigma,\bar{u})]$ and the assumption that μ is concentrated on $B_M = \{\|u\| \leq M\}$, it is immediate that $\|D_{\mathrm{opt}}(u_\sigma;\bar{u},\sigma)\| \leq M$ for any choice of u_σ. In particular, this implies that for $D = D_{\mathrm{opt}}$, we have

$$\mathbb{E}_{\eta\sim\mathcal{N}(0,\sigma^2)}\left[\|\phi_\eta\|_{L^\infty}\right] = \mathbb{E}_{\eta\sim\mathcal{N}(0,\sigma^2)}\left[\|D_{\mathrm{opt}}(u+\eta;\bar{u},\sigma)-u\|_{L^\infty}\right] \leq 2M. \tag{185}$$

For D^Δ, we can also show that $\|D^\Delta(u+\eta;\bar{u},\sigma)\| \leq M$. To see this, let us introduce the M-truncated mapping,

$$D^\Delta_M(u_\sigma;\bar{u},\sigma) := \begin{cases} D^\Delta(u_\sigma;\bar{u},\sigma), & \text{if } \|D^\Delta(u_\sigma;\bar{u},\sigma)\| \leq M, \\ \frac{M\,D^\Delta(u_\sigma;\bar{u},\sigma)}{\|D^\Delta(u_\sigma;\bar{u},\sigma)\|} & \text{if } \|D^\Delta(u_\sigma;\bar{u},\sigma)\| > M. \end{cases} \tag{186}$$

Then D^Δ_M is still L^*-Lipschitz. However, it is easy to see that for any $\|u\| \leq M$ and $\bar{u},\eta \in \mathcal{X}$, we have

$$\|D^\Delta_M(u+\eta;\bar{u},\sigma)-u\| \leq \|D^\Delta(u+\eta;\bar{u},\sigma)-u\|. \tag{187}$$

Upon taking expectations with respect to $u,\bar{u},\eta$, this in turn implies that $\mathcal{J}^\Delta(D^\Delta_M,\sigma) \leq \mathcal{J}^\Delta(D^\Delta,\sigma)$. However, D^Δ is by assumption the minimizer of the functional $\mathcal{J}^\Delta$, over the set of L^*-Lipschitz mappings. By the uniqueness of a minimizer over this (convex) set, and since D^Δ_M is still L^*-Lipschitz, it follows that $D^\Delta = D^\Delta_M$, i.e. D^Δ is uniformly bounded by M. Thus, also in this case, we have for $D = D^\Delta$:

$$\mathbb{E}_{\eta\sim\mathcal{N}(0,\sigma^2)}\left[\|\phi_\eta\|_{L^\infty}\right] = \mathbb{E}_{\eta\sim\mathcal{N}(0,\sigma^2)}\left[\|D^\Delta(u+\eta;\bar{u},\sigma)-u\|_{L^\infty}\right] \leq 2M. \tag{188}$$

Combining (188), (185), (184) and (180), we conclude that

$$\mathcal{J}(D^\Delta,\sigma) \leq \mathcal{J}(D_{\mathrm{opt}},\sigma) + CL^*W_1(\mu^\Delta,\mu), \tag{189}$$

for $C = 8M$. Since D_{opt} is the optimizer of the quadratic functional $\mathcal{J}$, it follows from Lemma SI 3.13 that

$$\mathbb{E}_{(u,\bar{u})\sim\mu}\mathbb{E}_{\eta\sim\mathcal{N}(0,\sigma^2)}\|D^\Delta(u+\eta;\bar{u},\sigma) - D_{\mathrm{opt}}(u+\eta;\bar{u},\sigma)\|^2 \leq \mathcal{J}(D^\Delta) - \mathcal{J}(D_{\mathrm{opt}}), \tag{190}$$

and hence

$$\mathbb{E}_{(u,\bar{u})\sim\mu}\mathbb{E}_{\eta\sim\mathcal{N}(0,\sigma^2)}\|D^\Delta(u+\eta;\bar{u},\sigma) - D_{\mathrm{opt}}(u+\eta;\bar{u},\sigma)\|^2 \leq CL^*W_1(\mu^\Delta,\mu), \tag{191}$$

by the previous bound. This completes the proof of Proposition SI 3.3. □

SI 3.4.4 Proof of Proposition SI 3.12

Proof of Proposition SI 3.12. We note that

$$\mathcal{J}(D^\Delta) \leq \mathcal{J}^\Delta(D^\Delta) + \left|\mathcal{J}(D^\Delta) - \mathcal{J}^\Delta(D^\Delta)\right| \tag{192}$$
$$\leq \mathcal{J}^\Delta(D_{\mathrm{opt}}) + \left|\mathcal{J}(D^\Delta) - \mathcal{J}^\Delta(D^\Delta)\right| \tag{193}$$

$$\leq \mathcal{J}(D_{\text{opt}}) + \left|\mathcal{J}(D^\Delta) - \mathcal{J}^\Delta(D^\Delta)\right| + \left|\mathcal{J}(D_{\text{opt}}) - \mathcal{J}^\Delta(D_{\text{opt}})\right|. \tag{194}$$

By assumption D^Δ is L^*-Lipschitz. Thus, (184) and (185) in the proof of Proposition SI 3.3 imply that

$$\left|\mathcal{J}(D^\Delta) - \mathcal{J}^\Delta(D^\Delta)\right| \leq 4M(L^* + 1)W_1(\mu, \mu^\Delta). \tag{195}$$

By assumption, D_{opt} is L^*-Lipschitz when restricted to $A \subset \mathbb{R}^d$. By the Kirszbraun theorem, there therefore exists $D : \mathbb{R}^d \to \mathbb{R}^d$ with $\text{Lip}(D) = \text{Lip}(D_{\text{opt}}|_A)$, $\|D\|_{L^\infty} = \|D_{\text{opt}}\|_{L^\infty}$ and $D|_A \equiv D_{\text{opt}}|_A$. Given such a choice of D, we now bound

$$|\mathcal{J}(D_{\text{opt}}) - \mathcal{J}^\Delta(D_{\text{opt}})| \leq |\mathcal{J}(D_{\text{opt}}) - \mathcal{J}(D)| + |\mathcal{J}(D) - \mathcal{J}^\Delta(D)| + |\mathcal{J}^\Delta(D) - \mathcal{J}^\Delta(D_{\text{opt}})|. \tag{196}$$

Denote $A^c = \mathbb{R}^d \setminus A$. The first term can be bounded by observing that

$$\begin{aligned}
|\mathcal{J}(D_{\text{opt}}) - \mathcal{J}(D)| &= \left|\mathbb{E}_u\|D_{\text{opt}}(u_\sigma; \bar{u}, \sigma) - u\|^2 - \mathbb{E}_u\|D(u_\sigma; \bar{u}, \sigma) - u\|^2\right| &(197)\\
&= \left|\mathbb{E}_u\left[1_{A^c}(u_\sigma, \bar{u})\|D_{\text{opt}}(u_\sigma; \bar{u}, \sigma) - u\|^2\right] - \mathbb{E}_u\left[1_{A^c}(u_\sigma, \bar{u})\|D(u_\sigma; \bar{u}, \sigma) - u\|^2\right]\right| &(198)\\
&\leq (\|D_{\text{opt}}\|_{L^\infty} + 1)^2 \text{Prob}_{\mu_\sigma}\left[A^c\right]. &(199)
\end{aligned}$$

Since D_{opt} is the optimal denoiser for μ, it follows that $\|D_{\text{opt}}\|_{L^\infty} \leq M$, from Corollary SI 3.10. Thus,

$$|\mathcal{J}(D_{\text{opt}}) - \mathcal{J}(D)| \leq (M + 1)^2 \text{Prob}_{\mu_\sigma}\left[A^c\right]. \tag{200}$$

Similarly, we can show that

$$\left|\mathcal{J}^\Delta(D_{\text{opt}}) - \mathcal{J}^\Delta(D)\right| \leq (M + 1)^2 \text{Prob}_{\mu_\sigma^\Delta}\left[A^c\right]. \tag{201}$$

Finally, (184) and (185) in the proof of Proposition SI 3.3 imply that

$$\left|\mathcal{J}(D) - \mathcal{J}^\Delta(D)\right| \leq 4M(L^* + 1)W_1(\mu, \mu^\Delta). \tag{202}$$

Combining these estimates, it follows that

$$\mathcal{J}(D^\Delta) - \mathcal{J}(D_{\text{opt}}) \leq C\left\{L^* W_1(\mu, \mu^\Delta) + \text{Prob}_{\mu_\sigma}[A^c] + \text{Prob}_{\mu_\sigma^\Delta}[A^c]\right\}, \tag{203}$$

where $C = C(M) > 0$ depends only on M. D_{opt} is the unconstrained optimizer of the quadratic functional $\mathcal{J}$. Thus, by Lemma SI 3.13, it follows that

$$\mathbb{E}_{(u,\bar{u})\sim\mu}\mathbb{E}_{\eta\sim\mathcal{N}(0,\sigma^2)}\|D^\Delta(u + \eta; \bar{u}, \sigma) - D_{\text{opt}}(u + \eta; \bar{u}, \sigma)\|^2 \leq \mathcal{J}(D^\Delta) - \mathcal{J}(D_{\text{opt}}). \tag{204}$$

The claimed bound on the error thus follows. □

SI 3.4.5 Proofs for Section SI 3.2

We here give the detailed proof of Proposition SI 3.5 and Proposition SI 3.6.

Deterministic Setting. We start with the proof of Proposition SI 3.5.

Proof of Proposition SI 3.5. We first note that $\tilde{\Psi}^\Delta(\bar{u}) = m(\bar{u}) + \mathbb{E}_{\delta\bar{u}}\left[s_N(\bar{u} + \delta\bar{u})\right]$ converges to $m(\bar{u})$ as $\Delta \to 0$. This follows easily from well-known facts about weak limits, which imply in particular that the rapidly oscillating function $\delta\bar{u} \mapsto s_N(\bar{u} + \delta\bar{u})$ satisfies,

$$\mathbb{E}_{\delta\bar{u}}\left[s_N(\bar{u} + \delta\bar{u})\right] = \frac{1}{2\tilde{\epsilon}}\int_{-\tilde{\epsilon}}^{\tilde{\epsilon}} \Lambda(N(\bar{u} + v))\, dv \to \int_0^1 \Lambda(\xi)\, d\xi = 0, \tag{205}$$

where the last equality follows from our definition of Λ. Since this convergence holds pointwise for any fixed $\bar{u}$, by dominated convergence, it follows also in $L^2([0,1])$. Thus, we conclude that $\tilde{\Psi}^\Delta(\bar{u})$ and $m(\bar{u})$ are asymptotically equivalent, in the sense that

$$\lim_{\Delta\to 0} \mathbb{E}_{\bar{u}\sim\bar{\mu}} \|\tilde{\Psi}^\Delta(\bar{u}) - m(\bar{u})\|^2 = 0. \tag{206}$$

It will thus suffice to show,

$$\lim_{\Delta\to 0} \mathbb{E}_{\bar{u}\sim\bar{\mu}} \|\Psi^\Delta(\bar{u}) - m(\bar{u})\|^2 = 0. \tag{207}$$

To this end, we first write

$$\mathbb{E}_{\bar{u}\sim\bar{\mu}} \|\Psi^\Delta(\bar{u}) - m(\bar{u})\|^2 = \mathbb{E}_{\bar{u}\sim\bar{\mu}} \|\Psi^\Delta(\bar{u}) - \mathcal{S}^\Delta(\bar{u})\|^2 \tag{208}$$

$$- 2\mathbb{E}_{\bar{u}\sim\bar{\mu}} \langle \Psi^\Delta(\bar{u}) - m(\bar{u}), s_N(\bar{u})\rangle - \mathbb{E}_{\bar{u}\sim\bar{\mu}} \|s_N(\bar{u})\|^2. \tag{209}$$

Since Ψ^Δ minimizes the first term over all L^*-Lipschitz functions, and since the mean function is L^*-Lipschitz by assumption, we obtain,

$$\mathbb{E}_{\bar{u}\sim\bar{\mu}} \|\Psi^\Delta(\bar{u}) - m(\bar{u})\|^2 \le \mathbb{E}_{\bar{u}\sim\bar{\mu}} \|m(\bar{u}) - \mathcal{S}^\Delta(\bar{u})\|^2 \tag{210}$$

$$- 2\mathbb{E}_{\bar{u}\sim\bar{\mu}} \langle \Psi^\Delta(\bar{u}) - m(\bar{u}), s_N(\bar{u})\rangle - \mathbb{E}_{\bar{u}\sim\bar{\mu}} \|s_N(\bar{u})\|^2 \tag{211}$$

$$= -2\mathbb{E}_{\bar{u}\sim\bar{\mu}} \langle \Psi^\Delta(\bar{u}) - m(\bar{u}), s_N(\bar{u})\rangle. \tag{212}$$

It is a textbook exercise in analysis to show that $\Psi(\bar{u}) \coloneqq \Psi^\Delta(\bar{u}) - m(\bar{u})$ is $2L^*$-Lipschitz continuous, and that there exists a constant $C > 0$, such that

$$\sup_{\mathrm{Lip}(\Psi)\le 2L^*} \mathbb{E}_{\bar{u}\sim\bar{\mu}} \langle \Psi(\bar{u}), s_N(\bar{u})\rangle \le \frac{C}{N} = C\Delta. \tag{213}$$

Thus, we conclude that $\lim_{\Delta\to 0} \mathbb{E}_{\bar{u}\sim\bar{\mu}} \|\Psi^\Delta(\bar{u}) - m(\bar{u})\|^2 = 0$, as claimed. □

Probabilistic Setting. We now consider the probabilistic setting of conditional diffusion models. Our asymptotic results will hold for any $\tilde{\epsilon} > 0$. We thus assume $\tilde{\epsilon}$ to be fixed (arbitrarily). We recall that,

$$\nu^\Delta(du \,|\, \bar{u}) \coloneqq \mathrm{Law}_{\delta\bar{u}} \left[m(\bar{u}) + s_N(\bar{u} + \delta\bar{u})\right], \quad \delta\bar{u} \sim \mathcal{U}([-\tilde{\epsilon}, \tilde{\epsilon}]), \tag{214}$$

and $\tilde{D}^\Delta(u_\sigma; \bar{u}, \sigma)$ denotes the optimal unconstrained conditional denoiser for ν^Δ. Since the derivation of Proposition SI 3.6 is more involved, we will first give an overview of the essential ingredients, and leave their proof for later paragraphs.

Our first result shows that $\nu^\Delta \approx \mathcal{U}([m(\bar{u}) - 1, m(\bar{u}) + 1])$ is approximately equivalent to a uniform distribution, in a suitable sense:

Lemma SI 3.15. *Let $\mu(du \,|\, \bar{u}) = \mathcal{U}([m(\bar{u}) - 1, m(\bar{u}) + 1])$ be a uniform measure. There exists a constant $C > 0$, independent of Δ, such that*

$$(1 - C\Delta)\mu(du \,|\, \bar{u}) \le \nu^\Delta(du \,|\, \bar{u}) \le (1 + C\Delta)\mu(du \,|\, \bar{u}). \tag{215}$$

The result of the last lemma is important because it allows us to identify the limit $D_{\mathrm{opt}} = \lim_{\Delta\to 0} \tilde{D}^\Delta$, owing to the following result.

Lemma SI 3.16. *Let μ, ν be probability measures on $\mathbb{R}^d$, supported on a bounded set $\{|u| \le M\}$ and suppose that for some $\epsilon \in (0, 1)$, we have*

$$(1 - \epsilon)\mu \le \nu \le (1 + \epsilon)\mu. \tag{216}$$

Let $D^\mu(u_\sigma; \sigma), D^\nu(u_\sigma; \sigma)$ denote the corresponding denoisers. Then

$$\|D^\mu(\,\cdot\,; \sigma) - D^\nu(\,\cdot\,; \sigma)\|_{L^\infty(\mathbb{R}^d)} \le 2M\epsilon. \tag{217}$$

Given the results of Lemma SI 3.15 and Lemma SI 3.16, the following corollary is now immediate:

Corollary SI 3.17. *Let μ be the uniform measure on $\mathcal{I} \coloneqq \{(u,\bar{u}) \in \mathbb{R}\times[0,1] \,|\, u \in [m(\bar{u})-1, m(\bar{u})+1]\}$. Let D_{opt} denote the optimal (unconstrained) conditional denoiser for μ. Then we have,*

$$\mathbb{E}_{(u,\bar{u})\sim\mu}\mathbb{E}_{\eta\sim\mathcal{N}(0,\sigma^2)} \left\| \tilde{D}^{\Delta}(u+\eta;\bar{u},\sigma) - D_{\mathrm{opt}}(u+\eta;\bar{u},\sigma) \right\|^2 \le C\Delta. \tag{218}$$

Due to the simplicity of μ, the optimal denoiser D_{opt} can be characterized quite explicitly, as shown next:

Lemma SI 3.18. *Let $D_{\mathrm{opt}}(u;\bar{u},\sigma)$ denote the optimal denoiser for the uniform measure μ on $\mathcal{I}$ introduced above. Then D_{opt} is L^*-Lipschitz continuous, uniformly as $\sigma \to 0$, for some constant $L^* > 0$, and*

$$\lim_{\sigma\to 0} D_{\mathrm{opt}}(u;\bar{u},\sigma) = g(u - m(\bar{u})), \tag{219}$$

where

$$g(u) = \begin{cases} -1, & \text{if } u < -1, \\ u, & \text{if } -1 \le u \le +1, \\ +1, & \text{if } u > +1. \end{cases} \tag{220}$$

The proof of Lemma SI 3.18 is given below. Given the above results, we can now finally come to the proof of Proposition SI 3.6.

Proof of Proposition SI 3.6. We recall that our goal is to show that

$$\lim_{\Delta\to 0} \mathbb{E}_{(u,\bar{u})\sim\mu}\mathbb{E}_{\eta\sim\mathcal{N}(0,\sigma^2)} \left\| D^{\Delta}(u+\eta;\bar{u},\sigma) - \tilde{D}^{\Delta}(u+\eta;\bar{u},\sigma) \right\|^2 = 0. \tag{221}$$

Corollary SI 3.17 shows that $\tilde{D}^{\Delta} \to D_{\mathrm{opt}}$ with D_{opt} the conditional diffusion model for μ. It will thus be enough to show that

$$\lim_{\Delta\to 0} \mathbb{E}_{(u,\bar{u})\sim\mu}\mathbb{E}_{\eta\sim\mathcal{N}(0,\sigma^2)} \left\| D^{\Delta}(u+\eta;\bar{u},\sigma) - D_{\mathrm{opt}}(u+\eta;\bar{u},\sigma) \right\|^2 = 0. \tag{222}$$

Since D_{opt} is L^*-Lipschitz continuous by Lemma SI 3.18, it follows from Proposition SI 3.3 that

$$\mathbb{E}_{(u,\bar{u})\sim\mu}\mathbb{E}_{\eta\sim\mathcal{N}(0,\sigma^2)} \left\| D^{\Delta}(u+\eta;\bar{u},\sigma) - D_{\mathrm{opt}}(u+\eta;\bar{u},\sigma) \right\|^2 \le CL^* W_1(\mu,\mu^{\Delta}). \tag{223}$$

Lemma SI 3.19 below shows that $W_1(\mu^{\Delta},\mu) \to 0$, completing the proof. ☐

The following lemma identifies a robust statistical limit for this toy problem.

Lemma SI 3.19. *Let $\mu \in \mathrm{Prob}(\mathbb{R}\times[0,1])$ be given by the uniform measure on*

$$\mathcal{I}(m) \coloneqq \{(u,\bar{u}) \in \mathbb{R}\times[0,1] \,|\, u \in [m(\bar{u})-1, m(\bar{u})+1]\}. \tag{224}$$

Then,

$$W_1(\mu^{\Delta},\mu) = O(\Delta) \to 0, \qquad \text{as } \Delta \to 0. \tag{225}$$

Interestingly, Lemma SI 3.19 shows that, even though $\mathcal{S}^{\Delta}$ is highly oscillatory and cannot possess a limiting function $\mathcal{S}^{\Delta} \not\to \mathcal{S}$, the associated probability measure μ^{Δ} nevertheless converges in a statistical sense to a well-defined limit μ. Disintegration of this limit μ yields,

$$\mu(du, d\bar{u}) = p(u \,|\, \bar{u})\, du\, d\bar{u}, \tag{226}$$

where $p(u \,|\, \bar{u}) = \mathcal{U}[m(\bar{u})-1, m(\bar{u})+1]$ is the uniform distribution on an interval centered around $m(\bar{u})$. In particular, *the limit is not a Dirac δ-distribution.*

Proofs of Lemma SI 3.15, Lemma SI 3.16, Lemma SI 3.18 and Lemma SI 3.19 In the following, we detail the proofs of Lemma SI 3.15, Lemma SI 3.16, Lemma SI 3.18 and Lemma SI 3.19.

Proof of Lemma SI 3.15. Note that ν^Δ, by definition, is the push-forward measure of $\mathcal{U}([-\tilde{\epsilon},\tilde{\epsilon}])$ under the mapping $f_N : [-\tilde{\epsilon},\tilde{\epsilon}] \to \mathbb{R}$, $f_N(\xi) = m(\bar{u}) + \Lambda(N\bar{u} + N\xi)$. Since Λ is a hat function mapping onto the range $[-1,1]$, and since its derivative $|\Lambda'| = 1$ has magnitude 1 almost everywhere, it follows from the change of variables formula for push-forward measures that $\nu^\Delta(du\,|\,\bar{u})$ is probability measure on $[m(\bar{u})-1, m(\bar{u})+1]$ with a probability density $q(u)$ whose value at a given u is proportional to the number of points in the pre-image of u, i.e.

$$q(u) = c\,\#\{\xi \in [-\tilde{\epsilon},\tilde{\epsilon}] \,|\, f_N(\xi) = u\}, \tag{227}$$

holds for almost every $u \in [m(\bar{u})-1, m(\bar{u})+1]$, where c is a normalization constant. Since f_N is $1/N$-periodic, there are $\lfloor 2\tilde{\epsilon}N \rfloor$ completed periods over the interval $[-\tilde{\epsilon},\tilde{\epsilon}]$. On each completed period, the equation $f_N(\xi) = u$ has two solutions for almost every $u \in [m(\bar{u})-1, m(\bar{u})+1]$. Thus, we have

$$2\lfloor 2\tilde{\epsilon}N \rfloor \le \#\{\xi \in [-\tilde{\epsilon},\tilde{\epsilon}] \,|\, f_N(\xi) = u\} \le 2\lfloor 2\tilde{\epsilon}N \rfloor + 1, \tag{228}$$

implying that

$$2c\lfloor 2\tilde{\epsilon}N \rfloor \le q(u) \le 2c\lfloor 2\tilde{\epsilon}N \rfloor + c, \quad \forall\, u \in [m(\bar{u})-1, m(\bar{u})+1]. \tag{229}$$

Integration over u, and using the fact that $\int q(u)\,du = 1$, then implies that $c \sim 1/4\lfloor \tilde{\epsilon}N \rfloor$, and hence,

$$q(u) = \frac{1}{2} + O\left(\frac{1}{\tilde{\epsilon}N}\right) = \frac{1}{2} + O(\tilde{\epsilon}^{-1}\Delta), \tag{230}$$

with an absolute implied constant in the big-O notation. Since $q_0(u) \equiv \frac{1}{2}$ for the considered values of u is the density of the uniform distribution $\mu = \mathcal{U}([m(\bar{u})-1, m(\bar{u})+1])$, we conclude that there exists a constant $C = C(\tilde{\epsilon}) > 0$, proportional to $1/\tilde{\epsilon}$, such that

$$(1 - C\Delta)\mu \le \nu^\Delta \le (1 + C\Delta)\mu. \tag{231}$$

□

Proof of Lemma SI 3.16. We recall that

$$D^\mu(w;\sigma) = \frac{\int u e^{-|w-u|^2/2\sigma^2}\mu(du)}{\int e^{-|w-u|^2/2\sigma^2}\mu(du)}, \quad D^\nu(w;\sigma) = \frac{\int u e^{-|w-u|^2/2\sigma^2}\nu(du)}{\int e^{-|w-u|^2/2\sigma^2}\nu(du)}. \tag{232}$$

We now compute

$$\begin{aligned} D^\mu(w;\sigma) - D^\nu(w;\sigma) &= \frac{\int u e^{-|w-u|^2/2\sigma^2}[\mu(du) - \nu(du)]}{\int e^{-|w-u|^2/2\sigma^2}\mu(du)} \\ &\quad + \frac{\int u e^{-|w-u|^2/2\sigma^2}\nu(du)}{\int e^{-|w-u|^2/2\sigma^2}\nu(du)} \left(\frac{\int e^{-|w-u|^2/2\sigma^2}\nu(du)}{\int e^{-|w-u|^2/2\sigma^2}\mu(du)} - 1 \right) \qquad (233) \\ &= \frac{\int u e^{-|w-u|^2/2\sigma^2}\left[1 - \frac{d\nu}{d\mu}\right]\mu(du)}{\int e^{-|w-u|^2/2\sigma^2}\mu(du)} \\ &\quad + D^\nu(w;\sigma)\left(\frac{\int e^{-|w-u|^2/2\sigma^2}\nu(du)}{\int e^{-|w-u|^2/2\sigma^2}\mu(du)} - 1 \right). \qquad (234) \end{aligned}$$

By assumption, we have $(1-\epsilon)\mu \le \nu \le (1+\epsilon)\mu$. This implies that the Radon-Nikodym derivative $d\nu/d\mu$ satisfies $-\epsilon \le \frac{d\nu}{d\mu} - 1 \le \epsilon$, implying that

$$\left\| 1 - \frac{d\nu}{d\mu} \right\|_{L^\infty(\mu)} \le \epsilon. \tag{235}$$

Furthermore, taking convolution with $e^{-|u|^2/2\sigma^2}$, the inequalities between μ and ν also imply,

$$(1-\epsilon)\int e^{-|w-u|^2/2\sigma^2}\mu(du) \le \int e^{-|w-u|^2/2\sigma^2}\nu(du) \le (1+\epsilon)\int e^{-|w-u|^2/2\sigma^2}\mu(du), \tag{236}$$

and hence,

$$\left| \frac{\int e^{-|w-u|^2/2\sigma^2}\nu(du)}{\int e^{-|w-u|^2/2\sigma^2}\mu(du)} - 1 \right| \le \epsilon. \tag{237}$$

Thus, we conclude that

$$\left| \frac{\int u e^{-|w-u|^2/2\sigma^2}\left[1 - \frac{d\nu}{d\mu}\right]\mu(du)}{\int e^{-|w-u|^2/2\sigma^2}\mu(du)} \right| \le \frac{\int |u| e^{-|w-u|^2/2\sigma^2}\mu(du)}{\int e^{-|w-u|^2/2\sigma^2}\mu(du)} \left\| 1 - \frac{d\nu}{d\mu} \right\|_{L^\infty(\mu)} \le M\epsilon, \tag{238}$$

and

$$\left| D^\nu(w;\sigma)\left(\frac{\int e^{-|w-u|^2/2\sigma^2}\nu(du)}{\int e^{-|w-u|^2/2\sigma^2}\mu(du)} - 1 \right) \right| \le |D^\nu(w;\sigma)|\epsilon \le M\epsilon, \tag{239}$$

where we have used the fact that $\mathrm{supp}(\mu), \mathrm{supp}(\nu) \subset \{|u| \le M\}$ in both estimates. Combining these estimates, we conclude that

$$\|D^\mu(\,\cdot\,;\sigma) - D^\nu(\,\cdot\,;\sigma)\|_{L^\infty(\mathbb{R}^d)} \le 2M\epsilon, \tag{240}$$

as claimed. □

Proof of Lemma SI 3.18. Due to the problem setup, it is easy to see that the optimal conditional denoiser $D_{\mathrm{opt}}(u_\sigma;\bar{u},\sigma)$ must be a shift by $m(\bar{u})$ of the optimal denoiser for the uniform data distribution $p(u) = \mathcal{U}[-1,1]$ over $[-1,1]$. It will therefore suffice to prove the statement for the optimal denoiser $D_{\mathrm{opt}}(u;\sigma)$ corresponding to this data distribution. We want to show:

1. $D_{\mathrm{opt}}(u;\sigma)$ is L^*-Lipschitz, uniformly as $\sigma \to 0$,
2. $\lim_{\sigma\to 0} D_{\mathrm{opt}}(u;\sigma) = g(u)$, given by (220).

To prove property (2.) we simply note that, by Lemma SI 3.9, the optimal denoiser converges to the closest point in the support of the data distribution $p(u) = \mathcal{U}[-1,1]$. The formula (220) is then immediate.

It remains to prove the uniform L^*-Lipschitz bound. To this end, we recall that, by Lemma SI 3.9, $D_{\mathrm{opt}}(w;\sigma)$ is given by

$$D_{\mathrm{opt}}(w;\sigma) = \frac{\int_{-1}^{+1} u e^{-(u-w)/2\sigma^2}\,du}{\int_{-1}^{+1} e^{-(u-w)/2\sigma^2}\,du}. \tag{241}$$

If we denote $\underline{u} = D_{\rm opt}(w;\sigma)$ for simplicity, then a short calculation implies that

$$|D'_{\rm opt}(w;\sigma)| = \frac{\int_{-1}^{+1}(u-\underline{u})^2 e^{-(u-w)^2/2\sigma^2}\,du}{\sigma^2\int_{-1}^{+1} e^{-(u-w)^2/2\sigma^2}\,du}. \tag{242}$$

We will prove that $|D'_*(w;\sigma)| \le L^*$ is uniformly bounded in σ and w. From the above formula, this is immediate for large σ; e.g. for $\sigma > 2$, we have

$$|D'_{\rm opt}(w;\sigma)| = \frac{\int_{-1}^{+1}(u-\underline{u})^2 e^{-(u-w)^2/2\sigma^2}\,du}{\sigma^2\int_{-1}^{+1} e^{-(u-w)^2/2\sigma^2}\,du} \le \frac{\int_{-1}^{+1} 4e^{-(u-w)^2/2\sigma^2}\,du}{4\int_{-1}^{+1} e^{-(u-w)^2/2\sigma^2}\,du} = 1, \qquad (\sigma > 2). \tag{243}$$

To establish an upper bound for $\sigma \in (0,2]$, we will distinguish between exterior points $\{w < -1\}, \{w > +1\} \subset [-1,1]^c$, and interior points $\{-1 < w < +1\} \subset [-1,1]$.

Exterior: We first consider the exterior domain $\{w < -1\}$. Fix $\xi > 0$, and set $w = -1-\xi$. Our goal is to bound $D'_{\rm opt}(-1-\xi;\sigma)$ for all $\xi > 0$ and $\sigma \in (0,2]$. Under the current assumptions, we have

$$|D'_{\rm opt}(-1-\xi;\sigma)| = \frac{\int_{-1}^{+1}(u-\underline{u})^2 e^{-(u+1+\xi)^2/2\sigma^2}\,du}{\sigma^2\int_{-1}^{+1} e^{-(u+1+\xi)^2/2\sigma^2}\,du} \tag{244}$$

After a change of variables $u \to u-1$ and noting that $\underline{u}$ minimizes the quadratic variation, it follows that

$$|D'_{\rm opt}(-1-\xi;\sigma)| \le \frac{\int_0^2 u^2 e^{-(u+\xi)^2/2\sigma^2}\,du}{\sigma^2\int_0^2 e^{-(u+\xi)^2/2\sigma^2}\,du}. \tag{245}$$

Expanding $(u+\xi)^2 = u(u+2\xi)+\xi^2$, we can write

$$|D'_*(-1-\xi;\sigma)| \le \frac{\int_0^2 u^2 e^{-u(u+2\xi)/2\sigma^2}\,du}{\sigma^2\int_0^2 e^{-u(u+2\xi)/2\sigma^2}\,du} \le \frac{\int_0^\infty u^2 e^{-u(u+2\xi)/2\sigma^2}\,du}{\sigma^2\int_0^2 e^{-u(u+2\xi)/2\sigma^2}\,du} \tag{246}$$

It will be convenient to estimate the denominator in terms of an integration over $[0,\infty)$ instead of $[0,2]$. To this effect, we note that

$$\int_2^\infty e^{-u(u+2\xi)/2\sigma^2}\,du = \int_0^\infty e^{-(2+u)(2+u+2\xi)/2\sigma^2}\,du \le e^{-2/\sigma^2}\int_0^\infty e^{-u(u+2\xi)/2\sigma^2}\,du. \tag{247}$$

And thus,

$$\int_0^2 e^{-u(u+2\xi)/2\sigma^2}\,du = \int_0^\infty e^{-u(u+2\xi)/2\sigma^2}\,du - \int_2^\infty e^{-u(u+2\xi)/2\sigma^2}\,du \tag{248}$$

$$\ge (1-e^{-2/\sigma^2})\int_0^\infty e^{-u(u+2\xi)/2\sigma^2}\,du. \tag{249}$$

It follows that

$$|D'_*(-1-\xi;\sigma)| \le \frac{\mathcal{I}}{1-e^{-2/\sigma^2}}, \qquad \mathcal{I} := \frac{\int_0^\infty u^2 e^{-u(u+2\xi)/2\sigma^2}\,du}{\sigma^2\int_0^\infty e^{-u(u+2\xi)/2\sigma^2}\,du}. \tag{250}$$

We note that $1-e^{-2/\sigma^2} \ge 1-e^{-1/2} > 0$ is uniformly lower bounded for all $\sigma \in (0,2]$. Therefore, to prove a uniform upper bound on $|D'_*(-1-\xi;\sigma)|$, it suffices to upper bound $\mathcal{I}$.

We now introduce $z := \xi/\sigma^2$, perform a change of variables $u \to \sigma u$, and write

$$\mathcal{I} = \frac{\int_0^\infty u^2 e^{-u(u+2z)/2}\,du}{\int_0^\infty e^{-u(u+2z)/2}\,du}. \tag{251}$$

Let us introduce $x = u(u+2z)$, so that

$$u = \sqrt{x+z^2} - z = \frac{x}{\sqrt{x+z^2}+z}, \tag{252}$$

and $dx = 2(u+z)du = 2\sqrt{x+z^2}\,du$. Then,

$$\mathcal{I} = \frac{\int_0^\infty \frac{x^2 e^{-x/2}\,dx}{(\sqrt{x+z^2}+z)^2\sqrt{x+z^2}}}{\int_0^\infty \frac{e^{-x/2}\,dx}{\sqrt{x+z^2}}}. \tag{253}$$

To bound this independently of $z \geq 0$, we first consider $z \in [0,1]$, and set $z = 0$ in the numerator, $z = 1$ in the denominator, to obtain the uniform bound:

$$\mathcal{I}|_{z\in[0,1]} \leq \frac{\int_0^\infty \sqrt{x} e^{-x/2}\,dx}{\int_0^\infty \frac{e^{-x/2}\,dx}{\sqrt{x+1}}}. \tag{254}$$

For $z \geq 1$, we observe that,

$$\int_0^\infty \frac{x^2 e^{-x/2}\,dx}{(\sqrt{x+z^2}+z)^2\sqrt{x+z^2}} \leq z^{-3}\int_0^\infty x^2 e^{-x/2}\,dx, \tag{255}$$

and

$$\int_0^\infty \frac{e^{-x/2}\,dx}{\sqrt{x+z^2}} \geq \frac{1}{\sqrt{1+z^2}}\int_0^1 e^{-x/2}\,dx \geq (2z)^{-1}\int_0^1 e^{-x/2}\,dx. \tag{256}$$

It follows that

$$\mathcal{I}|_{z\in[1,\infty)} \leq \frac{2\int_0^\infty x^2 e^{-x/2}\,dx}{z^2\int_0^1 e^{-x/2}\,dx}. \tag{257}$$

The last term is uniformly bounded for $z \in [1,\infty)$, and $\lesssim z^{-2}$ as $z \to \infty$. Recalling that $z = \xi/\sigma^2$ and $\xi = |w| - 1$, these two estimates on $\mathcal{I}$ imply an upper bound of the form,

$$|D'_{\mathrm{opt}}(w,\sigma)| \leq C\left(\frac{\sigma^2}{|w|-1+\sigma^2}\right)^2, \qquad (|w|>1,\ \sigma\in(0,2]). \tag{258}$$

Technically, we have only proved the above bound for $w < -1$. However, the same upper bound also holds for $w > +1$, by symmetry. From (258), we in fact observe that in the exterior domain, we have $D'_{\mathrm{opt}}(w;\sigma) \to 0$ except potentially at the boundary points $\{-1,+1\}$. This is consistent with the fact that $D_{\mathrm{opt}}(w;\sigma) \to \pm 1$ for $|w| > 1$.

Interior: Our final goal is to bound $D'_{\mathrm{opt}}(w;\sigma)$ in the interior, i.e. for all $w \in (-1,1)$. By symmetry about the origin, we may in fact assume that $w \in (-1,0)$. Under these assumptions, we have

$$|D'_{\mathrm{opt}}(w;\sigma)| \leq \frac{\int_{-1}^{+1}(u-w)^2 e^{-(u-w)^2/2\sigma^2}\,du}{\sigma^2\int_{-1}^{+1} e^{-(u-w)^2/2\sigma^2}\,du}. \tag{259}$$

Making the change of variables $u \to u - w$ and noting the set inclusion $w + [0,1] \subset [-1,1]$, it follows that

$$|D'_{\mathrm{opt}}(w;\sigma)| \leq \frac{\int_{-\infty}^{\infty} u^2 e^{-u^2/2\sigma^2}\,du}{\sigma^2\int_0^{+1} e^{-u^2/2\sigma^2}\,du} \tag{260}$$

Making the change of variables $\eta = u/\sigma$, and recalling that we consider $\sigma \in (0,2]$, we obtain

$$|D'_{\mathrm{opt}}(w;\sigma)| = \frac{\int_{-\infty}^{\infty} \eta^2 e^{-\eta^2/2}\, d\eta}{\int_0^{1/\sigma} e^{-\eta^2/2}\, d\eta} \le \frac{\int_{-\infty}^{\infty} \eta^2 e^{-\eta^2/2}\, d\eta}{\int_0^{1/2} e^{-\eta^2/2}\, d\eta}, \qquad (w \in [-1,1],\ \sigma \in (0,2]). \tag{261}$$

The right-hand side is independent of $w \in (-1,1)$ and $\sigma \in (0,2]$. Combining (243), (258) and (261), we have derived a unifom upper bound $|D'_{\mathrm{opt}}(w;\sigma)| \le L^*$, as desired. □

Proof of Lemma SI 3.19. Fix any $\phi \in \mathrm{Lip}_1(\mathbb{R} \times \mathbb{R})$, such that $\phi(0,0) = 0$. We note that by Kantorovich duality, $W_1(\mu^\Delta, \mu)$ is the supremum of

$$\mathcal{R}^\Delta(\phi) = \int \phi(u,\bar{u})\, d\mu^\Delta - \int \phi(u,\bar{u})\, d\mu, \tag{262}$$

over all such ϕ. By definition of μ^Δ and μ, we have

$$\mathcal{R}^\Delta(\phi) = \int_0^1 \phi(\mathcal{S}^\Delta(\bar{u}), \bar{u})\, d\bar{u} - \int_0^1 \int_{-1}^1 \phi(m(\bar{u}) + y, \bar{u})\, dy\, d\bar{u}. \tag{263}$$

To estimate $\mathcal{R}^\Delta(\phi)$ from above, we first observe that for any bounded function $f : \mathbb{R} \to \mathbb{R}$,

$$\int_0^1 f(\bar{u})\, d\bar{u} = \int_0^1 \mathbb{E}_{\eta\sim\mathcal{U}([0,\epsilon])} \left[f(\bar{u} + \eta) \right] d\bar{u} + r(f,\epsilon), \tag{264}$$

where the remainder $r(f;\epsilon)$ can be bounded by $|r(f;\epsilon)| \le 2\epsilon \|f\|_{L^\infty}$. This follows from the fact that, for uniformly distributed $\bar{u} \in [0,1]$ and $\eta \in [0,\epsilon]$, the sum $\bar{u} + \eta$ has uniform density $\equiv 1$ over $[\epsilon, 1]$, and is supported on $[0, 1+\epsilon]$.

Thus, taking $\bar{u} \sim \mathcal{U}[0,1]$, $\eta \sim \mathcal{U}[0,\epsilon]$, it follows that

$$\int_0^1 \phi(\mathcal{S}^\Delta(\bar{u}), \bar{u})\, d\bar{u} \le \mathbb{E}_{\bar{u}} \mathbb{E}_\eta \phi(\mathcal{S}^\Delta(\bar{u}+\eta), \bar{u}+\eta) + 2\|\phi\|_{L^\infty}\epsilon. \tag{265}$$

We next recall that

$$\mathcal{S}^\Delta(\bar{u}+\eta) = m(\bar{u}+\eta) + s_N(\bar{u}+\eta). \tag{266}$$

The first term is L^*-Lipschitz continuous, implying that

$$\mathcal{S}^\Delta(\bar{u}+\eta) = m(\bar{u}) + s_N(\bar{u}+\eta) + O_1(L^*\epsilon), \tag{267}$$

with implied constant for the remainder term bounded by 1. Let us introduce,

$$\tilde{\phi}(\bar{u}, y) := \phi(m(\bar{u}) + y, \bar{u}). \tag{268}$$

The last bound combined with the 1-Lipschitz continuity of ϕ, then implies that

$$\begin{aligned} \phi(\mathcal{S}^\Delta(\bar{u}+\eta), \bar{u}+\eta) &= \phi(m(\bar{u}) + s_N(\bar{u}+\eta), \bar{u}) + O(L^*\epsilon) && (269)\\ &= \tilde{\phi}(\bar{u}, s_N(\bar{u}+\eta)) + O(L^*\epsilon), && (270) \end{aligned}$$

where the only dependence on ϕ of the last term is via the Lipschitz bound L^*. The importance of this last expressions is that, if $\eta \sim \mathcal{N}[0,\epsilon]$ and if $N\epsilon \in \mathbb{N}$ is integer, then the push-forward,

$$y := s_N(\bar{u}+\eta) = \Lambda(N\bar{u} + N\eta), \tag{271}$$

has uniform distribution $y \sim \mathcal{U}[-1,1]$, independent of $\bar{u}$. Thus, we may choose $\epsilon := 1/N = \Delta$, for which it then follows that

$$\int_0^1 \phi(\mathcal{S}^\Delta(\bar{u}),\bar{u})\,d\bar{u} = \mathbb{E}_{\bar{u}}\mathbb{E}_\eta \phi(\mathcal{S}^\Delta(\bar{u}+\eta),\bar{u}+\eta) + O\left(\|\phi\|_{L^\infty}\Delta\right) \tag{272}$$

$$= \mathbb{E}_{\bar{u}}\mathbb{E}_\eta \tilde{\phi}(\bar{u}, s_N(\bar{u}+\eta)) + O\left((L^* + \|\phi\|_{L^\infty})\,\Delta\right) \tag{273}$$

$$= \mathbb{E}_{\bar{u}}\mathbb{E}_y \tilde{\phi}(\bar{u}, y) + O\left((L^* + \|\phi\|_{L^\infty})\,\Delta\right) \tag{274}$$

$$= \int_0^1 \int_{-1}^1 \tilde{\phi}(\bar{u}, y)\,dy\,d\bar{u} + O\left((L^* + \|\phi\|_{L^\infty})\,\Delta\right). \tag{275}$$

Recalling the definition of $\tilde{\phi}(\bar{u}, y) = \phi(m(\bar{u}) + y, \bar{u})$, it follows that

$$\mathcal{R}^\Delta(\phi) = \int_0^1 \phi(\mathcal{S}^\Delta(\bar{u}),\bar{u})\,d\bar{u} - \int_0^1\int_{-1}^1 \phi(m(\bar{u})+y,\bar{u})\,dy\,d\bar{u} \tag{276}$$

$$= O\left((L^* + \|\phi\|_{L^\infty})\,\Delta\right). \tag{277}$$

Taking the supremum over all $\phi \in \mathrm{Lip}_1$ such that $\phi(0,0) = 0$, we conclude that

$$W_1(\mu^\Delta,\mu) = \sup_\phi \mathcal{R}^\Delta(\phi) \le C L^* \Delta. \tag{278}$$

This proves the claim. □

SI 3.4.6 Proofs for Section SI 3.3

We next provide the proof of Proposition SI 3.7 and Proposition SI 3.8.

Deterministic Setting. We start with the proof of Proposition SI 3.7.

Proof of Proposition SI 3.7. The proof is very similar to the proof of Proposition SI 3.5. We first note that $\mathcal{S}^{(k)}(h)$ is highly oscillatory and has mean zero, implying that

$$\tilde{\Psi}^{(k)}(h) = \mathbb{E}_{\delta h}\left[\mathcal{S}^{(k)}(h+\delta h)\right] \to 0, \tag{279}$$

in $L^2([0,1])$. To complete the proof, it will thus suffice to show,

$$\lim_{k\to\infty} \mathbb{E}_{h\sim\bar{\mu}}\|\Psi^{(k)}(h)\|^2 = 0. \tag{280}$$

To this end, we simply note that

$$\mathbb{E}_{h\sim\bar{\mu}}\|\Psi^{(k)}(h)\|^2 = \mathbb{E}_{h\sim\bar{\mu}}\|\Psi^{(k)}(h) - \mathcal{S}^{(k)}(h)\|^2 + 2\mathbb{E}_{h\sim\bar{\mu}}\langle \Psi^{(k)}(h), \mathcal{S}^{(k)}(h)\rangle - \mathbb{E}_{h\sim\bar{\mu}}\|\mathcal{S}^{(k)}(h)\|^2. \tag{281}$$

Since Ψ^Δ minimizes the first term over all L^*-Lipschitz functions, we can compare with the 0 function to obtain,

$$\mathbb{E}_{h\sim\bar{\mu}}\|\Psi^{(k)}(h)\|^2 \le \mathbb{E}_{h\sim\bar{\mu}}\|0 - \mathcal{S}^{(k)}(h)\|^2 + 2\mathbb{E}_{h\sim\bar{\mu}}\langle \Psi^{(k)}(h), \mathcal{S}^{(k)}(h)\rangle - \mathbb{E}_{h\sim\bar{\mu}}\|\mathcal{S}^{(k)}(h)\|^2 \tag{282}$$

$$= 2\mathbb{E}_{h\sim\bar{\mu}}\langle \Psi^{(k)}(h), \mathcal{S}^{(k)}(h)\rangle. \tag{283}$$

It is straight-forward to show that there exists a constant $C > 0$, such that

$$\sup_{\mathrm{Lip}(\Psi)\le L^*} \mathbb{E}_{h\sim\bar{\mu}}\langle \Psi(h), \mathcal{S}^{(k)}(h)\rangle \le \frac{C}{k}. \tag{284}$$

Thus, we conclude that $\mathbb{E}_{h\sim\bar{\mu}}\|\Psi^{(k)}(h)\|^2 \le C/k \to 0$, as claimed. □

Probabilistic Setting. We now consider the probabilistic setting of conditional diffusion models. Our asymptotic results will hold for any $\tilde{\epsilon} > 0$. We thus assume $\tilde{\epsilon}$ to be fixed (arbitrarily). We recall that,

$$\nu^{(k)}(du \,|\, h) := \mathrm{Law}_{\delta h}\left[\mathbb{S}^{(k)}(h + \delta h)\right], \quad \delta h \sim \mathcal{U}([-\tilde{\epsilon}, \tilde{\epsilon}]), \tag{285}$$

and $\tilde{D}^{(k)}(u_\sigma; h, \sigma)$ denotes the optimal unconstrained conditional denoiser for $\nu^{(k)}$. The derivation of Proposition SI 3.8 is more involved, so we will first give an overview of the essential ingredients, and leave proofs for the next subsection.

Our first result shows that $\nu^{(k)} \approx \mathcal{U}(\mathbb{S}^1)$ is approximately equivalent to a uniform distribution:

Lemma SI 3.20. *Let $\mu(du \,|\, \bar{u}) = \mathcal{U}(\mathbb{S}^1)$ be a uniform measure. There exists a constant $C > 0$, independent of k, such that*

$$(1 - Ck^{-1})\mu(du \,|\, h) \le \nu^{(k)}(du \,|\, h) \le (1 + Ck^{-1})\mu(du \,|\, h). \tag{286}$$

Since the proof is completely analogous to the proof of Lemma SI 3.15, we will not discuss the details in this appendix. The result of the last lemma again allows us to easily identify the limit $D_{\mathrm{opt}} = \lim_{k\to\infty} \tilde{D}^{(k)}$, as an immediate consequence of Lemma SI 3.16 and Lemma SI 3.20:

Lemma SI 3.21. *Let $\mu = \mathcal{U}(\mathbb{S}^1) \otimes \mathcal{U}([0,1])$ be the uniform measure on $(u,h) \in \mathbb{S}^1 \times [0,1]$. Let D_{opt} denote the optimal (unconstrained) conditional denoiser for μ. Then we have,*

$$\mathbb{E}_{(u,h)\sim\mu}\mathbb{E}_{\eta\sim\mathcal{N}(0,\sigma^2)}\left\|\tilde{D}^{(k)}(u+\eta; h, \sigma) - D_{\mathrm{opt}}(u+\eta; h, \sigma)\right\|^2 \le Ck^{-1}. \tag{287}$$

Due to the simplicity of μ, the optimal denoiser D_{opt} can be computed explicitly, as shown next:

Lemma SI 3.22. *The optimal denoiser for $\mu = \mathcal{U}(\mathbb{S}^1) \otimes \mathcal{U}([0,1])$, is given by*

$$D_{\mathrm{opt}}(u; h, \sigma) = g_\sigma(|u|)\frac{u}{|u|}, \tag{288}$$

where $g_\sigma : \mathbb{R} \to \mathbb{R}$ is given by $g_\sigma(t) = I_1(t/\sigma^2)/I_0(t/\sigma^2)$, with $I_\alpha(z)$ the modified Bessel function of the first kind and of order α,

$$I_\alpha(z) = \frac{1}{\pi}\int_0^\pi \cos(\alpha\theta)e^{z\cos(\theta)}\,d\theta \sim \frac{e^z}{\sqrt{2\pi z}}, \quad \text{as } z \to \infty. \tag{289}$$

In particular,

$$\lim_{\sigma\to 0} D_{\mathrm{opt}}(u; h, \sigma) = \frac{u}{|u|}. \tag{290}$$

We include the details of the required calculation to prove Lemma SI 3.22 in the next subsection. As is clear from the limiting behavior as $\sigma \to 0$, we cannot have a uniform Lipschitz bound at the origin $u = 0$ in this case. Thus, we need to better understand the (local) Lipschitz behavior of D_{opt} in this limit.

Lemma SI 3.23. *Assume $D : \mathbb{R}^d \to \mathbb{R}^d$ is of the form $D(u) = g(|u|)\frac{u}{|u|}$. Let $A_\delta := \{|u| \ge \delta\}$. Then for any $\delta > 0$,*

$$\mathrm{Lip}(D|_{A_\delta}) \le \mathrm{Lip}(g|_{[\delta,\infty)}) + \frac{2\|g\|_{L^\infty}}{\delta}. \tag{291}$$

Given the explicit form of D_{opt}, we want to apply the last lemma with $g = g_\sigma$ to understand the Lipschitz behavior of D_{opt} near the origin. It remains to bound the Lipschitz constant of g_σ on $[\delta, \infty)$. This is the subject of the next lemma.

Lemma SI 3.24. *Let $g_\sigma(t) = g(t/\sigma^2)$ where $g(z) := I_1(z)/I_0(z)$ is a quotient of modified Bessel functions of the first kind. There exists a constant $C > 0$, such that for any $\delta > 0$,*

$$\mathrm{Lip}(g_\sigma|_{[\delta,\infty)}) \le \frac{C}{\delta}. \tag{292}$$

The following corollary is now immediate:

Corollary SI 3.25. *Let $D_{\mathrm{opt}}(u;\sigma) = g_\sigma(|u|)\frac{u}{|u|}$ be the optimal denoiser for $\mu = \mathcal{U}(\mathbb{S}^1)$. There exists a constant $C > 0$, such that for any $\delta, \sigma > 0$, and with $A_\delta := \{|u| \ge \delta\}$:*

$$\mathrm{Lip}(D_{\mathrm{opt}}|_{A_\delta}) \le C \min\left(\frac{1}{\delta}, \frac{1}{\sigma^2}\right). \tag{293}$$

The last corollary is the first ingredient required to apply Proposition SI 3.12. The second ingredient is contained in the following lemma:

Lemma SI 3.26. *If $\mu \in \mathcal{P}(\mathbb{R}^2)$ is a probability measure with $\operatorname{supp}\mu \subset \mathbb{S}^1$ and if $\delta \in (0, 1/2]$, then*

$$\mathrm{Prob}_{\mu_\sigma}\left[B_\delta(0)\right] \le \frac{\delta^2}{2\sigma} e^{-1/8\sigma^2}, \tag{294}$$

where $\mu_\sigma = \mu * \mathcal{N}(0, \sigma^2)$.

Given the above results, we can now finally come to the proof of Proposition SI 3.8.

Proof of Proposition SI 3.8. We recall that our goal is to show that

$$\limsup_{k\to\infty} \mathbb{E}_{(u,h)\sim\mu}\mathbb{E}_{\eta\sim\mathcal{N}(0,\sigma^2)} \left\| D^{(k)}(u+\eta;h,\sigma) - \tilde{D}^{(k)}(u+\eta;h,\sigma)\right\|^2 \le Ce^{-L^*/8C}. \tag{295}$$

Lemma SI 3.21 shows that $\tilde{D}^{(k)} \to D_{\mathrm{opt}}$ with D_{opt} the conditional diffusion model for μ. It will thus be enough to show that

$$\limsup_{k\to\infty} \mathbb{E}_{(u,h)\sim\mu}\mathbb{E}_{\eta\sim\mathcal{N}(0,\sigma^2)} \left\| D^{(k)}(u+\eta;h,\sigma) - D_{\mathrm{opt}}(u+\eta;h,\sigma)\right\|^2 \le Ce^{-L^*/8}. \tag{296}$$

Let

$$\mathcal{E} := \limsup_{k\to\infty} \mathbb{E}_{(u,h)\sim\mu}\mathbb{E}_{\eta\sim\mathcal{N}(0,\sigma^2)} \left\| D^{(k)}(u+\eta;h,\sigma) - D_{\mathrm{opt}}(u+\eta;h,\sigma)\right\|^2. \tag{297}$$

By Proposition SI 3.12, applied with $A := A_\delta$ and $\mathbb{R}^d \setminus A = B_\delta(0)$, we have the upper bound,

$$\mathcal{E} \le \limsup_{k\to\infty} C\left\{L^* W_1(\mu^{(k)}, \mu) + \mathrm{Prob}_{\mu_\sigma}[B_\delta(0)] + \mathrm{Prob}_{\mu_\sigma^{(k)}}[B_\delta(0)]\right\}, \tag{298}$$

under the constraint that $\mathrm{Lip}(D_{\mathrm{opt}}|_{A_\delta}) \le L^*$. By Corollary SI 3.25, this is the case provided that $C\min(\delta^{-1}, \sigma^{-2}) \le L^*$. An argument completely analogous to the proof of Lemma SI 3.19 then shows that $\lim_{k\to\infty} W_1(\mu^{(k)}, \mu) = 0$. And Lemma SI 3.26 implies that

$$\mathcal{E} \le \frac{C\delta^2}{\sigma} e^{-(1-\delta)^2/2\sigma^2}. \tag{299}$$

Again, we emphasize our constraint on $C\min(\delta^{-1}, \sigma^{-2}) \le L^*$. There are two cases, either (i) $\sigma^2 \ge C/L^2$ and we are free to let $\delta \to 0$, or (ii) $\sigma^2 < C/L^*$ and we will choose $\delta = C/L^*$. In the first case, we obtain

$$\mathcal{E} = 0. \tag{300}$$

In the second case, we have $1/\sigma^2 \geq L^*/C$, and we obtain

$$\mathcal{E} \leq \frac{C}{(L^*)^{3/2}} e^{-L^*/8C}, \tag{301}$$

for sufficiently large L^*. It follows that, independent of σ, we have the following upper bound,

$$\mathcal{E} \leq C e^{-L^*/8C}. \tag{302}$$

In particular, it follows that

$$\limsup_{k\to\infty} \mathbb{E}_{h,u,\eta} \| D^{(k)}(u_\sigma; h, \sigma) - \tilde{D}^{(k)}(u_\sigma; h, \sigma) \|^2 \leq C e^{-L^*/8C}. \tag{303}$$

□

Proofs of Lemma SI 3.22, Lemma SI 3.23, Lemma SI 3.24 and Lemma SI 3.26. In the following we detail the proofs of Lemma SI 3.22, Lemma SI 3.23, Lemma SI 3.24 and Lemma SI 3.26.

Proof of Lemma SI 3.22. By the explicit formula for D_{opt}, we have

$$D_{\mathrm{opt}}(w; h, \sigma) = \frac{\int u \exp(-(u-w)^2/2\sigma^2) p(u \,|\, h)\, du}{\int \exp(-(u-w)^2/2\sigma^2) p(u \,|\, h)\, du}. \tag{304}$$

Since the joint probability on (h, u) is a product measure, the conditioning on h is irrelevant, and $p(u \,|\, h) = \mathcal{U}(\mathbb{S}^1)$. By the symmetries of the problem, the expectation perpendicular to w vanishes, so that we can write

$$D_{\mathrm{opt}}(w; h, \sigma) = g_\sigma(|w|) \frac{w}{|w|}. \tag{305}$$

For the computation of $g_\sigma(|w|)$, we may wlog assume that $w = (|w|, 0)$ points in the first coordinate direction. We dot the above formula for D_{opt} by $w/|w|$, and parametrize $u = (\cos\theta, \sin\theta)$ by the angular variable $\theta \in [0, 2\pi]$. It follows that

$$g_\sigma(|w|) = \frac{\int_0^{2\pi} \cos\theta \exp\left(-\left\{(\cos\theta - |w|)^2 + \sin^2\theta\right\}/2\sigma^2\right)\, d\theta}{\int_0^{2\pi} \exp\left(-\left\{(\cos\theta - |w|)^2 + \sin^2\theta\right\}/2\sigma^2\right)\, d\theta}. \tag{306}$$

After expansion of the squares and elementary simplifications, we obtain

$$g_\sigma(|w|) = \frac{\int_0^{\pi} \cos\theta e^{|w|\cos(\theta)/\sigma^2}\, d\theta}{\int_0^{\pi} e^{|w|\cos(\theta)/\sigma^2}\, d\theta} = \frac{I_1(|w|/\sigma^2)}{I_0(|w|/\sigma^2)}. \tag{307}$$

This is the claimed formula. □

Proof of Lemma SI 3.23. Given $u, v \in \mathbb{R}^d$, we have

$$|D(u) - D(v)| \leq \left| g(|u|)\frac{u}{|u|} - g(|v|)\frac{u}{|u|} \right| + \left| g(|v|) \left(\frac{u}{|u|} - \frac{v}{|v|} \right) \right| \tag{308}$$

$$\leq \mathrm{Lip}(g|_{[\delta,\infty)})|u - v| + \|g\|_{L^\infty} \left| \frac{u}{|u|} - \frac{v}{|v|} \right|. \tag{309}$$

To estimate the last term, we note that

$$\left| \frac{u}{|u|} - \frac{v}{|v|} \right| \leq \left| \frac{u}{|u|} - \frac{v}{|u|} \right| + \left| \frac{v}{|u|} - \frac{v}{|v|} \right| \tag{310}$$

$$\leq \frac{1}{|u|}|u-v| + |v|\left|\frac{1}{|u|} - \frac{1}{|v|}\right| \tag{311}$$

$$= \frac{1}{|u|}|u-v| + \frac{1}{|u|}\left||v| - |u|\right|. \tag{312}$$

It thus follows that, for $|u| \geq \delta$,

$$\left|\frac{u}{|u|} - \frac{v}{|v|}\right| \leq \frac{2}{\delta}|u-v|. \tag{313}$$

Substitution in the first inequality now yields the claimed bound on $\mathrm{Lip}(D|_{A_\delta})$. □

Proof of Lemma SI 3.24. We have

$$\mathrm{Lip}(g_\sigma|_{[\delta,\infty)}) = \|g_\sigma'\|_{L^\infty([\delta,\infty))} \tag{314}$$

$$= \frac{1}{\sigma^2}\|g'\|_{L^\infty([\delta/\sigma^2,\infty))} \tag{315}$$

$$= \frac{1}{\delta}\frac{\delta}{\sigma^2}\|g'\|_{L^\infty([\delta/\sigma^2,\infty))} \tag{316}$$

$$\leq \frac{1}{\delta}\|zg'(z)\|_{L^\infty([\delta/\sigma^2,\infty))} \tag{317}$$

$$\leq \frac{1}{\delta}\|zg'(z)\|_{L^\infty([0,\infty))} \tag{318}$$

The claim follows by observing that $C \coloneqq \|zg'(z)\|_{L^\infty([0,\infty))} < \infty$. This last observation follows from the relationships $I_0'(z) = I_1(z)$, $I_1'(z) = I_0(z) - \frac{1}{z}I_1(z)$, so that

$$g'(z) = \frac{I_1'(z)I_0(z) - I_1(z)I_0'(z)}{I_0(z)^2} = (1 - g(z)^2) - \frac{1}{z}g(z), \tag{319}$$

and the asymptotics of $I_0(z)$, $I_1(z)$ as $z \to \infty$; Indeed, we have $zg'(z) = z(1-g(z)^2) - g(z)$, and the asymptotic expansion of $I_0(z)$, $I_1(z)$ as $z \to \infty$ implies that $g(z) \to 1$ and $1 - g(z)^2 \sim C/z$ for some constant C. Hence $zg'(z)$ remains uniformly upper bounded as $z \to \infty$. □

Proof of Lemma SI 3.26. For any $y \in \mathrm{supp}(\mu)$ and $x \in B_\delta(0)$, we clearly have $|x-y|^2 \geq (1-\delta)^2$. Hence,

$$\mathrm{Prob}_{\mu_\sigma}[B_\delta(0)] = \int\int 1_{B_\delta}(x)\frac{e^{-|x-y|^2/2\sigma^2}}{2\pi\sigma}\mu(dy)\,dx \tag{320}$$

$$\leq \frac{e^{-(1-\delta)^2/2\sigma^2}}{2\pi\sigma}|B_\delta| = \frac{\delta^2}{2\sigma}e^{-(1-\delta)^2/2\sigma^2}. \tag{321}$$

□

SI 4 Further Experimental Results

In this section, we expand on the discussion about the experimental results in the Main Text by contextualizing additional results and better highlighting the ones briefly discussed in the Main Text.

SI 4.1 GenCFD Generates High-quality Samples of the Flow

We start by recalling the ability of GenCFD to generate high-quality samples, drawn from the conditional distribution, which was already discussed in the Main Text. To this end, in Main Text Fig. 2 (A) and Fig. SI 7 here, we present samples of the conditional kinetic energy (square of the norm of the velocity field), corresponding to the TGV and CSF datasets, generated by GenCFD and compare them to ground-truth samples generated with the underlying CFD simulator. We observe from these figures that the pointwise kinetic energy samples are realistic for both datasets, with little to distinguish them visually from the ground-truth samples. In particular, the small-scale eddies are captured well in the generated velocity fields, and the diffusion model also provides a rich diversity of samples despite each of them corresponding to the *same initial condition.*

An even harder test of sample quality is investigated by computing the *vorticity* from the generated velocity fields by taking a *curl* and plotting the resulting pointwise vorticity intensity samples Main Text Fig. 2 (B) and Fig. SI 8 here, for TGV and CSF, respectively. When compared to the ground truth, we find the computed vorticity samples to be accurate with a realistic rendition of small-scale features for both datasets, particularly of looping vortex tubes which are the characteristics of a turbulent fluid [11]. These realistic vorticity profiles, generated by our AI algorithm, are noteworthy as the model itself has never been shown a vorticity field and has to infer it indirectly from the generated velocity field by taking its derivatives. This suggests that the covariate structures of the velocity fields are well captured by the AI algorithm.

In contrast to the high quality of samples generated by GenCFD, all the other ML baselines (see Main Text Figs. 2 (A and B) and Figs. SI 7 and SI 8 here, for C-FNO, which is the strongest baseline) only lead to poor quality as well as little diversity in terms of the generated samples, both for the kinetic energy and for the vorticity. In general, the samples generated by these baselines collapse to a field close to the mean velocity (and vorticity) field, rather than representing the statistical spread of the target conditional distribution.

This observation of high-quality sample generation with GenCFD is further reinforced in Figs. SI 30 to SI 32, where samples with other initial conditions for the CSF dataset are presented.

Moreover, in Fig. SI 12, we present samples of the density for the CSI dataset, generated by GenCFD and compare it to ones obtained from the ground truth and C-FNO to observe that this high quality of sample generation by GenCFD is also present for compressible fluid flow. In particular, we observe that GenCFD is able to generate the leading shock wave, the rising turbulent plume in its wake, and also the small-scale eddies marking the turbulent mixing zone near the trailing shocks. On the other hand, C-FNO is able to approximate the leading shock wave accurately but smooths out the rising plume while failing to generate the small-scale turbulent eddies in the wake.

In Figs. SI 15 and SI 16, we present the pointwise kinetic energy and vorticity intensity, respectively, of the nozzle flow experiment. Even for this experiment that is prototypical of real-world engineering flows, we see that GenCFD provides high-quality and diverse samples of the flow whereas the best performing baseline (UViT) regresses to the mean. In particular, the ability of GenCFD to generate this complex flow, with a very complicated distribution of eddies that are both wall-bounded and yet have a

freestream component, is noteworthy.

Finally, in Fig. SI 19, we visualize the x-component of the velocity field in the convective boundary layer (CBL) experiment to again find that GenCFD is able to generate realistic, diverse samples of the flow field, whereas the best performing baseline (UViT) smears out all the detailed flow features, especially the plumes going up and down due to convection.

SI 4.2 GenCFD Accurately Approximates Statistical Quantities of Interest

The high quality of AI-generated samples of fluid flow encourages us to test how well GenCFD approximates the statistical quantities of interest. We check this by computing the mean and the standard deviation of the conditional velocity field, generated by GenCFD and comparing them with the underlying ground truth and the statistics of the ML baselines. The results for all the datasets are presented in Main Text Fig. 2 (C and D) for TGV, Figs. SI 9, and SI 10 for CSF, Fig. SI 13 for CSI, Fig. SI 17 and Main Text Fig. 3 (A) for NF and Fig. SI 20 and Main Text Fig. 3 (C) for CBL. We observe from these figures that GenCFD approximates the mean and variance accurately. In contrast, the ML baselines (we present figures for the best performing baseline in each dataset) can approximate the mean fairly accurately but fail at approximating the standard deviation. This (approximate) collapse to mean for the ML baselines is also seen when we plot the one-point PDFs in Main Text Fig. 2 (E) for TGV, Fig. SI 11 for CSF, Fig. SI 14 for CSI, Fig. SI 18 for NF and Fig. SI 21 for CBL. In contrast, GenCFD accurately approximates the underlying point PDFs. We would like to point out that this ability of GenCFD to accurately predict the PDFs is particularly noteworthy as the spread out PDFs have to be generated from inputs (initial conditions) that are (approximately) Dirac distributions.

This accurate approximation of statistical quantities of interest with GenCFD is not just qualitative but also quantitative. We demonstrate this accuracy by presenting the errors in computing the mean, the standard deviation as well as (the first marginal of) the 1-Wasserstein distance between the conditional distributions and the CRP Scores (CRPS), computed by GenCFD and other ML baselines, and the ground truth computed with the CFD solvers in Tabs. SI 11, SI 12, SI 13, SI 14 and SI 15 for TGV, CSF, CSI, NF and CBL, respectively. We see from these tables that GenCFD is *significantly more accurate* than the ML baselines, particularly with respect to the standard deviation and the Wasserstein distance with gains ranging up to one order of magnitude for the Wasserstein distance and the standard deviation and demonstrating the ability of GenCFD for accurate statistical computation of turbulent fluid flows.

SI 4.3 GenCFD Provides Accurate Spectral Resolution

Energy spectra are key quantities of interest for the theoretical, experimental and computational study of turbulent fluid flows [56]. In particular, the famous K41 theory of turbulence is based on predicting the decay of these spectra with respect to wave number. Hence, spectral resolutions are often used as proxies for judging the quality of physics- [57] or AI-based [58] simulators of turbulent fluid flows. We compute the energy spectra for the GenCFD and baseline generated fields for all the datasets and plot them in Main Text Fig. 2 (F) for TGV and Fig. SI 24 here for the rest of the datasets. We clearly observe from these figures that GenCFD attains high spectral accuracy and the energy content, up to the highest frequencies, is accurately generated. On the other hand, the deterministic ML baselines are only able to generate a small fraction of the spectrum accurately and lose spectral resolution quickly. This poor effective spectral resolution of deterministic ML models has also been observed in the context of weather and climate modeling, see for instance Fig. 1 of [58].

SI 4.4 GenCFD Scales with Data

A key attribute of modern AI models is their ability to scale with data [59]. To test this, we compute the errors in standard deviation and with respect to the 1-Wasserstein metric for GenCFD as the number of training samples for the CSF dataset varies and plot the results in Fig. SI 23. We observe from this figure that these test errors with GenCFD decrease as the amount of training data increases.

SI 4.5 Statistical Computation with GenCFD is Robust

We recall that the test task for GenCFD is *out-of-distribution* as the test distribution is a Dirac measure whereas the training distribution is spread out. Yet, GenCFD computes the samples as well as the statistics accurately. We test this *generalization ability* further by choosing the functions, on which the test distribution is concentrated (54), from yet another distribution. To this end, we consider the CSF dataset and choose $p \in \{8, 9, 10, 11, 12\}$ uniformly at random for each sample, rather than constant and equal to 10. Additionally, the perturbation functions σ_y^i, and σ_z^j are extended with another parameter $\xi_y, \xi_z \sim \mathcal{U}_{[-0.0625, 0.0625]}$ by setting

$$\sigma_y^j(x) = \delta \sum_{k=1}^{p} \alpha_k^y \sin(2\pi k x - \beta_k^y) - \xi_y, \tag{322}$$

$$\sigma_z^j(x) = \delta \sum_{k=1}^{p} \alpha_k^z \sin(2\pi k x - \beta_k^z) - \xi_z. \tag{323}$$

This leads to a distribution ν, which is different from the training distribution.

Zero-shot Evaluation is performed by sampling the initial condition $\bar{u} \sim \nu$ and feeding it directly into the pretrained models (GenCFD and baselines). No adjustments or modifications to the pretrained models are needed for this evaluation.

For fine-tuning, the models are trained using the objectives (32) on 300 samples drawn from the distribution ν. During this step, all pretrained model parameters are updated.

Even with no additional training (*Zero-Shot mode*), GenCFD is able to generate high quality samples (see Fig. SI 33) as well as approximate mean, standard deviation and probability distribution quantitatively (Table SI 18) to reasonable accuracy. For instance, the 1-Wasserstein distance between the ground truth and the generated distribution increases, on average, by a factor of 2 over the previously tested distribution (see Table SI 12), with this *zero-shot* evaluation. By further *fine-tuning* the model with merely 300 trajectories generated from initial data, drawn from the new training distribution ν, the error is reduced to the previous levels, see Table SI 18 and compare with Table SI 12 further showcasing the ability of GenCFD to handle *distribution shifts.*

Another avenue for checking robustness arises when we check how the statistical errors with GenCFD increase over time. From Table SI 19, where we present the 1-Wasserstein distance between the ground truth and the GenCFD generated conditional distribution for the CSF dataset, we observe that after a modest increase in the beginning of evolution when the turbulence kicks in, the error is approximately constant for the time period when the turbulence is fully developed.

This demonstration of the robustness of our proposed algorithm to *time evolution* is particularly pertinent for the approximation of the Taylor–Green vortex as the flow starts laminar (in fact smooth) and dynamically transitions to turbulence over time. For instance, the flow at time $T = 0.8$ is still markedly laminar while it has become turbulent by time $T = 2$. Can GenCFD still be robust with such transitions from laminar to turbulent flow? In particular, can it be accurate at also approximating

deterministic flows? These questions are answered qualitatively in Figs. SI 25, SI 26, SI 27 and SI 28, from which we observe that GenCFD continues to provide realistic samples for both velocity and vorticity and approximates the mean and especially, the standard deviation, for the underlying laminar flow accurately. This observation is further buttressed by the results in Table SI 16 where we see that GenCFD has lower errors in every single metric when compared to the ML baselines. Thus, this experiment clearly showcases the ability of GenCFD to accurately approximate both deterministic and stochastic fluid flow.

SI 4.6 Statistical Computation with GenCFD is Fast

The computational cost of inference with the GenCFD algorithm scales *linearly* in the number of steps required for solving the reverse-SDE (12). In Table SI 20, we show how robust our algorithm is with respect to the number of steps in solving the reverse-SDE to find that as few as 16 steps suffice in ensuring acceptable statistical accuracy, with 32 steps being almost as good as 128 steps. Given this observation, we can deploy our model with 32 steps for the reverse SDE. The resulting inference time with GenCFD, in comparison to the underlying CFD solvers, is presented in Table SI 21. We observe from the figure that GenCFD requires approximately 1 to 4 seconds for a single inference run on a NVIDIA RTX4090 24GB GPU, for all the test cases that we have considered. These inference times will be even faster for more powerful GPUs. In contrast, the run times for CFD solvers vary considerably based on the underlying numerical method and on the hardware used to run them. An optimized code such as **Azeban** can perform 128^3 CFD simulations on a periodic domain with spectral viscosity method in approximately 10 seconds on GPUs. However, this run time is significantly larger at approximately 20 minutes, even on state-of-the-art H100 96GB GPUs for a more complicated test case like the nozzle flow, even when a scalable solver such as **OpenLB** is employed. On the other hand, all the CFD simulations, which are performed on CPUs, required run times in the order of hours. This is indicative of the real world as most CFD codes run on CPUs. Hence, from Table SI 21, we see that GenCFD can provide a speedup, ranging from one to three orders of magnitude with respect to GPU-based CFD codes while providing a three to five orders of magnitude speedup over CPU-based CFD codes. It is precisely this computational speed, coupled with accuracy, that makes GenCFD attractive for applications in many areas of fluid dynamics.

Finally, we point out that it is possible to further accelerate GenCFD by leveraging a latent diffusion model [60], where the generation is carried in a lower-dimensional latent space. This technique is key to generate high-resolution images at a reasonable cost while incurring a negligible perceptive deterioration. We leave this approach for future work, as it would require training variational autoencoders bespoke for the fluid flows which are able to preserve the statistical information.

Numerical Results with the Toy Models. Recalling Toy Model #1, which is given by the map $\mathcal{S}^{\Delta}$ (97), we present numerical results with a diffusion model and an underlying deterministic ML baseline in Fig. SI 34, from where we observe that the deterministic ML approximation does accurately approximate $\mathcal{S}^{\Delta}$, for moderate values of $\Delta \approx 0.1$. But for even lower values of Δ, the deterministic approximation collapses to the mean as predicted by the theory presented earlier. On the other hand, the diffusion model is able to approximate $\mathcal{S}^{\Delta}$ accurately, for moderate values of $\Delta \approx 0.05$ when the mapping is not too oscillatory in a deterministic manner, while at the same time, being able to approximate the statistical limit for very small values of $\Delta \approx 0.002$. Consistent with our theory, this ability of diffusion models to be robust with respect to any value of Δ in this case is worth highlighting. It also matches the empirical observation that GenCFD was able to approximate the Taylor–Green vortex flow accurately for both the laminar and turbulent regimes.

In Fig. SI 34, we also illustrate the different modes through which deterministic ML models and diffusion

models *learn during training.* A deterministic ML model first learns the mean and then adds oscillations as more and more gradient descent steps are taken, consistent with the well-known spectral bias of neural networks [52]. If the underlying map is too oscillatory, it simply does not add the oscillations and predicts the mean, which yields a (local) minimum for the L^2 loss. In contrast, diffusion models do the opposite. Already, very early in the training, they capture the statistical limit measure and as more gradient descent steps are taken, they start *clearing out* the measure to reveal more of the underlying deterministic oscillations. If the underlying map is too oscillatory, the diffusion model sticks to the measure-valued output even when more training steps are taken, enabling it to capture both deterministic approximations as well as statistical information, as necessary.

Finally in Fig. SI 35, we present results with a diffusion model and the underlying deterministic baseline on Toy Model #2, which was described and rigorously analyzed in the previous section. We observe from this figure that while the deterministic model is accurate for low wave numbers (around $k \approx 10$), it collapses to the mean for $k \geq 30$, even with a lot of training steps. This also implies that the phase space approximation is poor and the underlying constraint is violated at high wavenumber. In contrast, the diffusion model is able to learn the underlying map, both for small as well as large wavenumbers, even with a few training steps. The underlying constraint is satisfied for any of the tested wavenumbers and the contrast between the deterministic and diffusion models is shown in the (synthetic) spectra plotted in Fig. SI 24 (bottom row). The diffusion model is able to capture structures at all wave numbers whereas the deterministic model has a limited spectral resolution, explaining the spectral findings for fluid flows in Fig. SI 24. To obtain these results, we used the same model specifications and training procedure as described in Sec. SI 2.9.1. In contrast to that section, the deterministic model now maps a one-dimensional input to a two-dimensional output, $h \mapsto \Psi_{\text{det}}(h) \approx \mathcal{S}^{(k)}(h)$. The diffusion model has four-dimensional input, (u_σ, h, σ), where $u_\sigma \in \mathbb{R}^2$, $h \in [0,1]$, $\sigma \in \mathbb{R}$, and outputs a two-dimensional (denoised) vector u, $(u, h, \sigma) \mapsto D(u; h, \sigma)$. In each case, the model is trained on 2048 samples, with $h \sim \mathcal{U}([0,1])$ uniformly sampled over the input range $[0,1]$. All other implementation details are identical to the ones specified in Sec. SI 2.9.1.

SI 4.7 Comparison with CRPS Training

As demonstrated extensively, neural operators such as U-ViT and CFNO, that are trained to minimize the mean absolute (square) error, regress to the mean and do not capture the statistical spread of the target conditional distribution. As mentioned earlier, a similar regression to the mean is also observed when AI models are applied for medium-term weather forecasting [58, 2]. Consequently, there is considerable interest in the weather and climate modeling community for using different loss functions which better represent the inherently stochastic nature of the underlying learning tasks. A particularly attractive framework is to train with (different versions of) CRPS loss, for instance in [61]. Below, we adapt this training framework for our learning tasks and compare it with GenCFD.

Let $\mathcal{G}\colon \mathcal{X} \mapsto \mathcal{Y}^{n_E}$ be a neural surrogate model that approximates a dynamical system of interest. Here, $\mathcal{X}$ is an input space that also contains randomly injected noise channel as in [61]. The space $\mathcal{Y}$ is the output space. The integer n_E is the *ensemble size*, whose role will be explained later. Let $\mathcal{B} = \{(x_i, y_i)\}_{i=1}^N$ be a batch of I/O pairs. The CRPS loss is given by

$$\mathcal{L}(\mathcal{B};\, \mathcal{G}) = \frac{1}{N}\sum_{i=1}^{N}\Big(\frac{1}{n_E}\sum_{j=1}^{n_E} ||\mathcal{G}(x_i)_j - y_i||_2 - \frac{\alpha}{2n_E(n_E-1)}\sum_{j=1}^{n_E}\sum_{k=1}^{n_E} ||\mathcal{G}(x_i)_j - \mathcal{G}(x_i)_k||_2\Big). \tag{324}$$

Here, α is a scaling constant that influences the magnitude of the second term of the loss which influences *the spread of the ensemble*. The first term is responsible *for capturing the mean* of the sample. The loss

decomposition reads as

$$\mathcal{L}(\mathcal{B};\, \mathcal{G}) = \mathcal{L}_1(\mathcal{B};\, \mathcal{G}) - \alpha \cdot \mathcal{L}_2(\mathcal{B};\, \mathcal{G}), \tag{325}$$

with $\mathcal{L}_1$ aiming to approximate the mean as accurately as possible and $\mathcal{L}_2$ trying to maximize the underlying variance (statistical spread) of the distribution. It is empirically observed that a stable convergence can be achieved by setting the $\alpha = 0.5$. According to [61], a good choice for the ensemble size is $n_E = 4$.

We test CRPS training in the case of the Taylor–Green vortex experiment. We use a ViT with a convolutional encoder and decoder (the first and the last blocks of layers) as a backbone for $\mathcal{G}$ and present the results in Table SI 8 below. From this table, we clearly observe that ViT with CRPS training captures the mean with similar accuracy to GenCFD as well as C-FNO. It is more accurate than the deterministically trained C-FNO in terms of both the variance and the Wasserstein distance. However, there is still a big gap in its performance, amounting to a factor of 5-10x less accuracy, on the statistical metrics, when compared to GenCFD. This difference is clearly visualized in Figs. SI 3 and SI 4, where we compare samples of ground-truth x-velocity and vorticity with the ones obtained from ViT with CRPS training. It is clear that the CRPS training does not suffice to replicate the fine-scale structure of the underlying distribution. Rather, it approximates the mean and adds some uncorrelated noise to provide better approximation of the statistical spread. This should be contrasted with the results shown for GenCFD (see Fig. 2 (B), where the sampled fields were much more similar to the ground-truth field and the statistical spread was captured accurately.

Given these results, it is natural to examine if the ensemble size n_E in (324) was too small for CRPS trained models to be effective. To test this proposition, we vary the ensemble size n_E in the Taylor–Green vortex experiment to study the impact of this parameter on performance. The results are given in Table SI 9. When $n_E = 1$, we retrieve collapse to the mean phenomenon for a ViT neural operator. As the number of ensemble members is increased to 4, the error in both variance as well as Wasserstein distance reduces significantly. However, increasing the ensemble size further to 16, only leads to a deterioration in statistical accuracy, consistent with the findings of [61], from which the choice of $n_E = 4$ was adopted. Thus, the poor performance of CRPS in our setting is not attributable to the number of ensemble members but is grounded in the basic nature of our learning task, where a relatively long-time statistical evolution of the system has to be tracked, in contrast to the short- to medium-term weather forecasting which involves relatively shorter time scales.

SI 4.8 Role of Incompressibility

Velocity fields for incompressible fluids, such as those considered in many of our numerical experiments (Taylor–Green vortex, cylindrical shear flow), satisfy the divergence-free condition, i.e., $\mathrm{div}(u) = 0$, for all times. GenCFD does not explicitly enforce the underlying PDEs, including this incompressibility criterion. Thus, it is a priori unclear if the velocity fields, sampled by GenCFD, are physically consistent in this sense.

However, it is straightforward to enforce the divergence-free condition for the velocity fields, generated by GenCFD by using the well-known *Leray projection operator*. To do this, we consider,

$$\mathbb{P}\hat{u}_k = \left(1 - \frac{kk^T}{|k|^2}\right) \cdot \hat{u}_k, \tag{326}$$

where k is the wave vector and $\hat{u}_k$ is the vector of Fourier coefficients of the x-, y-, and z-component of the velocity field, generated by GenCFD at any given time instant (time-dependence is suppressed for

notational simplicity), at wave vector k. These projected solutions are then compared to a reference solution computed with a classical numerical solver generating the ground-truth data. Table SI 10 shows the obtained statistical metrics. As one can clearly observe, the results of the Leray-projected velocity fields match almost exactly with those of the underlying flow in all instances. Moreover, the Leray-projected fields are visually very similar to the GenCFD generated fields. These results show that, even if the divergence constraint is not imposed explicitly, the velocity fields generated by GenCFD, approximate the ground-truth velocity fields with as high a statistical accuracy as imposing incompressibility explicitly through Leray projection, would do.

SI 4.9 Additional Statistical Metrics for Turbulent Fluid Flows.

There is a deep theory characterizing turbulent fluid flows [56] and 1-point statistics, such as computing the mean and the variance of the velocity field, do not suffice to capture the full range of rich physics that a turbulent fluid flow can encapsulate. So far, we have focused on whether GenCFD can accurately approximate the 1-point statistics of the underlying ground-truth conditional distributions, generated by numerical methods at fixed resolutions and have clearly demonstrated that GenCFD is successfully able to do so.

The natural question that arises is whether GenCFD can go further and approximate even finer properties of turbulent fluids accurately. In terms of 1-point statistics, we clearly go beyond mean and variance by also presenting the Wasserstein distance between 1-point velocity PDFs to show that GenCFD accurately approximates these comprehensive 1-point statistics, outperforming the baselines, including CRPS trained ViTs. To further investigate the statistical accuracy of GenCFD, we consider the 3rd, 4th and 5th order central moments of the velocity field for the Taylor–Green vortex at time $T = 2$ and present the errors in these quantities with GenCFD and the baselines in Table SI 17. We observe that GenCFD approximates these higher moments more accurately than baselines. The resulting high-order moments are visualized in Fig. SI 29 and we observe how well the moments generated by GenCFD approximate the ground truth. These results establish that 1-point statistics of turbulent fluid flows are accurately approximated by GenCFD.

However, it is well-known that 1-point statistics do not fully characterize turbulent fluid flows. Rather, 2-point statistics are a necessary component of the physical theories of turbulence [56]. How well does GenCFD capture such two-point statistics? A partial answer is already provided in Fig. SI 24 where we present the energy spectra of all the flows that we considered in this paper and show how well GenCFD is able to approximate the spectrum of the ground-truth distribution generated by numerical methods. In particular, GenCFD captures a large proportion of length scales in flows. In contrast, the deterministic baselines only approximate the large scales and fail at small scale approximation. The energy spectra are particularly relevant for homogeneous isotropic turbulence on account of the well-known Kolmogorov's 5/3-rd law on the spectral decay. However, given the resolutions that we consider for generating the ground truth, i.e., 128^3, the spectral properties of the flow that are not that well-resolved to begin with, steering the numerical solution to a very small inertial range for the 5/3-rd law to be valid.

Moreover, the spectrum is a Fourier transform of the so-called 2nd-order structure function. So accurately approximating the ground-truth spectrum is tantamount to an accurate approximation of its 2nd-order structure function.

Another relevant object in the characterization of turbulence is the *3rd-order longitudinal structure*

function given by,

$$S^3_{\parallel,r}(u) = \int_{\mathcal{F}} \int_D \int_{\partial B_r(0)} \left((u(x+h) - u(x)) \cdot \frac{h}{|h|} \right)^3 \,\mathrm{d}\rho \,\mathrm{d}x \, d\mu, \tag{327}$$

where μ is the underlying statistical solution, with the explicit time-dependence suppressed for notational convenience.

The role of the third-order structure function is highlighted by the 4/5-th law of Kolmogorov, where one can show that under the assumptions of statistical homogeneity, isotropy, and stationarity of the flow, one obtains [56]

$$S^3_{\parallel,r}(u) = -\frac{4}{5}\varepsilon r \tag{328}$$

in the system's inertial range, where ε is the mean energy dissipation rate of the system. This result is especially important, as it is an analytical result derived directly from the Navier–Stokes equations. Therefore, it is able to provide direct evidence for the energy cascade and the resulting anomalous energy dissipation rate in the vanishing viscosity limit. In the case of developed turbulence, where all of the assumptions are met, the 4/5-th law also acts as a benchmark to check whether the simulation produces physically meaningful turbulence. Previous numerical experiments have shown a reasonable agreement between the slope of the 3rd-order structure function of the ground-truth simulation and the expected slope predicted by the 4/5-th law at later times. However, for the timescales considered in this paper, the turbulence is not yet developed and is highly intermittent. Moreover, the resolution at 128^3 is still rather low. Therefore there is no reason to expect the slope of the structure function to exactly follow the 4/5-th law. Nevertheless, it is interesting to examine if the flow generated by GenCFD is able to match the behavior of the 3rd-order structure function of the ground-truth distribution.

In Fig. SI 5 on the left, we visualize the 3rd-order structure function $S^3_{\parallel,r}$ of the ground truth, the unprocessed output from GenCFD, and the output from GenCFD after projecting it onto divergence-free vector fields with a Leray projection. The general shape of the structure function seems to agree well. However, GenCFD underestimates its magnitude. Nonetheless, GenCFD seems to be able to reproduce the general 2-point statistics of the flow as measured by this structure function. It is of interest now, though, to investigate whether the discrepancy stems from insufficient training or from a fundamental restriction in the model. Fig. SI 5 on the right visualizes how the relative error of the 3rd-order structure function's slope changes over the course of training the diffusion model. A clear algebraic convergence can be observed leading to the conclusion that there is no fundamental flaw in the model and, like for other numerical methods, more compute implies more accurate results also in the case of GenCFD.

Thus, these results clearly establish that not only is GenCFD able to match the 1-point statistics of the ground-truth simulated fluid flow, but also much finer 2-point statistics. Given the limited numerical resolution of the ground-truth flow, the precise agreement with existing physical and empirical theories is hard to expect. However, the observed results augur well for GenCFD to approximate finer statistical quantities of fully-developed turbulent fluid flow, once it is successfully trained on simulated data at even higher resolutions.

SI 5 Supplementary Figures

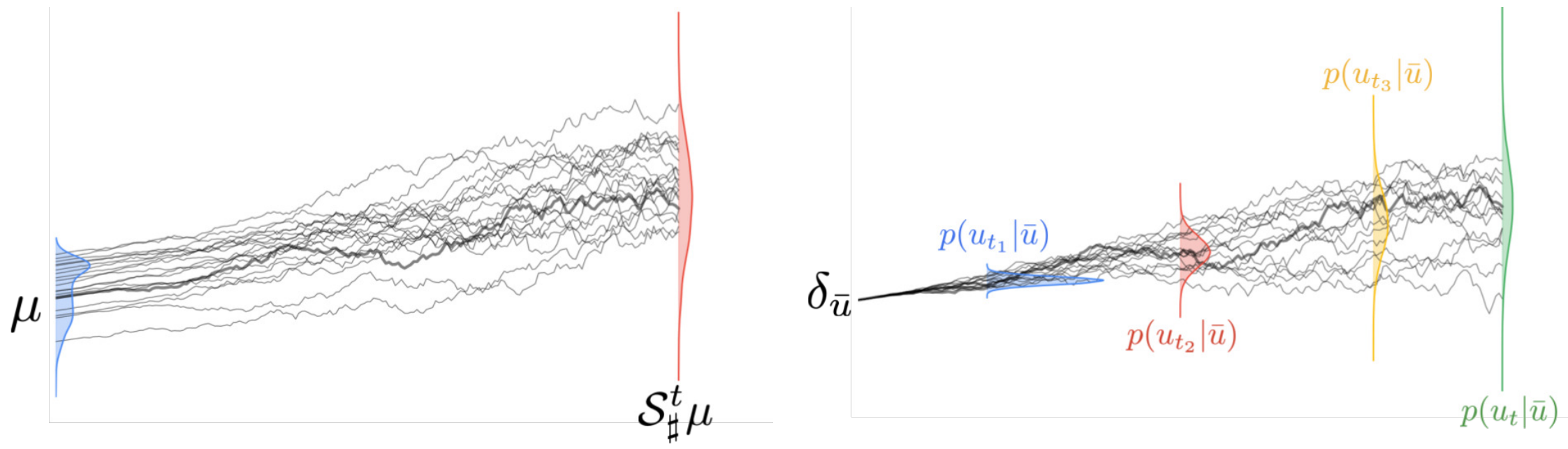

Figure SI 1: Schematic of the train/testing task. Left: Typical evaluation pipeline of diffusion models, for a given distribution μ and evolved samples that results in a push-forward distribution $\mathcal{S}_\sharp\mu$. Right: Schematic for the testing task in this manuscript. For an initial condition $\bar{u}$ we compute the conditional distribution at different times. Here we train the network on a *single* trajectory per condition, as shown in bold.

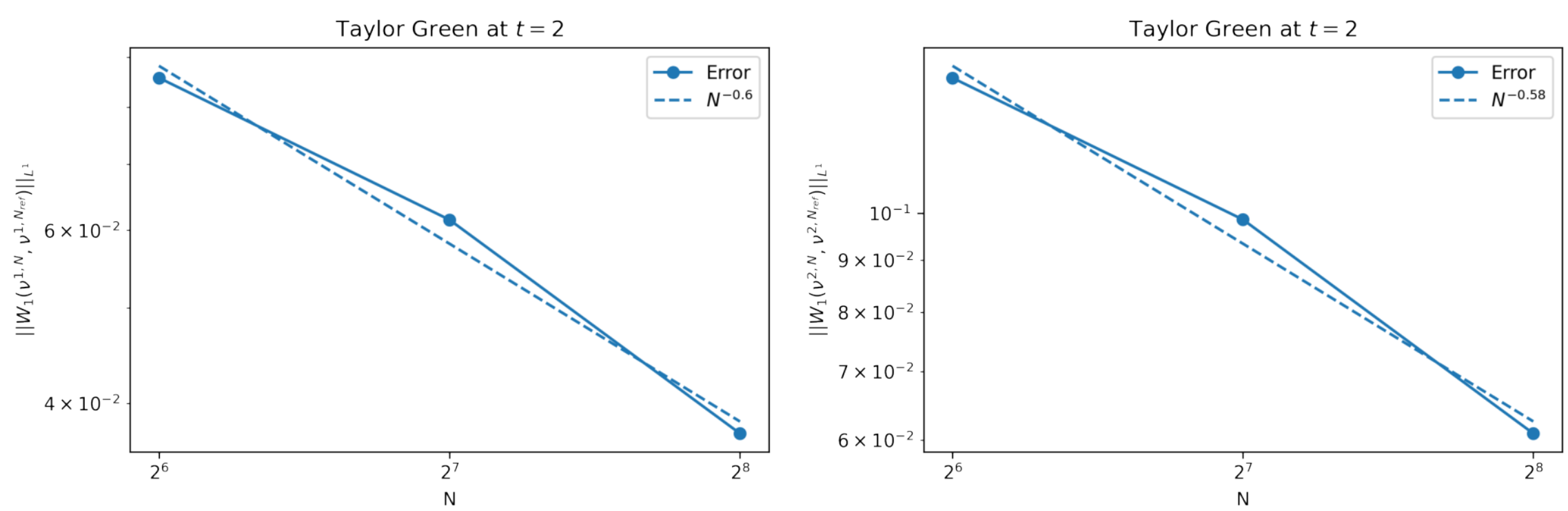

Figure SI 2: Statistical convergence of the ground truth under mesh refinement. The convergence is measured in the 1-Wasserstein metric for the first correlation marginal $\nu^{1,N}$ (left) and the second correlation marginal $\nu^{2,N}$ (right) for the Taylor–Green vortex at time $t = 2$. A simulation with mesh resolution $N = 512$ was used as a reference solution. Source data are provided as a Source Data file.

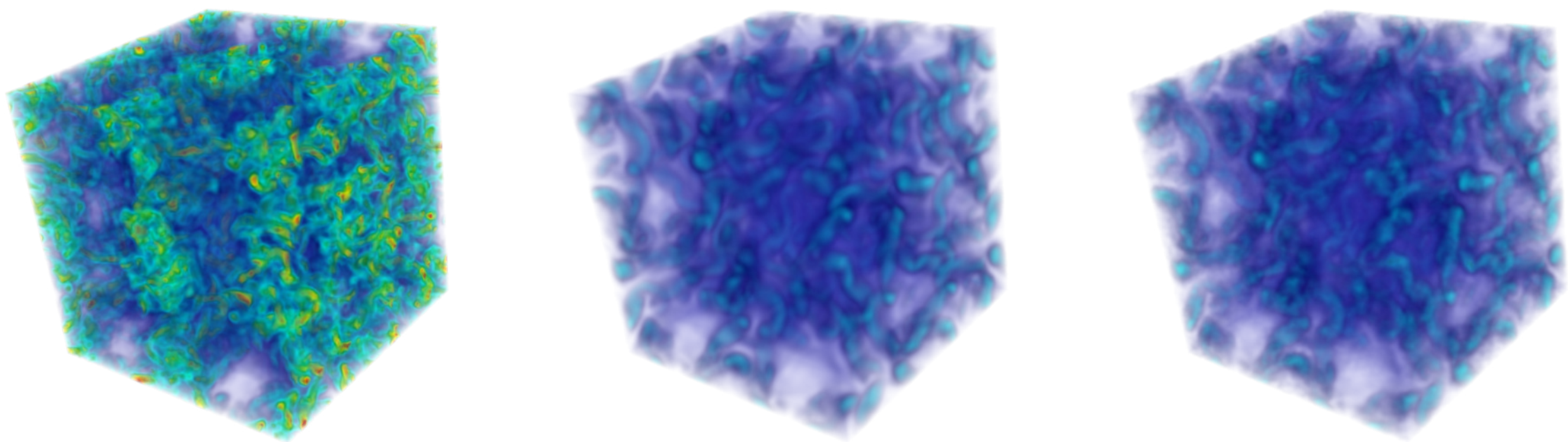

Figure SI 3: Visualization of the vorticity of the Taylor–Green vortex at $T = 2.0$. The results are produced with the ground-truth simulation, CRPS (1), and CRPS (4) (individual samples from left to right). The CRPS training essentially adds incoherent noise on top of the mean of the distribution.

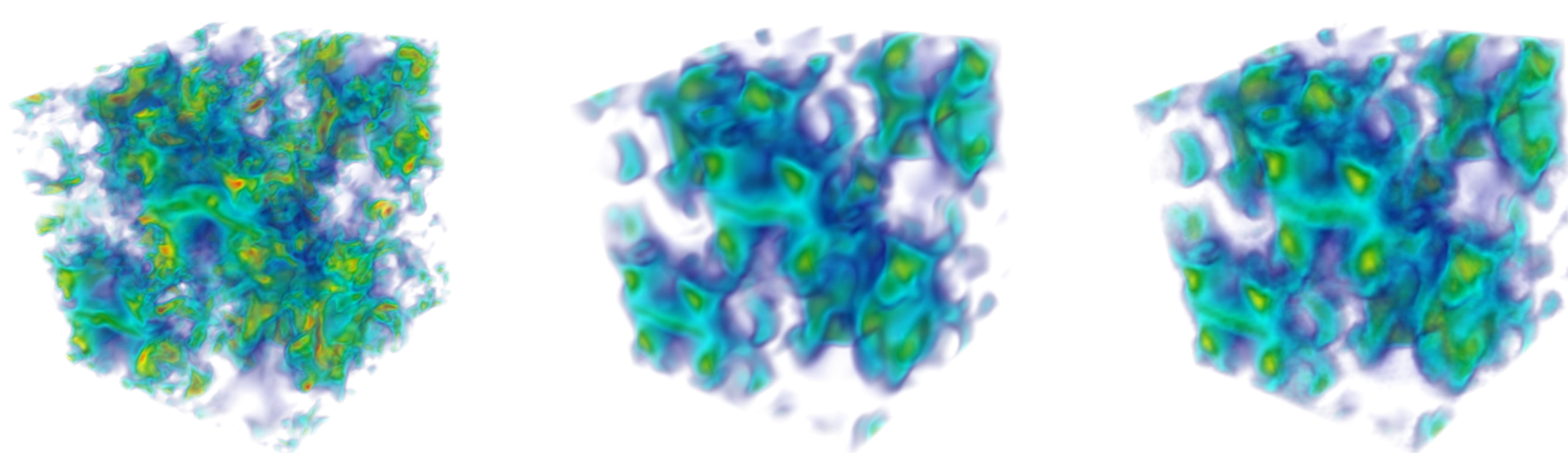

Figure SI 4: Visualization of the velocity u_x of the Taylor–Green vortex at $T = 2.0$. The results are produced with the ground-truth simulation, CRPS (1), and CRPS (4) (individual samples from left to right). The CRPS training essentially adds incoherent noise on top of the mean of the distribution.

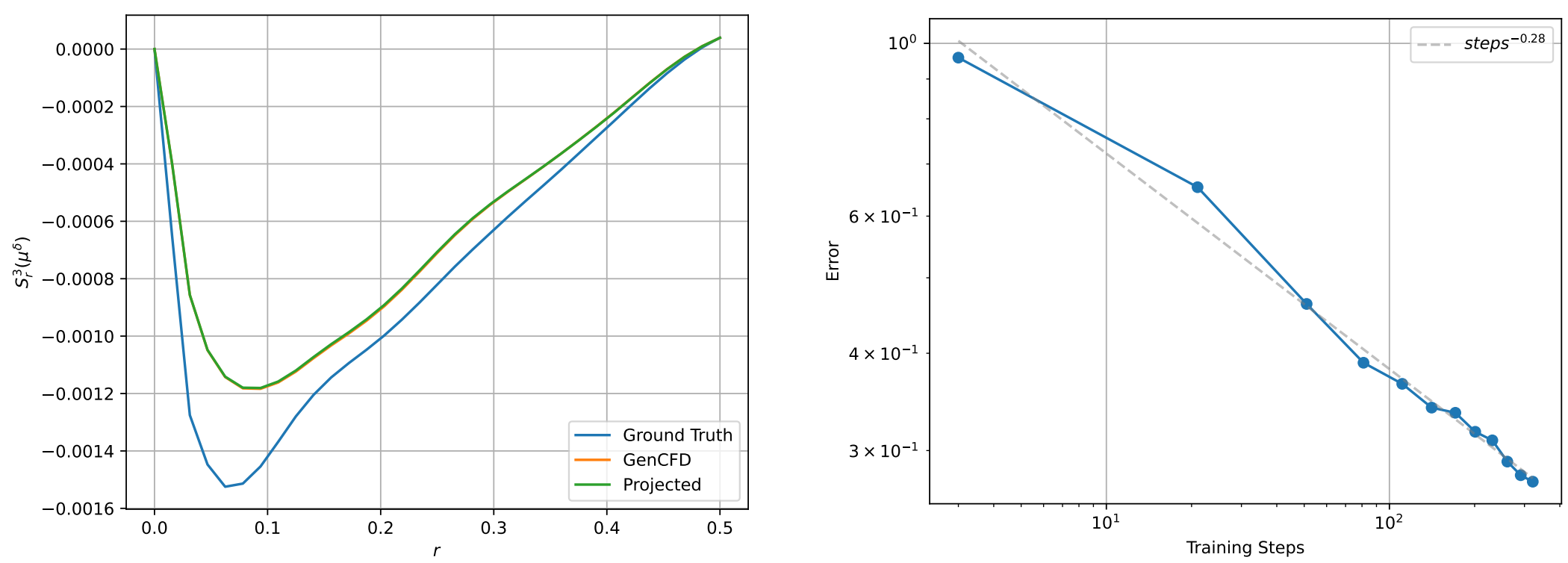

Figure SI 5: Third-order structure function of the Taylor–Green vortex at $T = 2.0$ and convergence of its slope in the inertial range towards the ground truth. The third order structure functions produced with the ground truth, GenCFD, and including a projection are plotted over r (left). The error of the slope of the structure functions in the inertial range is plotted over the number of training steps $\times 10^3$ (right). The ground truth is provided by the Monte Carlo simulation. Source data are provided as a Source Data file.

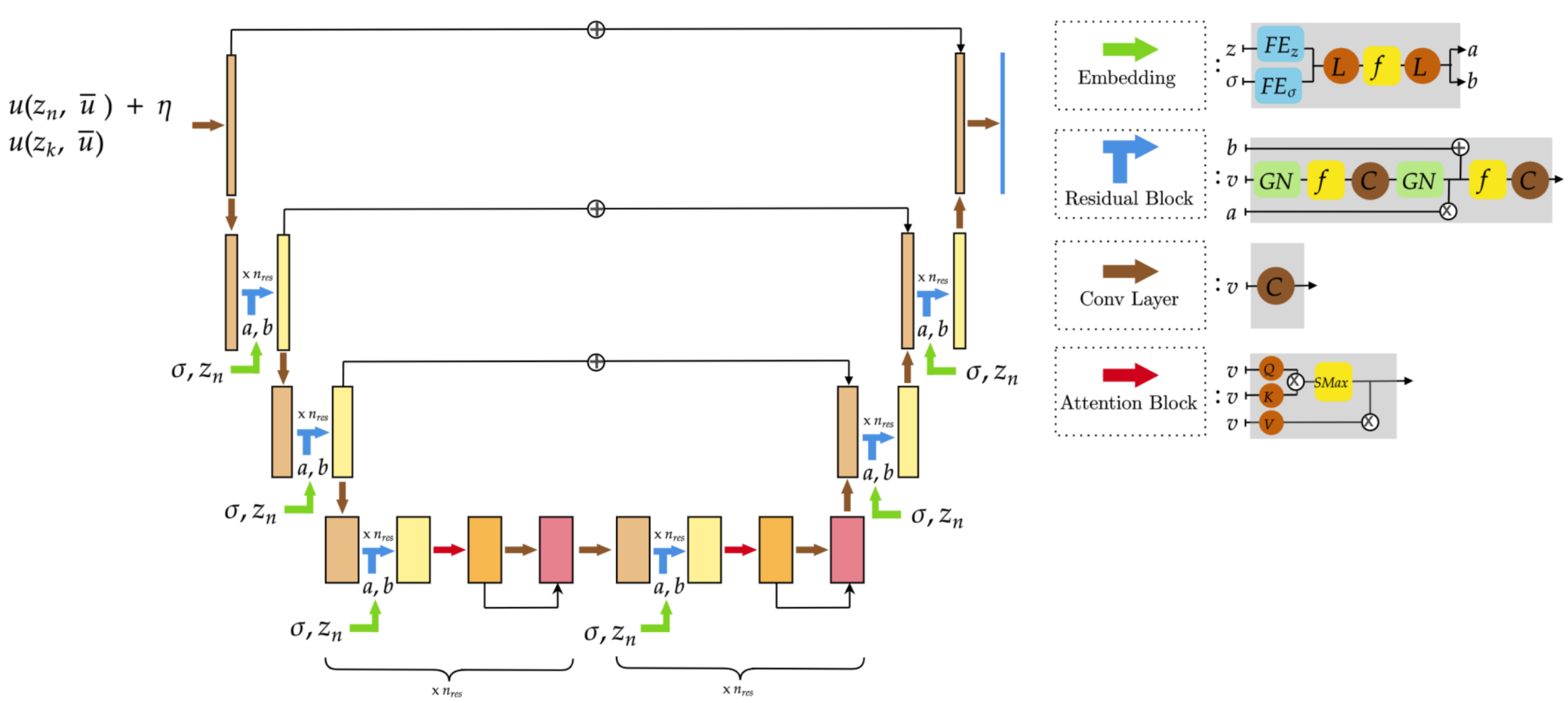

Figure SI 6: Schematic of the architecture of the UViT neural network. The architecture is used as the model for the denoiser in GenCFD. A detailed description of the model is provided in Sec. SI 2.

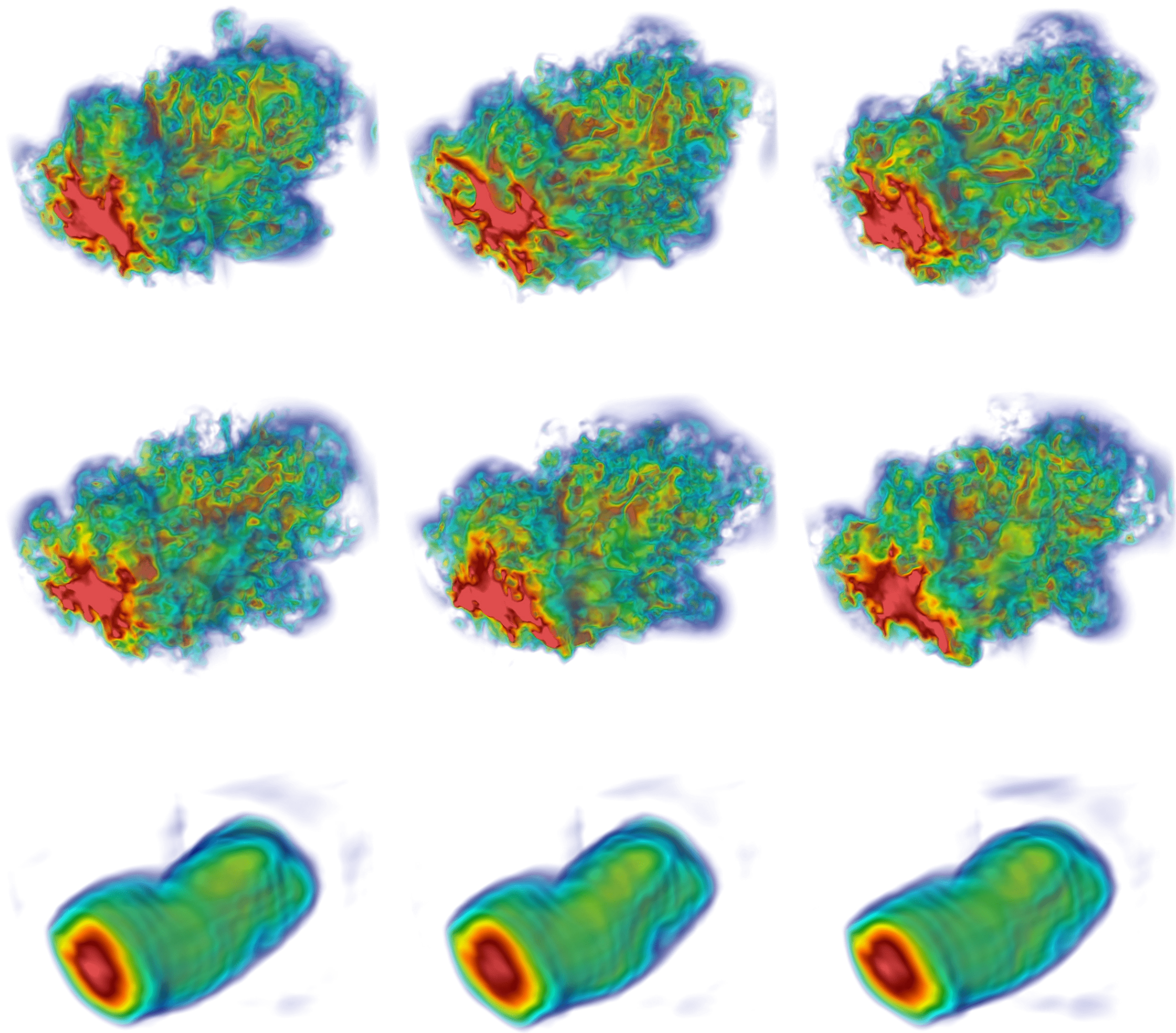

Figure SI 7: Visualization of pointwise kinetic energy for three randomly generated samples for the cylindrical shear flow experiment at time $T = 1$. Ground truth (top row), GenCFD (middle row) and C-FNO (bottom row). The colormap for all the figures ranges from 0.6 (dark blue) to 1.7 (dark red).

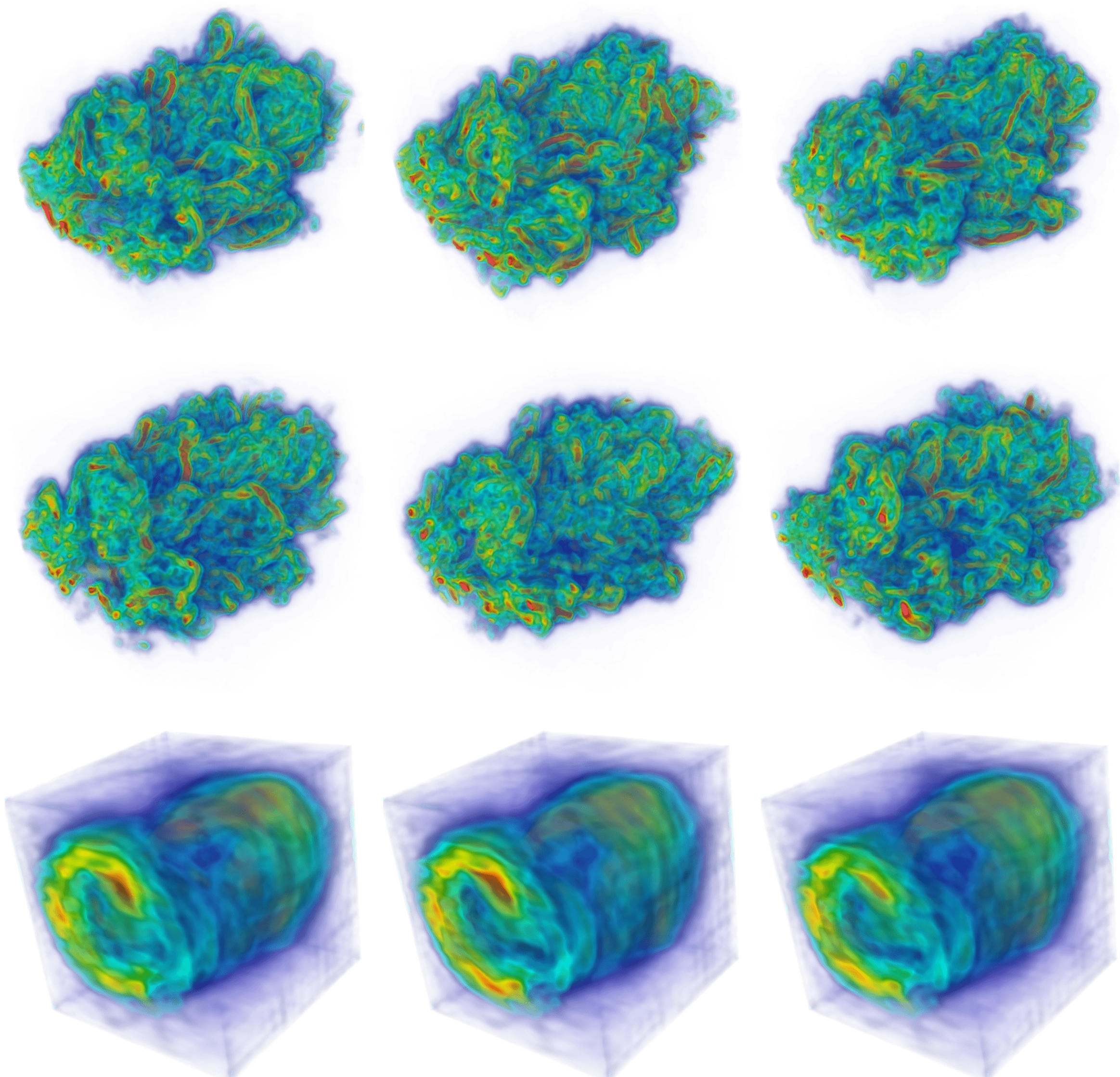

Figure SI 8: Visualization of pointwise vorticity intensity for three randomly generated samples for the cylindrical shear flow experiment at time $T = 1$. Ground truth (top row), GenCFD (middle row) and C-FNO (bottom row). The colormap for the top and middle rows ranges from 10^{-4} (dark blue) to 40.0 (dark red), whereas for the bottom row, it ranges from 0.5 (filtering the low values) to 19.5.

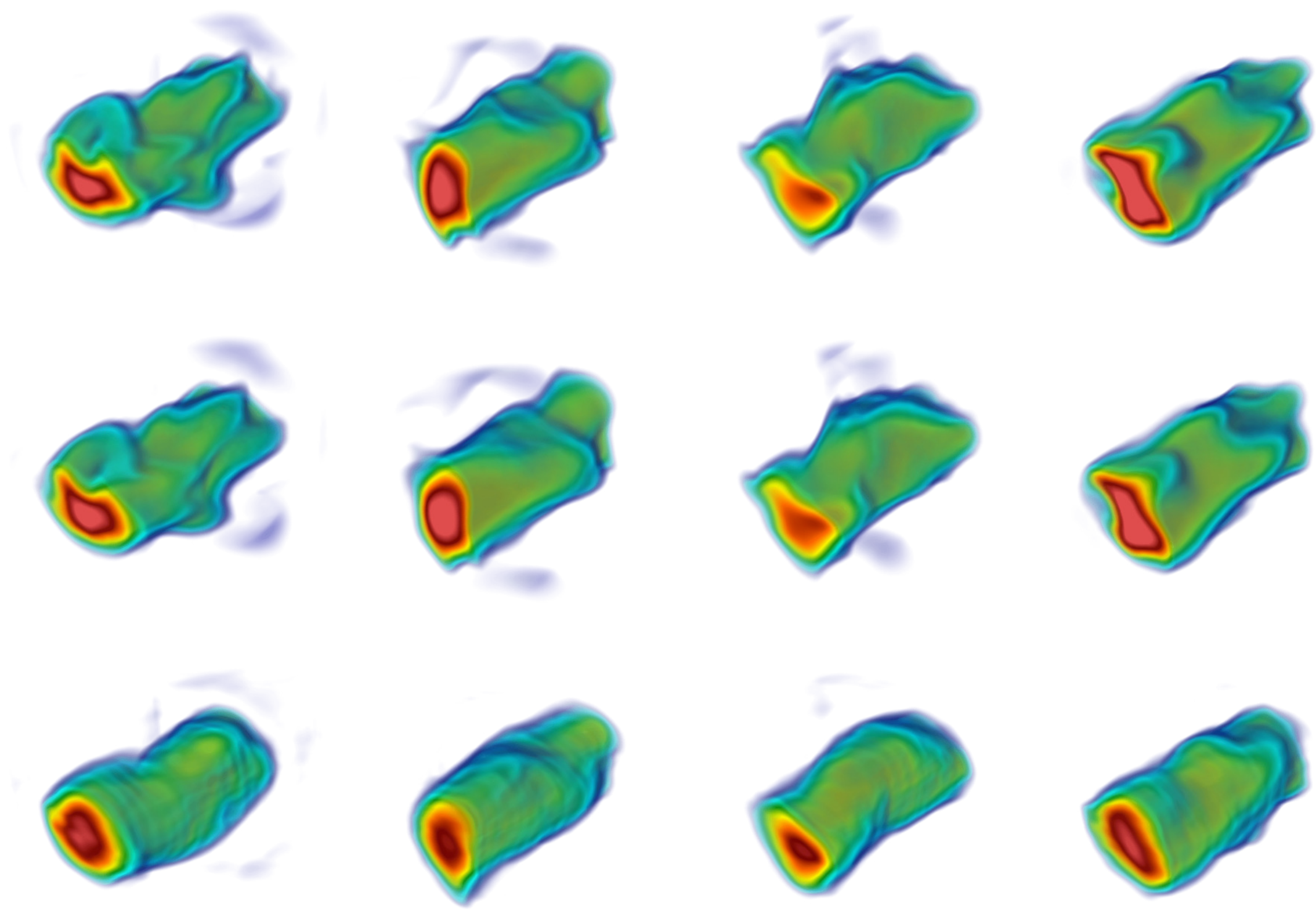

(a) $\bar{u} = \bar{u}^1$ **(b)** $\bar{u} = \bar{u}^2$ **(c)** $\bar{u} = \bar{u}^3$ **(d)** $\bar{u} = \bar{u}^4$

Figure SI 9: Visualization of the mean of the pointwise kinetic energy for the cylindrical shear flow experiment at time $T = 1$, for four different initial distributions. Data generated by the ground truth (top row), GenCFD (middle row) and C-FNO (bottom row). The colormap for all the figures ranges from 0.6 (dark blue) to 1.7 (dark red).

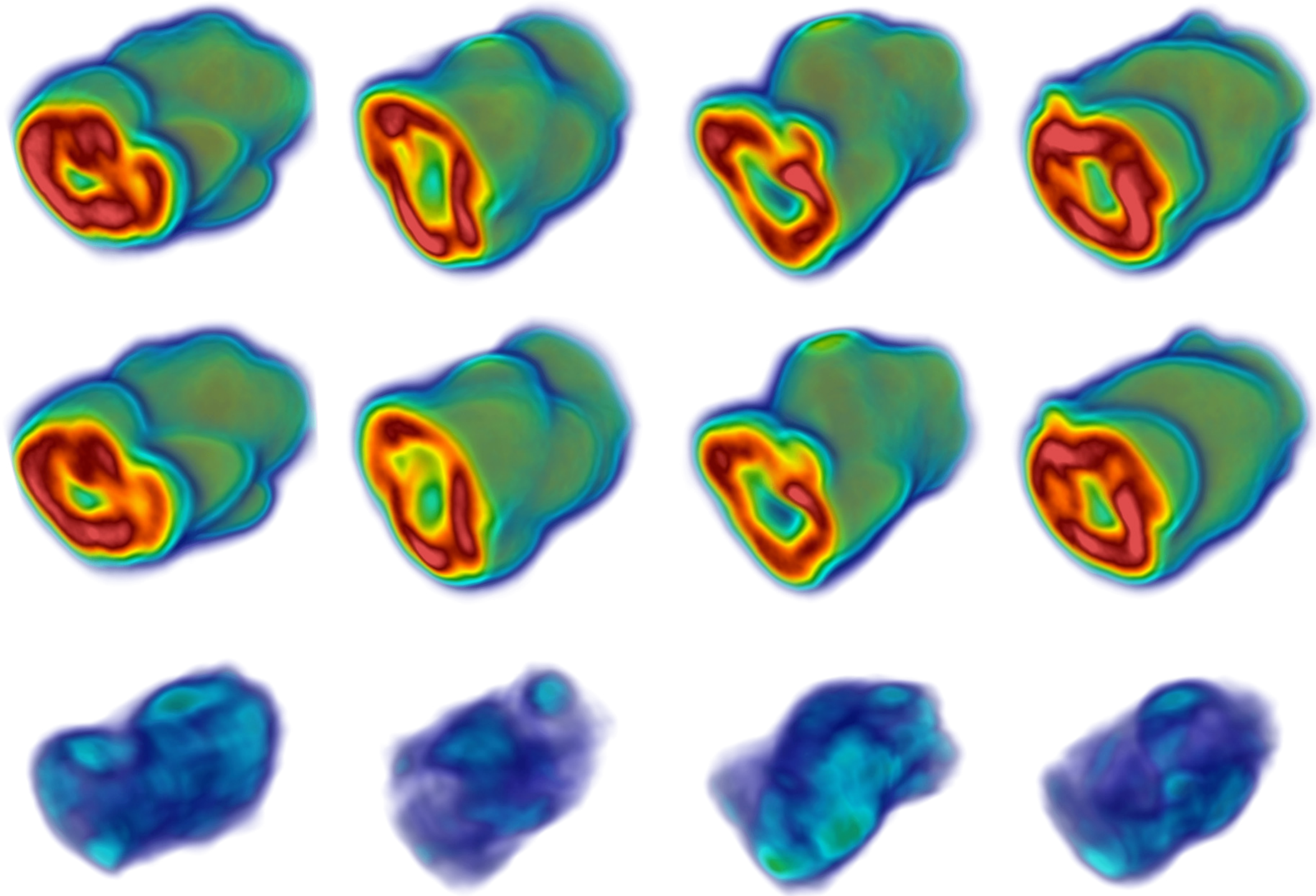

(a) $\bar{u} = \bar{u}^1$ (b) $\bar{u} = \bar{u}^2$ (c) $\bar{u} = \bar{u}^3$ (d) $\bar{u} = \bar{u}^4$

Figure SI 10: Visualization of the standard deviation of the kinetic energy for the cylindrical shear flow experiment at time $T = 1$, for four different initial distributions. Data generated by the ground truth (top row), GenCFD (middle row) and C-FNO (bottom row). The colormap for the top and middle rows ranges from 0.05 (dark blue) to 0.65 (dark red), whereas for the bottom row, it ranges from 0.05 to 0.25.

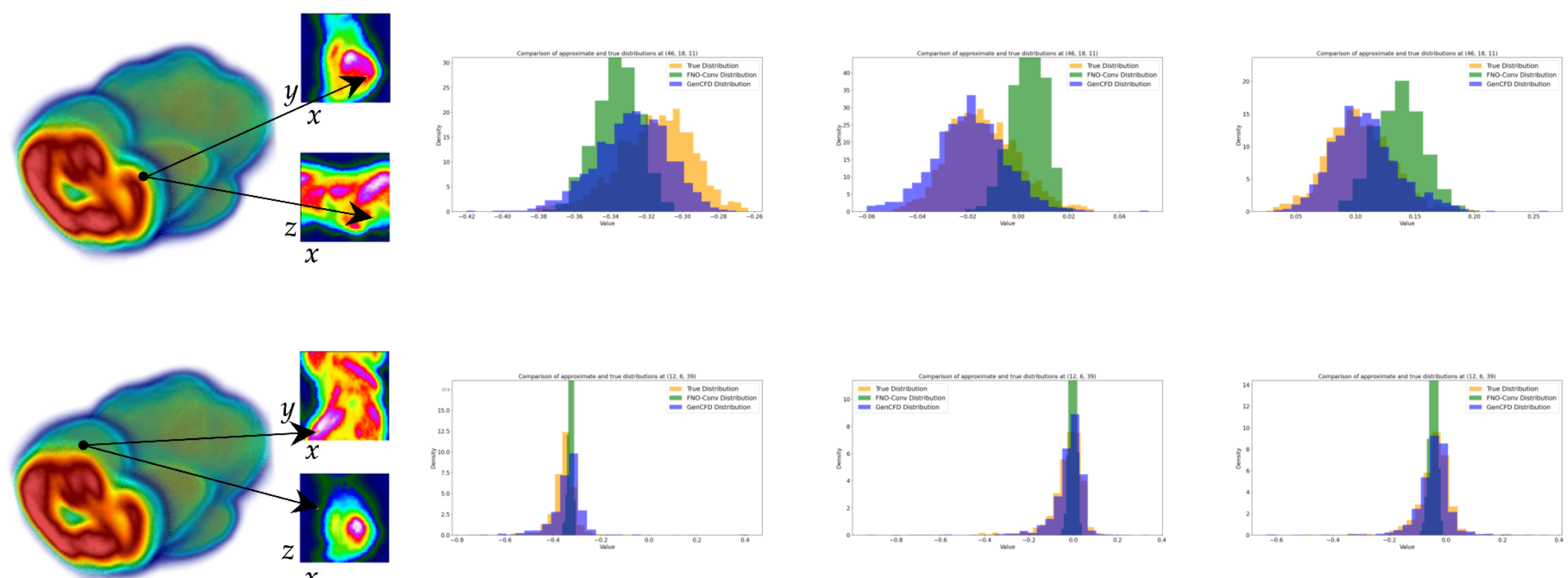

(b) u_x **(c)** u_y **(d)** u_z

(a) Points

Figure SI 11: Histograms of the point PDFs, at two different points, of all velocity components for the cylindrical shear flow experiment at time $T = 1$. Results are shown for the ground truth, GenCFD and C-FNO. **(a):** Location of the two points in the computational domain. **(b,c,d):** PDFs for u_x-, u_y-, and u_z-velocity, respectively, produced with ground truth, GenCFD and C-FNO. Source data are provided as a Source Data file.

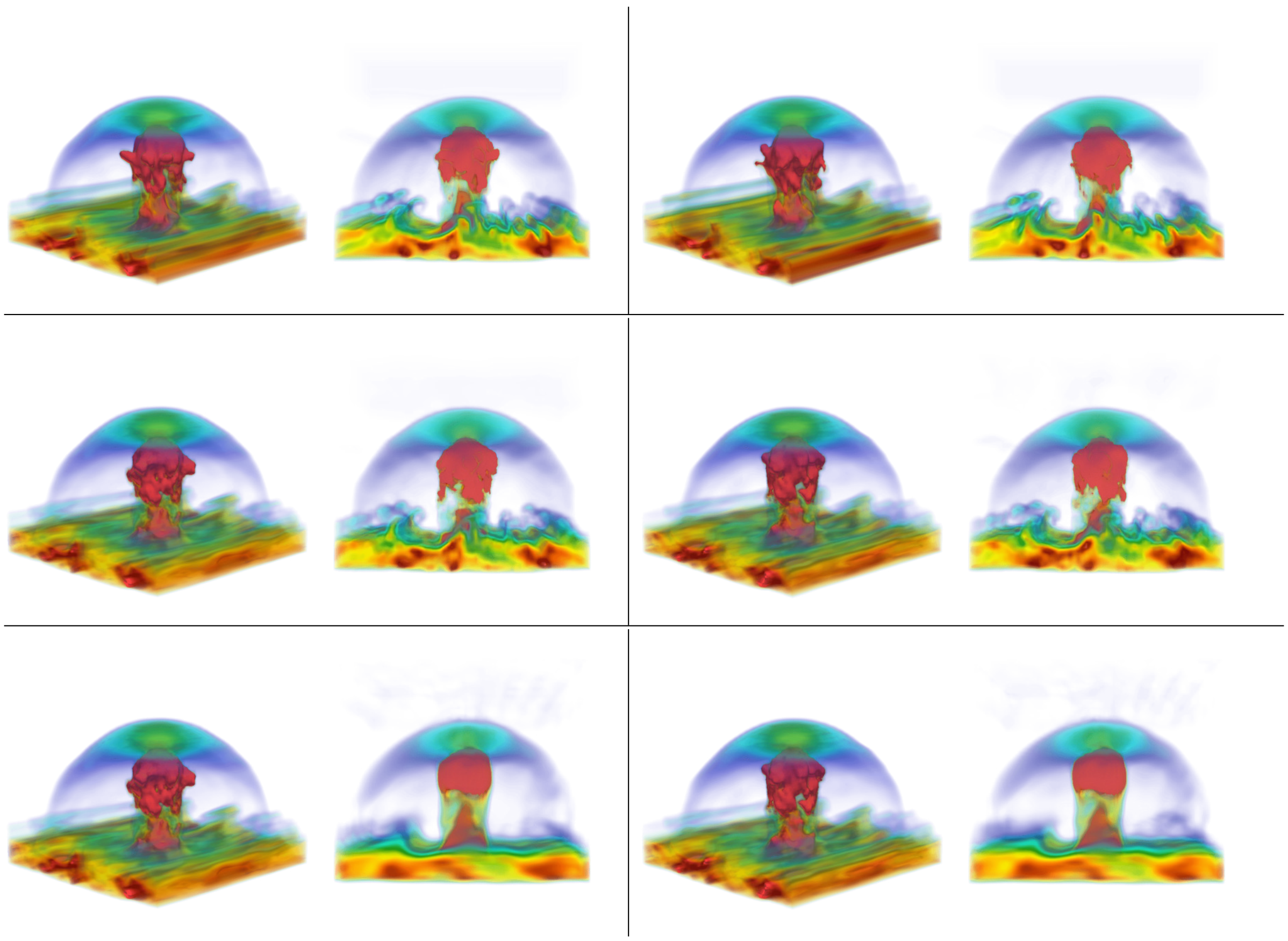

Figure SI 12: Visualization from two different angles of the density at time $T = 0.06$ for the cloud-shock interaction experiment. Results are shown for the ground truth (top row), GenCFD (middle row) and C-FNO (bottom row). The colormap for all the figures ranges from 3.90 (dark blue) to 6.35 (dark red).

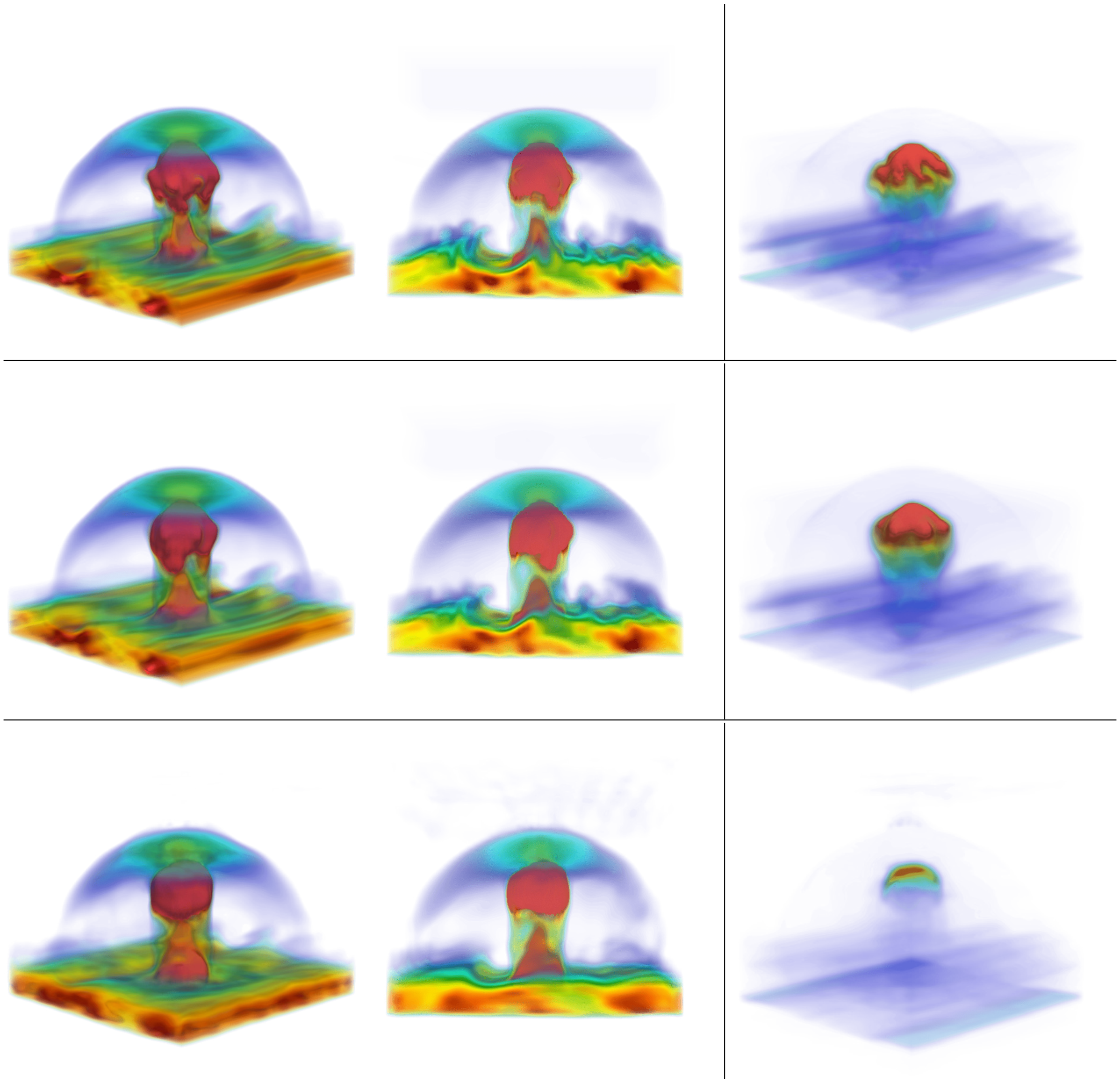

Figure SI 13: Visualization of the mean and the standard deviation of the density at time $T = 0.06$ for the cloud-shock interaction experiment. Two different angles of the mean are shown in left and center columns and the standard deviation is shown in the right column. Results are shown for the ground truth (top row), GenCFD (middle row) and C-FNO (bottom row). The colormap for the figures representing the means ranges from 3.90 (dark blue) to 6.35 (dark red). The colormap for the figures representing the standard deviations ranges from 0.0075 to 3.0 for the top and middle rows, and from 0.0075 to 2.0 for the bottom row.

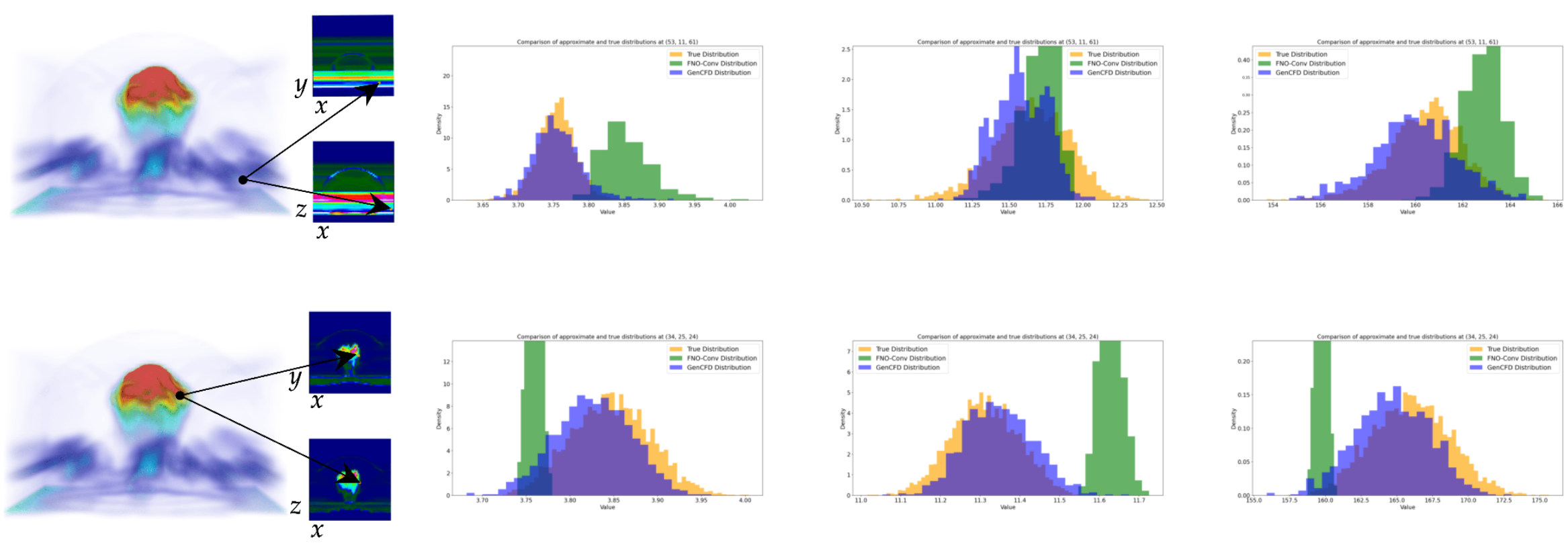

(b) ρ **(c)** u_x **(d)** p

(a) Points

Figure SI 14: Histograms of the point PDFs at two different points of the density, x-velocity and pressure, at time $T = 0.06$ of the cloud-shock interaction experiment. Results are shown for the ground truth, GenCFD and C-FNO. **(a):** Location of the two points in the computational domain. **(b,c,d):** PDFs for the density, u_x-velocity, and pressure, respectively, produced with ground truth, GenCFD and C-FNO. Source data are provided as a Source Data file.

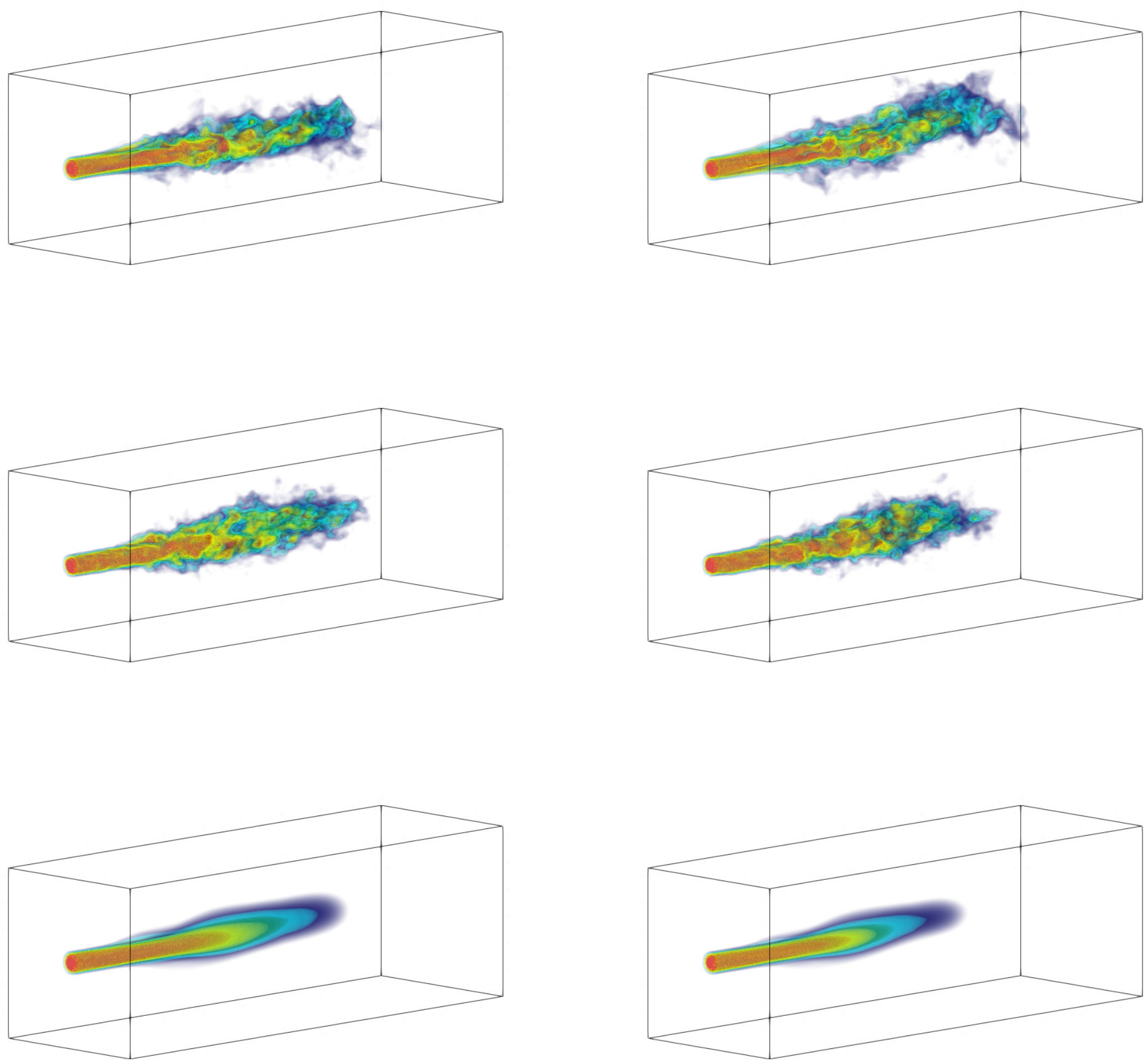

Figure SI 15: Visualization of two random samples, for the same injection velocity, of the pointwise kinetic energy at time $T = 130$ for the nozzle flow experiment. Two different sample results (column-wise) are shown for the ground truth (top row), GenCFD (middle row) and UViT (bottom row).

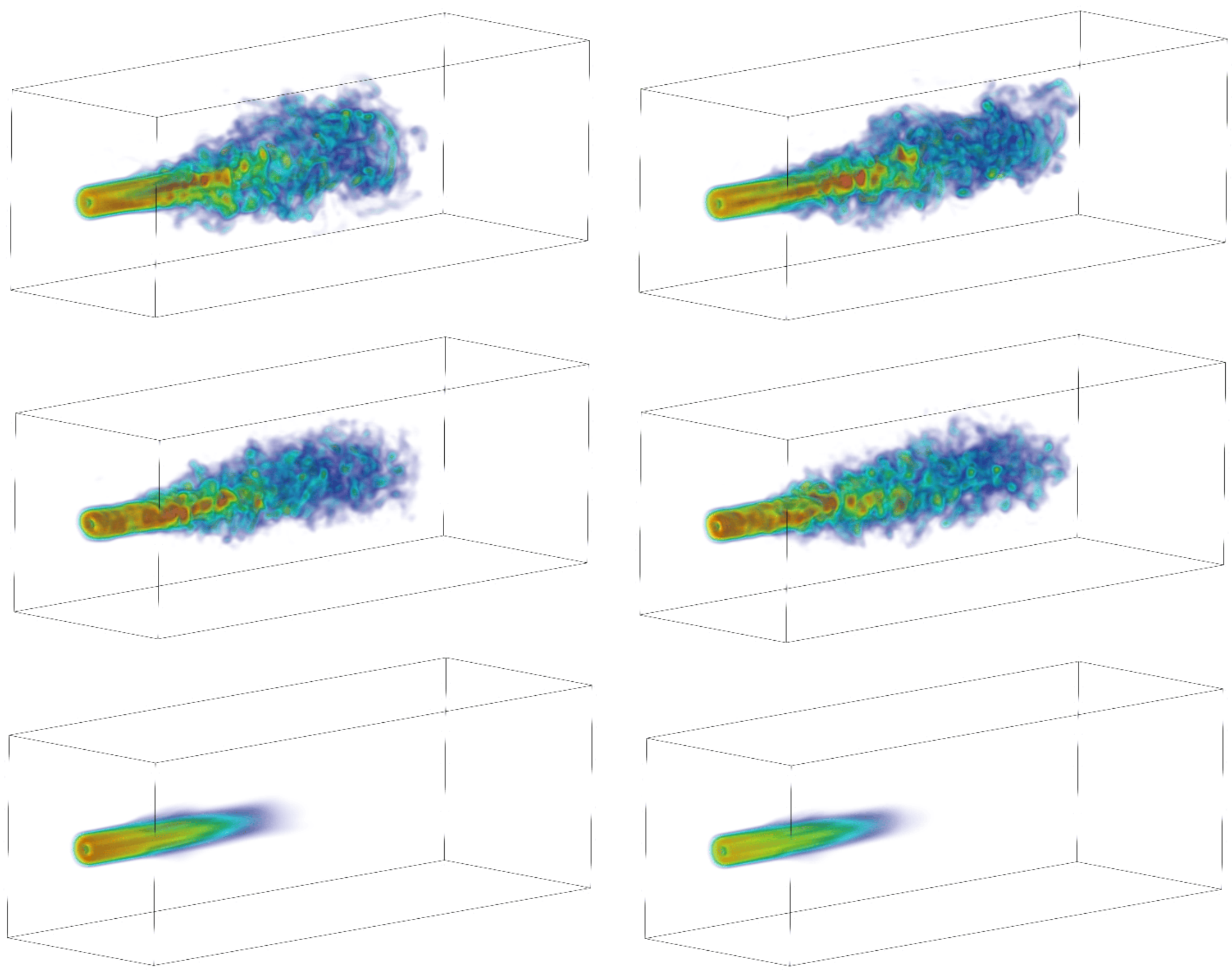

Figure SI 16: Visualizations of two random samples, for the same injection velocity, of the pointwise vorticity intensity at time $T = 130$ for the nozzle flow experiment. Two different sample results are shown (column-wise) for the ground truth (top row), GenCFD (middle row) and UViT (bottom row).

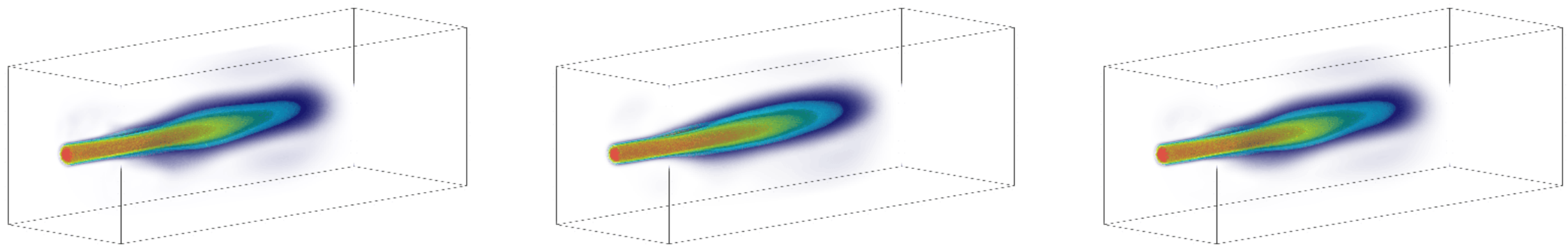

Figure SI 17: Visualization of the mean of the pointwise kinetic energy at time $T = 130$ for the nozzle flow experiment. Results are shown for the ground truth (left), GenCFD (center) and UViT (right).

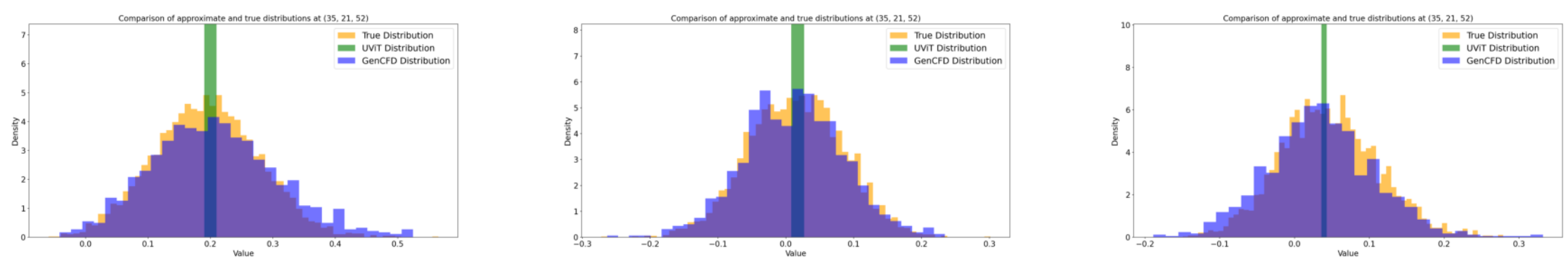

Figure SI 18: Histograms of the point PDFs of the three velocity components at the spatial point $(0.547, 0.328, 0.271)$ at time $T = 130$ for the nozzle flow experiment. Results are shown for the ground truth, GenCFD and UViT, respectively. From left to right: PDFs for u_x-, u_y-, and u_z-velocity, respectively, produced with ground truth, GenCFD and UViT. Source data are provided as a Source Data file.

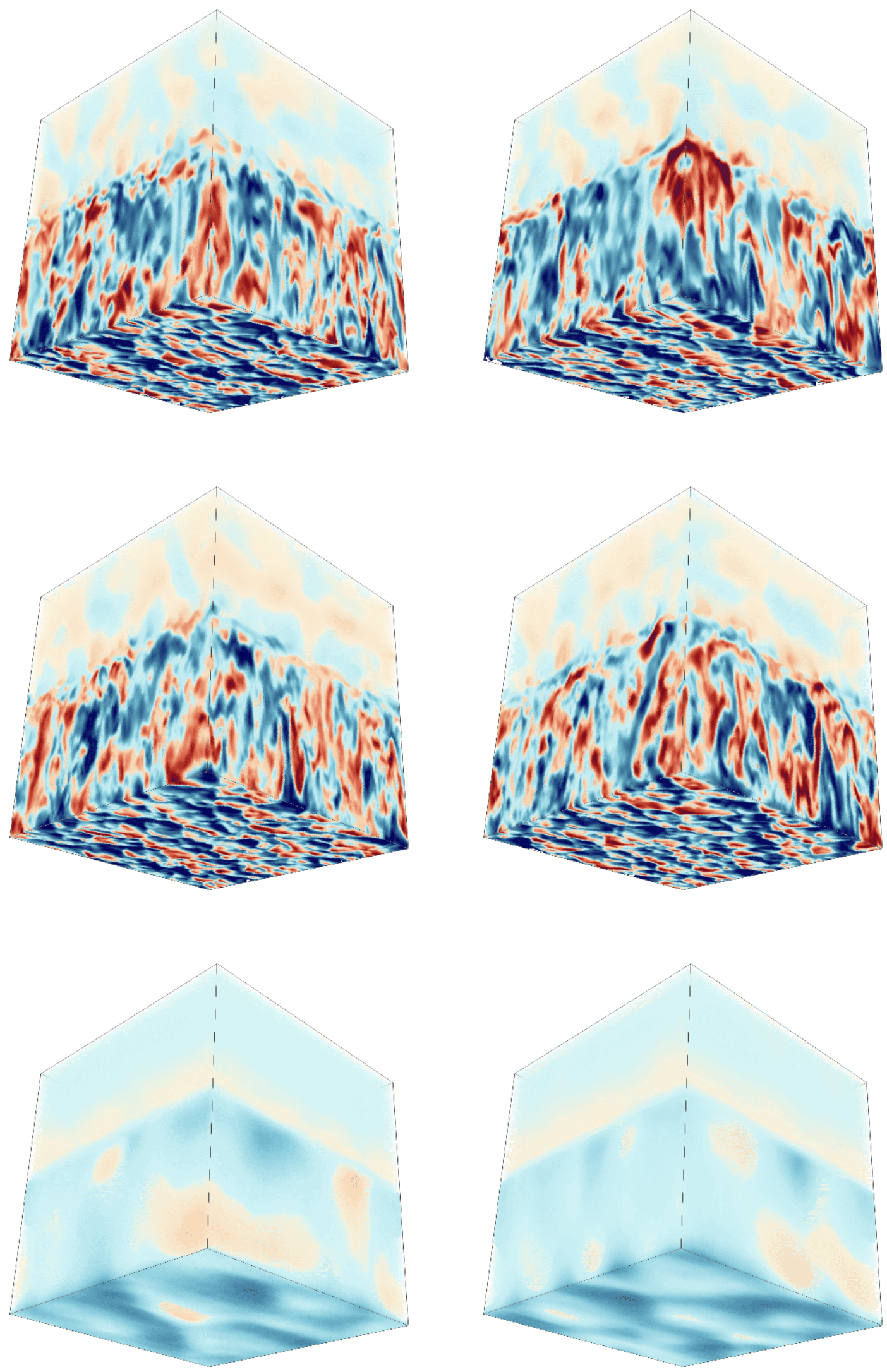

Figure SI 19: Visualization of two random samples, of the velocity component u_x at time $T = 2.4$ for the convective boundary layer experiment. Results of two samples (column-wise) are shown for the ground truth (top row), GenCFD (middle row) and UViT (bottom row).

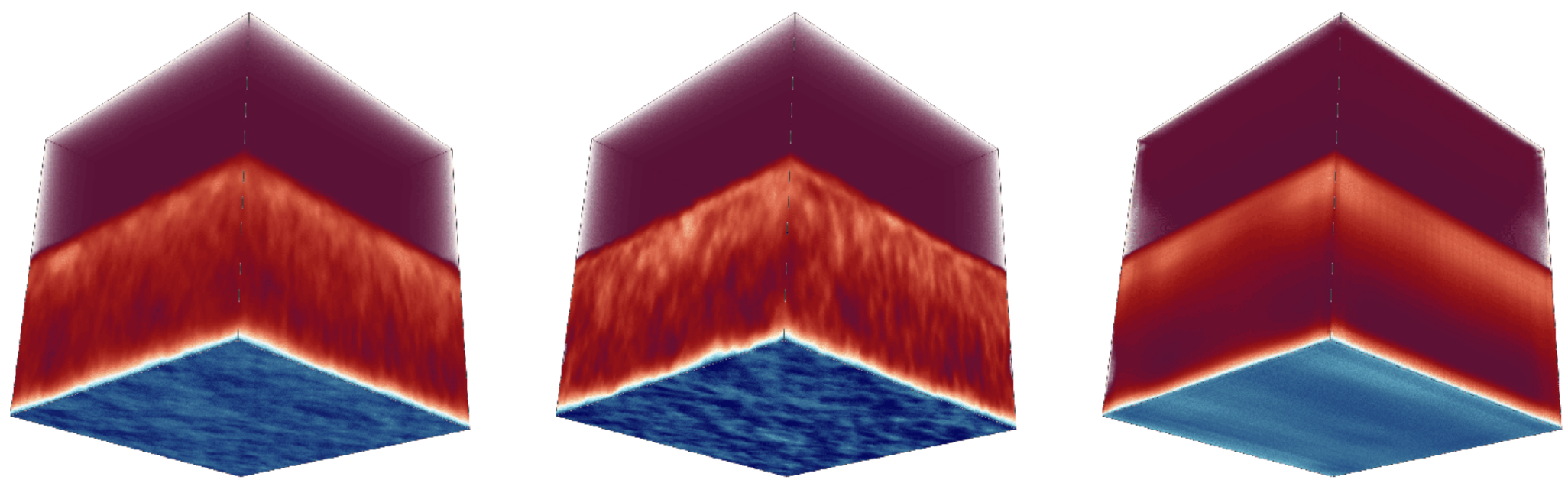

Figure SI 20: Visualization of the mean of the velocity component u_x at time $T = 2.4$ for the convective boundary layer experiment. Results are shown for the ground truth (left), GenCFD (center) and UViT (right).

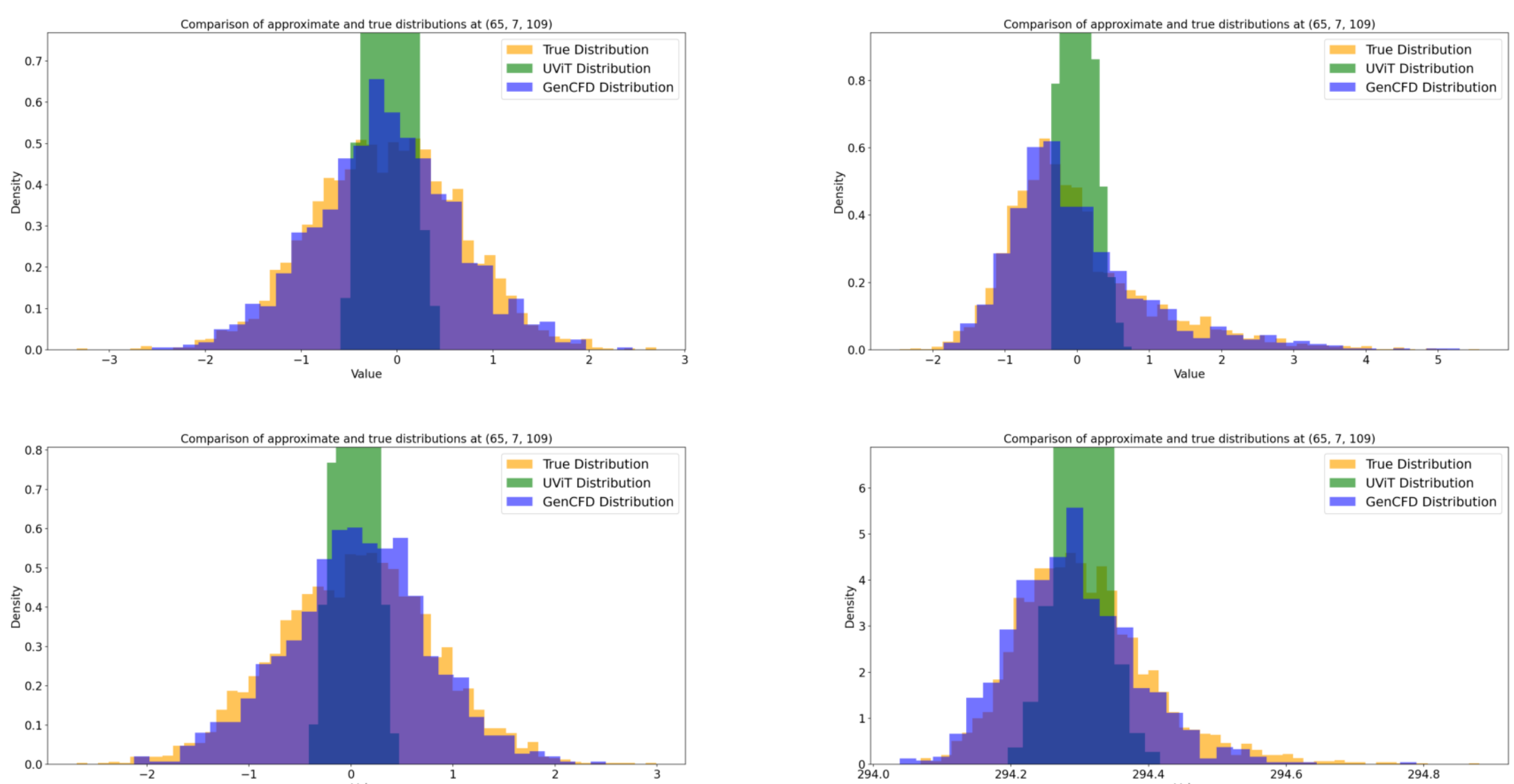

Figure SI 21: Histograms of the point PDFs of the temperature and the three velocity components at the spatial point $(0.508, 0.055, 0.852)$ at time $T = 2.4$ for the convective boundary layer experiment. Results are shown for the ground truth, GenCFD and UViT, respectively. From left to right and top to bottom: PDFs for the temperature and for u_x-, u_y-, and u_z-velocity, respectively, produced with ground truth, GenCFD and UViT. Source data are provided as a Source Data file.

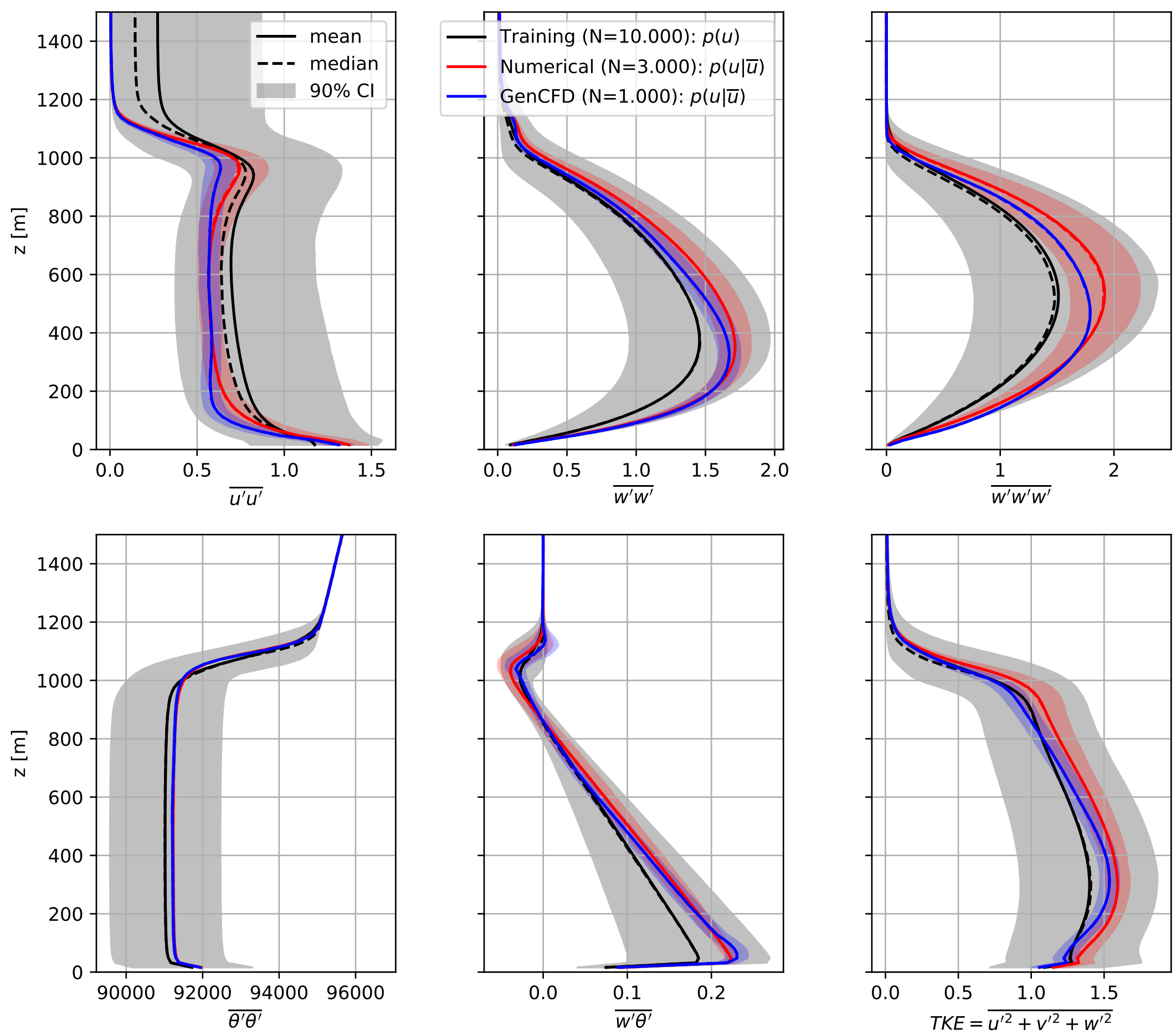

Figure SI 22: Profiles (horizontal statistics) for the convective boundary layer experiment for GenCFD and ground truth at final simulation time $T = 7200$ s. The distribution of (unconditional) training and validation data $p(u)$ is shown in gray. For one selected set of inputs $\overline{u}$, the conditional distribution $p(u|\overline{u})$ is shown in red for the numerical ground-truth model and in blue for samples generated with GenCFD. From left to right and top to bottom: x_1-velocity variance, vertical velocity variance, vertical velocity skewness, potential temperature variance, turbulent vertical flux of potential temperature, and turbulent kinetic energy.

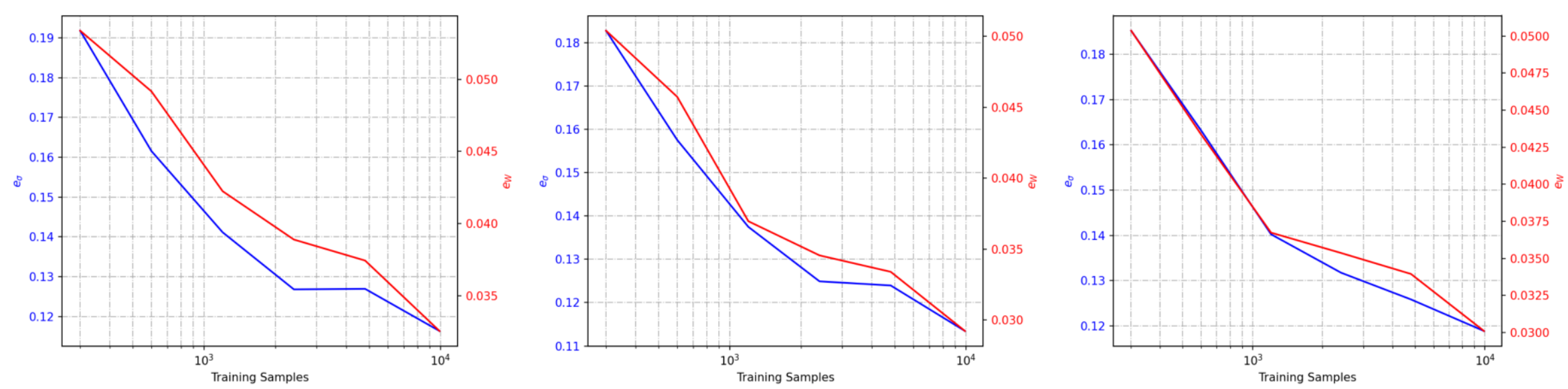

(a) u_x **(b)** u_y **(c)** u_z

Figure SI 23: Scaling of standard deviation and Wasserstein metric error of GenCFD vs. number of training samples for the cylindrical shear flow. The Wasserstein metric errors e_σ and e_W of GenCFD are plotted over the number of training samples. The errors are computed with respect to the velocities **(a):** u_x, **(b):** u_y, and **(c):** u_z for the cylindrical shear flow experiment. Source data are provided as a Source Data file.

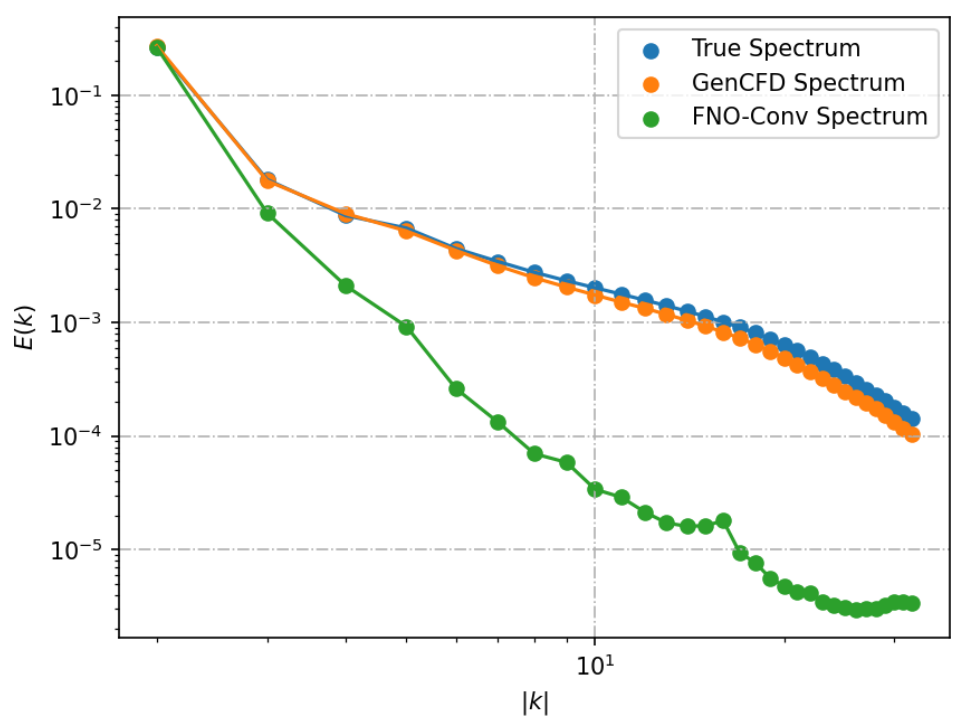

(a) Cylindrical shear flow

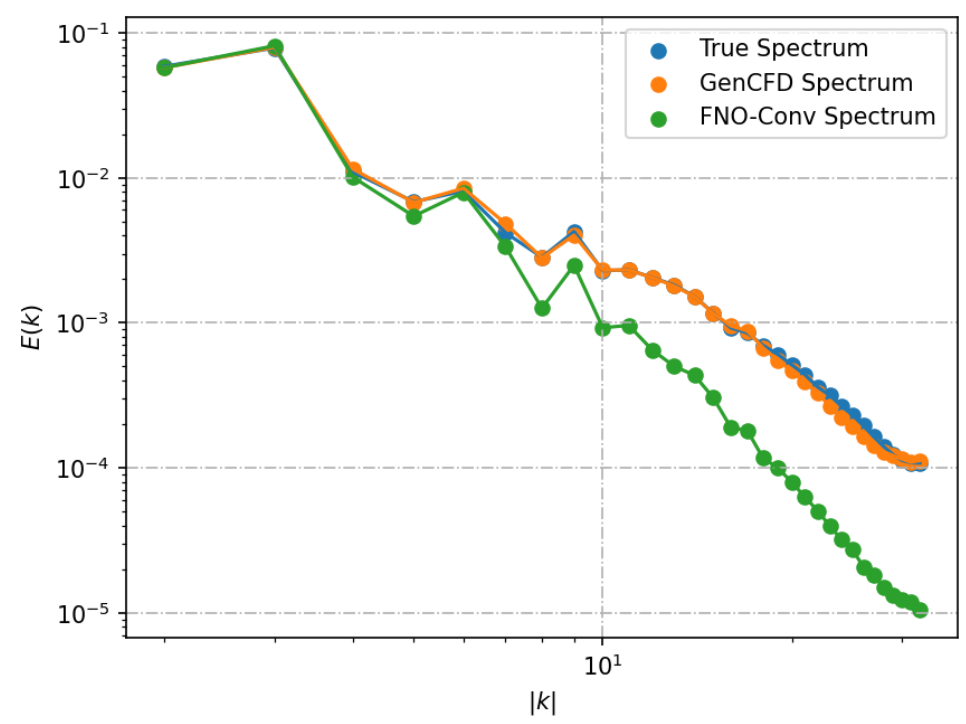

(b) Cloud-shock interaction

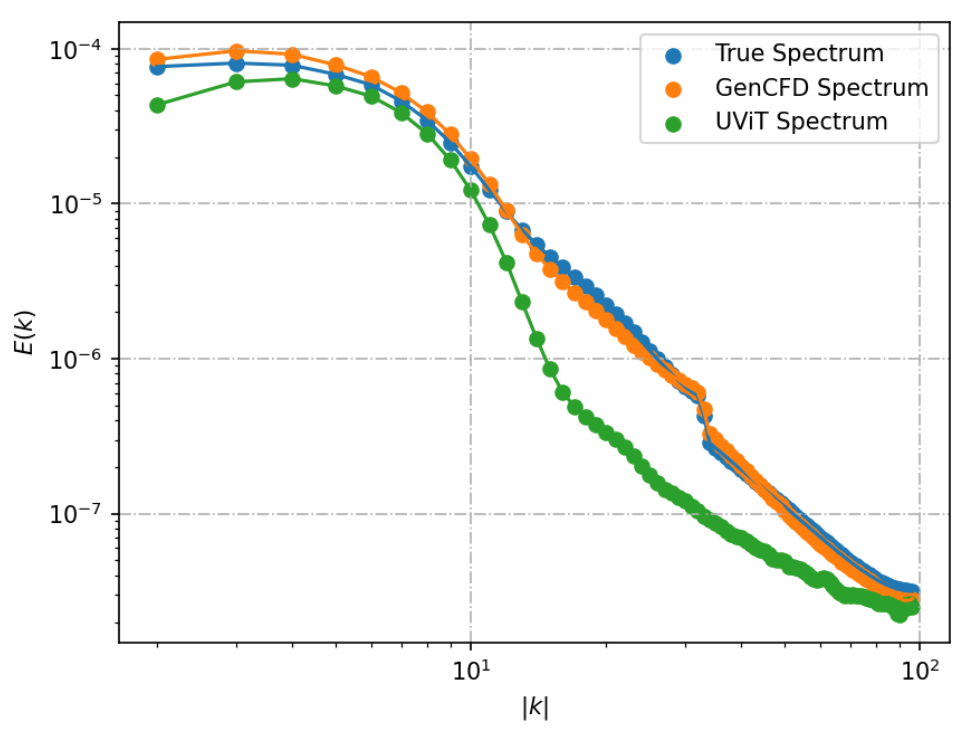

(c) Nozzle flow

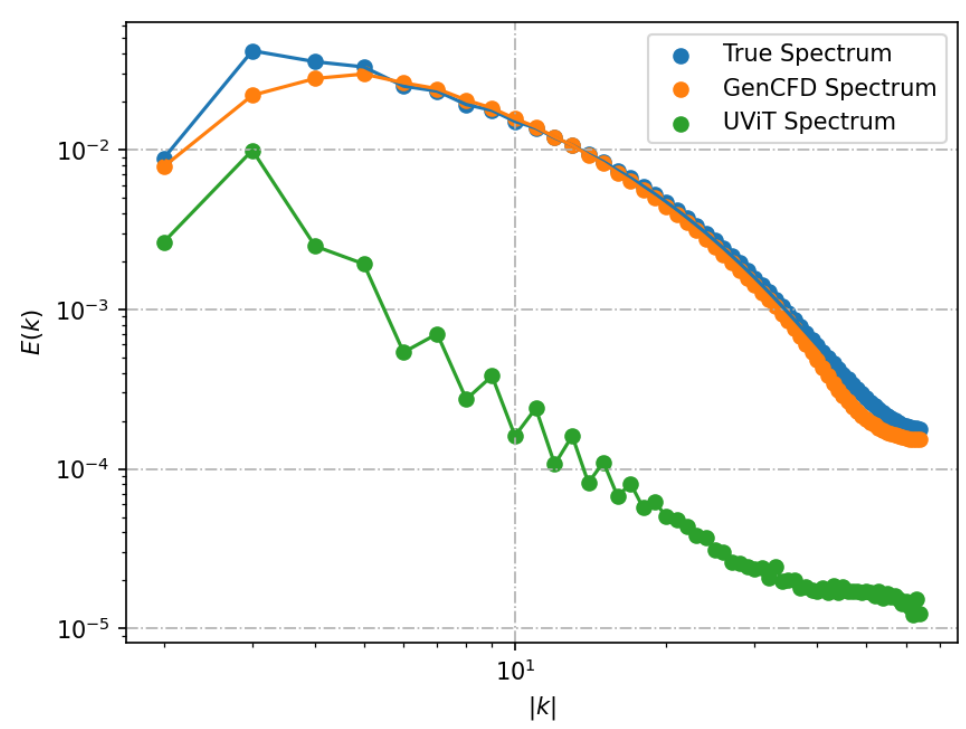

(d) Convective boundary layer

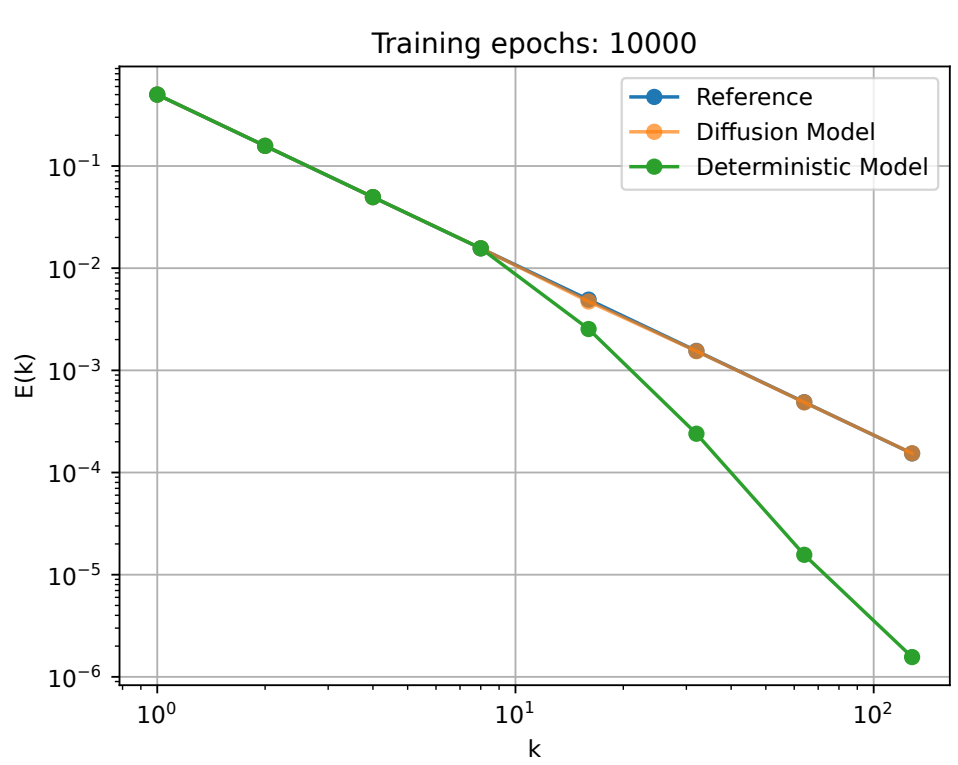

(e) Toy Problem #2

Figure SI 24: Energy spectra, generated by the ground truth, GenCFD and the best-performing baseline for four of the 3D datasets and the spectrum of Toy Model #2. (a): Cylindrical shear flow experiment, **(b):** Cloud-shock interaction, **(c):** Nozzle flow, **(d):** Convective boundary layer, and **(e):** Toy Problem #2. Note that for the cloud-shock interaction experiment, the spectrum of the z-velocity is shown. Source data are provided as a Source Data file.

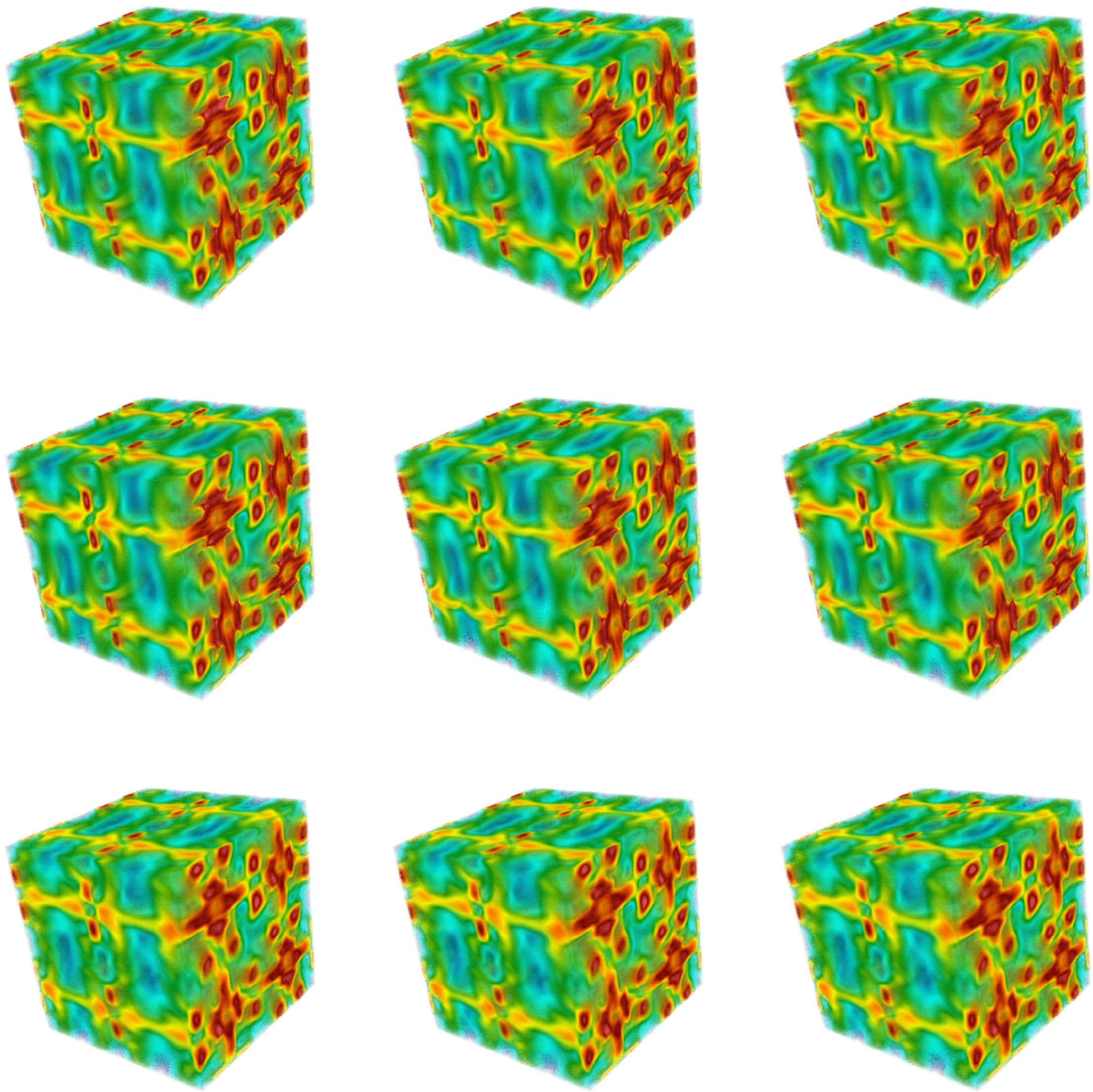

Figure SI 25: Visualization of pointwise kinetic energy for three randomly generated samples for the Taylor–Green vortex experiment at time $T = 0.8$. Results are shown for the ground truth (top row), GenCFD (middle row) and C-FNO (bottom row). The colormap for all the figures ranges from 0.0 (dark blue) to 1.0 (dark red).

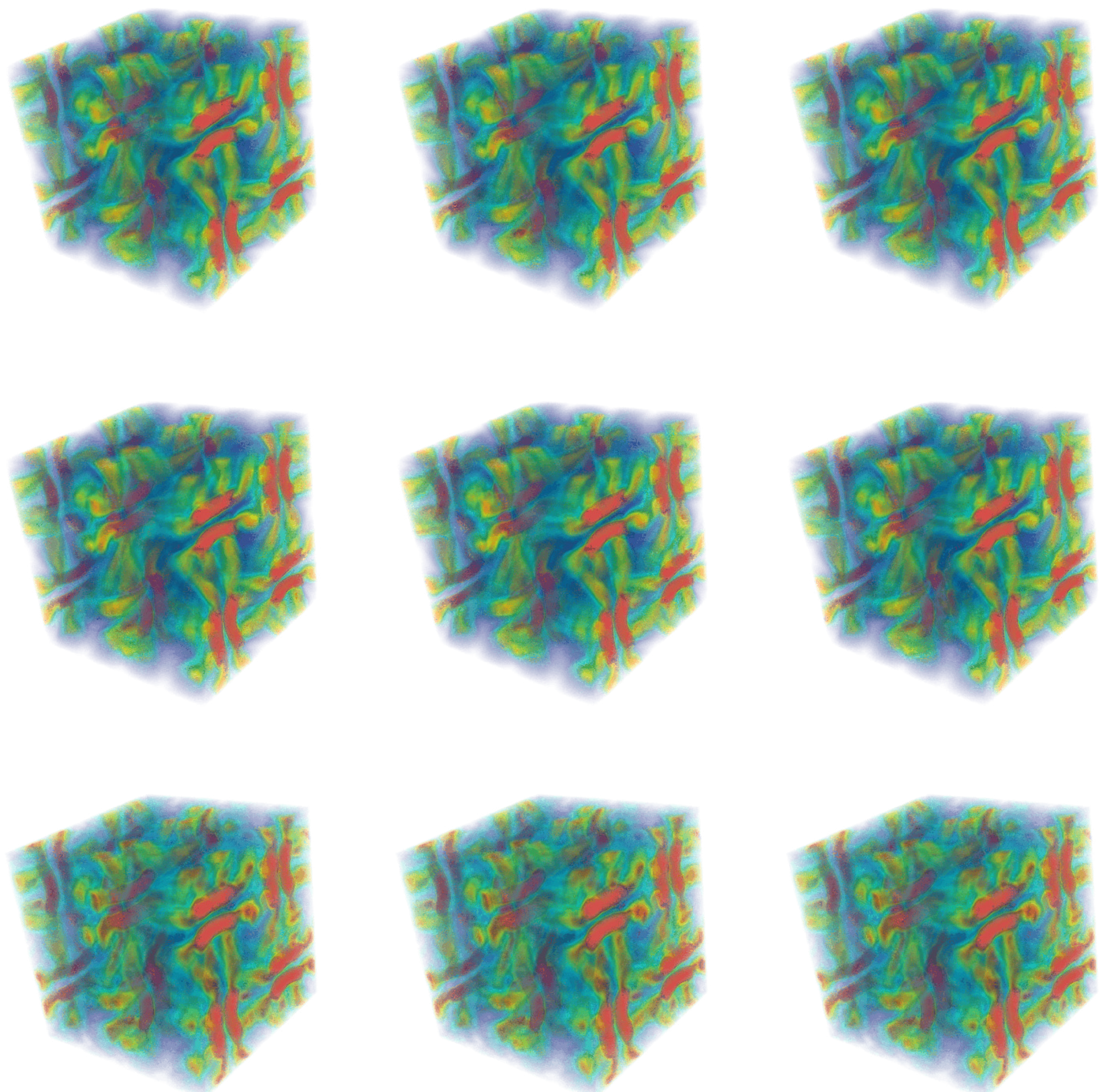

Figure SI 26: Visualization of pointwise vorticity intensity for three randomly generated samples for the Taylor–Green vortex experiment at time $T = 2$. Results are shown for the ground truth (top row), GenCFD (middle row) and C-FNO (bottom row). The colormap for the top and middle rows ranges from 10^{-4} (dark blue) to 40.0 (dark red), whereas for the bottom row, it ranges from 10^{-4} to 35.0.

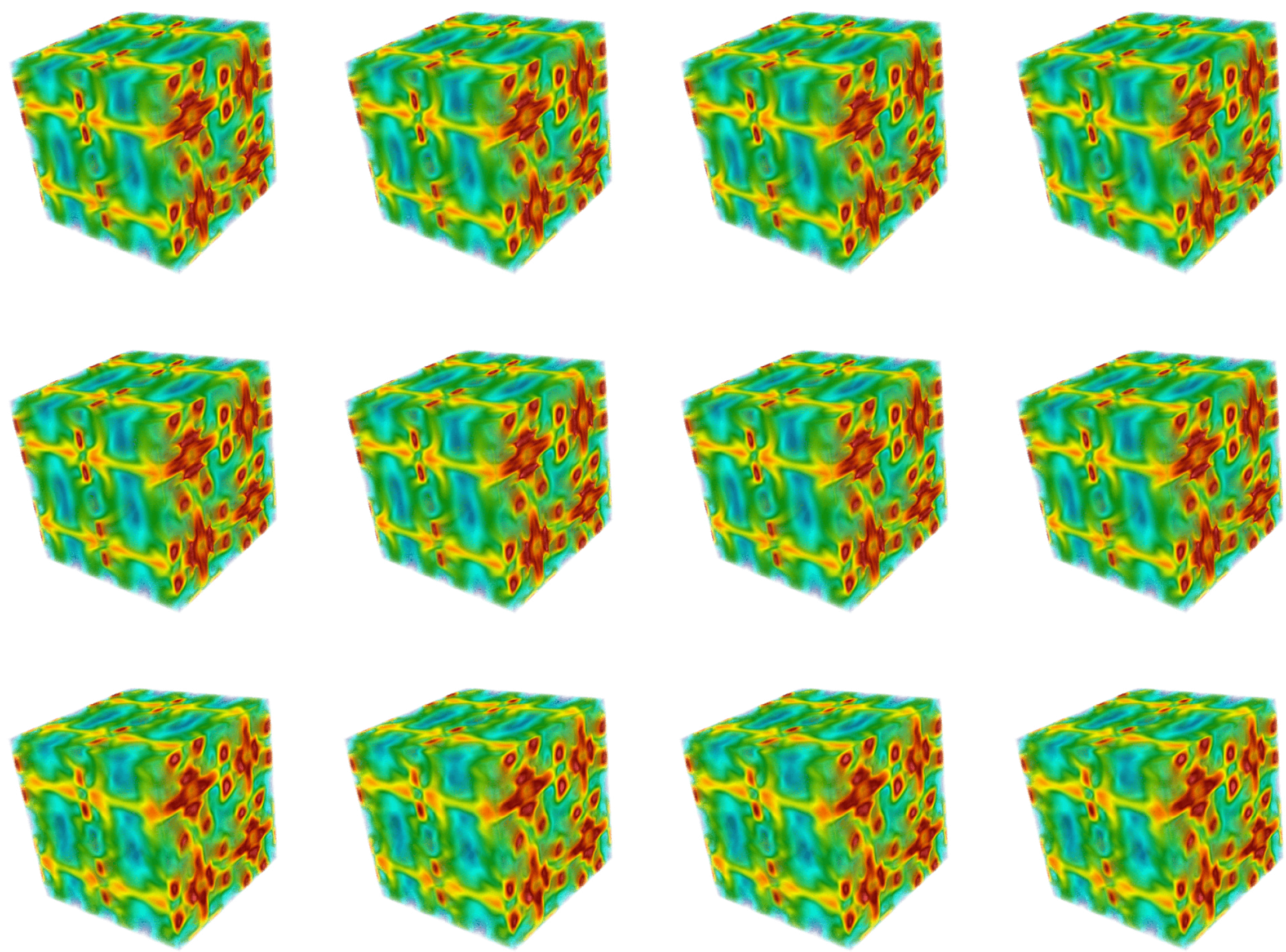

(a) $\bar{u} = \bar{u}^1$ **(b)** $\bar{u} = \bar{u}^2$ **(c)** $\bar{u} = \bar{u}^3$ **(d)** $\bar{u} = \bar{u}^4$

Figure SI 27: Visualization of the mean of the approximate statistical solution for the Taylor–Green vortex experiment at time $T = 2$, for four different initial distributions. Initial distributions (column-wise): **(a):** $\bar{u} = \bar{u}^1$, **(b):** $\bar{u} = \bar{u}^2$, **(c):** $\bar{u} = \bar{u}^3$, **(d):** $\bar{u} = \bar{u}^4$. Results are shown for the ground truth (top row), GenCFD (middle row) and C-FNO (bottom row). The colormap for all the figures ranges from 0.0 (dark blue) to 1.0 (dark red).

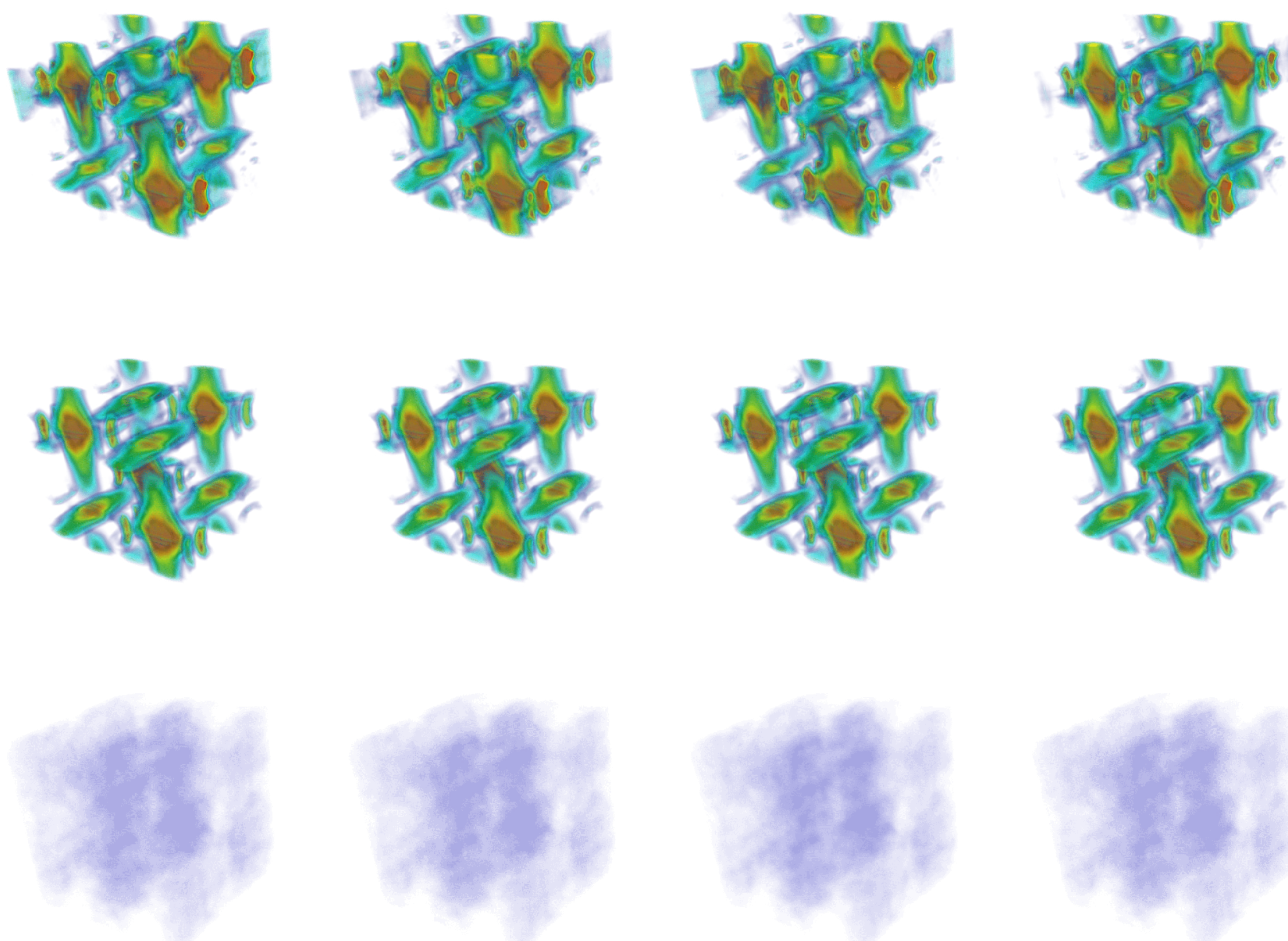

(a) $\bar{u} = \bar{u}^1$ **(b)** $\bar{u} = \bar{u}^2$ **(c)** $\bar{u} = \bar{u}^3$ **(d)** $\bar{u} = \bar{u}^4$

Figure SI 28: Visualization of the standard deviation of the pointwise kinetic energy for the Taylor–Green vortex experiment at time $T = 0.8$, for four different initial distributions. Initial distributions (column-wise): **(a):** $\bar{u} = \bar{u}^1$, **(b):** $\bar{u} = \bar{u}^2$, **(c):** $\bar{u} = \bar{u}^3$, **(d):** $\bar{u} = \bar{u}^4$. Results are shown for the ground truth (top row), GenCFD (middle row) and C-FNO (bottom row). The colormap for the top and middle rows ranges from 0.05 (dark blue) to 0.15 (dark red), whereas for the bottom row, it ranges from 1.3×10^{-4} to 3.5×10^{-3}.

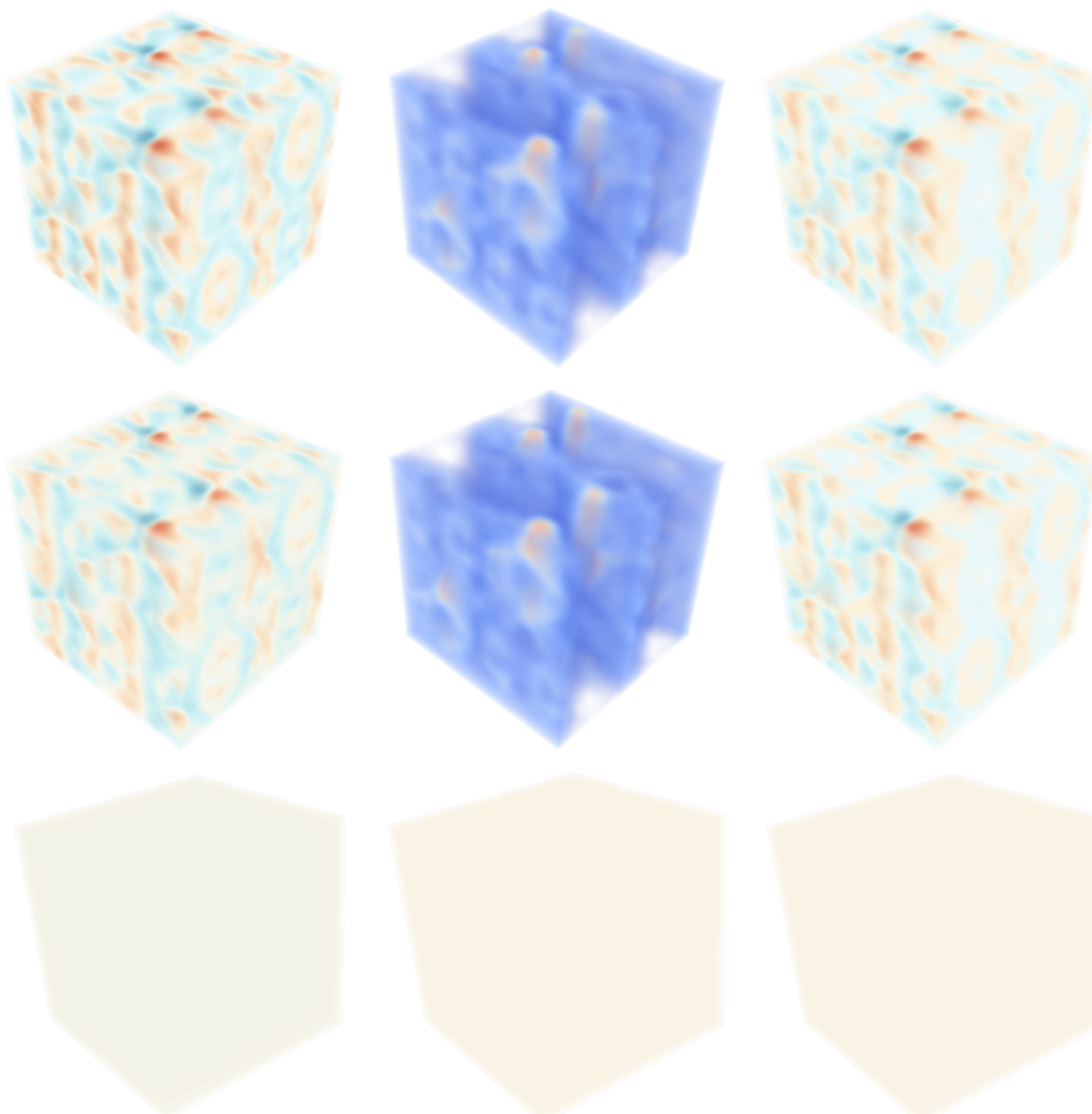

(a) μ_3' (b) μ_4' (c) μ_5'

Figure SI 29: Visualization of the third to fifth central moments of the approximate statistical solution for the Taylor–Green vortex experiment at time $T = 2$. Results are shown for the ground truth (top row), GenCFD (middle row), and FNO (bottom row). The moments **(a):** μ_3', **(b):** μ_4', and **(c):** μ_5' (column-wise) are computed pointwise from the velocity field $u(x, t)$. The colormap range for the third central moment (left column) is from -0.01 (dark blue) to 0.01 (dark red); for the fourth central moment (middle column), it ranges from 4.1×10^{-5} to 1.3×10^{2}; and for the fifth central moment (right column), it ranges from -4.3×10^{-3} to 4.4×10^{-3}.

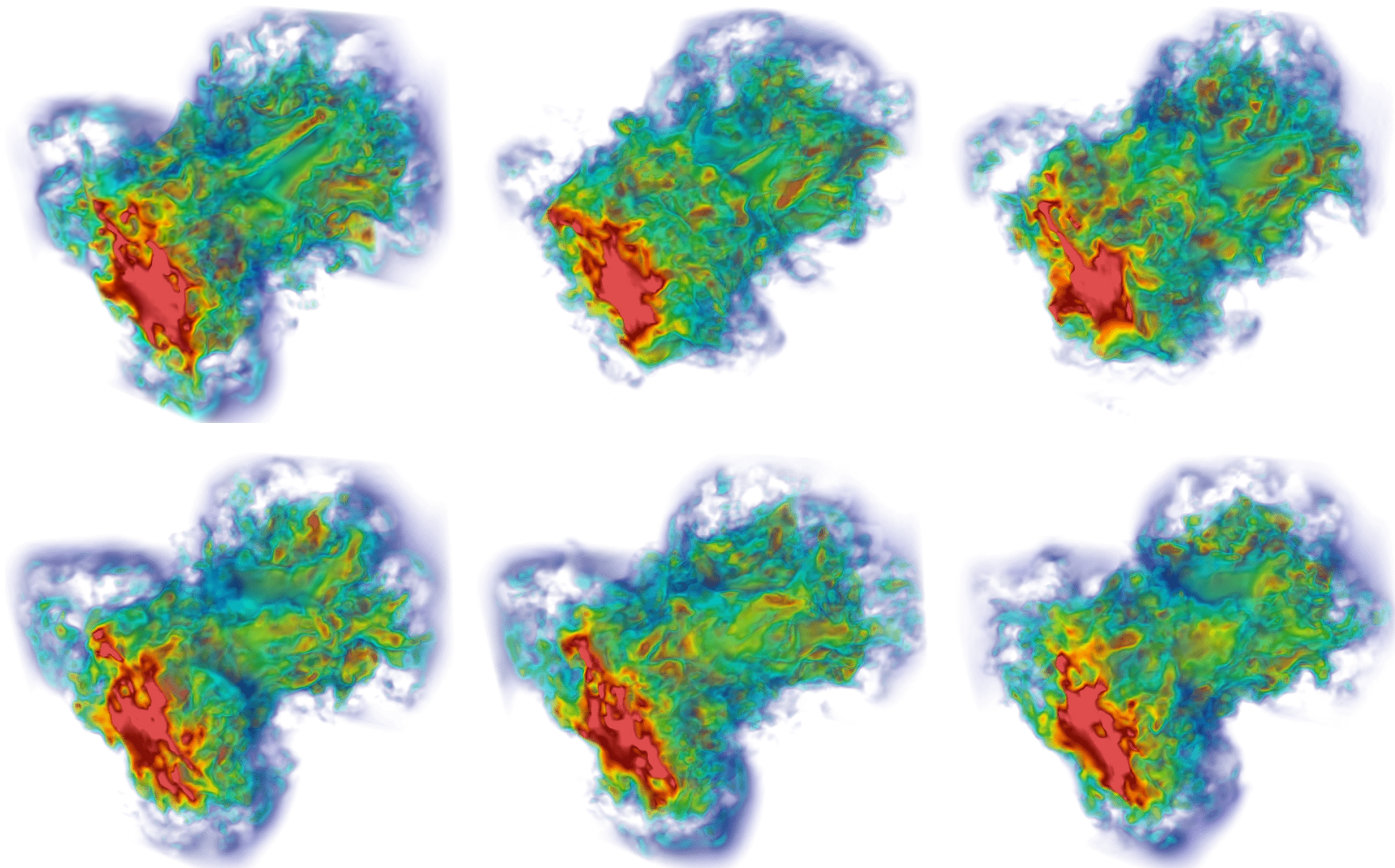

(a) Kinetic energy of ground truth (top) and GenCFD (bottom)

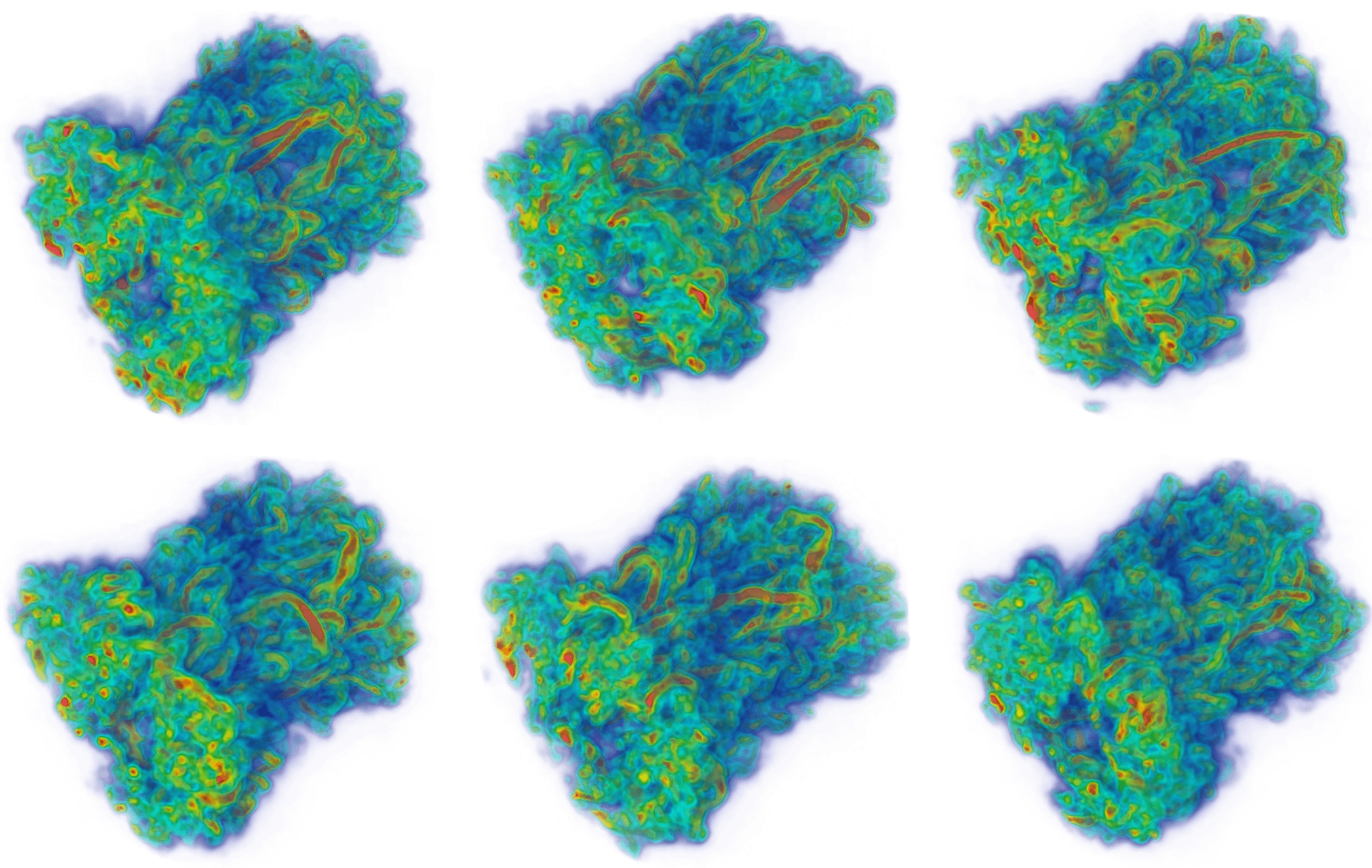

(b) Vorticity of ground truth (top) and GenCFD (bottom)

Figure SI 30: Visualization of pointwise kinetic energy and vorticity for second batch of three different randomly generated samples for the cylindrical shear flow experiment at time $T = 1$. Results of **(a):** pointwise kinetic energy and **(b):** vorticity are shown for three different samples (column-wise) from the ground truth (top row) and GenCFD (bottom row), respectively. The initial condition is different from the one presented in Fig. SI 7. Colormaps are identical to the ones used in Fig. SI 7.

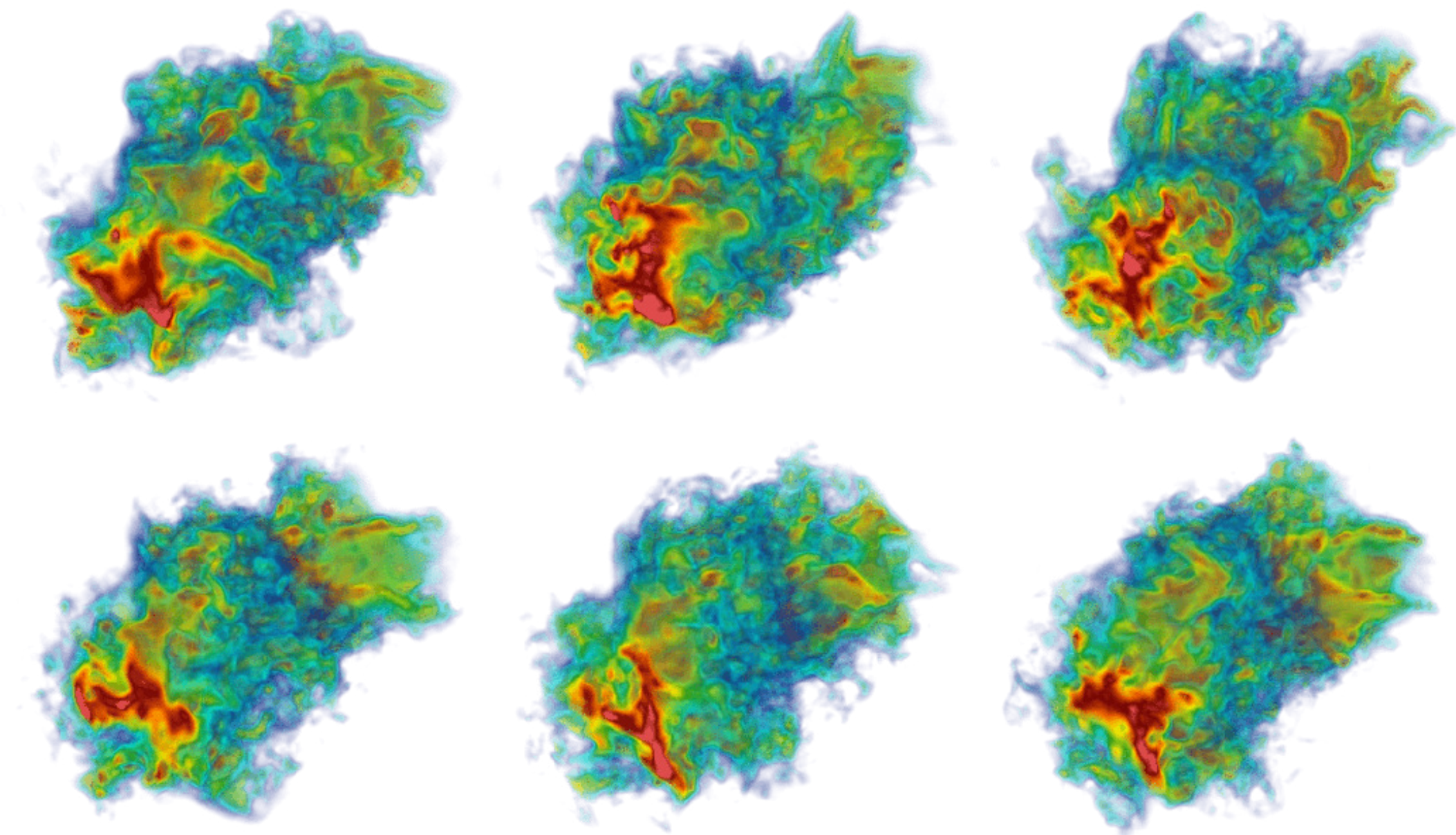

(a) Kinetic energy of ground truth (top) and GenCFD (bottom)

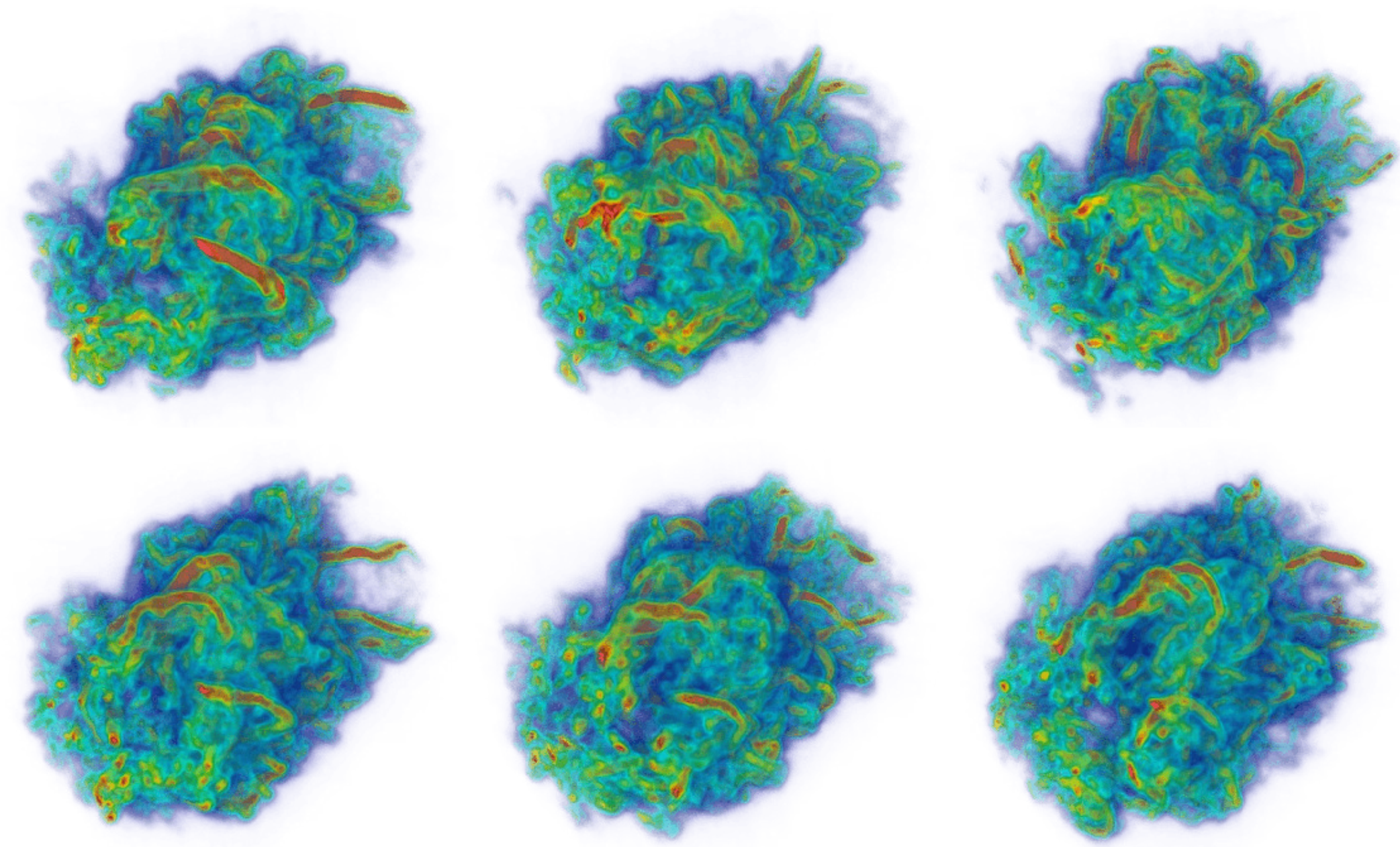

(b) Vorticity of ground truth (top) and GenCFD (bottom)

Figure SI 31: Visualization of pointwise kinetic energy and vorticity for third batch of three different randomly generated samples for the cylindrical shear flow experiment at time $T = 1$. Results of **(a):** pointwise kinetic energy and **(b):** vorticity are shown for three different samples (column-wise) from the ground truth (top row) and GenCFD (bottom row), respectively. The initial condition is different from Figs. SI 7 and SI 30. Colormaps are identical to the ones used in Fig. SI 7.

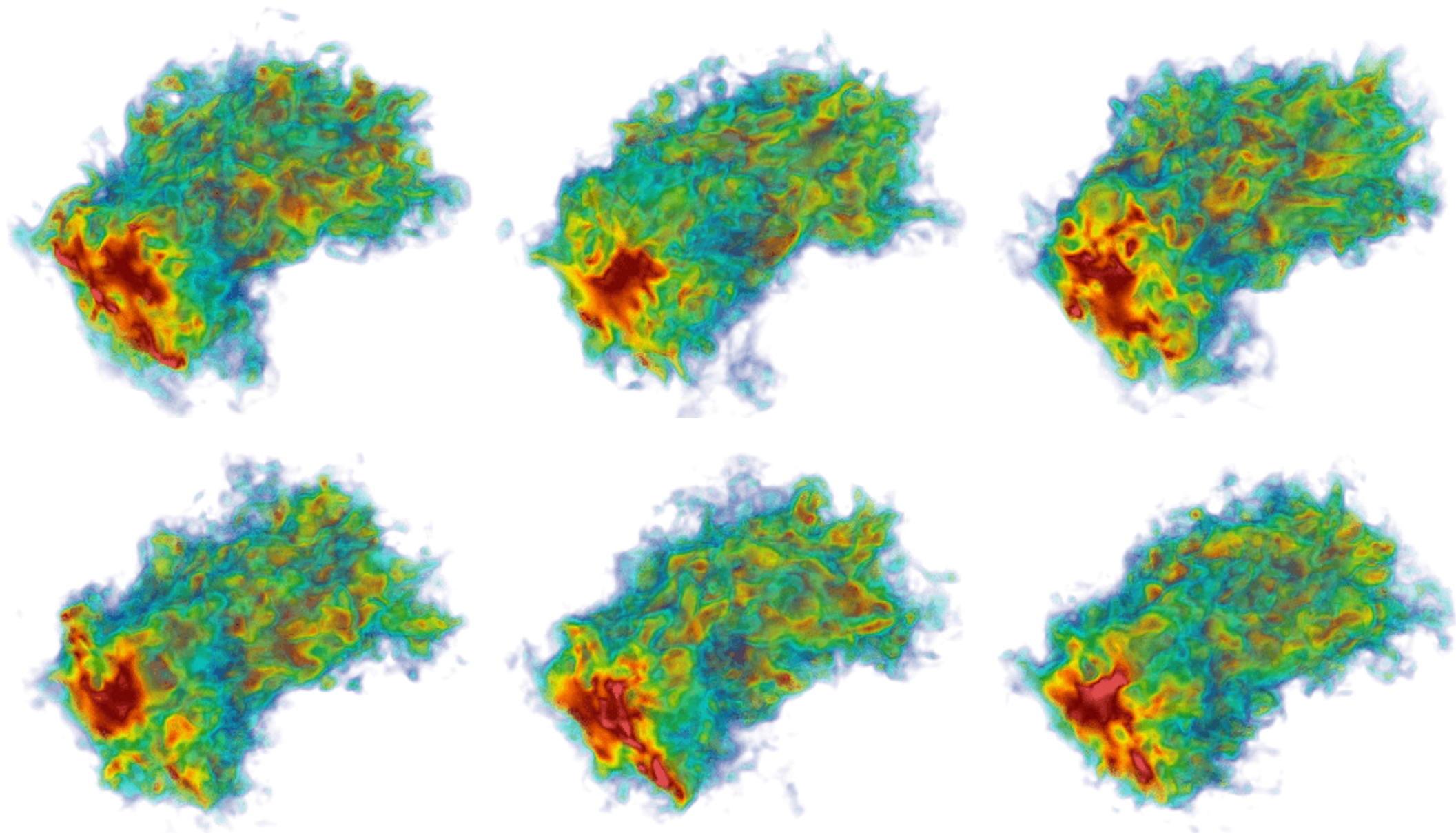

(a) Kinetic energy of ground truth (top) and GenCFD (bottom)

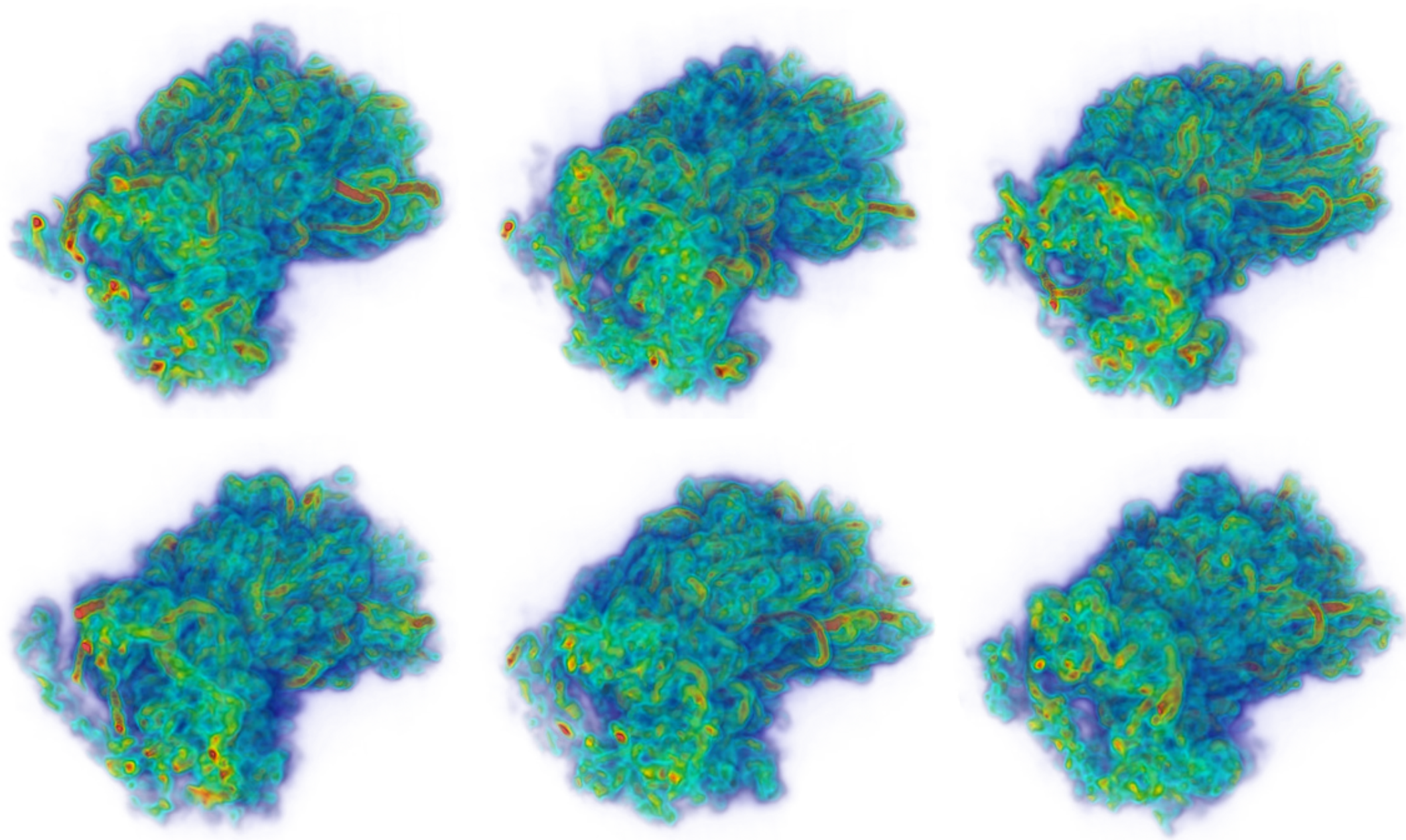

(b) Vorticity of ground truth (top) and GenCFD (bottom)

Figure SI 32: Visualization of pointwise kinetic energy and vorticity for fourth batch of three different randomly generated samples for the cylindrical shear flow experiment at time $T = 1$. Results of **(a):** pointwise kinetic energy and **(b):** vorticity are shown for three different samples (column-wise) from the ground truth (top row) and GenCFD (bottom row), respectively. The initial condition is different from Figs. SI 7, SI 30 and SI 31. Colormaps are identical to the ones used in Fig. SI 7.

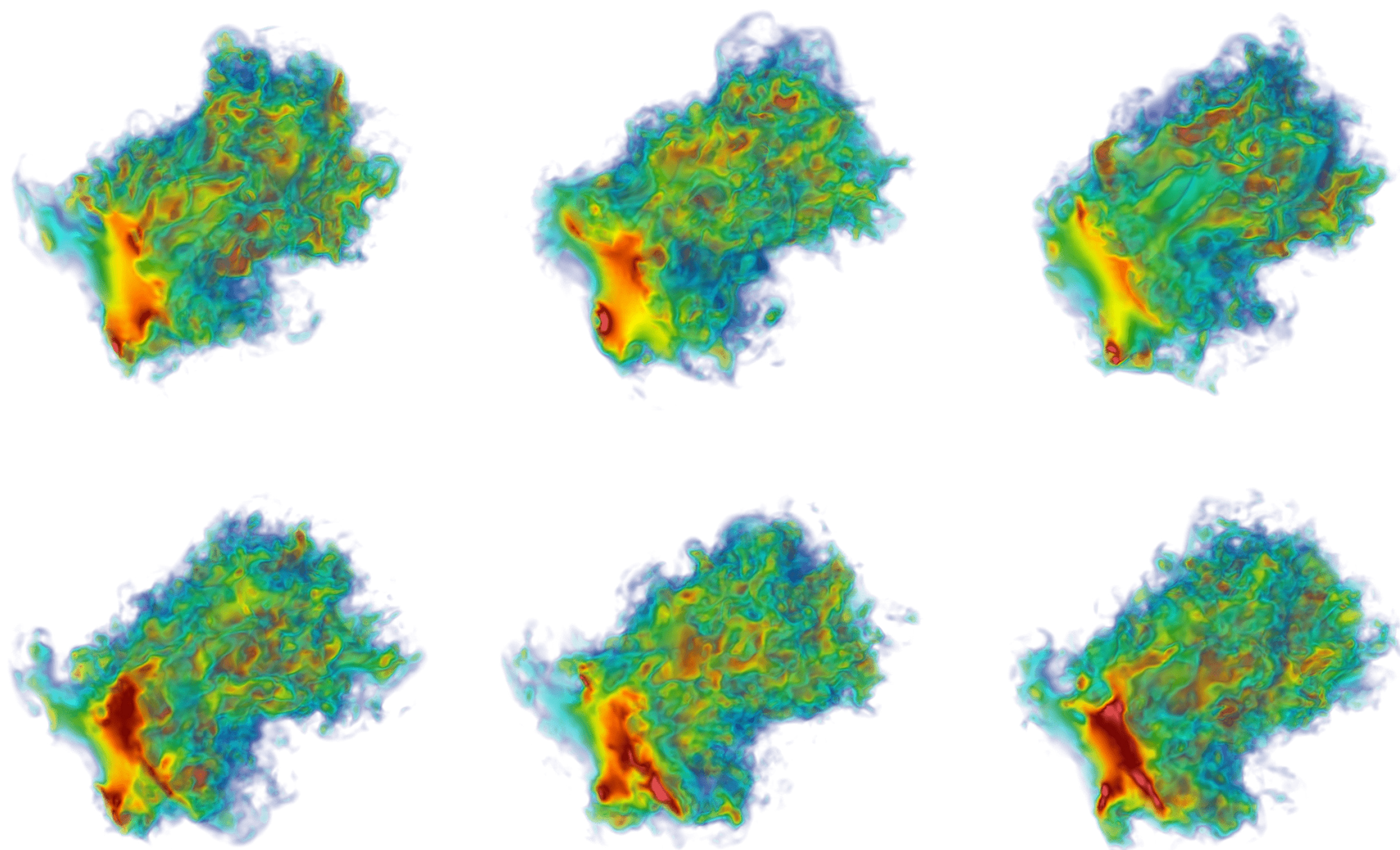

(a) Kinetic energy of ground truth (top) and GenCFD (bottom)

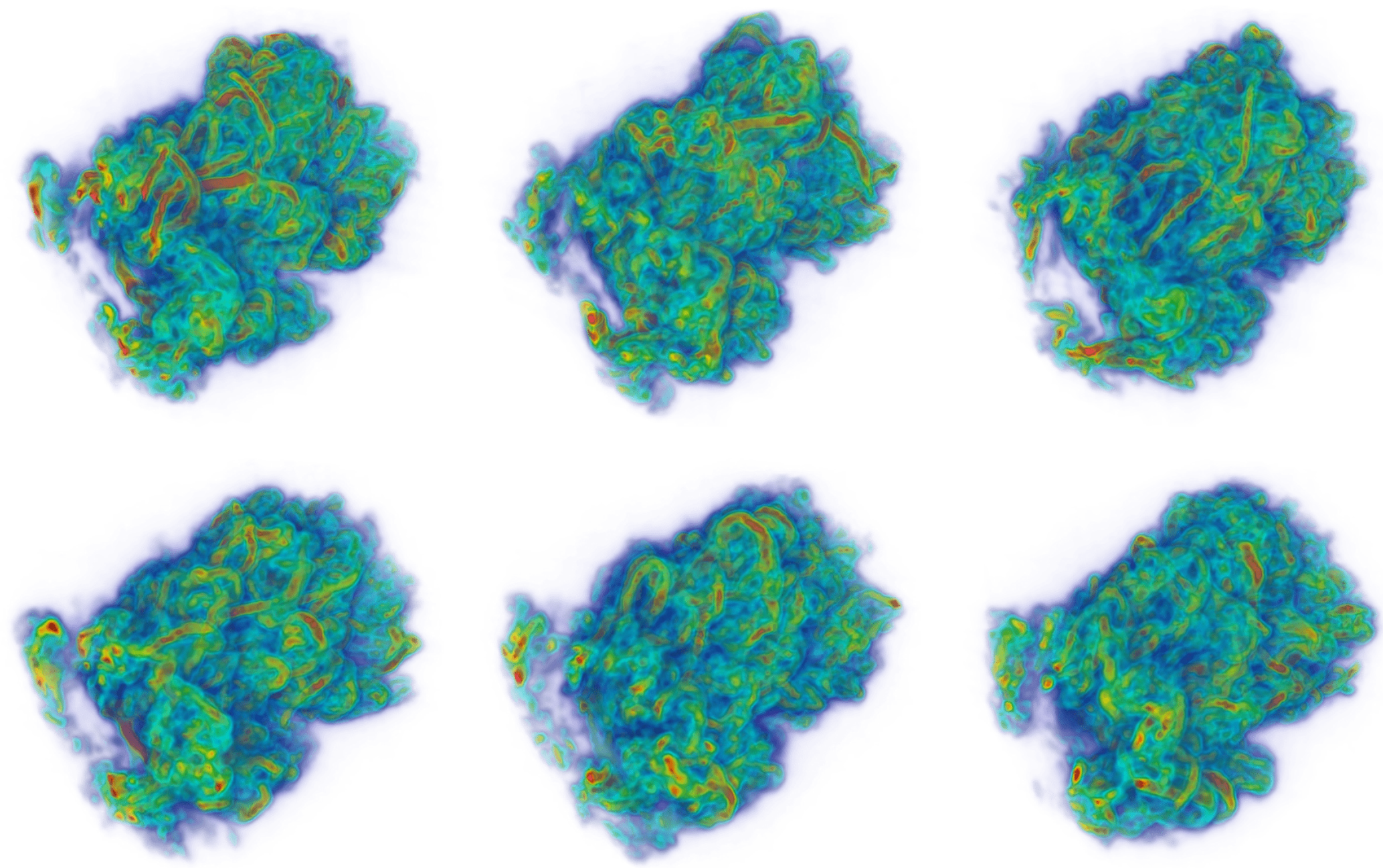

(b) Vorticity of ground truth (top) and GenCFD (bottom)

Figure SI 33: Visualization of the kinetic energy of samples drawn from the ground truth and approximated conditional distribution and corresponding computed vorticity intensity at time $T = 1$. Results of **(a):** pointwise kinetic energy of samples drawn from the ground truth and approximated conditional distribution $p(u|\bar{u} = \bar{u}^2)$ and **(b):** corresponding vorticity intensity are shown for three different samples (column-wise) from the ground truth (top row) and GenCFD (bottom row), respectively. Colormaps are identical to the ones used in Fig. SI 7.

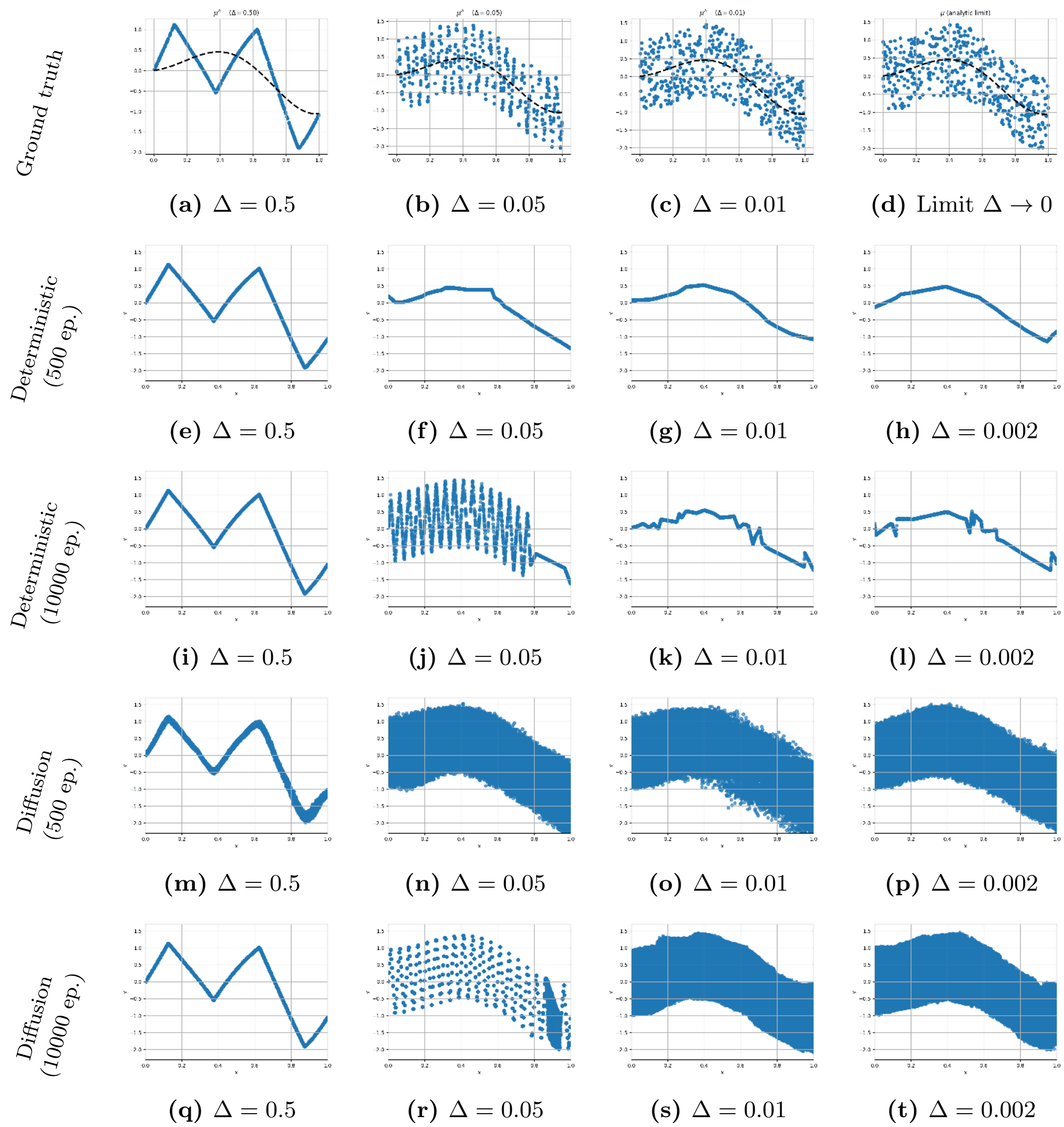

Figure SI 34: Results for Toy Model #1 at different Δ. (**Row 1**): Ground truth, (**Row 2**): Deterministic ML model with 500 epochs of training, (**Row 3**): Deterministic ML model with 10000 epochs, (**Row 4**): Diffusion model with 500 epochs of training, (**Row 5**): Diffusion model with 10000 epochs. Source data are provided as a Source Data file.

$k = 5$:

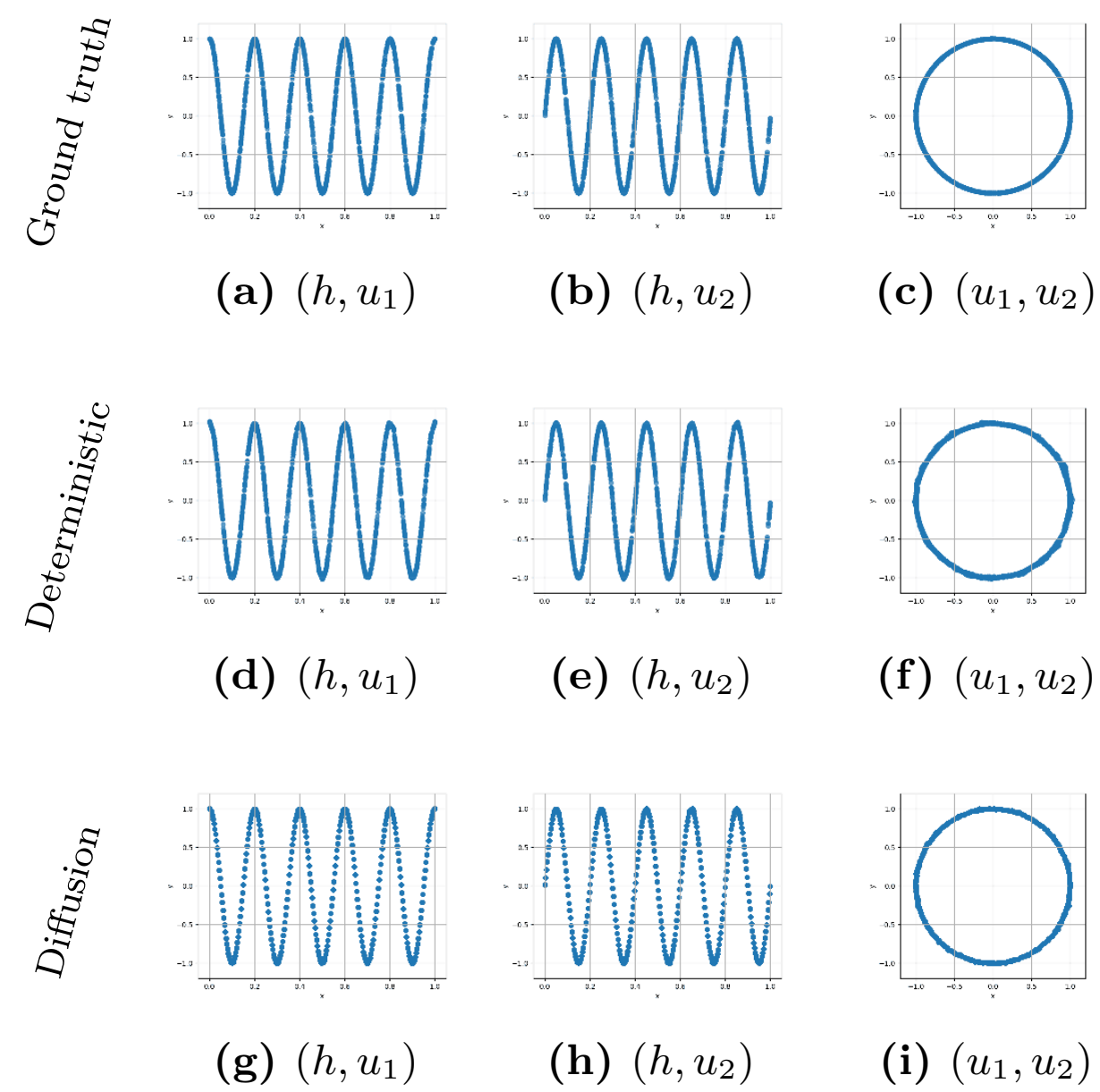

$k = 30$:

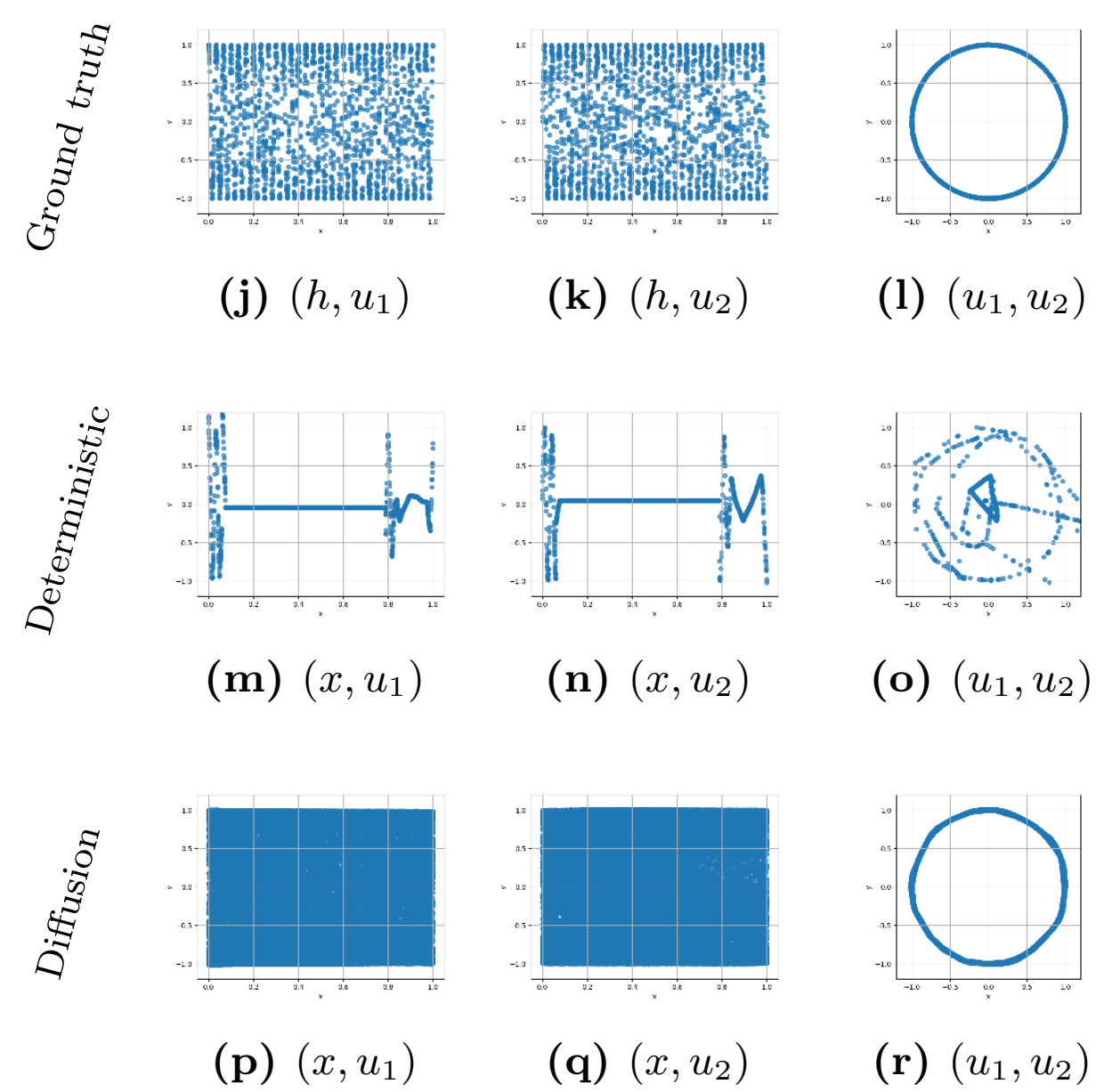

Figure SI 35: Results for Toy Model #2 at two different frequencies. Results for $k = 5$ (top 3 rows) and $k = 30$ (bottom 3 rows) are shown, computed from ground truth (top), deterministic ML model (middle) and diffusion model (bottom) for each frequency, respectively. Source data are provided as a Source Data file.

SI 6 Supplementary Tables

Table SI 1: Comparative summary of generative models for fluid dynamics. The row describing this work is colored in red.

**Work**	**Primary Application**	**Spatial Dimensions**	**Turbulence Fidelity**	**Conditional Dist. Bench.**	**Key Architectural Approach**
Li *et al.* [1]	Weather	2D	N/A (Weather Physics)	Yes (Ensemble Aug.)	Conditional diffusion for ensemble in-filling
Price *et al.* [2]	Weather	2D	N/A (Weather Physics)	No (Prob. Trajectory)	Auto-regressive diffusion on GNNs
Couairon *et al.* [3]	Weather	2D	N/A (Weather Physics)	No (Prob. Trajectory)	Auto-regressive flow matching
Lienen *et al.* [4]	CFD	3D (1 type)	LES (Smooth)	No	Unconditional diffusion
Saydemir *et al.* [5]	CFD	3D (1 type)	LES (Smooth)	No	4D diffusion for short sequences
Gao *et al.* [6]	CFD	3D (1 example)	LES (Smooth)	No	Coarse-space dynamics + Diffusion decoder
Oomen *et al.* [8]	CFD	Mostly 2D	LES	No	Deterministic operator + Diffusion corrector
Valencia *et al.* [9]	CFD (Aero)	3D (Surface)	RANS/LES (Equilibrium)	No	Latent diffusion/ flow matching on graphs
Du *et al.* [10]	CFD	Mostly 2D	DNS (Coarse)	No	Unconditional latent diffusion on neural field
**This Work**	**CFD / UQ**	**3D (5 examples)**	**DNS, LES**	**Yes**	**End-to-end conditional score-based diffusion**

Table SI 2: Parameters in the convective boundary layer experiment. Default values are as given in [43], the lower and upper bounds are used for uniform sampling of the training data. For testing with a Dirac distribution, the default values are used.

Parameter	Unit	Type	Lower bound	Default value	Upper bound
Q_*	Kms^{-1}	Forcing	0.1	0.24	0.3
U_g	ms^{-1}	Forcing	0.0	1.0	5.0
T_g	K	Initial condition	297.5	300.0	302.5
z_i	m	Initial condition	974.0	1024.0	1074.0
$(\partial_3\theta)_i$	Km^{-1}	Initial condition	0.03	0.08	0.15

Table SI 3: Details of the GenCFD architectures used in the benchmarks. The column *In/Out Ch.* corresponds to the number of input/output channels of the models.

Benchmark	Model	Size	Resolution	In/Out Ch.
CSF, TGV	*base*	70.2M	64^3	3/3
CSI	*base*	70.2M	64^3	4/4
NF	*base*	70.2M	$64 \times 64 \times 192$	4/3
CBL	*small*	40.1M	128^3	5/4

Table SI 4: Experimental details for the GenCFD models. The first column indicates the benchmark, the second shows the number of trajectories (*Num. Traj.*) used for training, and the third lists the total number of training samples for all-to-all training (*Num. Sam.*). The fourth column specifies the batch size (*B. S.*), the fifth details the number of gradient steps (*Num. Grad. S.*) during training, and the sixth provides the approximate number of training epochs (*Epoch*). Finally, the seventh column notes the number of GPUs used for training along with their memory (*GPU (num:mem)*).

Benchmark	Num. Traj.	Num. Sam.	B. S.	Num. Grad. S.	Epoch	GPU (num : mem)
TGV	6.6K	99K	5	1.0M	50.5	1 : 24GB
CSF	9.9K	99K	5	1.0M	50.5	1 : 24GB
CSI	9.9K	99K	2	0.8M	16.2	1 : 24GB
NF	9K	189K	4	0.1M	2.2	1 : 80GB
CBL	7.5K	157.5K	4	0.25M	6.3	4 : 80GB

Table SI 5: Details of the FNO architectures used in the benchmarks. The d_v corresponds to the lifting dimension, L to the number of Fourier layers, M number of modes used in the Fourier layer, lr to the peak learning rate, *B. S.* to the batch size. The architectures are obtained using a random grid search over a range of hyperparameters (typically 10 to 20 configurations).

Benchmark	d_v	L	M	lr	B. S.	Size
TGV	64	5	12	0.0001	2	42.1M
CSL	64	5	16	0.0001	5	95.2M
CSI	64	5	16	0.0001	2	95.2M
NF	64	5	12	0.0001	1	42.1M
CBL	48	4	16	0.0001	1	43.1M

Table SI 6: Details of the C-FNO architectures used in the benchmarks. The d_v corresponds to the lifting dimension, L to the number of Fourier layers, M number of modes used in the Fourier layer, lr to the peak learning rate, $B.\ S.$ to the batch size. The architectures are obtained using a random grid search over a range of hyperparameters (typically 10 to 20 configurations).

Benchmark	d_v	L	M	lr	B. S.	Size
TGV	64	4	12	0.0001	2	40.0M
CSL	64	4	16	0.0001	3	82.4M
CSI	64	3	16	0.0001	2	62.1M
NF	64	5	16	0.0001	1	102.7M
CBL	48	4	16	0.0001	1	46.9M

Table SI 7: Overview of error metrics and other case-specific quantities evaluated in the present benchmark computations.

Benchmark	e_μ	e_σ	W_1	CRPS_G	E_k	PDF	Others
TGV	√	√	√	√	√	√	At several timesteps, higher order moments $e_{\mu_i'}$
CSL	√	√	√	√	√	√	At several timesteps, for several distributions, for several reverse SDE steps
CSI	√	√	√	√	√	√	
NF	√	√	√	√	√	√	
CBL	√	√	√	√	√	√	Other statistics (cf. Fig. SI 22)

Table SI 8: Error metrics for different models for the Taylor–Green vortex experiment. The aim is to compare CRPS training with GenCFD. The metrics, defined in Sec. SI 2.8, are all computed at time $T = 2.0$. Results for the best performing model are in bold.

		e_μ	e_σ	W_1
GenCFD	u_x	0.154	**0.056**	**0.0165**
	u_y	**0.155**	**0.055**	**0.0172**
	u_z	0.282	**0.053**	**0.0145**
C-FNO	u_x	0.210	1.000	0.117
	u_y	0.210	1.000	0.118
	u_z	0.402	1.000	0.115
CRPS	u_x	0.162	0.499	0.0616
	u_y	0.160	0.496	0.0610
	u_z	**0.242**	0.494	0.0580

Table SI 9: Error metrics for CRPS in the Taylor–Green vortex experiment. The aim is to compare CRPS training for different number of ensemble members n_E in (324). The metrics, defined in Sec. SI 2.8, are all computed at time $T = 2.0$. Results for the best performing model are in bold.

model (n_E)		e_μ	e_σ	W_1
CRPS (1)	u_x	0.174	0.988	0.1142
	u_y	0.173	0.988	0.1137
	u_z	0.255	0.989	0.1089
CRPS (4)	u_x	0.162	**0.499**	**0.0616**
	u_y	0.160	**0.496**	**0.0610**
	u_z	0.242	**0.494**	**0.0580**
CRPS (16)	u_x	**0.158**	0.596	0.0687
	u_y	**0.155**	0.593	0.0678
	u_z	**0.232**	0.597	0.0645

Table SI 10: Error metrics for the GenCFD generated velocity fields and their Leray projected versions, for the Taylor–Green vortex at $T = 2.0$.

		e_μ	e_σ	W_1
Original	u_x	0.149	0.090	0.018
	u_y	0.157	0.086	0.019
	u_z	0.287	0.089	0.017
Projection	u_x	0.149	0.091	0.018
	u_y	0.157	0.087	0.019
	u_z	0.287	0.089	0.017

Table SI 11: Error metrics for different models for the Taylor–Green vortex experiment. The metrics, as defined in Sec. SI 2.8, are computed at time $T = 2.0$. Results for the best performing model are in bold.

		e_μ	e_σ	W_1	CRPS_G
GenCFD	u_x	**0.154**	**0.056**	**0.0165**	**0.481**
	u_y	**0.155**	**0.055**	**0.0172**	**0.479**
	u_z	**0.282**	**0.053**	**0.0145**	**0.469**
UViT	u_x	0.883	0.813	0.130	0.768
	u_y	0.944	0.829	0.138	0.802
	u_z	1.016	0.881	0.110	0.648
FNO	u_x	0.359	0.999	0.121	0.690
	u_y	0.362	0.999	0.123	0.690
	u_z	0.756	0.998	0.119	0.671
C-FNO	u_x	0.210	1.000	0.117	0.670
	u_y	0.210	1.000	0.118	0.668
	u_z	0.402	1.000	0.115	0.653
GenCFD* $\circ$ FNO	u_x	0.168	0.073	0.0175	0.853
	u_y	0.158	0.068	0.0177	0.591
	u_z	0.328	0.068	0.0165	0.558

Table SI 12: Error metrics for different models at $T = 1.0$ for the cylindrical shear flow experiment. Results for the best performing model are in bold.

		e_μ	e_σ	W_1	CRPS_G
GenCFD	u_x	**0.088**	**0.114**	**0.034**	**0.347**
	u_y	**0.271**	**0.110**	**0.030**	**0.316**
	u_z	**0.268**	**0.113**	**0.032**	**0.317**
UViT	u_x	0.604	0.562	0.253	0.708
	u_y	1.096	0.558	0.147	0.443
	u_z	1.038	0.663	0.150	0.475
FNO	u_x	0.301	0.957	0.169	0.547
	u_y	0.889	0.959	0.148	0.486
	u_z	0.815	0.962	0.150	0.485
C-FNO	u_x	0.217	0.864	0.133	0.452
	u_y	0.622	0.880	0.120	0.405
	u_z	0.641	0.880	0.124	0.417

Table SI 13: Results for error metrics for different models at time $T = 1.0$ for the cloud-shock interaction experiment. Results for the best performing model are in bold.

		e_μ	e_σ	W_1	CRPS_G
GenCFD	ρ	**0.049**	**0.381**	**0.054**	**0.0035**
	u_x	**0.015**	**0.332**	**0.093**	**0.0040**
	u_y	**0.195**	**0.203**	**0.054**	0.0033
	u_z	**0.171**	**0.301**	**0.021**	**0.0012**
	p	**0.015**	**0.316**	**1.525**	**0.0436**
UViT	ρ	0.252	0.993	0.400	0.0151
	u_x	0.095	0.985	0.631	0.0151
	u_y	0.849	0.992	0.283	0.0065
	u_z	0.722	0.992	0.158	0.0046
	p	0.127	0.980	13.68	0.2834
FNO	ρ	0.138	0.921	0.242	0.0085
	u_x	0.071	0.940	0.393	0.0111
	u_y	0.680	0.973	0.223	0.0054
	u_z	0.424	0.929	0.100	0.0027
	p	0.081	0.892	8.328	0.1782
C-FNO	ρ	0.081	0.798	0.133	0.0052
	u_x	0.037	0.688	0.264	0.0058
	u_y	0.399	0.613	0.127	**0.0032**
	u_z	0.236	0.878	0.042	0.0017
	p	0.038	0.630	4.128	0.0811

Table SI 14: Results for error metrics for different models at time $T = 1.0$ for the nozzle flow experiment. Results for the best performing model are in bold. Note that the term N/A for the mean error e_μ for the u_y, u_z components signifies the fact that the ground-truth means of these velocity components are 0 and the relative errors are not well-defined.

		e_μ	e_σ	W_1	CRPS_G
GenCFD	u_x	0.148	**0.258**	**0.0073**	**0.268**
	u_y	N/A	**0.207**	**0.0049**	**0.508**
	u_z	N/A	**0.218**	**0.0050**	**0.515**
	E_k	**0.151**	**0.240**	**0.00065**	**0.230**
UViT	u_x	**0.074**	0.850	0.0109	0.324
	u_y	N/A	0.926	0.0098	0.657
	u_z	N/A	0.943	0.0100	0.669
	E_k	0.167	0.827	0.00128	0.276
FNO	u_x	0.176	0.864	0.0139	0.358
	u_y	N/A	0.934	0.0111	0.678
	u_z	N/A	0.956	0.0112	0.693
	E_k	0.237	0.848	0.00136	0.316
C-FNO	u_x	0.124	0.858	0.0116	0.333
	u_y	N/A	0.923	0.0100	0.661
	u_z	N/A	0.938	0.0102	0.673
	E_k	0.207	0.835	0.00134	0.287

Table SI 15: Results for error metrics for different models at time $T = 1.0$ for the dry convective boundary layer experiment. Results for the best performing model are in bold. Note that the term N/A for the mean error e_μ for the u_y, u_z components signifies the fact that the ground-truth means of these velocity components are 0 and the relative errors are not well-defined.

		e_μ	e_σ	W_1	CRPS_G
GenCFD	u_x	**0.223**	**0.072**	**0.036**	**0.557**
	u_y	N/A	**0.094**	**0.038**	**0.567**
	u_z	N/A	**0.059**	**0.037**	**0.553**
	T	$10 \cdot 10^{-5}$	**0.091**	**0.025**	**0.00060**
	E_k	**0.109**	**0.137**	**0.072**	
UViT	u_x	0.235	0.807	0.305	0.692
	u_y	N/A	0.827	0.308	0.714
	u_z	N/A	0.825	0.403	0.704
	T	$\mathbf{6 \cdot 10^{-5}}$	0.890	0.108	0.00086
	E_k	0.956	0.965	0.644	
FNO	u_x	0.345	0.936	0.343	0.753
	u_y	N/A	0.849	0.315	0.730
	u_z	N/A	0.973	0.470	0.779
	T	$18 \cdot 10^{-5}$	0.612	0.080	0.00074
	E_k	0.978	0.986	0.657	
C-FNO	u_x	0.293	0.875	0.332	0.722
	u_y	N/A	0.922	0.360	0.762
	u_z	N/A	0.909	0.453	0.754
	T	$44 \cdot 10^{-5}$	0.807	0.143	0.00100
	E_k	0.977	0.981	0.659	

Table SI 16: Error metrics for different models at $T = 0.8$ for the Taylor–Green vortex experiment. Results for the best performing model are in bold.

		e_μ	e_σ	W_1	CRPS_G
GenCFD	u_x	**0.030**	**0.228**	**0.0077**	**0.053**
	u_y	**0.030**	**0.227**	**0.0075**	**0.050**
	u_z	**0.030**	**0.251**	**0.0061**	**0.039**
UViT	u_x	0.843	1.219	0.203	0.832
	u_y	0.880	1.328	0.207	0.869
	u_z	0.957	1.207	0.175	0.762
FNO	u_x	0.458	0.989	0.100	0.489
	u_y	0.459	0.987	0.102	0.489
	u_z	0.556	0.978	0.096	0.473
C-FNO	u_x	0.151	0.997	0.0389	0.171
	u_y	0.146	0.997	0.0367	0.166
	u_z	0.166	0.997	0.0317	0.147

Table SI 17: Error metrics of higher central moments for different models at $T = 2.0$ for the Taylor–Green vortex experiment. Results for the best performing model are in bold.

		$e_{\mu'_3}$	$e_{\mu'_4}$	$e_{\mu'_5}$
GenCFD	u_x	**0.349**	**0.129**	**0.360**
	u_y	**0.317**	**0.107**	**0.322**
	u_z	**0.326**	**0.129**	**0.364**
FNO	u_x	1.000	1.000	1.000
	u_y	1.000	1.000	1.000
	u_z	1.000	1.000	1.000

Table SI 18: Error metrics for GenCFD for the cylindrical shear flow experiment at $T = 1$. The tests were performed with data from a different distribution than the ones from Table SI 12.

		e_μ	e_σ	W_1	CRPS_G
0-Shot	u_x	0.230	0.240	0.100	0.422
	u_y	0.507	0.228	0.051	0.344
	u_z	0.468	0.227	0.051	0.343
Fine-Tuned	u_x	**0.097**	**0.136**	**0.037**	**0.339**
	u_y	**0.309**	**0.133**	**0.034**	**0.309**
	u_z	**0.309**	**0.134**	**0.032**	**0.310**

Table SI 19: Errors in Wasserstein metric for the distribution generated by GenCFD for the cylindrical shear flow experiment. The errors are with respect to the ground-truth distribution for the cylindrical shear flow experiment at different times in the evolution (column-wise) and for each velocity component (row-wise).

		$T = 0.25$	$T = 0.5$	$T = 0.75$	$T = 1$
GenCFD	u_x	0.016	0.022	0.027	0.034
	u_y	0.013	0.021	0.025	0.030
	u_z	0.014	0.022	0.024	0.032

Table SI 20: Errors in Wasserstein metric for the distribution generated by GenCFD for the cylindrical shear flow experiment at $T = 1$. The errors are with respect to the ground-truth distribution and computed for different numbers of steps K of the reverse SDE (12) (column-wise) and for each velocity component (row-wise).

		$K = 8$	$K = 16$	$K = 32$	$K = 64$	$K = 128$
GenCFD	u_x	1.736	0.051	0.035	0.034	0.034
	u_y	2.311	0.049	0.031	0.030	0.030
	u_z	2.087	0.045	0.033	0.032	0.032

Table SI 21: Runtimes and speedup for generating a single sample with the CFD solvers and with GenCFD. The CFD solvers were used to simulate the ground truth (see Sec. SI 2.6 on which CFD solver is used for which experiment). The inference time to generate a single sample with GenCFD was measured on a NVIDIA RTX 4090 GPU with 24GB of memory. The term N/A implies that the corresponding GPU or CPU code is not available or has not been tested for the corresponding CFD solver. Note that in this comparison different machines have been used than for the sample generation. The computation time in seconds is rounded and includes I/O operations. For the TGV, CSF, and CSI the ground truth (CPU) has been computed on a single Intel Core i7-9750H with 6 cores. The respective ground truth (GPU) was computed on an NVIDIA H100 GPU with 96GB of memory. For the NF the ground truth (CPU) has been computed on two Intel Xeon Gold 6326 with 16 cores each and the ground truth (GPU) has been computed on an NVIDIA H100 GPU, respectively. For CBL the ground-truth data was generated on a cluster with diverse CPU hardware (mostly AMD EPYC 7H12 and AMD EPYC 7763 processors), and the mean runtime on a single core is reported. Speedups of using GenCFD for sample generation are in bold. It should be noted that this comparison reflects inference in terms of sample generation only and excludes the initial training time for GenCFD.

Benchmark	Ground truth (GPU)	Ground truth (CPU)	GenCFD (GPU)	**Speedup (wrt GPU)**	**Speedup (wrt CPU)**
TGV, CSF	1.07×10^1 secs	0.72×10^3 secs	0.450×10^0 secs	$\mathbf{2.37 \times 10^1}$	$\mathbf{1.60 \times 10^3}$
CSI	0.390×10^3 secs	1.80×10^4 secs	0.450×10^0 secs	$\mathbf{0.87 \times 10^3}$	$\mathbf{0.40 \times 10^5}$
NF	1.20×10^3 secs	1.17×10^4 secs	1.45×10^0 secs	$\mathbf{0.83 \times 10^3}$	$\mathbf{0.81 \times 10^4}$
CBL	N/A	0.48×10^5 secs	0.38×10^1 secs	N/A	$\mathbf{1.25 \times 10^4}$

Table SI 22: Error metrics for GenCFD and the training statistics surrogate model for the Taylor–Green vortex experiment at $T = 2$. The aim of this table was to show how different training and test distributions are in this setting. Best results are in bold. For instance, using the mean of the training data as the predictor resulted in errors that are five times larger than GenCFD for the mean, one order of magnitude larger in the variance and two orders of magnitude larger in the Wasserstein distance. These mean predictors are also significantly worse than the deterministic baselines.

		e_μ	e_σ	W_1
GenCFD	u_x	**0.154**	**0.056**	**0.0165**
	u_y	**0.155**	**0.055**	**0.0172**
	u_z	**0.282**	**0.053**	**0.0145**
Train Dist.	u_x	0.844	1.000	0.5922
	u_y	0.843	1.000	0.5865
	u_z	0.937	1.000	0.6932

References

[1] L. Li et al. "Generative emulation of weather forecast ensembles with diffusion models". In: *Science Advances* 10.13 (2024), eadk4489. DOI: 10.1126/sciadv.adk4489.

[2] I. Price et al. *GenCast: Diffusion-based ensemble forecasting for medium-range weather*. 2024. DOI: 10.48550/arXiv.2312.15796. arXiv: 2312.15796 [cs.LG]. URL: https://arxiv.org/abs/2312.15796.

[3] G. Couairon et al. *ArchesWeather & ArchesWeatherGen: a deterministic and generative model for efficient ML weather forecasting*. 2024. DOI: 10.48550/arXiv.2412.12971. arXiv: 2412.12971 [cs.LG]. URL: https://arxiv.org/abs/2412.12971.

[4] M. Lienen et al. "From Zero to Turbulence: Generative Modeling for 3D Flow Simulation". In: *The Twelfth International Conference on Learning Representations*. 2024. URL: https://openreview.net/forum?id=ZhlwoC1XaN.

[5] A. Saydemir, M. Lienen, and S. Günnemann. *Unfolding Time: Generative Modeling for Turbulent Flows in 4D*. 2024. DOI: 10.48550/arXiv.2406.11390. arXiv: 2406.11390 [physics.flu-dyn]. URL: https://arxiv.org/abs/2406.11390.

[6] H. Gao, S. Kaltenbach, and P. Koumoutsakos. "Generative learning for forecasting the dynamics of high-dimensional complex systems". In: *Nature Communications* 15 (2024), p. 8904. DOI: 10.1038/s41467-024-53165-w.

[7] Z. Y. Wan et al. "Debias Coarsely, Sample Conditionally: Statistical Downscaling through Optimal Transport and Probabilistic Diffusion Models". In: *Advances in Neural Information Processing Systems*. Ed. by A. Oh et al. Vol. 36. Curran Associates, Inc., 2023, pp. 47749–47763.

[8] V. Oommen et al. "Integrating neural operators with diffusion models improves spectral representation in turbulence modelling". In: *Proceedings of the Royal Society A: Mathematical, Physical and Engineering Sciences* 481.2309 (Mar. 2025), p. 20240819. DOI: 10.1098/rspa.2024.0819.

[9] M. L. Valencia, T. Pfaff, and N. Thuerey. "Learning Distributions of Complex Fluid Simulations with Diffusion Graph Networks". In: *The Thirteenth International Conference on Learning Representations*. 2025. URL: https://openreview.net/forum?id=uKZdlihDDn.

[10] P. Du et al. "Conditional neural field latent diffusion model for generating spatiotemporal turbulence". In: *Nature Communications* 15.1 (2024), p. 10416. DOI: 10.1038/s41467-024-54712-1.

[11] A. J. Majda and A. L. Bertozzi. *Vorticity and Incompressible Flow*. Cambridge Texts in Applied Mathematics. Cambridge University Press, 2001. DOI: 10.1017/CBO9780511613203.

[12] L. D. Landau and E. M. Lipschitz. *Fluid Mechanics, 2nd edition*. Butterworth Heinemann, 1987.

[13] C. M. Dafermos. *Hyperbolic conservation laws in continuum physics*. Vol. 3. Springer, 2005.

[14] U. S. Fjordholm, S. Mishra, and E. Tadmor. "On the computation of measure-valued solutions". In: *Acta Numerica* 25 (2016), pp. 567–679. DOI: 10.1017/S0962492916000088.

[15] U. S. Fjordholm et al. "Construction of approximate entropy measure-valued solutions for hyperbolic systems of conservation laws". In: *Foundations of Computational Mathematics* 17.3 (2017), pp. 763–827. DOI: 10.1007/s10208-015-9299-z.

[16] S. Lanthaler, S. Mishra, and C. Parés-Pulido. "Statistical solutions of the incompressible Euler equations". In: *Mathematical Models and Methods in Applied Sciences* 31.02 (2021), pp. 223–292. ISSN: 1793-6314. DOI: 10.1142/s0218202521500068.

[17] U. S. Fjordholm et al. "Statistical solutions of hyperbolic systems of conservation law: Numerical approximation". In: *Mathematical Models and Methods in Applied Sciences* 30.3 (2020), pp. 539–609. DOI: 10.1142/S0218202520500141.

[18] C. Foias et al. *Navier-Stokes Equations and Turbulence*. Encyclopedia of Mathematics and its Applications. Cambridge University Press, 2001. DOI: 10.1017/CBO9780511546754.

[19] U. S. Fjordholm, S. Lanthaler, and S. Mishra. "Statistical Solutions of Hyperbolic Conservation Laws: Foundations". In: *Archive for Rational Mechanics and Analysis* 226.2 (2017), pp. 809–849. ISSN: 1432-0673. DOI: 10.1007/s00205-017-1145-9.

[20] U. S. Fjordholm, S. Mishra, and F. Weber. "On the Vanishing Viscosity Limit of Statistical Solutions of the Incompressible Navier–Stokes Equations". In: *SIAM Journal on Mathematical Analysis* 56.4 (2024), pp. 5099–5143. DOI: 10.1137/23M1566261.

[21] J. Ho, A. Jain, and P. Abbeel. "Denoising diffusion probabilistic models". In: *Proceedings of the 34th International Conference on Neural Information Processing Systems*. NIPS '20. Vancouver, BC, Canada: Curran Associates Inc., 2020. ISBN: 9781713829546.

[22] P. Vincent. "A connection between score matching and denoising autoencoders". In: *Neural Computation* 23.7 (2011), pp. 1661–1674. DOI: 10.1162/NECO_a_00142.

[23] Y. Song and S. Ermon. "Generative modeling by estimating gradients of the data distribution". In: *Proceedings of the 33rd International Conference on Neural Information Processing Systems*. Red Hook, NY, USA: Curran Associates Inc., 2019.

[24] Y. Song et al. *Score-Based Generative Modeling through Stochastic Differential Equations*. 2021. DOI: 10.48550/arXiv.2011.13456. arXiv: 2011.13456 [cs.LG]. URL: https://arxiv.org/abs/2011.13456.

[25] W. Tang and H. Zhao. *Score-based Diffusion Models via Stochastic Differential Equations – a Technical Tutorial*. 2024. DOI: 10.48550/arXiv.2402.07487. arXiv: 2402.07487 [cs.LG]. URL: https://arxiv.org/abs/2402.07487.

[26] T. Karras et al. "Elucidating the Design Space of Diffusion-Based Generative Models". In: *Advances in Neural Information Processing Systems*. Ed. by S. Koyejo et al. Vol. 35. Curran Associates, Inc., 2022, pp. 26565–26577. URL: https://proceedings.neurips.cc/paper_files/paper/2022/file/a98846e9d9cc01cfb87eb694d946ce6b-Paper-Conference.pdf.

[27] G. Batzolis et al. *Conditional Image Generation with Score-Based Diffusion Models*. 2021. DOI: 10.48550/arXiv.2111.13606. arXiv: 2111.13606 [cs.LG]. URL: https://arxiv.org/abs/2111.13606.

[28] M. Herde et al. *Poseidon: Efficient Foundation Models for PDEs*. 2024. DOI: 10.48550/arXiv.2405.19101. arXiv: 2405.19101 [cs.LG]. URL: https://arxiv.org/abs/2405.19101.

[29] C. Saharia et al. "Photorealistic Text-to-Image Diffusion Models with Deep Language Understanding". In: *Advances in Neural Information Processing Systems*. Ed. by S. Koyejo et al. Vol. 35. Curran Associates, Inc., 2022, pp. 36479–36494. URL: https://proceedings.neurips.cc/paper_files/paper/2022/file/ec795aeadae0b7d230fa35cbaf04c041-Paper-Conference.pdf.

[30] O. Ronneberger, P. Fischer, and T. Brox. "U-Net: Convolutional Networks for Biomedical Image Segmentation". In: *Medical Image Computing and Computer-Assisted Intervention – MICCAI 2015*. Ed. by N. Navab et al. Cham: Springer International Publishing, 2015, pp. 234–241. ISBN: 978-3-319-24574-4. DOI: 10.1007/978-3-319-24574-4_28.

[31] J. Ho et al. *Axial Attention in Multidimensional Transformers*. 2019. DOI: 10.48550/arXiv.1912.12180. arXiv: 1912.12180 [cs.CV]. URL: https://arxiv.org/abs/1912.12180.

[32] Z. Li et al. *Fourier Neural Operator for Parametric Partial Differential Equations*. 2021. DOI: 10.48550/arXiv.2010.08895. arXiv: 2010.08895 [cs.LG]. URL: https://arxiv.org/abs/2010.08895.

[33] B. Raonic et al. *GenCFD: Representative samples from each dataset*. Zenodo, July 2026. DOI: 10.5281/zenodo.21340739. URL: https://doi.org/10.5281/zenodo.21340739.

[34] G. I. Taylor and A. E. Green. "Mechanism of the Production of Small Eddies from Large Ones". In: *Proceedings of the Royal Society of London. Series A, Mathematical and Physical Sciences* 158.895 (1937), pp. 499–521. ISSN: 00804630. DOI: 10.1098/rspa.1937.0036.

[35] T. Rohner and S. Mishra. "Efficient Computation of Large-Scale Statistical Solutions to Incompressible Fluid Flows". In: *Proceedings of the Platform for Advanced Scientific Computing Conference*. PASC '24. ACM, June 2024. DOI: 10.1145/3659914.3659922.

[36] R. J. LeVeque. *Numerical methods for conservation laws*. Vol. 3. Springer, 1992.

[37] S. Mishra, C. Schwab, and J. Šukys. "Multi-level Monte Carlo finite volume methods for nonlinear systems of conservation laws in multi-dimensions". In: *Journal of Computational Physics* 231.8 (2012), pp. 3365–3388. ISSN: 0021-9991. DOI: 10.1016/j.jcp.2012.01.011.

[38] K. O. Lye. "Computation of statistical solutions of hyperbolic systems of conservation laws". PhD thesis. ETH Zurich, 2020.

[39] A. Kummerländer et al. "Optimization of Single Node Load Balancing for Lattice Boltzmann Methods on Heterogeneous High Performance Computers". In: *Available at SSRN* (2024). DOI: 10.2139/ssrn.4713497. URL: https://ssrn.com/abstract=4713497.

[40] M. J. Krause et al. "OpenLB—Open source lattice Boltzmann code". In: *Computers & Mathematics with Applications* 81 (2021), pp. 258–288. DOI: 10.1016/j.camwa.2020.04.033. URL: https://www.sciencedirect.com/science/article/pii/S0898122120301875.

[41] A. Kummerländer et al. *OpenLB Release 1.7: Open Source Lattice Boltzmann Code*. Version 1.7.0. Feb. 2024. DOI: 10.5281/zenodo.10684609. URL: https://doi.org/10.5281/zenodo.10684609.

[42] A. Kummerländer et al. "Advances in Computational Process Engineering using Lattice Boltzmann Methods on High Performance Computers". In: *High Performance Computing in Science and Engineering '22*. Ed. by W. E. Nagel, D. H. Kröner, and M. M. Resch. Cham: Springer Nature Switzerland, 2024, pp. 233–247. ISBN: 978-3-031-46870-4. DOI: 10.1007/978-3-031-46870-4_16.

[43] P. P. Sullivan and E. G. Patton. "The Effect of Mesh Resolution on Convective Boundary Layer Statistics and Structures Generated by Large-Eddy Simulation". In: *Journal of the Atmospheric Sciences* 68.10 (2011), pp. 2395–2415. DOI: 10.1175/JAS-D-10-05010.1.

[44] C.-H. Moeng and P. P. Sullivan. "A Comparison of Shear- and Buoyancy-Driven Planetary Boundary Layer Flows". In: *Journal of the Atmospheric Sciences* 51.7 (1994). Publisher: American Meteorological Society Section: Journal of the Atmospheric Sciences, pp. 999–1022. ISSN: 0022-4928, 1520-0469. DOI: 10.1175/1520-0469(1994)051<0999:ACOSAB>2.0.CO;2. URL: https://journals.ametsoc.org/view/journals/atsc/51/7/1520-0469_1994_051_0999_acosab_2_0_co_2.xml (visited on 10/14/2024).

[45] T. Schneider et al. "Climate goals and computing the future of clouds". In: *Nature Climate Change* 7.1 (2017), pp. 3–5. ISSN: 1758-6798. DOI: 10.1038/nclimate3190. URL: https://www.nature.com/articles/nclimate3190 (visited on 04/19/2023).

[46] O. Pauluis. “Thermodynamic Consistency of the Anelastic Approximation for a Moist Atmosphere”. In: *Journal of the Atmospheric Sciences* 65.8 (2008), pp. 2719–2729. ISSN: 0022-4928, 1520-0469. DOI: 10.1175/2007JAS2475.1. URL: https://journals.ametsoc.org/view/journals/atsc/65/8/2007jas2475.1.xml (visited on 02/03/2023).

[47] K. G. Pressel et al. “Large-eddy simulation in an anelastic framework with closed water and entropy balances”. In: *Journal of Advances in Modeling Earth Systems* 7.3 (2015), pp. 1425–1456. ISSN: 1942-2466. DOI: 10.1002/2015MS000496. URL: https://onlinelibrary.wiley.com/doi/abs/10.1002/2015MS000496 (visited on 02/03/2023).

[48] K. G. Pressel et al. “Numerics and subgrid-scale modeling in large eddy simulations of stratocumulus clouds”. In: *Journal of Advances in Modeling Earth Systems* 9.2 (2017), pp. 1342–1365. ISSN: 1942-2466. DOI: 10.1002/2016MS000778. URL: https://onlinelibrary.wiley.com/doi/abs/10.1002/2016MS000778 (visited on 06/12/2023).

[49] A. Nichol and P. Dhariwal. *Improved Denoising Diffusion Probabilistic Models*. 2021. DOI: 10.48550/arXiv.2102.09672. arXiv: 2102.09672 [cs.LG]. URL: https://arxiv.org/abs/2102.09672.

[50] L. Feng, L. Zhang, and C. H. Lai. *Optimal Machine Intelligence at the Edge of Chaos*. 2020. DOI: 10.48550/arXiv.1909.05176. arXiv: 1909.05176 [cs.LG]. URL: https://arxiv.org/abs/1909.05176.

[51] L. Zhang et al. *Edge of chaos as a guiding principle for modern neural network training*. 2021. DOI: 10.48550/arXiv.2107.09437. arXiv: 2107.09437 [cs.LG]. URL: https://arxiv.org/abs/2107.09437.

[52] N. Rahaman et al. *On the Spectral Bias of Neural Networks*. 2019. DOI: 10.48550/arXiv.1806.08734. arXiv: 1806.08734 [stat.ML]. URL: https://arxiv.org/abs/1806.08734.

[53] R. Pascanu, T. Mikolov, and Y. Bengio. “On the Difficulty of Training Recurrent Neural Networks”. In: *Proceedings of the 30th International Conference on Machine Learning*. Vol. 28. ICML’13. Atlanta, GA, USA: JMLR.org, 2013, III–1310–III–1318.

[54] J. Hadamard. *Lectures on Cauchy’s problem in linear partial differential equations*. New Haven Yale University Press, 1923.

[55] F. Bartolucci et al. *Representation Equivalent Neural Operators: a Framework for Alias-free Operator Learning*. 2023. DOI: 10.48550/arXiv.2305.19913. arXiv: 2305.19913 [cs.LG]. URL: https://arxiv.org/abs/2305.19913.

[56] U. Frisch. *Turbulence: The Legacy of A.N. Kolmogorov*. Cambridge University Press, 1995. ISBN: 9780521451031.

[57] S. Ghoshal. “An analysis of numerical errors in large eddy simulations of turbulence”. In: *Journal of Computational Physics* 125.1 (1996), pp. 187–206. DOI: 10.1006/jcph.1996.0088.

[58] M. Bonavita. “On Some Limitations of Current Machine Learning Weather Prediction Models”. In: *Geophysical Research Letters* 51.12 (2024), e2023GL107377. DOI: 10.1029/2023GL107377.

[59] J. Kaplan et al. *Scaling Laws for Neural Language Models*. 2020. DOI: 10.48550/arXiv.2001.08361. arXiv: 2001.08361 [cs.LG]. URL: https://arxiv.org/abs/2001.08361.

[60] R. Rombach et al. “ High-Resolution Image Synthesis with Latent Diffusion Models ”. In: *2022 IEEE/CVF Conference on Computer Vision and Pattern Recognition (CVPR)*. Los Alamitos, CA, USA: IEEE Computer Society, June 2022, pp. 10674–10685. DOI: 10.1109/CVPR52688.2022.01042.

[61] S. Lang et al. *AIFS-CRPS: Ensemble forecasting using a model trained with a loss function based on the Continuous Ranked Probability Score*. 2024. DOI: 10.48550/arXiv.2412.15832. arXiv: 2412.15832 [physics.ao-ph]. URL: https://arxiv.org/abs/2412.15832.
