# Supplementary material for: Generative AI for efficient statistical computation of fluids
Source: Nat Commun. 2026 Aug 17;17:9846. doi: 10.1038/s41467-026-76390-x (PMC13578293; doi:10.1038/s41467-026-76390-x)
Supplement: Supplementary file 2 [file 41467_2026_76390_MOESM2_ESM.pdf]

nature portfolio

Peer Review File

Generative AI for fast and accurate statistical computation of fluids

Corresponding Author: Dr Leonardo Zepeda-Núñez

This file contains all reviewer reports in order by version, followed by all author rebuttals in order by version.

Attachments originally included by the reviewers as part of their assessment can be found at the end of this file.

Version 0:

Reviewer comments:

Reviewer #1

(Remarks to the Author)
While the paper presents a compelling framework (GenCFD) for statistical computation of turbulent flows, its validation and claims have some shortcomings:

1. By comparing a probabilistic model against deterministic ones, the paper's demonstration of superiority is unsurprising. It should be compared against other probabilistic models - for example, ones trained using a CRPS loss (https://arxiv.org/abs/2412.15832, https://arxiv.org/abs/2507.12144) The CRPS loss-based training shows promise as a cheaper way of training probabilistic models compared to diffusion. Comparisons can be quantified using metrics like Spread-Skill ratio along with CRPS to gauge the ensemble prediction quality.

2. It should also be compared against other baselines that aim to preserve invariant measures like MMD loss (https://arxiv.org/abs/2402.04467) and the BSP loss (https://arxiv.org/abs/2502.00472). A key feature of learning the distribution (or in some representation invoking the arrangement of scales in wavenumber space) is the ability to preserve invariant measures in very long forecast horizons. This needs to be explored as done in some of the mentioned papers. Using an MSE-based training is no longer a very competitive baseline.

3. I would recommend some conservatism in the statement that GenCFD is orders of magnitude faster than the CFD solver. This is not an apples to apples comparison - the solver generalizes to completely unseen geometries and physics, where any ML model would need retraining/fine-tuning.

4. I really enjoyed the discussion about the theoretical justification against simple MSE-based models for dynamical systems exhibiting deterministic chaos. Thank you for this. Could this theoretical justification also be fused with point 2 above? I think a study where additional loss terms eventually show up as a recovery of the invariant property, in expectation, would be a very nice contribution to the community.

(Remarks on code availability)

Reviewer #2

(Remarks to the Author)
Plz see pdf file attached

(Remarks on code availability)

Reviewer #3

(Remarks to the Author)

The experiments and studies of GenCFD show some exciting results, as it is proving that conditional score-based diffusion models were able to simulate three-dimensional fluids even given the statistical complexity, validified by the toy problem experiment you deliberately designed to present its overall ability in simulating fluid dynamics, with astonishing low cost GPU, fast training and inference time, and quick experiment turnaround. I've been working with a similar diffusion model for Earth systems and had not too much experience with CFD. So I am curious in the discussion of the DL model approach GenCFD utilized.

The paper is well structured to show why conditioned diffusion model approached is chosen given it is ability to incorporate conditional input and specific optimizations to make it even suitable for the case of UQ, such as refinement of loss function, using of UViT instead of traditional UNet for denoiser.

There is good amount of ablation study in the paper, enough to show that score-based diffusion model is a good approach (UViT vs GenCFD for deterministic vs indeterministic). However, there was lots of emphasis on the ability of GenCFD to incorporate conditional generation, to match conditional probability distribution, but there is no ablation study to show the skill of inference between GenCFD and an unconditional diffusion model. It was mentioned many times that a conditional inference would "guide" the model to generate not the "average" but more accurate distribution. I've seen this in my work as well, and I firmly believe that is the novelty of GenCFD here, that it shall outperforms an unconditional model, just like in the TG toy problem it outperforms GenCFD* +FNO, which is taking very coarse prediction. Adding that ablation could potentially help strengthen GenCFD.

The trainings of baselines are carefully conducted to ensure they are the best in their kind. Vast hyper parameter search and cross validation were conducted and learning of the model takes enough time for it to be converged. CRPS and 1-Wasserstein distance make evaluation of ensemble prediction easier. The energy spectrum really tells how performance of GenCFD over different frequencies.

The baselines selected are not as competitive as GenCFD is. They are showing that GenCFD is working, even though by visualization and energy spectrum comparing to the ground truth is saying it is working very well. FNO and CFNO are not in the same magnitude of GenCFD, by model size, loss function, architectural complexity or training cost. UViT alone without diffusion architecture is showing an ablation which is also not competitive. There were a few models mentioned in the prior work with competitive enough structure such as latent diffusion model, or GenCFD but use UNet as the denoiser in place of UViT. If this is not viable in the current state, it can be included in future work to further strengthen the novelty of GenCFD.

Afterall, the visualization over different toy problems and the energy spectrum of GenCFD generation looks very convincing vs the ground truth. GenCFD shows a huge potential and road sign that conditional score-based diffusion model can be used to solve robust statistical computation of three-dimensional turbulent fluid flows or others while maintaining accuracy, being artifact free, and with low training and inference cost.

(Remarks on code availability)

Version 1:

Reviewer comments:

Reviewer #1

(Remarks to the Author)
Thank you for addressing my comments. I support the publication of this article.

(Remarks on code availability)

Reviewer #2

(Remarks to the Author)
The reviewer acknowledges the quality of the revised work and suggests acceptance.

(Remarks on code availability)

Reviewer #3

(Remarks to the Author)
Thank you for taking the comments and addressing them in the revised manuscript.
The current unconditional generation as the baseline should be satisfactory, given that the utility of an unconditional model can be constrained and the yield would not worth it. The comparison in Figure 26 is clearly showing that it is outperforming the unconditional ground truth, which should be a better baseline than any unconditional model.
Glad to see the introduction of Latent Diffusion Models in this concept is recognized as a potential future approach. The

implementation task presents itself as an entirely different problem. VAE does need cross channel correlation, which is unsuitable for physical heterogenous CFD fields, however VQ-regularized AE could serve a better role for this purpose while maintaining a continuous latent space for LDM to operate on. The CRPS-metric model should serve a better baseline as it is a convincing metric, and it doesn't pose itself as an entirely different approach.
I am glad to find that the authors have thoroughly addressed all of comments. The revision with the baselines strengthens the manuscript, and I praise the authors for their effort to respond and attention to detail.

(Remarks on code availability)

Open Access This Peer Review File is licensed under a Creative Commons Attribution 4.0 International License, which permits use, sharing, adaptation, distribution and reproduction in any medium or format, as long as you give appropriate credit to the original author(s) and the source, provide a link to the Creative Commons license, and indicate if changes were made.
In cases where reviewers are anonymous, credit should be given to 'Anonymous Referee' and the source.
The images or other third party material in this Peer Review File are included in the article's Creative Commons license, unless indicated otherwise in a credit line to the material. If material is not included in the article's Creative Commons license and your intended use is not permitted by statutory regulation or exceeds the permitted use, you will need to obtain permission directly from the copyright holder.
To view a copy of this license, visit https://creativecommons.org/licenses/by/4.0/

Revision of:

Generative AI for fast and accurate statistical computation of fluids

Roberto Molinaro [1,3,*], Samuel Lanthaler [2,*], Bogdan Raonić [1,4,*], Tobias Rohner [1,*], Victor Armegioiu [1], Stephan Simonis [1,5], Dana Grund [1,6], Yannick Ramic [1], Zhong Yi Wan [7], Fei Sha [7], Siddhartha Mishra [1,4,†], Leonardo Zepeda-Núñez [7,†]

[1] Seminar for Applied Mathematics, D-MATH, ETH Zurich, Switzerland,
[2] University of Vienna, Vienna, Austria,
[3] Jua.ai, Zurich, Switzerland,
[4] ETH AI Center, Zurich, Switzerland,
[5] Karlsruhe Institute of Technology (KIT), 76131 Karlsruhe, Germany,
[6] D-USYS, ETH Zurich, Switzerland,
[7] Google Research, Mountain View, CA 94043, USA,
[*] Equal contribution, [†] Co-corresponding authors.

Manuscript # NCOMMS-25-71316

At the outset, we would like to thank the editor for providing us with the opportunity to be carefully evaluated by the reviewers.

We sincerely thank the reviewers for their thorough evaluation and constructive feedback. Their guidance has demonstrably strengthened the contextualization of our research. Detailed responses to the specific critiques of each reviewer are provided below.

Response to Reviewer #1

We are deeply encouraged that the reviewer found our manuscript to offer a "compelling framework (GenCFD) for statistical computation of turbulent flows" and that they "really enjoyed the discussion about the theoretical justification against simple MSE-based models for dynamical systems exhibiting deterministic chaos." Below, we provide a detailed response to their points of criticism.

Comment 1: *By comparing a probabilistic model against deterministic ones, the paper's demonstration of superiority is unsurprising. It should be compared against other probabilistic models - for example, ones trained using a CRPS loss (https://arxiv.org/abs/2412.15832, https://arxiv.org/abs/2507.12144). The CRPS loss-based training shows promise as a cheaper way of training probabilistic models compared to diffusion. Comparisons can be quantified using metrics like Spread-Skill ratio along with CRPS to gauge the ensemble prediction quality.*

Response: We appreciate this insightful perspective regarding the comparative landscape of probabilistic models. We also acknowledge that CRPS training is being increasingly used for probabilistic neural surrogate predictions, particularly in the AI for weather and climate community.

Given this, we have successfully implemented an AIFS-like model to facilitate an empirical comparison between GenCFD and an AI model (ViT) trained with CRPS loss. The detailed results and discussion are presented in the Section 11.7 of the SI of the revised version of our paper and a summary is provided in the main text (Section 4). From these results, we clearly observe that CRPS training does improve the mean and the statistical spread, when compared to deterministic neural operator baselines; however it is outperformed by GenCFD in terms of statistical accuracy. Table 8 and Figures 7 and 8 in the SI show that GenCFD outperforms this new model in statistical metrics like variance errors and Wasserstein distances. Moreover, this trend holds even when increasing the number of ensemble members in CRPS training (Table 9). Thus, we clearly demonstrate that CRPS modeling is not able to outperform GenCFD at the timescales of prediction that we consider in our paper.

It is critical to demarcate the functional divergence between the respective physical objectives. The vast majority of models optimized via CRPS in the contemporary literature (including the highly effective AIFS-CRPS and FourCastNet 3 frameworks cited by the reviewer) are fundamentally tailored for medium-range weather forecasting and atmospheric downscaling. These architectures operate under the paradigm of a singular ground truth trajectory, targeting next-step predictions where the deterministic forecasting skill remains relatively narrow, and the predictions are within the predictability window (or from the perspective of dynamical system, the model act within the Lyapunov time). On other hand, the GenCFD framework is engineered to compute the complete conditional probability distribution of fluid systems evolving into fully chaotic regimes, i.e., GenCFD works well beyond the predictability window of the given problem (i.e., well beyond the Lyapunov time).

Regarding the recommendation to utilize the Spread-Skill ratio alongside CRPS, we agree that the Spread-Skill ratio is a key metric for gauging ensemble calibration when evaluating predictions against a specific, deterministically known ground truth sequence. However, at the extended temporal horizons evaluated within our study, the fluid solutions transition into a much more chaotic and deterministically unpredictable states. At this juncture, the system exists well beyond the conventional predictability window characteristic of short to medium-range meteorological models.

As such, the skill of the ensemble mean provides negligible signal regarding the physical fidelity of the statistical solution that describes the system. Under these specific fluid dynamic conditions, metrics explicitly and mathematically tailored to quantify the optimal transport cost between probability distributions—such as the Wasserstein metric—are significantly more suitable and informative than

trajectory-centric measurements. By leveraging our framework's capacity to generate extensive ensembles on demand (generating approximately 10,000 samples through initial condition perturbation), we were able to fully and rigorously benchmark the conditional distribution predicted by the diffusion model against the true target distribution.

Nevertheless, we would like to thank the reviewer again for their excellent suggestion to baseline against CRPS training as it significantly strengthens the message of our paper.

Comment 2: *It should also be compared against other baselines that aim to preserve invariant measures like MMD loss (https://arxiv.org/abs/2402.04467) and the BSP loss (https://arxiv.org/abs/2502.00472). A key feature of learning the distribution (or in some representation invoking the arrangement of scales in wavenumber space) is the ability to preserve invariant measures in very long forecast horizons. This needs to be explored as done in some of the mentioned papers. Using an MSE-based training is no longer a very competitive baseline.*

Response: The reviewer highlights a very important question in computational dynamics. We concur entirely that the preservation of spectral consistency and invariant measures over extensive forecast horizons using advanced loss formulations, such as Maximum Mean Discrepancy (MMD) and Binned Spectral Power (BSP), represents a vital objective in the field.

However, these specific losses (and architectures) fundamentally diverge from the primary scope and physical intent of our current manuscript. The GenCFD methodology was designed to explicitly circumvent a key limitation identified in prior architectures (most notably the DySLIM framework which shares co-authorship with the present manuscript): a central limitation of methodologies dependent upon explicit invariant-measure forcing is their restricted ability to accurately compute transient regimes, critical temporal periods during which the system statistics are actively evolving and are strictly out of statistical equilibrium.

The primary scientific question this paper sought to answer was whether an end-to-end conditional score-based diffusion model could autonomously capture this highly complex phase transition from a quasi-deterministic initial state into a fully stochastic, turbulent regime, relying entirely on the learned probabilistic description without the exogenous imposition of batch-dependent, attractor-forcing losses. We believe that GenCFD clearly demonstrates this ability for a very challenging test suite of 5 transient 3D benchmarks encompassing different types of fluid flows, incompressible, compressible and anelastic.

While we agree that exploring hybrid approaches utilizing MMD or BSP is a necessary evolution of the field, introducing them within this manuscript would obscure the fundamental, autonomous capability of the generative model being evaluated.

Comment 3: *I would recommend some conservatism in the statement that GenCFD is orders of magnitude faster than the CFD solver. This is not an apples to apples comparison - the solver generalizes to completely unseen geometries and physics, where any ML model would need retraining/fine-tuning.*

Response: We fully accept this valid critique regarding generalization of physics-based solvers to unseen geometries. Simultaneously, we assert that the computational speedup comparisons provided within our defined experimental scope were constructed to be as equitable and rigorous as physically possible. To actively avoid the "weak baseline" comparison pitfalls extensively documented in recent computational physics literature (specifically, the rigorous analyses demonstrating systemic overoptimism in machine learning for PDEs published by McGreivy and Hakim in Nature Machine Intelligence), our benchmarks utilized state-of-the-art, heavily parallelized, GPU-accelerated pseudo-spectral computational fluid dynamics solvers. The evaluation architectures and software were equivalent to, or strictly superior to, standard implementations, establishing true hardware and algorithmic parity. Furthermore, to provide absolute clarity regarding the purpose of the total computational runtimes we report (inference in terms

of sample generation), we have added a conservative statement to SI Table 22.

Comment 4: *I really enjoyed the discussion about the theoretical justification against simple MSE-based models for dynamical systems exhibiting deterministic chaos. Thank you for this. Could this theoretical justification also be fused with point 2 above? I think a study where additional loss terms eventually show up as a recovery of the invariant property, in expectation, would be a very nice contribution to the community.*

Response: We deeply appreciate the reviewer's validation of the theoretical discourse surrounding the limitations of deterministic optimization in chaotic environments. The prospect of mathematically and architecturally fusing this theoretical justification with the explicit recovery of invariant properties through specialized supplementary loss terms constitutes a highly compelling and exciting direction. We entirely agree that such a holistic integration would deliver a profound contribution to the scientific community. However, owing to the rigorous spatial constraints of the journal and the strictly delineated scope of evaluating score-matching methodologies, this sophisticated architectural fusion is currently reserved as the focus for a dedicated, upcoming study.

Response to Reviewer #2

We are glad that the reviewer found that our paper should be accepted for publication. In addition, we are highly encouraged by the positive aspects of our manuscript mentioned by the reviewer: level of clarity, clean mathematical explanation, complexity and diversity of 3D turbulent benchmarks, code availability, strong performance and high value of GenCFD Besides, the expert guidance on potential limitations of our manuscript provided by the reviewer has demonstrably strengthened the contextualization of our research. Detailed responses to the specific critiques are provided below.

Comment 1: Pros:

- *This work provides a very clear and understandable description of diffusion models in general and GenCFD in particular. Its main strength lies in the simple and rigorous mathematical formulation of the forward and backward SDE steps, as well as the use of Tweedie's formula to obtain a neat and interpretable loss functional. It allows the reader to follow the model without major difficulty, aside from the inherent complexity of the neural network (UViT) itself. It is worth emphasizing that this level of clarity is not common in many ML papers, which are often overloaded with technical acronyms and cross-references. Therefore, this work is noteworthy for its pedagogical qualities.*
- *The authors attempt to give a clean mathematical explanation for the performance of their models compared with traditional or baseline approaches by providing an estimate in Wasserstein distance; Proposition S10.3. In this respect, the Supplementary Information contains many useful and interesting results that unfortunately cannot all appear in the main text.*
- *The complexity and diversity of the 3-D turbulent-like benchmarks are also strong points of this work, supported by rigorous and well-organized tests. The detailed descriptions, together with the fact that the authors provide the code on GitHub, reinforce the seriousness and professionalism of this study.*
- *The performance of the model itself is of course one of its major strengths – in particular its low sample-complexity – is highly valuable. The model (not surprisingly) shows outstanding results compared with baseline approaches. However, this raises a number of criticisms related to the way the authors present their model (see below).*

Response: We thank the reviewer for pointing out the value of our work and its strengths.

Comment 2.1 (Better metrics for statistical performance):

Cons:

- *The authors' claim of "statistical fidelity" for 3-D incompressible Navier–Stokes flows is somewhat exaggerated. Their diagnostics focus mainly on the mean and variance of the generated velocity fields. They also present one-point probability distributions and energy spectra. While these diagnostics are mandatory and the model passes them in an impressive way, they remain insufficient to determine whether GenCFD reproduces the essential features of turbulent dynamics.*

  From the quantities shown in this work, it is impossible to assess the realism of the cascade or the small-scale statistics. The model therefore appears somewhat oversold from a turbulence-physics perspective, even though the visual results and the mandatory sanity checks remain impressive.

  A more convincing evaluation would require diagnostics commonly used in the turbulence community. For example, in the Taylor–Green vortex at such low resolution, one expects only a very short inertial interval. Showing a compensated $k^{5/3}E(k)$ spectrum (or equivalently the second-order

structure function) would already be valuable. More robust indicators would include measurements of intermittency through the non-Gaussian tails of velocity increments, and shell-to-shell energy-flux analyses to diagnose the direct cascade. Another relevant diagnostic would be the transition from anisotropic behavior at large scales to isotropic behavior at small scales. Note that the reviewer is not asking for a verification of the 4/5 law or anomalous dissipation, as this would require much higher resolutions.

In any case, a discussion of how diffusion models might behave in higher-Reynolds-number regimes would be very interesting.

A second related point concerns incompressibility. The forward diffusion step adds Gaussian noise that is not divergence-free, and the same issue arises for the reverse SDE, so the generated fields are not strictly divergence-free. One could consider modifying the forward and backward dynamics by applying the Leray projection to enforce $\nabla \cdot \mathbf{u} = 0$ *at every step. It is unclear whether the authors' method would improve, or whether the resulting samples would differ significantly, but without testing such a projection—or at least reporting the magnitude of* $\nabla \cdot \mathbf{u}$ *(by scales)—it is difficult to evaluate the physical fidelity of the generated flows. What would be the effect of this lack of incompressibility on the K41 spectrum?*

To further strengthen the impact and usefulness of this work, the reviewer would welcome the addition of a dedicated section addressing this topic in greater depth. Note that the reviewer is not asking for additional experiments at higher data resolution, unless the authors wish to include them, but rather for a comprehensive discussion of whether diffusion models (GenCFD) are able—or not able—to generate realistic turbulent flows.

Response: We are thankful to the reviewer for critically assessing the statistical fidelity of GenCFD for generating 3D incompressible Navier–Stokes flows, specifically from a "turbulence-physics" perspective.

It is clear that GenCFD has to learn all the underlying physics of turbulence from the training data. As the reviewer correctly (and astutely!) points out, as the underlying *ground truth* data is generated at fairly low numerical resolutions of 128^3 (for Taylor–Green and shear flow), the data itself only contains an approximate (coarse) subset of the very rich small-scale phenomena that characterizes the physics of turbulent fluid flows. Hence, it is unrealistic to expect GenCFD to capture the very rich physics of turbulent fluid flows.

Nevertheless, it is still meaningful to examine what exactly can be captured by GenCFD, at least with respect to the training data. As the reviewer has already observed, GenCFD already excels at approximating the 1-point statistics of the training data very well, with low errors for the mean, variance and Wasserstein distance of 1-point velocity pdfs. We had also added errors for third, fourth and fifth-order central moments in SI Table 17 and Figure 33 and show that GenCFD provides very accurate approximations of high-order moments of the numerical data.

However, the reviewer is correct in pointing out that 1-point statistics does not necessarily characterize the rich physics of turbulent flows and 2-point statistics are necessary to characterize correct energy cascades from large to small scales, particularly for fully developed homogeneous isotropic turbulence. We had already considered the approximation of the energy spectra in the SI Figure 7 of our paper and shown how well GenCFD approximates the spectra of the data generated by the numerical methods for each of the five flows that he have considered in the paper. The energy spectra are related to the second-order structure function, showing that this particular 2-point statistic of the simulated flow can also be accurately approximated. However, as the reviewer correctly pointed out, the inertial range at this level of resolution was indeed small.

Following the comments of the reviewer, we sought to further stress test our method, and examine if

GenCFD can approximate the third-order longitudinal structure function of the numerical data. We present the results in SI Section 11.9 of our revised manuscript. Following SI Figure 9 we find a surprising agreement between GenCFD and the numerical data in the slope of this key quantity at small-scales. Moreover, we also show that this slope converges as more training steps are used to train GenCFD, indicating that GenCFD is able to accurately approximate the rich small-scale physics of turbulent flows, at the resolutions of the training data that we have considered here. We believe that this will hold even for higher-resolution numerical data but as the reviewer has pointed out, testing this hypothesis is clearly very compute intensive and outside the scope of this paper.

Summarizing, the reviewer has raised a very important point, as such we have added a full section in the SI to address it. We have also summarized these results in the main text, while explicitly acknowledging that the physical fidelity of GenCFD is necessarily limited by the resolution of its training data.

The second point of the reviewer is regarding whether the flows generated by GenCFD are divergence-free or not, reflecting the incompressible nature of the underlying fluids. Moreover, reviewer's suggestion of projecting the generated flow fields into the space of divergence-free fields is excellent, as the velocity fields generated by GenCFD are not explicitly enforced to be divergence-free. Following the reviewer's suggestion, we implemented a very simple Leray projection in Fourier space (see SI Section 11.8 of the revised version for details) of the generated fields, which yields divergence-free fields. Nevertheless, following SI Table 10 for we found that the statistical properties (as well as sample quality) of the generated fields are almost identical to their divergence-free projections. These results clearly demonstrate that although incompressibility is not directly enforced in the generated velocity fields, it does not induce any noticeable difference in the statistical behavior of the approximated distributions. Moreover, if desired, divergence-free fields can be recovered from the generated fields by applying numerical projection operators, without leading to any deterioration in the statistical accuracy of the resulting velocity fields.

Thus, we have added two additional sections SI 11.8 and 11.9 to address these very valid concerns of the reviewer and hope to have done so to their satisfaction.

Comment 3 (Explanation of the performance):

- *The explanation for the performance of diffusion models over baseline "regression" approaches is discussed in depth and provides valuable insights. However, this is (1) not surprising and (2) misses the fundamental reason why this improvement occurs.*

  Let $(S_t)_{t\geq 0}$ be the semigroup associated with the underlying PDE, and let $\bar{u} + du$, with $du \ll 1$, be the initial condition. The goal is to learn $u \coloneqq S_t(\bar{u} + du)$.

  In method A (as clearly explained by the authors), one minimizes $\mathbb{E}_{du}[\|N_\theta(\bar{u} + du) - u\|^2]$. Since $N_\theta(\bar{u} + du) \approx N_\theta(\bar{u})$ by the insensitivity hypothesis, the argmin is approximately $\mathbb{E}[u|\bar{u}]$. Thus, the estimator produces a mean-flow image of $u = S_t(\bar{u} + du)$, drastically reducing the Variance.

  In method B, one minimizes $\mathbb{E}_\eta\mathbb{E}_{du}[\|N_\theta(u + \eta; \bar{u} + du) - u\|^2]$, which, under the same insensitivity hypothesis for N_θ, gives approximately $E[u|U = u + \eta, \bar{u}]$.

  This leads to radically different interpretations:

  > ***method A** cannot represent any turbulent spread because the mapping $\bar{u} \to S_t\bar{u}$ behaves as a discontinuous map (the PDE is effectively ill-posed), and the object to approximate is measure-like. As a result, training collapses to a mean condensate.*
  >
  > ***method B** learns the score ∇logp through Tweedie's formula up to a shift, namely the drift of the backward SDE. Instead of working at the level of trajectories/states (at time t), the neural network works at the level of measures. Even if the full probability p is not recovered,*

the score is largely insensitive to small perturbations of the initial condition (as the authors implicitly wrote in several places). Therefore, rather than learning individual solutions, method B aims at capturing the full ensemble. Individual solutions are then obtained by integrating the SDE backward once training is complete. Method B is therefore inherently superior, since it captures a measure-theoretic object ($\nabla \log p$) that is smooth (and well posed).

In some sense, method A attempts to learn the deterministic (ill-posed) semigroup. Note also (see Additional Remarks) that this extreme sensitivity is a consequence of chaos (as is likely the case for the benchmarks), but in more extreme regimes with larger Re, an even more radical phenomenon occurs: spontaneous stochasticity, where S_t ceases to be a function and a fully measure-theoretic description becomes necessary at a very fundamental level. This largely justifies the use of diffusion models in CFD in both situations.

The take-home message of this work could thus be expressed more clearly and concisely, without being buried under technical details.

Response: We sincerely thank the reviewer for this detailed re-interpretation of what our approach does. The reviewer clearly nails the essence of our argument but in different words. We have made changes in the phrasing in Section 6, also see comments about Spontaneous Stochasticity below, in order to rephrase our theoretical understanding to be more consistent with the reviewer's interpretation.

Comment 4: *Overall, the reviewer believes that this paper should be accepted, provided the revisions suggested above are addressed. More detailed remarks and suggestions are also given below.*

Response:
We thank the reviewer again for their positive evaluation of our paper and hope that their main concerns have been addressed to their satisfaction.

p.2-l37. Here and in many other places, the authors do not seem to be aware of the phenomenon of spontaneous stochasticity, where truly intrinsic stochasticity emerges from a deterministic system in the inviscid limit (e.g., $Re \to \infty$ for 3D Navier–Stokes) provided the inviscid system (e.g., Euler) is ill-posed. Its characteristics manifest even at finite Reynolds numbers as extreme sensitivity, with Lyapunov exponents typically very large and blowing up in the inviscid limit. This phenomenon is tied to the lack of regularity (non-Lipschitz behavior) in typical CFD flows. It was first identified by Lorenz (1969) and later studied from a Lagrangian viewpoint in the 2000s (Gawedzki et al.), and it has recently emerged as an important topic in turbulence and other PDE studies. In this sense, justifying intrinsic stochasticity is straightforward, but the authors should mention at some point (Eulerian) spontaneous stochasticity.

Response:

We thank the reviewer for bringing this point up, as it was exactly what we wanted to convey. We have incorporated the notion of spontaneous stochasticity in the main text of the revised version, including references to the articles the reviewer has suggested, indicating how the approach of GenCFD aligns with these fundamental ideas.

In S30, l.10: This is not exactly due to the potential ill-posedness of 3D Navier–Stokes, but rather to the high sensitivity that emerges as Re becomes large, for the reason mentioned above (spontaneous stochasticity) that velocity field is non-Lipschitz $1/3$ in the limit (Onsager conjecture/ theorem).

Response:

We thank the reviewer again for raising this point. Here we consider the ill-possedness in the sense of Hadamard, where the a well-posed problem is defined as a problem whose solution exists, is unique and

it depends continuously (or smoothly) with respect to the inputs. As the reviewer correctly states, as the Reynolds number goes to infinity, the systems violate the last condition of well-possedness. We added a small comment to make sure that we refer to this particular definition of ill-possedness including a reference to the seminal work of Jacques Hadamard.

p.4–Eq (1). To be self-consistent with (S12), the authors must either add the term $\dot{s}_\tau/s_\tau u_\tau$ and note that they are using the variance-exploding approach with $s_\tau = 1$, or remove it everywhere. They should also explain why this is beneficial for CFD (see also p.S14-l6 for the VE diffusion scheme). Readers would appreciate this clarification.

Response:

The reviewer is correct, we are using the variance exploding approach as mentioned in S14. We deliberately avoid it in the main manuscript to minimize the notational burden. We have added a footnote with this information.

Tweedie formula p.4–l15. The presentation is unnecessarily complicated. The reasoning (see also S28, l14) is reversed compared to what is expected: the backward SDE requires knowledge of $\nabla u \log p_\tau$, and from Tweedie, this term is exactly $\mathbb{E}[u|u_\tau] - u_\tau\sigma_\tau^2$. Moreover, $\mathbb{E}[u|u_\tau] = \operatorname{argmin}_f \mathbb{E}[\|f(u_\tau) - u\|^2]$, where f is parametrized/replaced by the neural network D_θ with $u_\tau = u + \eta$, $\eta \sim N(0, \sigma)$. Clarifying this would help the reader better understand the choice of the loss function, the backward SDE formulation as well as the sensitivity analysis.

Response:

We thank the reviewer for pointing out this point of confusion. We have now re-written the description. In particular, we hope that the revised version makes it clearer that Tweedie's formula is an identity relating the conditional expectation, $E[u_\tau|\bar{u}]$, to the gradient of the log-probability. As pointed out by the reviewer, the conditional expectation in turn is the true minimizer of the employed diffusion loss.

Fig. 1. There appears to be a typo in panel B: the order seems reversed. u_K should be on the right rather than on the left.

Response:

Thank you for comment. We have addressed this issue in the figure.

S28 l.33. same as main remark above: although both terms (S89 and S90) yield concentration around $S(\bar{u}$, their role is completely different. The first one yields a neural representation of the semigroup, whereas the second is a representation of the drift term in the backward SDE. p.9 l.13 and after: Why use the heavy notation $\bar{u}^\star$ when $\bar{u}$ or u would be sufficient?

Figures 2, S8, S11, S15. The use of histograms to show statistics at a single location (true–GenCFD–C–FNO), combined with the choice of a dark purple color, makes the plots difficult to read. In Fig. 2, the authors should at least mention in the caption that the thin, almost Dirac-like bar corresponds to C–FNO, or reconsider the visual design to improve clarity.

Response:

Thank you for comment. We have addressed this issue in the figure of the main text.

Toy models S9.9.1. The feedforward NNs used to test the toy model have quite large capacity (512) and shallow depth (3). Would a different regime—deeper networks with much smaller capacity—exhibit the same behavior?

Response:

We thank the reviewer for the comment. Indeed, a lower capacity model will showcase the same behavior,

but faster! (i.e., for larger Δ), as it is not able to capture all the high frequency information and it will collapse to the mean. We tried a smaller capacity model first, but in order to avoid a limited capacity argument that would have make the comparison ambiguous, we opted for presenting a model with very high capacity, which was then highly over-trained, in order to cleanly showcase the transition.

The choice of the tent map seems motivated by its solvability and by the possibility of deriving rigorous results (as done in the Supplementary Material). However, one could also consider (non-solvable) simple dynamical flows such as: (1) the Lorenz63 system (chaotic but deterministic), or (2) the SDE $dx_t = x^{1/3}dt + \sqrt{\varepsilon}dW_t$, $x(0) = 0$. *This system becomes genuinely stochastic in the small-noise limit: solutions at a fixed time t become non-unique, and convergence occurs in a measure-theoretic sense to* $\frac{\delta_{+a_t}+\delta_{-a_t}}{2}$ *where* $a_t = (\frac{2}{3})^{3/2}$. *Model (2) is historically very important in turbulence and is a paradigm of Lagrangian Richardson's law. Using such a simple model (in fact, it can be made deterministic by mollifying the non-Lipschitz singularity at* $x = 0$ *in an ad-hoc way) would be appealing to the fundamental turbulence community.* ***This is not mandatory, however.***

Response:

We thank the reviewer for the great suggestions. We agree that studying these systems under the lens of statistical solutions would be of interest. However, we would like to argue that is is outside the scope of (already very long!) paper and should be considered in future work.

Response to Reviewer #3

We thank the Reviewer for their very positive feedback to our manuscript. We are glad that the reviewer finds that "GenCFD shows a **huge potential** and **road sign**" and that our methodology is able to "maintaining accuracy, being artifact free, and with low training and inference cost."

We address their detailed comments below.

Comment 1: *The experiments and studies of GenCFD show some exciting results, as it is proving that conditional score-based diffusion models were able to simulate three-dimensional fluids even given the statistical complexity, validified by the toy problem experiment you deliberately designed to present its overall ability in simulating fluid dynamics, with astonishing low cost GPU, fast training and inference time, and quick experiment turnaround. I've been working with a similar diffusion model for Earth systems and had not too much experience with CFD. So I am curious in the discussion of the DL model approach GenCFD utilized.*

The paper is well structured to show why conditioned diffusion model approached is chosen given it is ability to incorporate conditional input and specific optimizations to make it even suitable for the case of UQ, such as refinement of loss function, using of UViT instead of traditional UNet for denoiser.

Response:
We thank the reviewer again for their positive evaluation of our paper.

Comment 2: *There is good amount of ablation study in the paper, enough to show that score-based diffusion model is a good approach (UViT vs GenCFD for deterministic vs indeterministic). However, there was lots of emphasis on the ability of GenCFD to incorporate conditional generation, to match conditional probability distribution, but there is no ablation study to show the skill of inference between GenCFD and an unconditional diffusion model. It was mentioned many times that a conditional inference would "guide" the model to generate not the "average" but more accurate distribution. I've seen this in my work as well, and I firmly believe that is the novelty of GenCFD here, that it shall outperforms an unconditional model, just like in the TG toy problem it outperforms GenCFD* +FNO, which is taking very coarse prediction. Adding that ablation could potentially help strengthen GenCFD.*

Response:

We thank the reviewer for bringing this point. We could train a unconditional model, however, the utility of such model would be quite limited, as it will be sampling from a very wide distribution, and it would be only able to capture statistics over the full distribution (Following Figure 5, we would be seeking to capture statistics of $\mathcal{S}_\sharp\mu$, and not necessarily $p(u_t, \bar{u})$. However, we completely agree that showcasing the statistical performance of the conditional model with respect to an unconditional generation is important. In some sense this is similar to measuring the behavior of the model to a "climatology". In this case we considered an even more powerful comparison: comparing the our model against the unconditional ground truth, i.e., the $\mathcal{S}_\sharp\mu$, computed with the CFD solver. We compared both against the *conditional ground truth* in Appendix Table 22. We can observe that our methods outperforms the unconditional ground truth by a massive margin. In our opinion, a key reason for this observation is the fact that our (conditional) test distribution is significantly different from the (unconditional) training distribution, making the inference task genuinely OOD, due to this massive distribution shift.

In addition, we have updated Figure 26 in the SI of the revised version, to showcase the same behavior: GenCFD outperforms the unconditional ground-truth when approximating the conditional distribution for this anelastic flow from atmospheric dynamics.

We thank the reviewer again for their excellent suggestion to compare against the unconditional

distribution as it highlights the ability of GenCFD to operate in OOD settings. We have added short descriptions of these observations in the main text of the revised version.

Comment: *The trainings of baselines are carefully conducted to ensure they are the best in their kind. Vast hyper parameter search and cross validation were conducted and learning of the model takes enough time for it to be converged. CRPS and 1-Wasserstein distance make evaluation of ensemble prediction easier. The energy spectrum really tells how performance of GenCFD over different frequencies.*

The baselines selected are not as competitive as GenCFD is. They are showing that GenCFD is working, even though by visualization and energy spectrum comparing to the ground truth is saying it is working very well. FNO and CFNO are not in the same magnitude of GenCFD, by model size, loss function, architectural complexity or training cost. UViT alone without diffusion architecture is showing an ablation which is also not competitive. There were a few models mentioned in the prior work with competitive enough structure such as latent diffusion model, or GenCFD but use UNet as the denoiser in place of UViT. If this is not viable in the current state, it can be included in future work to further strengthen the novelty of GenCFD.

Response:

We thank the reviewer for this comment. The poor performance of the baselines highlights the main thesis of our paper: modeling the distribution rather than individual trajectories captures the underlying statistics much more accurately than trajectory-based models, even when using identical architectures. As the reviewer noted, we invested considerable effort into tuning the baselines to ensure a fair comparison. Indeed, our author list includes co-authors of the original CNO paper, as well as researchers with extensive experience training FNOs.

We also appreciate the suggestion to compare against Latent Diffusion Models (LDMs). The primary advantage of LDMs is computational efficiency, achieved by running the diffusion process in a compressed latent space. However, constructing this latent space requires training a variational autoencoder (VAE), which is non-trivial in this context. To ensure accurate physical reconstructions, the VAE must capture field statistics across components (e.g., velocity, energy, density) that are far less correlated than RGB channels in natural images. Consequently, this approach requires bespoke architectural choices for each distinct example presented in this paper. While exploring LDMs could yield further computational gains, a proper implementation falls outside the scope of the current work. We have added a discussion on LDMs to Section 11.6 of the SI, to highlight this research direction.

Finally, at the request of Reviewer 1, we introduced a new baseline utilizing Continuous Ranked Probability Score (CRPS) training, a technique increasingly common in the medium-range probabilistic weather forecasting community. These new results, included in Section 11.7 of the revised SI, demonstrate that GenCFD significantly outperforms CRPS-trained models, further strengthening our baseline comparisons.

Review of NCOMMS-25-71316
Generative AI for fast and accurate statistical computation of fluids, R.Molinaro et al.

December 9, 2025

Pros

- This work provides a very clear and understandable description of diffusion models in general and GenCFD in particular. Its main strength lies in the simple and rigorous mathematical formulation of the forward and backward SDE steps, as well as the use of Tweedie's formula to obtain a neat and interpretable loss functional. It allows the reader to follow the model without major difficulty, aside from the inherent complexity of the neural network (UViT) itself. It is worth emphasizing that this level of clarity is not common in many ML papers, which are often overloaded with technical acronyms and cross-references. Therefore, this work is noteworthy for its pedagogical qualities.
- The authors attempt to give a clean mathematical explanation for the performance of their models compared with traditional or baseline approaches by providing an estimate in Wasserstein distance; Proposition S10.3. In this respect, the Supplementary Information contains many useful and interesting results that unfortunately cannot all appear in the main text.
- The complexity and diversity of the 3-D turbulent-like benchmarks are also strong points of this work, supported by rigorous and well-organized tests. The detailed descriptions, together with the fact that the authors provide the code on GitHub, reinforce the seriousness and professionalism of this study.
- The performance of the model itself is of course one of its major strengths – in particular its low sample-complexity – is highly valuable. The model (not surprisingly) shows outstanding results compared with baseline approaches. However, this raises a number of criticisms related to the way the authors present their model (see below).

Cons

- The authors' claim of "statistical fidelity" for 3-D incompressible Navier–Stokes flows is somewhat exaggerated. Their diagnostics focus mainly on the mean and variance of the generated velocity fields. They also present one-point probability distributions and energy spectra. While these diagnostics are mandatory and the model passes them in an impressive way, they remain insufficient to determine whether GenCFD reproduces the essential features of turbulent dynamics.

  From the quantities shown in this work, it is impossible to assess the realism of the cascade or the small-scale statistics. The model therefore appears somewhat oversold from a turbulence-physics perspective, even though the visual results and the mandatory sanity checks remain impressive.

  A more convincing evaluation would require diagnostics commonly used in the turbulence community. For example, in the Taylor–Green vortex at such low resolution, one expects only a

very short inertial interval. Showing a compensated $k^{5/3}E(k)$ spectrum (or equivalently the second-order structure function) would already be valuable. More robust indicators would include measurements of intermittency through the non-Gaussian tails of velocity increments, and shell-to-shell energy-flux analyses to diagnose the direct cascade. Another relevant diagnostic would be the transition from anisotropic behavior at large scales to isotropic behavior at small scales. Note that the reviewer is not asking for a verification of the 4/5 law or anomalous dissipation, as this would require much higher resolutions.

In any case, a discussion of how diffusion models might behave in higher-Reynolds-number regimes would be very interesting.

A second related point concerns incompressibility. The forward diffusion step adds Gaussian noise that is not divergence-free, and the same issue arises for the reverse SDE, so the generated fields are not strictly divergence-free. One could consider modifying the forward and backward dynamics by applying the Leray projection to enforce $\nabla \cdot u = 0$ at every step. It is unclear whether the authors' method would improve, or whether the resulting samples would differ significantly, but without testing such a projection—or at least reporting the magnitude of $\nabla \cdot u$ (by scales)—it is difficult to evaluate the physical fidelity of the generated flows. What would be the effect of this lack of incompressibility on the K41 spectrum?

To further strengthen the impact and usefulness of this work, the reviewer would welcome the addition of a dedicated section addressing this topic in greater depth. Note that the reviewer is not asking for additional experiments at higher data resolution, unless the authors wish to include them, but rather for a comprehensive discussion of whether diffusion models (GenCFD) are able—or not able—to generate realistic turbulent flows.

- The explanation for the performance of diffusion models over baseline "regression" approaches is discussed in depth and provides valuable insights. However, this is (1) not surprising and (2) misses the fundamental reason why this improvement occurs.

  Let $(S_t)_{t\geq 0}$ be the semigroup associated with the underlying PDE, and let $\bar{u}+du$, with $du \ll 1$, be the initial condition. The goal is to learn $u := S_t(\bar{u} + du)$.

  In method A (as clearly explained by the authors), one minimizes $\mathbb{E}_{du}\left[\|N_\theta(\bar{u} + du) - u\|^2\right]$. Since $N_\theta(\bar{u}+du) \approx N_\theta(\bar{u})$ by the *insensitivity hypothesis*, the argmin is approximately $\mathbb{E}[u \mid \bar{u}]$. Thus, the estimator produces a mean-flow image of $u = S_t(\bar{u} + du)$, drastically reducing the variance.

  In method B, one minimizes $\mathbb{E}_\eta \mathbb{E}_{du}\left[\|N_\theta(u + \eta; \bar{u} + du) - u\|^2\right]$, which, under the same insensitivity hypothesis for N_θ, gives approximately $\mathbb{E}[u \mid U = u + \eta, \bar{u}]$.

  This leads to radically different interpretations:

  > **method A** cannot represent any turbulent spread because the mapping $\bar{u} \mapsto S_t\bar{u}$ behaves as a discontinuous map (the PDE is effectively ill-posed), and the object to approximate is measure-like. As a result, training collapses to a mean condensate.
  >
  > **method B** learns the score $\nabla \log p$ through Tweedie's formula up to a shift, namely the drift of the backward SDE. Instead of working at the level of trajectories/states (at time t), the neural network works at the level of measures. Even if the full probability p is not recovered, the score is largely insensitive to small perturbations of the initial condition (as the authors implicitly wrote in several places). Therefore, rather than learning individual solutions, method B aims at capturing the full ensemble. Individual solutions are then obtained by integrating the SDE backward once training is complete. Method B is therefore inherently superior, since it captures a measure-theoretic object ($\nabla \log p$) that is smooth (and well posed).
  >
  > In some sense, method A attempts to learn the deterministic (ill-posed) semigroup. Note also (see Additional Remarks) that this extreme sensitivity is a consequence of chaos (as is likely the case for the benchmarks), but in more extreme regimes with larger Re,

> an even more radical phenomenon occurs: spontaneous stochasticity, where S_t ceases to be a function and a fully measure-theoretic description becomes necessary at a very fundamental level. This largely justifies the use of diffusion models in CFD in both situations.

The take-home message of this work could thus be expressed more clearly and concisely, without being buried under technical details.

Overall, the reviewer believes that this paper should be accepted, provided the revisions suggested above are addressed. More detailed remarks and suggestions are also given below.

Additional remarks

- *p.2-l37.* Here and in many other places, the authors do not seem to be aware of the phenomenon of spontaneous stochasticity, where truly intrinsic stochasticity emerges from a deterministic system in the inviscid limit (e.g., $Re \to \infty$ for 3D Navier–Stokes) provided the inviscid system (e.g., Euler) is ill-posed. Its characteristics manifest even at finite Reynolds numbers as extreme sensitivity, with Lyapunov exponents typically very large and blowing up in the inviscid limit. This phenomenon is tied to the lack of regularity (non-Lipschitz behavior) in typical CFD flows. It was first identified by Lorenz (1969) and later studied from a Lagrangian viewpoint in the 2000s (Gawedzki et al.), and it has recently emerged as an important topic in turbulence and other PDE studies. In this sense, justifying intrinsic stochasticity is straightforward, but the authors should mention at some point (Eulerian) spontaneous stochasticity.

  In S30, l.10: This is not exactly due to the potential ill-posedness of 3D Navier–Stokes, but rather to the high sensitivity that emerges as Re becomes large, for the reason mentioned above (spontaneous stochasticity) that velocity field is non-Lipschitz 1/3 in the limit (Onsager conjecture/ theorem).

- *p.4–Eq (1).* To be self-consistent with (S12), the authors must either add the term $\dot{s}_\tau/s_\tau u_\tau$ and note that they are using the variance-exploding approach with $s_\tau = 1$, or remove it everywhere. They should also explain why this is beneficial for CFD (see also p.S14-l6 for the VE diffusion scheme). Readers would appreciate this clarification.

- Tweedie formula *p.4–l15.* The presentation is unnecessarily complicated. The reasoning (see also S28, l14) is reversed compared to what is expected: the backward SDE requires knowledge of $\nabla_u \log p_\tau$, and from Tweedie, this term is exactly $\frac{\mathbb{E}[u|u_\tau]-u_\tau}{\sigma_\tau^2}$. Moreover, $\mathbb{E}[u|u_\tau] = \mathrm{argmin}_f \mathbb{E}[\|f(u_\tau) - u\|^2]$, where f is parametrized/replaced by the neural network D_θ with $u_\tau = u + \eta$, $\eta \sim \mathcal{N}(0, \sigma^2)$. Clarifying this would help the reader better understand the choice of the loss function, the backward SDE formulation as well as the sensitivity analysis.

- Fig. 1. There appears to be a typo in panel B: the order seems reversed. u_K should be on the right rather than on the left.

- *S28 l.33.* same as main remark above: although both terms (S89 and S90) yield concentration around $S(\bar{u})$, their role is completely different. The first one yields a neural representation of the semigroup, whereas the second is a representation of the drift term in the backward SDE. *p.9 l.13 and after*: Why use the heavy notation $\bar{u}^\star$ when $\bar{u}$ or $u^\star$ would be sufficient?

- Figures 2, S8, S11, S15. The use of histograms to show statistics at a single location (true–GenCFD–C–FNO), combined with the choice of a dark purple color, makes the plots difficult to read. In Fig. 2, the authors should at least mention in the caption that the thin, almost Dirac-like bar corresponds to C–FNO, or reconsider the visual design to improve clarity.

- *Toy models S9.9.1.* The feedforward NNs used to test the toy model have quite large capacity (512) and shallow depth (3). Would a different regime—deeper networks with much smaller capacity—exhibit the same behavior? The choice of the tent map seems motivated by its

solvability and by the possibility of deriving rigorous results (as done in the Supplementary Material). However, one could also consider (non-solvable) simple dynamical flows such as: (1) the Lorenz63 system (chaotic but deterministic), or (2) the SDE $dx_t = x^{1/3}\,dt + \sqrt{\epsilon}\,dW_t,\ x(0) = 0$. This system becomes genuinely stochastic in the small-noise limit: solutions at a fixed time t become non-unique, and convergence occurs in a measure-theoretic sense to $\frac{\delta_{+a_t} + \delta_{-a_t}}{2}$, where $a_t = \left(\frac{2}{3}t\right)^{3/2}$. Model (2) is historically very important in turbulence and is a paradigm of Lagrangian Richardson's law. Using such a simple model (in fact, it can be made deterministic by mollifying the non-Lipschitz singularity at $x = 0$ in an ad-hoc way) would be appealing to the fundamental turbulence community. This is not mandatory, however.
